# Vestibular nucleus stimulation for ameliorating locomotor dynamics in a Parkinsonian mouse model

**DOI:** 10.1038/s41467-026-76183-2

**Published:** 2026-08-04

**Authors:** Johannes Hartig, Maximilian Uwe Friedrich, Jérémy Signoret-Genest, Maria Gruber, Sawako Tabuchi, Hamidreza Alimohammadi, Max Leuschner, Nina Schöneberg, Robert Peach, Dennis Doll, Tobias Petschner, Amélie Neumann, Susanne Knorr, Jens Volkmann, Philip Tovote, Chi Wang Ip

**Affiliations:** 1 Department of Neurology, University Hospital Wuerzburg, Wuerzburg, Germany; 2 Institute of Clinical Neurobiology, University Hospital Wuerzburg, Wuerzburg, Germany; 3 Center for Mental Health, University Hospital Wuerzburg, Wuerzburg, Germany; 4 Indoc Research Europe gGmbH, Mainz, Germany

**Keywords:** Parkinson's disease, Basal ganglia

## Abstract

Postural and locomotor dysfunction represent axial symptoms of Parkinson’s disease (PD), which remain poorly treated by medication and deep brain stimulation. Whilst non-invasive neuromodulation of the vestibular system via the vestibular nucleus complex (VNC) offers a novel therapeutic avenue, the underlying circuits are still poorly characterized. Here we show that the mouse VNC feeds extensive *Vglut2*-defined projections into striato-thalamo-subthalamic and caudal medulla motor hubs and receives substantial input from the sensorimotor cortex. Optogenetic activation of excitatory VNC neurons at sub-symptomatic intensities increased *cFos*-based activity in basal ganglia-associated and brainstem motor targets. Unbiased pose dynamics and motion analysis respectively showed enhancement of behavioural modularity and locomotion with threshold-level stimulation. In a mouse model of PD, the latter further favored naturalistic gait patterns through improved motor coordination. Our data identify excitatory VNC processes as candidates for therapeutic targeting of axial motor dysfunction in the context of PD.

## Abstract (web summary)

In this work by Hartig et al., inspired by non-invasive stimulation of the vestibular system in humans, the authors use a thresholded optical stimulation paradigm to alleviate symptoms of locomotor dysfunction in a Parkinson’s disease mouse model.

## Introduction

Parkinson’s disease (PD) represents a significant and escalating public health challenge^1^, and is marked by dysfunction in dopaminergic circuits^2^. Increasingly recognized as a neurodegenerative spectrum disorder with a diverse phenotypical combination of motor and non-motor features^3^, PD’s motor aspects - such as bradykinesia, rigidity, and tremor - typically respond well to dopaminergic medication and deep brain stimulation (DBS) targeting the subthalamic nucleus (STN) or globus pallidus internus. However, axial motor symptoms, encompassing dysfunctional gait (e.g., freezing of gait) and postural instability, exhibit poor responsiveness^4–7^ and persist as a clinical concern. Occasionally classified as the PIGD subtype^5^ (postural instability and gait difficulty), axial phenotypes remain both insufficiently understood and inadequately addressed by current therapeutic interventions^8^. Thus, there is an urgent need for innovative strategies to address treatment-resistant parkinsonian gait and posture disorders. Previous approaches towards improving axial PD symptoms have emphasized the potential involvement of basal ganglia-brainstem interactions^9–12^ (see Supplementary Fig. 1 for graphical summary). Specifically, neuromodulation strategies for PIGD have motivated trials of DBS targeting brainstem mesencephalic locomotor region (MLR) nuclei - pedunculopontine (PPN) and cuneiform (CnF) - which yielded inconsistent results^13,14^. This likely reflects the hitherto poorly understood, complex nature of gait and posture (disorders) in PD^8^ and the difficulty of targeting specific MLR pro-locomotor populations identified by cell type-specific means with (electrical) DBS^10,15,16^. Recognizing the advantages of non-invasive neuromodulation techniques in terms of ease-of-use and lower complication rates, there is a growing interest in exploring these approaches across different (patho-)physiological states^17,18^, including PD. However, a prerequisite for better PIGD neuromodulation approaches is a better mechanistic understanding of the circuitry underlying predominantly axial phenotypes.

Vestibular stimulation (VS) is a non-invasive method that utilizes, for example, noisy (i.e., thresholded), galvanic (nGVS) or caloric means to activate (central) vestibular circuits via peripheral excitation of the vestibular organ^19^. This technique has shown promise in mitigating motor and non-motor symptoms of PD^20–23^, potentially by integrating vestibular information for movement into a vestibulo-thalamo-basal ganglia loop^24,25^. Specifically, some data support a positive impact on axial postural deficits in both PD^23,26^ and atypical parkinsonism^27^, yet less so for gait in PD^28^. Furthermore, nGVS has been reported to improve parkinsonian symptoms, specifically locomotion (putatively by enhanced GABA release by the substantia nigra (SN)) in rats^29^. Vestibular circuits likely exhibit glutamatergic predominance^30–32^, especially with regard to motor targets^33^. Although anatomical studies suggest possible circuit mechanisms involving vestibulo-thalamic inputs to basal ganglia loops^24,34,35^, the structural and functional^25^ contributions of vestibular circuits for movement, especially in PD^36^, remain insufficiently understood.

Our study thus aimed to better understand how the vestibular nucleus complex (VNC) feeds into basal ganglia loops and contributes to motor function, specifically in the context of parkinsonian neuropathology. First, we sought to further define the former through viral tracing and functional mapping. Second, based on the hypothesis that thresholded human nGVS feeds pro-locomotor information into basal ganglia loops, we tested whether optical activation of the VNC produces therapeutic effects.

## Results

### Glutamatergic vestibular circuits innervate basal ganglia-associated and brainstem motor hubs

**Fig. 1:**
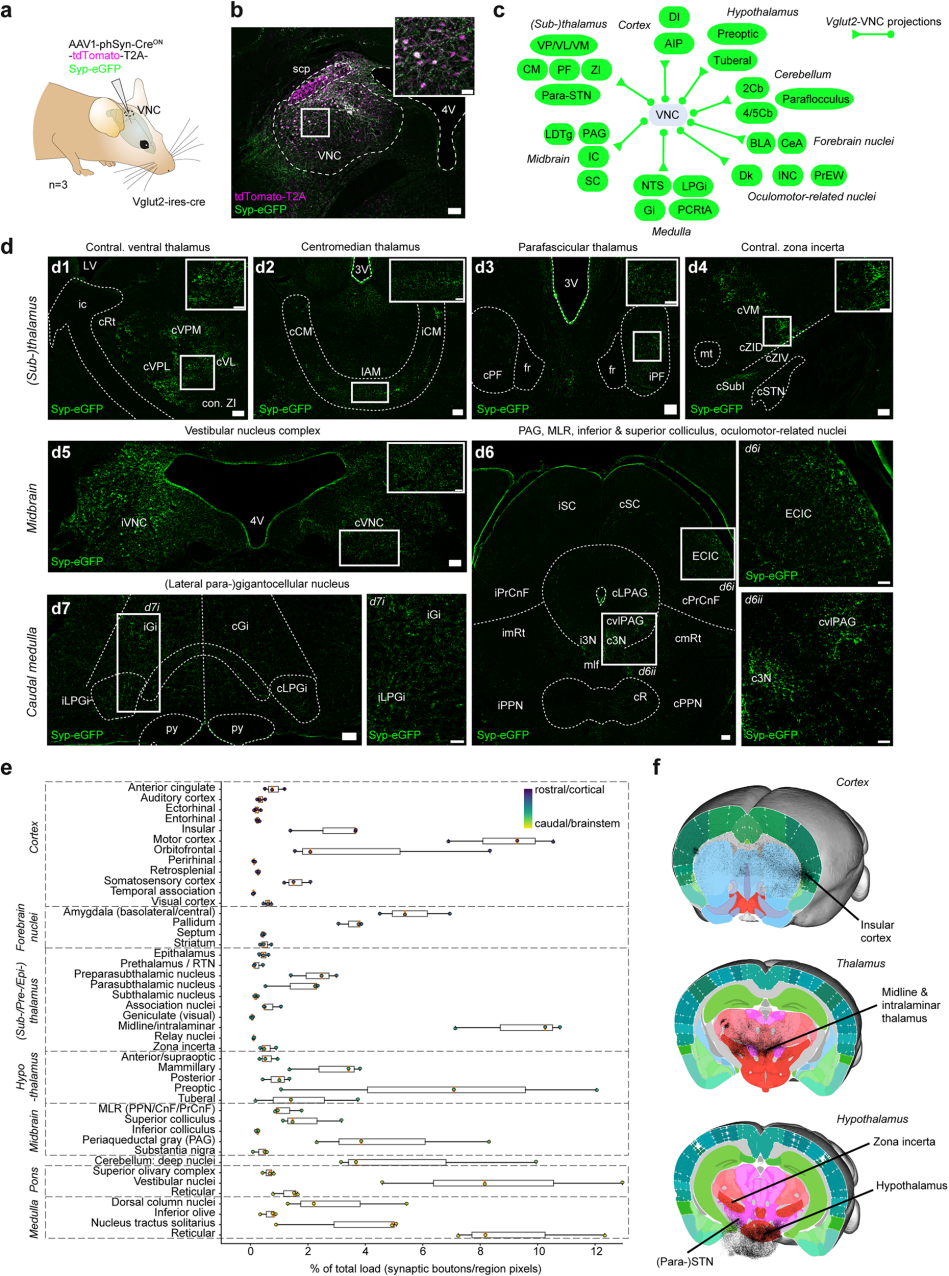
Glutamatergic VNC neurons feed extensive brain-wide projections, including projections to basal ganglia-associated nodes. **a** Schematic illustrating the tracing strategy for the cohort (*n* = 3). **b** Microscopy image depicting the injection site with somatic signal of tdTomato visible in magenta. Scale bars: 200 µm (global image, top), 20 µm (inset, bottom). AP: −5.80 mm from bregma. **c** Wiring diagram summarizing the main Vglut2-VNC targets identified. **d** Set of representative images (see also Supplementary Fig. 2) showing synaptic terminals from the VNC. The ventral thalamic group (d1), centromedian (d2) and parafascicular (d3) thalamic nuclei as well as the subthalamic zona incerta (d4) receive direct VNC projections. The VNC establishes some commissural projections (d5), but only sends sparse projections to the mesencephalic locomotor region (MLR, d6), the superior and inferior colliculi (d6, d6i), and innervates the oculomotor-related nuclei (d6ii), as expected. The VNC further projects into diverse subdivisions of the reticular caudal medulla (d7, d7i). Scale bars: 200 µm (global picture), 20 µm (inset). AP levels (from bregma): d1-d7: −1.34, −1.58, −2.18, −1.94, −5.80, −4.24, −6.00 mm. **e** Unbiased quantification across brain regions reveals targeting of the caudal medulla reticular formation and intralaminar thalamus, but also hypothalamus, (para-)subthalamic nucleus and amygdala (see Supplementary Fig. 2). **f** Exemplary pseudo-3D representations of cortical (top), thalamic (middle) and hypothalamic (bottom) innervation by the VNC (data points from *n* = 3 animals shown). Source data are provided as a Source data file. Micrographs: experiments repeated *n* = 3 times with similar results. Orange lines are medians, boxes are interquartile ranges. Whiskers are most extreme data points. Mouse head drawing adapted from ref. ^77^.

To investigate the (potentially therapeutic) effects of vestibular neuromodulation, we first anatomically examined the underlying circuit elements in mice and sought to determine how the VNC is embedded within movement-related pathways, particularly those interlinking brainstem and basal ganglia^9^. For brain-wide mapping of glutamatergic VNC connectivity, we used an AAV-based strategy to Cre-dependently label Vglut2^+^ neurons in the VNC with two reporters encoded within the same construct (*hSyn-DIO-T2A-tdTomato-SypEGFP*) in *Vglut2*-cre (vesicular glutamate transporter 2-ires-cre*)* mice: a synaptophysin–eGFP fusion protein to mark synaptic boutons and a tdTomato membrane marker to visualize both transfected VNC cells and overall axonal projections (*n* = 3; Fig. 1a, b, and Supplementary Figs. 2 and 3). We then quantified VNC synaptic bouton (eGFP) projection density (Supplementary Fig. 2) across all reference atlas regions with the QUINT workflow (see “Methods”) and generated a comprehensive map of VNC output. Importantly, quantification of tdTomato-positive fibres mainly matched the results from this quantification (Supplementary Fig. 3).

Focusing on subcortical regions related to basal ganglia-brainstem interactions associated with posture and locomotion^9^, we found that the VNC maintains a glutamatergic connectome (Fig. 1c, output wiring diagram), projecting to several important sensorimotor hubs in the thalamus proper, including the ventral (VP/VM/VL, Fig. 1d1), centromedian (CM, Fig. 1d2) and parafascicular (Pf, Fig. 1c,d) nuclei, but also the subthalamus (Fig. 1d4, zona incerta and (pre/para)-subthalamic nucleus; Supplementary Fig. 2A8). Contrary to our initial hypotheses, we found no consistent evidence for a strong projection to the MLR (Fig. 1d6), which we postulated to be regulating gait adjustments. Only some fibres and isolated synapses reached the contra- and ipsilateral PPN; however, few synaptic punctae were found in the MLR (either PPN or CnF).

Notably, we found that the VNC directly targets the reticular formation in the caudal medulla, namely the gigantocellular reticular (Gi) and paragigantocellular (e.g., lateral, LPGi) nuclei (Fig. 1d7), thus potentially exerting influence on reticulospinal effector pathways^12,37^. Consistent with correct injection placement, we observed the expected projections to various oculomotor-related nuclei (Fig. 1d6, and Supplementary Fig. 2A8) and the inferior/superior colliculi. Interestingly, we also identified a relatively isolated cortical projection to the insular and orbitofrontal cortices (Supplementary Fig. 2A8), basolateral/central amygdala (Supplementary Fig. 2A6,8,9) and some terminals in the periaqueductal gray (PAG) (Fig. 1d6).

Additionally, to assess overlaps between VNC projections and motor ensembles that have been implicated in neuromodulation and motor rescue in PD rodents^38,39^, and considering the evidence for a vestibulo-thalamo-striatal tract^24^, we conducted a complementary viral tracing study (Supplementary Fig. 4a). The glutamatergic VNC projection with synaptic terminals in the intralaminar thalamus - within the Pf and CM - were found in proximity with neurons retrogradely labeled from the dorsolateral striatum (Supplementary Fig. 4b–d), supporting the existence of a vestibulo-thalamo-striatal pathway in rodents^24^ via the intralaminar/midline complex.

### Glutamatergic vestibular circuits receive predominant monosynaptic input from sensorimotor cortices, brainstem and cerebellum

**Fig. 2:**
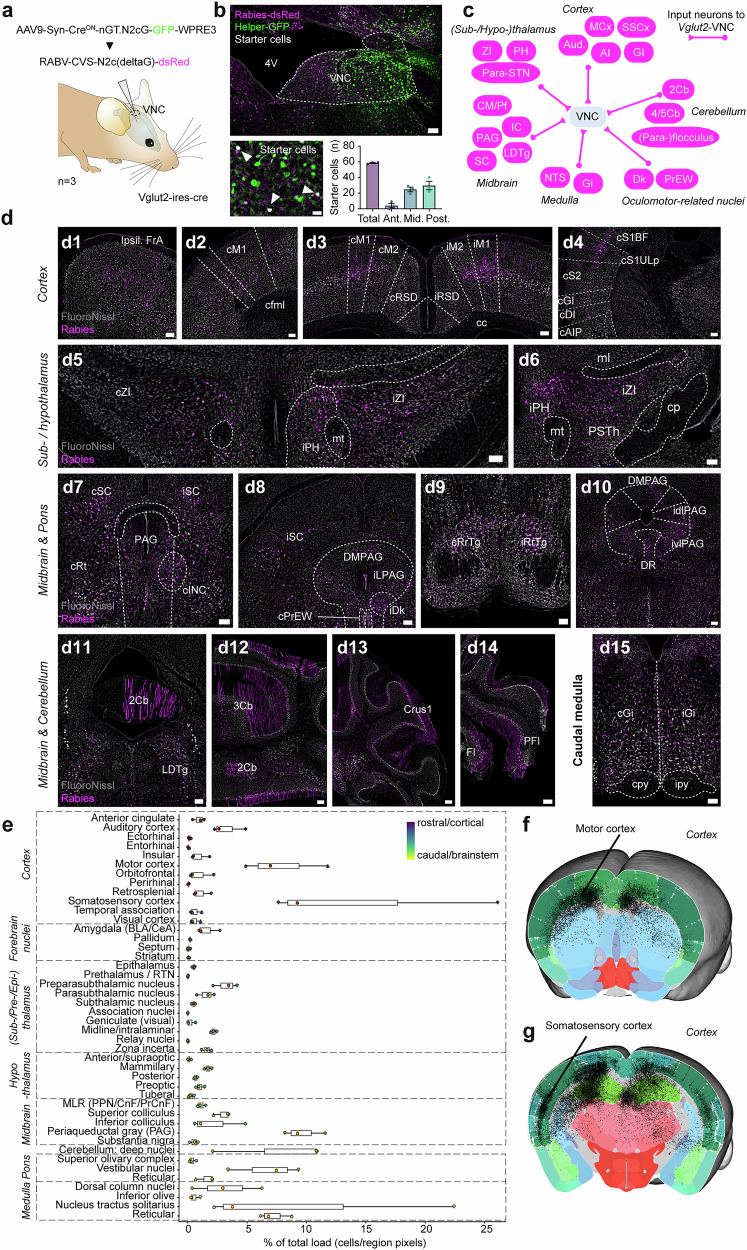
Sensorimotor cortices provide strong afferences to Vglut2-vestibular nucleus complex neurons. **a** Schematic illustrating the tracing strategy for the cohort (*n* = 3 mice). **b** Injection region within the VNC. Starter cells are visible in white (overlap of the two signals, arrowheads on the zoomed-in picture), and their quantification (bar graph shows mean ± s.e.m. and individual data points) showed a homogenous count across animals. Scale bars: 200 µm (top), 20 µm (bottom). AP: −6.24 mm from bregma. **c** Wiring diagram of monosynaptic inputs to Vglut2-VNC neurons identified by rabies tracing. **d** Set of example images of regions providing monosynaptic input to the VNC, including frontal (d1), motor (d2-3), sensorimotor and insular cortices (d3-4). Subthalamic regions, namely (para-)subthalamic nucleus and zona incerta, and paraventricular hypothalamus project to VNC (d5-6). Midbrain periaqueductal gray, superior colliculi and oculomotor-related nuclei provide input to VNC (d7-10). VNC receives projections from cerebellar vermis, (para-)flocculus - vestibulocerebellum (d11-14) as does the caudal medulla reticular formation (d15) and nucleus of the solitary tract. Scale bars: 200 µm. AP levels (from bregma): d1-d15: +2.68, +1.94, −0.70, −1.06, −1.82, −2.30, −3.16, −3.64, −4.24, −4.48, −5.02, −6.72, −5.20 mm. **e** Unbiased quantification results across brain regions show targeting of VNC by the sensorimotor and motor cortex as well (para-)subthalamus (see Supplementary Fig. 5). Exemplary pseudo-3D representations of motor (**f**) and sensorimotor (**g**) cortical afferences to the VNC (data points from *n* = 3 animals shown). Source data are provided as a Source data file. Micrographs: experiments repeated *n* = 3 times with similar results. Orange lines are medians, boxes are interquartile ranges. Whiskers are most extreme data points. Mouse head drawing adapted from^77^.

We next anatomically examined the afferences to glutamatergic VNC neurons, specifically testing whether any originate from VNC output targets, which would suggest potential feed-forward motifs. To achieve this, we sequentially injected a helper and then a pseudo-typed rabies virus (RABV) into the VNC of *Vglut2*-cre mice (*n* = 3, Fig. 2a) to delineate monosynaptic, non-cell type-specific inputs to the *Vglut2*-VNC. Before performing brain-wide quantification by atlas region again, we first quantified starter cell count in the VNC, which showed a homogeneous distribution amongst all animals (Fig. 2b). We then analyzed inputs to the VNC and built an afferentome on that basis (input wiring diagram, Fig. 2c). We found that the VNC receives monosynaptic inputs from sensorimotor and, to a lesser extent, frontal & motor cortices (Fig. 2d1-4). Sparse inputs also arise from the insular cortex, demonstrating a reciprocal connection (Fig. 2d4). Within the subthalamus and thalamus proper, we similarly observed reciprocal connectivity with the zona incerta and (para-)STN (Fig. 2d5-6), but not with the ventral thalamic group. In addition, we identified an input source to the VNC within the (paraventricular) hypothalamus (PH, Fig. 2d6). Moreover, we found a reciprocal connection between the VNC and the PAG (Fig. 2d7,10), alongside expected inputs from oculomotor-related nuclei and the superior colliculi (Fig. 2d7-8). Input from the pontine reticular formation to the VNC was also observed (Fig. 2d9). At the level of the cerebellum, both the vermis (Fig. 2d11-13) and the (para-)flocculus (vestibulocerebellum) provide input to the VNC (Fig. 2d14), as expected from vestibulo-cerebellar anatomy. Finally, the medullary reticular formation (Fig. 2d15) and nucleus of the solitary tract (NTS) also project to the VNC (also see Supplementary Fig. 5 for examples along the rostro-caudal axis of the brain).

Together, these two complementary anatomical tracing results demonstrate the widespread embedding of the VNC specifically within cortical, (sub-)thalamic, midbrain and caudal medulla sensorimotor circuitry. These findings set the anatomical pre-requisite of the VNC for modulatory impact across multiple anatomical levels, particularly via the intralaminar thalamus and sensorimotor cortex.

### Titrated optoactivation of glutamatergic vestibular nucleus complex neurons yields a posture and locomotor symptom continuum in healthy and parkinsonian mice

After investigating the structural embedding of the VNC within sensorimotor pathways, we next asked how the perturbation of *Vglut2*-positive VNC neurons would alter kinematics and behavior, and whether such modulation could have therapeutic effects in parkinsonian mice.

To that end, we first confirmed disease-model effects in a viral vector-mediated, alpha-synucleinopathic parkinsonian mouse model: A53T mice^40^.

Specifically, previous studies injected *AAV1/2-A53T* into the SN of mice to induce dopaminergic cell death and nigrostriatal denervation via overexpression of α-synuclein (α-Syn)^40^, thereby establishing a model for parkinsonian α-synucleinopathy. First, wildtype mice (A53T-Wildtype) were unilaterally injected with *AAV1/2-A53T* into the left SN (15 × 10^12^ genomic particles/ml; 500 nl), and (neuro-)degeneration was analyzed via stereological, immunohistochemical and behavioral analyzes four and eight weeks later (see Supplementary Fig. 6). Control mice (EV-Wildtype) received unilateral (left-sided) injection of an identical load of viral particles containing an empty vector-based construct (EV; 15 × 10^12^ genomic particles/ml; 500 nl).

First, we analyzed the optical density (OD) of tyrosine hydroxylase (TH)-positive fibers in the striatum (representative of nigrostriatal dopamine terminals) in both EV- and A53T-Wildtype mice (A53T-Wildtype: week4, *n* = 9; week8, *n* = 6. EV-Wildtype: week4, *n* = 7; week8, *n* = 8). In both the week4 and week8 groups, ipsilateral OD was reduced by ~50% relative to the respective EV-Wildtype group (week4: 51,68%; week8: 48,00%; Supplementary Fig. 6a-b). To further substantiate dopaminergic ipsilateral neurodegeneration of the nigrostriatal pathway, we stereologically estimated TH-positive cell loss in the SN. In A53T-Wildtype mice, TH cell counts in the SN were reduced at both week4 (58,33%) and week8 (59,00%) by approximately 60% compared with EV-Wildtype mice (Supplementary Fig. 6c). Additionally, we found highly significant positive correlations between stereologically estimated SN TH-positive cell counts and both striatal TH^+^ OD (Supplementary Fig. 6d) and SN Nissl-stained cell count (confirming TH-positive cells as neurons; Supplementary Fig. 6e) in both week4 and week8 groups. Lastly, we examined behavioral signatures in the open field test (OF). A53T-Wildtype mice showed an ipsilateral heading bias (quantified as head rotation degree during locomotion) at both week4 and week8 compared with EV-Wildtype mice (Supplementary Fig. 6f). Finally, we qualitatively confirmed α-synuclein deposition 4 and 8 weeks after vector injection in the ipsilateral SN of A53T-Wildtype mice by immunoreactivity (Supplementary Fig. 6g).

Taken together, these data confirm the development of a cellular and behavioral parkinsonian phenotype induced by virally-mediated α-synucleinopathy in the SN in A53T mice.

After establishing the effects of the parkinsonian A53T mouse model in wildtype mice at both an early (week4) and later (week8) time point, we next wanted to functionally interrogate vestibular circuits governing postural and locomotor control in both parkinsonian A53T and healthy EV mice.

**Fig. 3:**
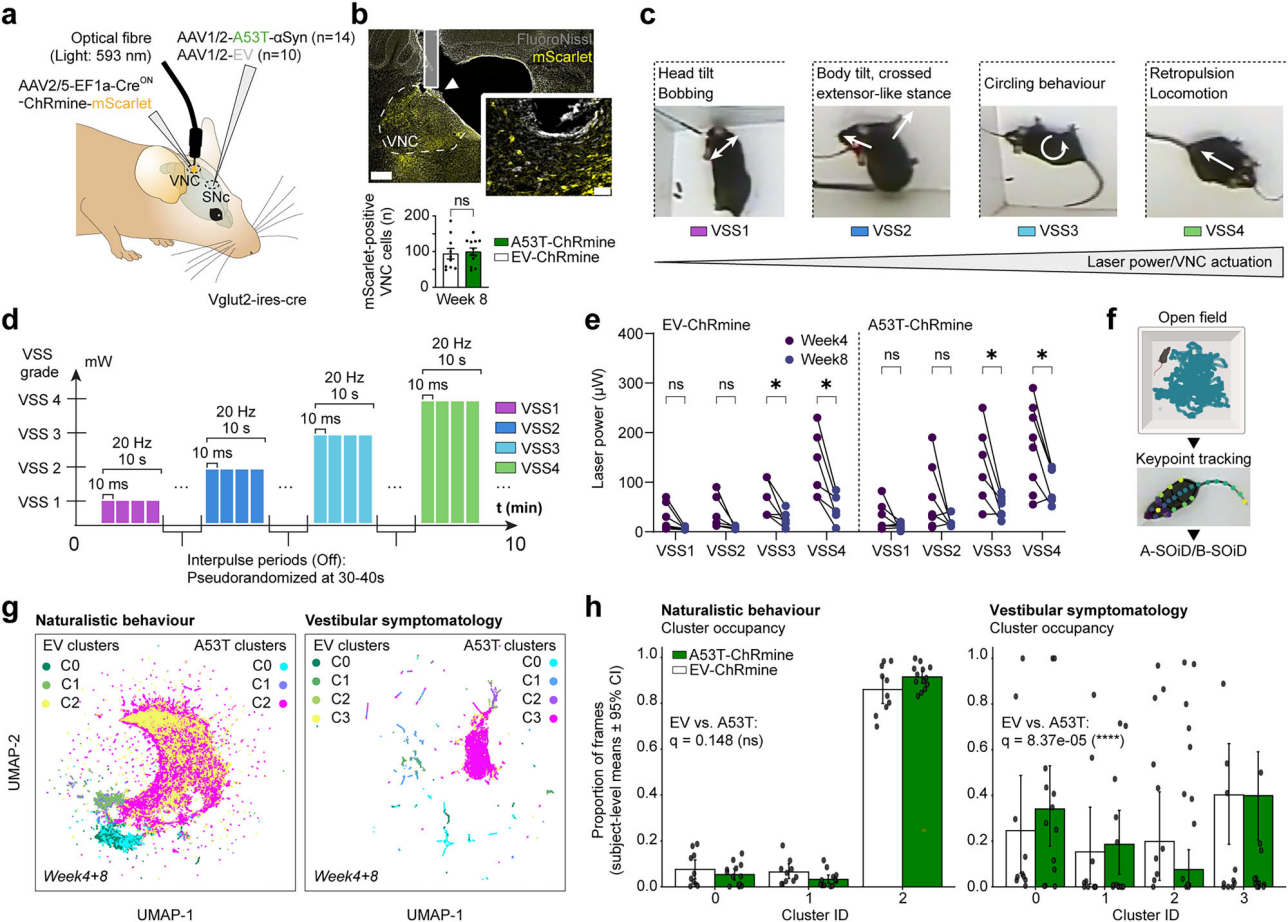
Glutamatergic vestibular nucleus complex neurons regulate posturo-locomotor subtypes in healthy and parkinsonian mice. **a** Optogenetics cohort (*n* = 24), comprising Parkinsonian A53T-ChRmine (*n* = 14) and healthy EV-ChRmine (*n* = 10) mice generated by additive viral vector injection. **b** Representative microscopy image and zoom-in around the fibre tip showing mScarlet-positive cells in the VNC. Quantification showed no difference between A53T and EV mice (two-tailed unpaired *t*-test; A53T: *n* = 12, EV: *n* = 10). Data are mean ± s.e.m. Dotted line, VNC; arrow, optical fibre tip; lines, optical fibre trajectory. Scale bars: 500 µm and 100 µm. AP: −6.00 mm from bregma. **c** Pilot open-field experiments revealed four vestibular symptom dimensions, summarized in a Vestibular Symptom Scale (VSS) capturing phenotype–power relationships (Supplementary Video 1). **d** Based on the VSS, we developed a titration vestibular optomodulation framework in which increasing optical thresholds systematically elicited distinct vestibular symptoms across power levels (Supplementary Videos 2 and 3). **e** Laser power required to elicit each VSS decreased over time in both EV and A53T mice (mixed-effects model: EV, VSS: *p* = 0.0009, TimePoint: *p* = 0.0074, Interaction: *p* = 0.0330; A53T, VSS: *p* = 0.0001, TimePoint: *p* = 0.0172, Interaction: *p* = 0.0163). Two-sided Tukey post hoc tests showed significant reductions for VSS3 and VSS4 only (EV: VSS3: *p* = 0.0322, VSS4: *p* = 0.0106; A53T: VSS3: *p* = 0.0215, VSS4: *p* = 0.0142; VSS1–2, n.s.). Individual datapoints are connected by lines. **f** Pose data from weeks 4 and 8 titration experiments (EV: *n* = 10, A53T: *n* = 14) were classified with A-SOiD^49^ into vestibular symptoms versus naturalistic behavior, then separately clustered and embedded using B-SOiD^50^ to identify behavioral subtypes. **g** UMAP embeddings during systematic titration experiments revealed three naturalistic-behavior and four vestibular-symptom clusters. Colors indicate cluster occupancy per group, showing modest differences between groups. **h** Subject-level mean frame proportions ± 95% CI showed a trend toward group differences in naturalistic clusters and significant group differences in vestibular-symptom clusters (multinomial logistic regression with FDR/Holm-adjusted Wald tests; A53T: *n* = 13, EV: *n* = 10, Statistics: Supplementary Tables 3 and 4). Source data: Source data file. Micrographs: experiments repeated *n* = 3 times with similar results. Boxes are interquartile ranges, whiskers are most extreme data points and lines are medians. Mouse head drawing adapted from^77^.

For vestibular nucleus stimulation, we used optogenetics by introducing the photosensitive actuator *ChRmine*^41,42^ (a red-shifted, cation-conducting channelrhodopsin that excites neurons upon red/orange-light illumination) into the VNC of *Vglut2*-cre mice (Fig. 3a). As previously, a subset of these mice was rendered parkinsonian by unilateral (left-sided) overexpression of A53T-mutated α-synuclein in the SN, while the others were kept as controls and injected unilaterally with EV virus instead. These groups are referred to as A53T-ChRmine and EV-ChRmine hereafter, respectively (both: 15 × 10^12^ genomic particles/ml; 500 nl; Fig. 3a)^40^.

We again qualitatively confirmed α-Syn deposition in the ipsilateral SN of A53T- vs. EV-ChRmine mice by immunoreactivity (Supplementary Fig. 7a–c). Stereological analysis revealed a significant reduction of TH-positive cells in the SN of A53T-ChRmine mice (56,08% loss) compared with EV-ChRmine mice at week8 (A53T: *n* = 12, EV: *n* = 10), indicating dopaminergic cell death (Supplementary Fig. 7d). Again, confirming these cells as neurons, TH-positive cell counts positively correlated with Nissl-stained cell count (Supplementary Fig. 7e). We included behavioral analyzes beyond OF kinematics (cylinder and amphetamine tests) to confirm and extend previous findings^40,43^. As a further surrogate of nigrostriatal dopaminergic dysfunction^44^, we measured total amphetamine-induced rotations (without optogenetic manipulation). This measure strongly discriminated A53T- from EV-ChRmine mice (total rotations, week8 off; AUROC = 0.9375; A53T-ChRmine: *n* = 12; EV-ChRmine: *n* = 10), confirming impaired nigrostriatal dopaminergic output function in diseased mice (Supplementary Fig. 7f). These findings were supported by additional evidence from the cylinder test and top-view OF recordings. In the latter, A53T mice exhibited a similar heading bias, reflecting altered steering of directionality longitudinally (Supplementary Fig. 7g-h, week8 off; A53T-ChRmine: *n* = 12, EV-ChRmine: *n* = 10). In the cylinder test, we confirmed increased forepaw use ipsilateral to the injection site in A53T- versus EV-ChRmine mice (Supplementary Fig. 7i; week8 off; A53T-ChRmine: *n* = 12; EV-ChRmine: *n* = 10), as described previously^40^. Beyond these results, A53T-ChRmine mice also displayed a strong, ipsilesional rotatory bias in the cylinder test over time (Supplementary Fig. 7j), reflected by a skewed rotatory symmetry index compared with EV mice (week8 off; A53T-ChRmine: *n* = 12; EV-ChRmine: *n* = 10). Of note, this static rotatory asymmetry did show a mild, non-significant reduction (5.5%, corr. *p* = 0,4867, two-sided Tukey’s multiple comparisons) with perithreshold VNC actuation. Lastly, the maximum velocity (*V*_max_) on a treadmill correlated significantly with both dopaminergic neurodegeneration (positively, Supplementary Fig. 7k) and rotatory asymmetry in the cylinder test (negatively, Supplementary Fig. 7l; see also Fig. 7 for treadmill-related findings).

After verifying and extending the characterization of model effects in A53T- vs. EV-ChRmine mice, we then estimated and compared VNC transduction levels by quantifying mScarlet-positive cells, encoded by the viral construct, to avoid confounding by group-differential opsin uptake. Transfected VNC neuron counts did not differ significantly between groups (Fig. 3b, week8; A53T-ChRmine: *n* = 12; EV-ChRmine: *n* = 10).

Furthermore, a sham optical control group was injected with a similar virus lacking an opsin (Supplementary Fig. 8a). These mice did not show any vestibular symptoms upon light activation up to 0,5 mW at the fibre tip and showed no motion/kinematic changes with light illumination (Supplementary Video 4, and Supplementary Fig. 8b-c).

By contrast, in optogenetic pilot experiments in the open field (OF), we found that increasing laser power for VNC activation yielded differential posture and locomotor symptoms in both A53T- and EV-ChRmine mice (Fig. 3c, and Supplementary Video 1). Across experiments, we generally used 20 Hz orange-light optical illumination at 10 ms pulse width within pseudo-randomized (by pause intervals) separated 10 s pulses of optostimulation. Canonically “vestibular” symptoms (see below) occurred time-locked upon activation of VNC illumination, and prompted us to develop a categorical *Vestibular Symptom Scale* (*VSS*; [Fig. 3c; see ref. ^32^ for a similar scale) for “clinical” assessment and scaling of the former. Frequently, the first optically-induced aberrant behavior to appear was head tilting, most notably in the yaw and roll plane (*VSS1*). Additionally, some animals showed head bobbing under low stimulation intensity. With increasing laser intensity, ipsilateral body turning, body tilting and crossed extensor-like responses^45^ emerged (both *VSS2*), followed by ipsilateral circling movements (*VSS3*). Finally, at high laser intensities, some animals showed retropulsion or retro-locomotion (*VSS4*; Supplementary Videos 1–3). In many mice, the milder VSS symptoms (*VSS1-2*) were still present to some degree when eliciting stronger VSS symptoms (*VSS3-4*), demonstrating symptom overflow.

To better systematically capture these disruptive stimulation effects, we recorded both A53T- and EV-ChRmine mice in the OF during a Titration Vestibular Optomodulation paradigm (Fig. 3d). First, when looking at the VSS thresholds over time, we found that in both EV- and A53T-ChRmine mice (Fig. 3e), the laser power to elicit each VSS symptom was significantly lower at week4 vs. week8, reaching statistical significance for VSS3 and 4 (the “stronger symptomatology” end of the spectrum) in both groups. Of note, for both A53T-and EV-ChRmine groups, even the strongly disruptive vestibular effects were temporary and reversed after light cessation, as seen by normalisation of disrupted body angles in between stimulation pulses (Supplementary Fig. 9). However, average whole traces of the angle of the head relative to the upper body for body tilting kinematics on a group level indicated that A53T-ChRmine animals seemed to show the more severe postural aberrations earlier than EV-ChRmine mice (Supplementary Fig. 9).

Yet, because our VSS was based on subjective, rater-based observations, we next aimed to check whether a partially supervised machine learning approach based on unbiased pose data would also identify different subtypes of vestibular symptoms when purely looking at pose changes from keypoint tracking. Therefore, we made use of top-view OF-based pose estimation via *DeepLabCut* (DLC, Fig. 3f)^46,47^ from systematic titration experiments (A53T-ChRmine: *n* = 12; EV-ChRmine: *n* = 10). These videos encompassed the whole spectrum of rater-observed, induced vestibular symptoms, but also spontaneous behavior which we categorically annotated (inside *BORIS*^48^) as “*Vestibular symptomatology”* and “*Naturalistic behaviour”*, respectively. We then leveraged a semi-supervised machine learning pipeline (*A-SoiD*^49^) to train an active learning classifier, which performed well at detecting both behavioral categories (average performance: “*Vestibular symptomatology”*: 91%, “*Naturalistic behaviour”*: 98%; see Supplementary Fig. 10 for details on workflow and training data).

Subsequently, we applied this active learning classifier to the full set of our longitudinally acquired titration data, including both week4 and week8 data (A53T-ChRmine: *n* = 12 mice with week4: *n* = 11, week8: *n* = 12 videos; EV-ChRmine: *n* = 10 mice with week4: *n* = 9, week8: *n* = 10). We used clustering and embedding algorithms (*B-SoiD*^49,50^) implemented in A-SoiD to identify distinct subtypes within “*Vestibular symptomatology”* and “*Naturalistic behaviour”* in A53T- vs. EV-ChRmine mice (*n* = 42 videos total). Further, we used statistical and visualization methods to identify distinct behavior within week4 vs. week8. Naturalistic behavior (Fig. 3g, left) from both healthy EV- and parkinsonian A53T-ChRmine fell similarly into three behavioral clusters. One cluster (*cluster 2*) was predominant in that it contained most data points, reflecting the highest proportion of time spent in that behavioral cluster. Albeit not reaching statistical significance, there was a trend towards a shift in the distribution of cluster occupancy in “*Naturalistic behavior”* between EV- and A53T-ChRmine mice (Fig. 3h, left), suggesting very subtle disease state differences. Vestibular symptomatology behavior was classified into 4 separate clusters, again with one being overly represented (*cluster 3*) (Fig. 3g, right). Interestingly here, A53T-ChRmine mice showed significantly different cluster occupancy compared with EV-ChRmine mice, however mostly relating to the smaller, noisier clusters (*cluster 0, 1, 2*) (Fig. 1h, right).

We then secondarily used multinomial logistic regression (see Supplementary Tables 3 and 4) to assess potential longitudinal changes in cluster occupancy between week4 and week8 for both behavioral categories (Supplementary Fig. 11a-b). For *‘Naturalistic behavior’* clusters (Supplementary Fig. 11c), no significant changes were detected. However, we found indications of changes in the distribution of symptoms for both A53T- and EV-ChRmine mice for “*Vestibular symptomatology”* clusters along weeks (Supplementary Fig. 11d).

**Fig. 4:**
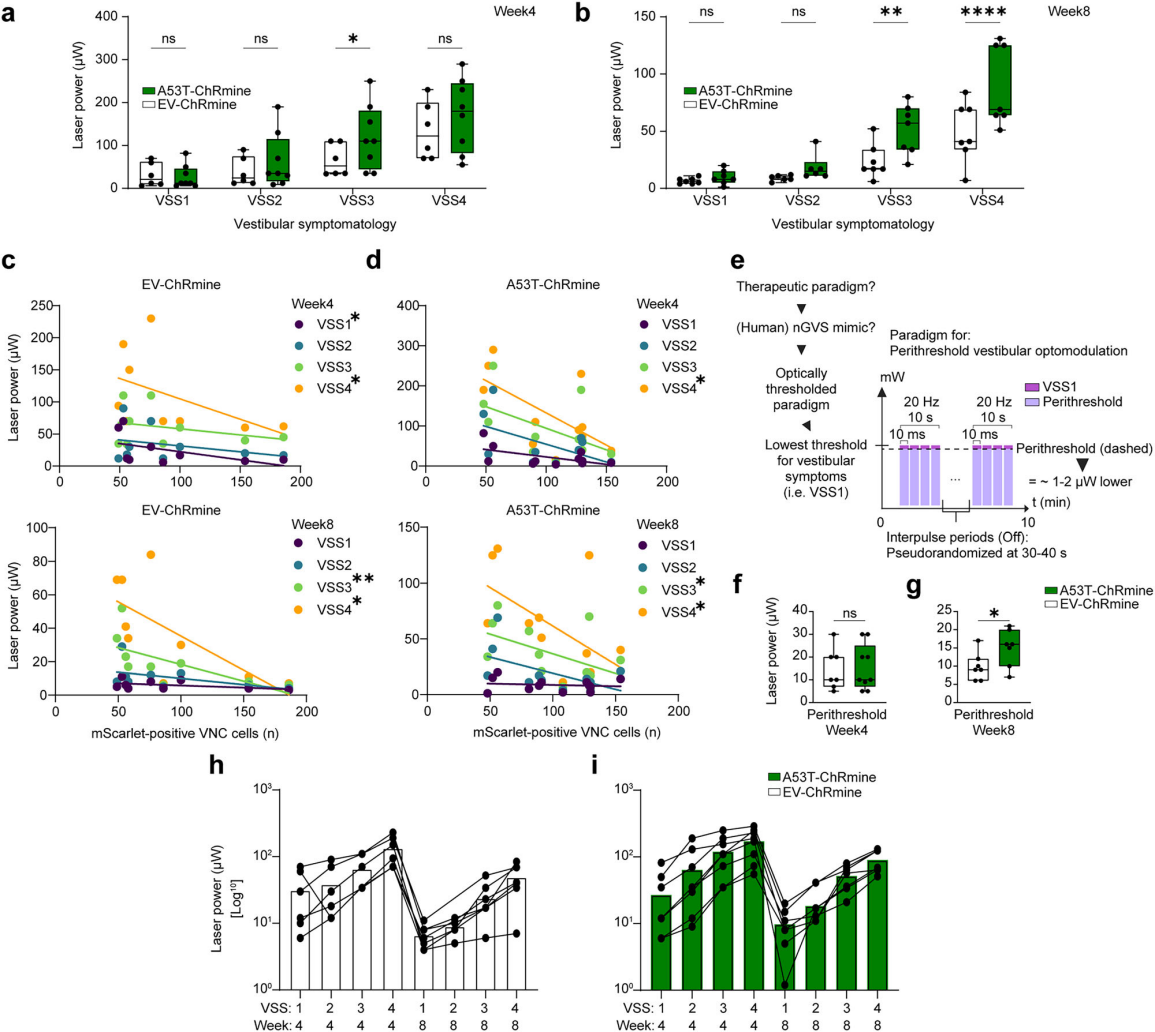
Glutamatergic VNC neurons become less optically responsive in Parkinsonian A53T mice. **a** Laser power (µW) required to elicit the 4 VSS categories at week4. VSS3 power is significantly higher in A53T (*n* = 8) than in EV (*n* = 6) mice (mixed-effects model: VSS: *p* = <0.0001, Group: *p* = 0.2525, Interaction: *p* = 0.0189; two-sided Tukey’s multiple comparisons). **b** Laser power (µW) required to elicit the 4 VSS categories at week8. VSS3 power is significantly higher in A53T (VSS1,3,4: *n* = 7, VSS2: *n* = 6) than in EV (VSS1,3,4: *n* = 7, VSS2: *n* = 6) mice (mixed-effects model: VSS: *p* = <0.0001, Group: *p* = 0.0145, Interaction: *p* = 0.0019; two-sided Tukey’s multiple comparisons). **c** Relationship between laser power and mScarlet-positive VNC cell count in EV mice shows a significant correlation for VSS1, 3 and 4 (all *n* = 9, Spearman’s *r*: week4: VSS1: *r* = -0.66, *p* = 0.0294; VSS4: *r* = -0.68, *p* = 0.0258; week8: VSS3: *r* = -0.82, *p* = 0.0047; VSS4: *r* = -0.76, *p* = 0.0117) **d** Relationship between laser power and mScarlet-positive VNC cell count in A53T mice shows a correlation for VSS3 and 4 (week4: *n* = 11, week8: *n* = 12, Spearman’s *r*; week4: VSS4: *r* = -0.53, *p* = 0.0488; week8: VSS3: *r* = -0.55, p = 0.0325; VSS4: *r* = -0.63, *p* = 0.0164). **e** Schematic of the *Perithreshold vestibular optostimulation* paradigm, developed as an ‘optogenetic mimic’ to human (noisy) galvanic vestibular stimulation (nGVS), by stimulating just below the threshold for vestibular symptoms. The rationale is to target the observed reduced optical responsiveness for therapeutic neuromodulation. **f** Laser power used for perithreshold stimulation at week4 was not significantly different between A53T (*n* = 9) and EV (*n* = 7) mice (one-tailed Welch’s *t*-test). **g** Laser power used at week8 in perithreshold stimulation was significantly higher in A53T (*n* = 7) than in EV (*n* = 7) mice (one-tailed Welch’s *t*-test). VSS thresholds (VSS1-4) at week4 and week8 in EV-ChRmine (min. *n* = 6, **h**) and A53T-ChRmine (min. *n* = 6, **i**) groups per individual animal. All except one animal in EV-ChRmine group show consistent linear optical power increases for each VSS threshold. Bars show means, points are single animals each connected by a line across each VSS. Source data are provided as a Source data file. Boxes are interquartile ranges, whiskers are most extreme data points and lines are medians (**a**, **b**, **f**, **g**).

Lastly, we used another cohort of VNC-ChRmine and sham VNC-mCherry mice to investigate whether the higher laser powers used to elicit more “severe” vestibular phenotypes (e.g., VSS2 or VSS4) lead to greater VNC activation than in perithreshold (see Fig 4) and sham-stimulated mCherry mice (Supplementary Fig. 12). To assess optoactivation-induced activity patterns, we analyzed changes in c-*Fos* expression, an immediate early gene (IEG), as a surrogate for recent neuronal activity^51,52^. We found that both perithreshold and VSS4 stimulation led to significantly higher (and a trend for VSS2) *c-Fos* expression in the VNC of ChRmine-expressing mice compared with sham-stimulated mice (Supplementary Fig. 12d). However, we did not detect differences in c-Fos expression between the investigated VSS symptomatologies (VSS2, VSS4) and perithreshold stimulation, suggesting a mechanism independent of gross VNC cell recruitment.

Taken together, these results demonstrate the dose-dependent, progressively disruptive effects on posture and locomotor control by increasing light intensity for VNC optoactivation, and also reveal behavioral differences, as visible not only via subjective observations but also through unsupervised discovery. In addition, we show that optically-induced vestibular symptoms in general, but not their increasing severity, relate to enhanced *c-Fos* activation in the VNC.

### Parkinsonian A53T mice progressively desensitize to VNC optoactivation

Since we saw that the thresholds for vestibular symptoms changed over time in both healthy EV- and parkinsonian A53T-ChRmine mice, we next asked whether these dynamics differed between groups when examined longitudinally. We therefore compared the laser powers required to elicit each VSS category at both week4 (Fig. 4a) and week8 (Fig. 4b). A53T-ChRmine mice required significantly higher laser powers than EV-ChRmine mice to elicit strong vestibular symptoms (VSS3 and 4), specifically at week8 (Fig. 4b), indicating hypo-responsiveness to vestibular optoactivation in PD mice. Furthermore, we tested whether the laser powers needed to elicit each VSS category correlated with mScarlet-positive VNC cell count, i.e., opsin-expressing neurons. We found that, predominantly at the extreme end of the symptom scale (VSS3 and 4), laser power thresholds negatively correlated with the number of mScarlet-positive cells in the EV- (Fig. 4c) and A53T-ChRmine animals (Fig. 4d) at both week4 and week8. This means that only the extreme, strong vestibular symptomatology seems to depend on the amount of activated VNC neurons.

The strong symptomatic effects of increasing VNC optoactivation on posture and locomotion in titration experiments and the impaired sensitivity to VNC optoactivation in Parkinsonian A53T mice suggested enhancement of VNC drive for putative therapeutic approaches. To address this notion, we designed a second, therapeutic regime for optical neuromodulation: Perithreshold Vestibular Optomodulation (Fig. 4e). With this perithreshold regime, we specifically aimed to mimic the perceptually thresholded electrical vestibular neuromodulation by nGVS^20,23,26,27^. We calibrated our optogenetic activation at the threshold for vestibular symptoms to appear (i.e., VSS1) and stimulated just below this threshold (operationally defined as an online optical power reduction 1–2 µW below individual VSS1 thresholds), aiming to achieve central activation of vestibular networks^25^ akin to GVS^53^. However, repeated stimulation may (on an individual basis) alter thresholds or responsiveness to stimulation, e.g., due to different ongoing behaviors. We thus termed this approach “perithreshold stimulation” rather than “subthreshold stimulation”. Consistent with the titration experiments thresholds, perithreshold optoactivation required significantly higher laser powers at week8 (but not week4) in the A53T-ChRmine vs. EV-ChRmine group (Fig. 4f, g).

Taken together, our data show how optical thresholds to activate the vestibular system change over time and in a Parkinsonian disease state in mice. These findings, together with our characterization of the disruptive effects of suprathreshold vestibular optoactivation, enabled us to develop a therapeutic optogenetic stimulation regime.

### Sub-symptomatic excitation of glutamatergic VNC neurons causes target region engagement in (sub-)thalamus and brainstem

**Fig. 5:**
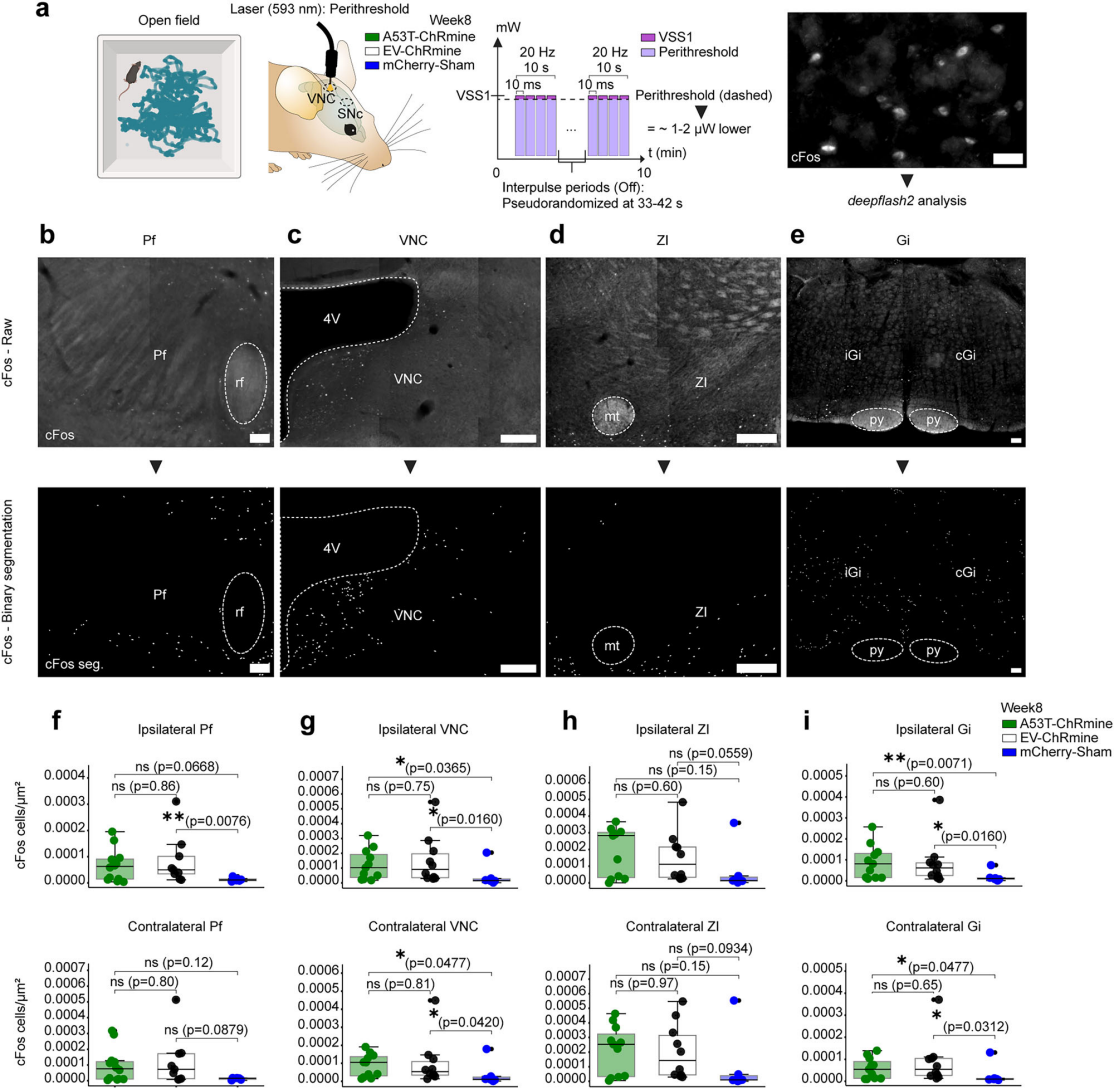
Perithreshold activation of glutamatergic VNC neurons enhances (sub-)thalamic and caudal medulla *c-Fos* expression. **a** Schematic of the experiment with final perithreshold activation of glutamatergic VNC in the open field at week8. *c-Fos*-positive cells were quantified in the major projection targets and the VNC using *deepflash2*^85^ (total *n* = 526 images; A53T-ChRmine: *n* = 216; EV-ChRmine: *n* = 184, sham VNC-mCherry cohort 1; *n* = 127). Example microscopy image from the ipsilateral gigantocellular nucleus (Gi) in one animal showing raw somatic c-Fos signal. Raw *c-Fos* signal (top) around Pf (**b**), VNC (**c**), ZI (**d**), Gi (**e**) and corresponding example segmentations by our model ensemble (bottom). **f** *c-Fos* segmented cells per square micrometer. Ipsilateral Pf shows significantly higher *c-Fos* immunoreactive cell count in EV-ChRmine vs. sham VNC-mCherry mice and a trend in A53T-ChRmine vs. sham VNC-mCherry. Contralateral Pf shows a trend for higher counts in EV-ChRmine vs. sham VNC-mCherry. **g** Significantly higher *c-Fos* immunoreactive cell count in both ipsi- and contralateral VNC in A53T-ChRmine and EV-ChRmine vs. sham VNC-mCherry. **h** Trend for a higher *c-Fos* immunoreactive cell count in both ipsi- and contralateral ZI for EV-ChRmine vs. sham VNC-mCherry. **i** Strong increase of *c-Fos* immunoreactive cell count in A53T-ChRmine, but also EV-ChRmine, vs. sham VNC-mCherry in the ipsilateral >contralateral Gi. Kruskal–Wallis test with two-sided Dunn’s post-hoc tests for each region-hemisphere combination in **f**, **g**. (A53T-ChRmine: *n* = 10, EV-ChRmine: *n* = 9, sham VNC-mCherry cohort 1: *n* = 5 mice). Orange lines are medians; boxes are interquartile ranges; whiskers are most extreme data points. Scale bars: 50 µm (**a**); 200 µm (**b**–**e**). AP levels (from bregma): **b**–**e**: −2.30, −5.80, −1.82, −6.00 mm. Source data are provided as a Source data file. Micrographs: experiments repeated *n* = 3 times with similar results. OF sketch created in BioRender. Ip, A. (2026) https://BioRender.com/7uh3a5l. Mouse head drawing adapted from^77^.

After anatomically identifying VNC targets within (canonical) motor circuitry and demonstrating the disruptive effects of suprathreshold VNC activation, we next tested the functional relevance of VNC perithreshold stimulation in driving activity in selected important target areas in the (sub-)thalamus and brainstem. We again used changes in c-*Fos* expression (here including spatial depth information from *z*-axis), which we quantified using the *deepflash2* pipeline^54^ (a deep learning-based image analysis tool, Fig. 5a) after perithreshold stimulation in the OF context. Anatomically, we focused on subcortical brain regions targeted by the VNC: specifically the widely interconnected ZI (proposed as a global behavioral state regulator^55^), the Pf, the Gi and finally the VNC itself (Fig. 5b–e). We examined A53T-ChRmine, EV-ChRmine and sham mCherry mice lacking a functional opsin for comparisons. Perithreshold vestibular optomodulation in an OF context resulted in increased (mostly ipsilateral) *c-Fos* expression in Pf, Gi, and VNC in both A53T and EV groups compared to mCherry-injected control animals lacking a functional opsin (Fig. 5f–i; A53T: *n* = 12, EV: *n* = 9–10, mCherry: *n* = 5–6). A similar trend was observed for the ZI as well.

Overall, these structural (viral tracing) and functional (*c-Fos*) mapping data show that the VNC links both (striato-)thalamo-subthalamic and motor circuits in the reticular formation of the caudal medulla. These data further provide disease-context evidence that major downstream nodes of the VNC network are functionally recruited in A53T-PD mice under matched optostimulation conditions vs. healthy EV mice. Notably, comparable recruitment is not observed in sham-stimulated (mCherry) controls under the same perithreshold paradigm.

### Thresholded vestibular optoactivation drives pro-locomotor and global behavioural changes

**Fig. 6:**
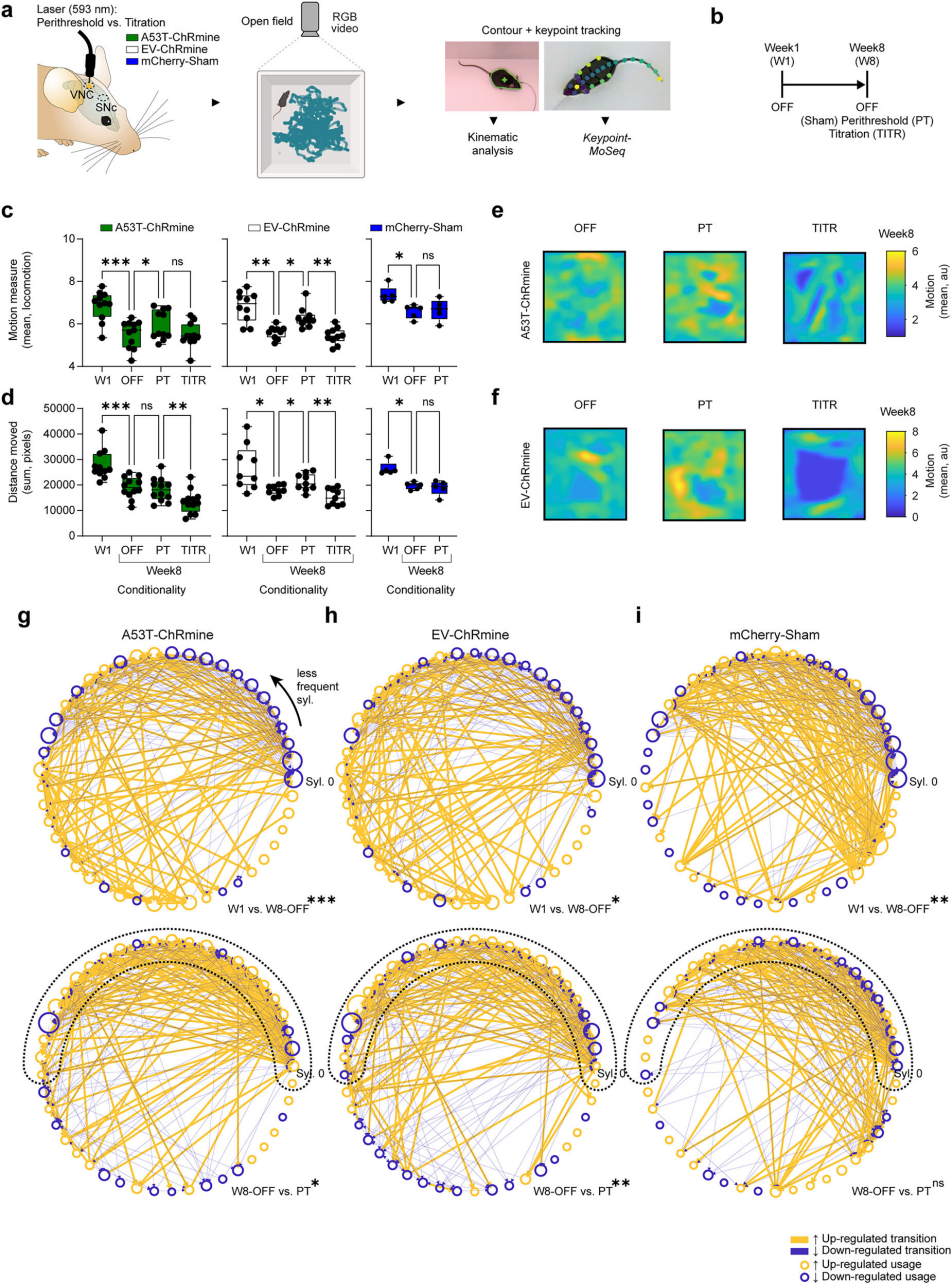
Glutamatergic VNC neurons enhance locomotor performance (animal motion), but not capacity (animal distance moved), in Parkinsonian mice by perithreshold optoactivation. **a** Open-field assessment during vestibular optoexcitation in A53T-ChRmine, EV-ChRmine and sham-mCherry mice. Videos were analyzed using contour-based motion^56^ and Keypoint-MoSeq^58^ syllables (EV-ChRmine: *n* = 10; A53T-ChRmine: *n* = 14; sham-mCherry cohort 2: *n* = 5). **b** Timeline and conditions for weeks 1 and 8. **c** Motion decreased at week 8 off versus week 1. A53T-ChRmine (lognormal RM one-way ANOVA, *F* = 13.08, *p* < 0.0001, *n* = 12) and EV-ChRmine (Friedman statistic = 22.68, *p* < 0.0001, *n* = 10), but not sham-mCherry mice (RM one-way ANOVA, *F* = 11.07, *p* = 0.0213, *n* = 5), showed increased perithreshold motion; absent during titration. **d** Distance moved was lower at week 8 off than week 1 in all groups (sham-mCherry: Friedman statistic = 7.600, *p* = 0.0239, *n* = 5). Only EV-ChRmine mice increased distance during perithreshold stimulation, whereas suprathreshold titration reduced distance in EV-ChRmine (RM one-way ANOVA, *F* = 9.550, *p* = 0.0076, *n* = 9) and A53T-ChRmine (mixed-effects model, *p* < 0.0001, *n* = 12). Week-8 mean-motion heatmaps illustrate perithreshold increases and suprathreshold decreases in A53T-ChRmine (**e**) and EV-ChRmine (**f**). **g**–**i** Circular plots show syllable-transition networks: nodes are syllables, node size reflects usage, and arrow thickness transition probability. Syllables are ordered clockwise (0–44; frequency >0.005; excluded: 40, 45, 47, 48); dotted encircling marks the ~50% most frequent syllables. In A53T-ChRmine mice, habituation and parkinsonian state reorganized global transitions from week 1 to week 8 (**g**, upper), further modified by perithreshold VNC stimulation (**g**, lower). In EV-ChRmine mice, habituation caused milder rearrangement (**h**, upper), while perithreshold stimulation induced rewiring similar to A53T-ChRmine (**h**, lower). In sham-mCherry mice, habituation caused subtle/inconsistent changes (**i**, upper), and perithreshold stimulation did not alter global transitions (**i**, lower). Directional-shift statistics used sequence-level permutation testing. DI used one-sided *p*-values in observed direction and two-sided *p*-values for |DI|; L1 and Frobenius norm statistics used upper-tail *p*-values. Bonferroni correction was applied within test families. Significance is based on L1 *p*-values of each two-sided comparison (“Methods”, Supplementary Table 1). Source data file. Stats (**g**–**i**): Supplementary Table 1. Boxes are interquartile ranges, whiskers extremes and lines medians (**c**, **d**). OF sketch created in BioRender. Ip, A. (2026) https://BioRender.com/7uh3a5l. Mouse head drawing adapted from^77^.

Having confirmed the parkinsonian phenotype in A53T mice and characterized a perithreshold VNC stimulation protocol, we next closely examined motor function and behavior to assess the potential therapeutic effects of this vestibular optomodulation. Using top-view keypoint-based pose estimation together with contour-based tracking, we quantified higher-order behavioral patterns and kinematic changes, respectively, to determine how both these hierarchical levels were altered by perithreshold optoactivation of glutamatergic VNC circuitry. A53T-ChRmine, EV-ChRmine and sham-stimulated mCherry mice (Fig. 6a) were assessed at week1 and at week8 (Fig. 6b).

First, we used contour-based tracking to analyze overall activity in the mice^56^, as opposed to pure locomotion. We found that A53T-ChRmine, EV-ChRmine and sham mCherry mice all showed reduced motion from week1 to week8. However, only A53T-ChRmine and EV-ChRmine mice, but not sham-stimulated mCherry mice, exhibited increased motion (i.e., locomotor performance) with perithreshold stimulation (Fig. 6c). In contrast, suprathreshold stimulation did not produce this pro-locomotor effect in A53T-ChRmine or EV-ChRmine. To additionally quantify locomotor capacity, we retrained the DLC’s “*superanimal_topviewmouse”* network^46,47,57^ for top-view pose estimation on our own recordings (see Fig. 6a and “Methods”). Keypoint-based tracking revealed that distance moved (i.e., locomotor capacity) decreased over time in all three groups, but perithreshold stimulation increased distance moved only in EV-ChRmine mice and not in A53T-ChRmine mice. Importantly, suprathreshold stimulation instead had anti-locomotor effects in our systematic titration experiments (Fig. 6d). Consistent with these results, while overall motion within the OF increased during perithreshold stimulation, it was reduced in titration experiments in both A53T-ChRmine (Fig. 6e) and EV-ChRmine (Fig. 6f) groups.

In order to assess global behavioral state changes associated with parkinsonian disease state and with perithreshold vs. sham stimulation, we then used this large and diverse top-view pose estimation dataset from the top-view OF and applied an unbiased, unsupervised machine learning approach. *Keypoint-MoSeq*^58^ allows for identifying distinct sub-second behavioral modules in pose dynamics, which have been shown to be highly informative for characterizing mouse behavior^59^. We identified 49 principal behavioral “syllables” (i.e., modules or motif**s**) that mapped the naturally occurring behavioral repertoire of mice (syllables 0-44 used with frequency >0.005, see ref. ^58^; Supplementary Video 5 & “Methods”).

To quantitatively assess the behavioral state changes, we first analyzed the transition matrices (Supplementary Fig. 13) of A53T-ChRmine, EV-ChRmine and sham-stimulated mCherry mice, focusing on changes in transition probability across conditions. We found that optoactivation of glutamatergic VNC neurons specifically altered the behavioral transition signature in A53T- and EV-ChRmine groups compared to sham-stimulated mice (Supplementary Fig. 13a–c, bottom). Moreover, even habituation over weeks reshaped the grammar of behavioral transitions in all groups (A53T- and EV-ChRmine, sham mCherry; Supplementary Fig. 13a–c, top).

To investigate this further, we generated network plots to visualize how the overall usage of, and transitions between, distinct behavioral syllables changed with perithreshold VNC stimulation and in the Parkinsonian disease model. In addition to these graphical representations, we performed a multi-level statistical assessment using a global transition-matrix shift permutation approach. This analysis addressed three complementary aspects of how the transition structure changed: (a) the direction index (DI), which measures whether transitions across all syllables are overall preferentially up- or downregulated, (b) the L1 distance, which measures how much the transition probabilities change overall, and (c) a permutation *p*-value, which tests whether the overall mismatch between the two transition matrices is larger than expected by chance.

In detail, we found evidence for a major reorganisation of the transition structure from week1 to week8 in A53T-ChRmine animals, with significant changes in direction bias, overall transition probabilities, and global mismatch between matrices (Supplementary Table 1). EV-ChRmine animals showed a milder rearrangement, driven mainly by changes in overall transition probabilities, whereas sham mCherry animals exhibited only subtle or inconsistent changes from week1 (Fig. 4g–I, top; Supplementary Table 1).

The overall structure of up- and downregulation shows that optoactivation in A53T-ChRmine and EV-ChRmine groups tends to increase the usage of the most frequent syllables. Statistically, perithreshold VNC optoactivation in A53T-ChRmine animals induces measurable but moderate changes in how syllables flow, with no systematic direction bias, but small, yet significant changes in transition probabilities and global mismatch between matrices (Supplementary Table 1). By contrast, EV-ChRmine animals show clear off vs. perithreshold state-dependent transition rewiring, with robust changes in overall transition structure and global mismatch (Fig. 4g–I, bottom; Supplementary Table 1). Most importantly, sham-stimulated mCherry animals do not show any detectable transition rewiring, and transition structure remains essentially identical in off vs. perithreshold state (Supplementary Table 1).

In summary, global motion measure clearly demonstrates pro-locomotor effects with perithreshold stimulation in both healthy and Parkinsonian mice, yet enhancement of locomotor capacity only in healthy animals. Pose estimation at the level of sub-second behavioral syllables further reveals that thresholded actuation of glutamatergic VNC neurons leads to overall reconfiguration of transitions between these behavioral building blocks, with more frequent switching between syllables and a more structured use of the behavioral repertoire during VNC activation. In Parkinsonian A53T mice, exogenous enhancement of VNC excitatory drive thus exerts a differential rescue effect on locomotor performance, but not on locomotor capacity.

### Optoactivation of glutamatergic VN neurons yields disease-specific alleviation of parkinsonian gait dynamics

**Fig. 7:**
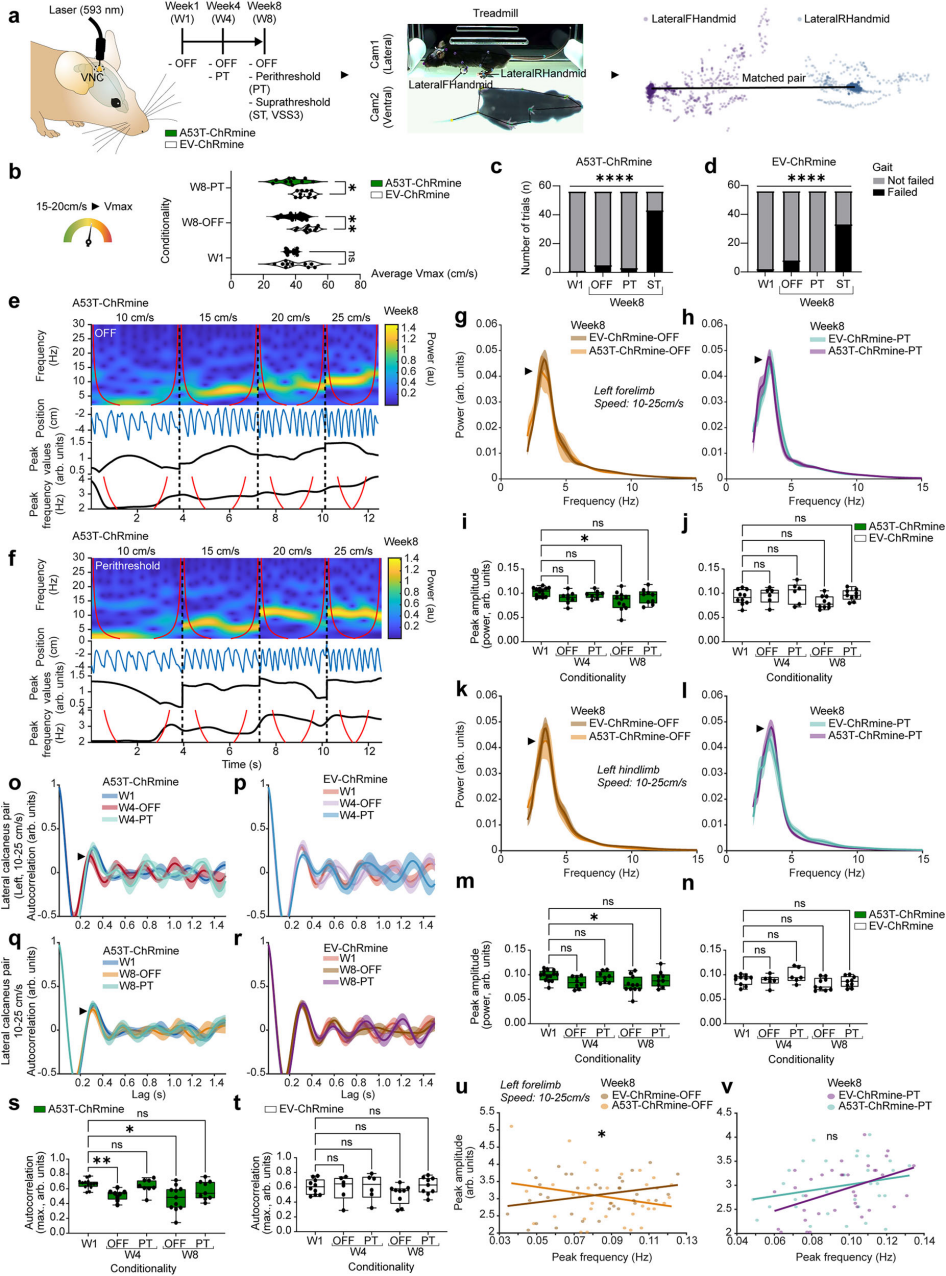
Glutamatergic VNC neurons aid in normalizing gait oscillations in parkinsonian mice. **a** Experimental approach: treadmill optogenetics with top and side cameras, followed by pose estimation^46,47^ to extract gait characteristics (right, example egocentric paw trajectory). Mice were tested at weeks 1, 4 and 8 across four belt speeds (10, 15, 20, 25 cm/s)^12,37^, yielding *n* = 853 videos total (sample sizes in “Methods”). **b** Treadmill V_max_ was higher in EV-ChRmine than A53T-ChRmine mice in off and perithreshold conditions (*n* = 10 per group; two-way RM ANOVA: time/stim, *p* = 0.0294; group, *p* = 0.0030; interaction, *p* = 0.1613; two-sided Šídák tests). Violin plots show full data range. **c**, **d** Using the fluidity of gait index (FGI; FGI1 = failed, FGI2 + 3 = not failed; see “Methods”), suprathreshold stimulation impaired gait versus perithreshold stimulation in A53T-ChRmine (*p* < 0.0001, *n* = 12) and EV-ChRmine (*p* < 0.0001, *n* = 10). Failed/impaired gait showed no rescue by perithreshold versus off stimulation in either group (FGI1 vs. FGI2 + 3; two-sided Fisher’s exact tests). Data show trial numbers across animals per group and condition. Week-8 wavelet scalograms from the left handmid marker of one A53T-ChRmine mouse in off (**e**) and perithreshold (**f**) conditions show increased peak-frequency power with perithreshold stimulation, especially at 20 cm/s. Red lines indicate the wavelet cone of influence. **g**–**n** Periodograms show loss of gait-frequency power and amelioration by perithreshold VNC actuation in A53T- versus EV-ChRmine mice across fore- and hindlimbs and speeds. A53T-ChRmine mice show reversion of peak-amplitude loss to baseline at week 8 with perithreshold stimulation. **o**–**t** Autocorrelation of matched limb markers shows reconstitution to baseline gait synchronicity at weeks 4 and 8 with perithreshold VNC actuation. **g**–**t** were analyzed using mixed-effects models with two-sided Dunnett’s tests. **s**–**v** Peak frequency-amplitude coupling of the left distal limb marker shows loss of correlation across speeds (10–25 cm/s) at week 8 in A53T-ChRmine mice (**u**). Under perithreshold stimulation, this relationship no longer differs between A53T-ChRmine and EV-ChRmine mice (**v**). **g**, **h**, **k**, **l**, **o**–**r** mean ± s.e.m.; **s**–**v** one-sided *F*-tests, lines are intercepts. Source data file. Statistical testing results (**g**–**v**): Supplementary Information. Boxes are interquartile ranges, whiskers extremes and lines medians (**i**, **j**, **m**, **n**, **s**, **t**). Mouse head drawing adapted from ref. ^77^.

Based on our findings that VNC optical actuation produced pattern-specific behavioral improvement, we next asked whether the same intervention would also affect gait, the motor pattern underlying both locomotor performance and capacity. To this end, we first assessed maximum velocity on a treadmill and applied DLC-based analysis of gait kinematics using a dual camera system (Fig. 7a). Whereas EV-ChRmine mice exhibited increased maximal velocity over time (mean: 39.6 vs. 47.7 cm/s, Fig. 7b), A53T-ChRmine mice were unable to make the same adjustments (mean: 37.3 vs. 39.1 cm/s, Fig. 7b). Interestingly, maximal velocity did not change with perithreshold VNC actuation, but it closely correlated with both dopaminergic neurodegeneration and rotatory bias in the cylinder test (see Supplementary Fig. 7k, l).

Second, to cross-validate the specificity of perithreshold (vs. suprathreshold) optoactivation on locomotion, we quantified gait failure with a three-level scoring system, which we termed the fluidity of gait (FGI) index. We based the FGI on the number of consecutive strides (as previously used in disease models^60^) and added gait interruptions as a complementary measure. One condition (the most severe) had to be met: FGI 1, “failure” (consecutive strides ≤4, gait interruptions ≥3); FGI 2, “sufficient” (consecutive strides = 5–7, gait interruptions ≤2); FGI 3, “fluid gait” (consecutive strides ≥8, gait interruptions ≤1). Perithreshold stimulation did not affect gait in either A53T- or EV-ChRmine mice, whereas suprathreshold stimulation (mice stimulated at VSS2-3 thresholds) caused markedly increased gait failure (Fig. 7c,d, and Supplementary Video 6). Hence, while a forced paradigm (max. velocity) seems to de-mask a gait deficit in our PD mice, the FGI could not reliably capture this, yet substantiate the deleterious effects of suprathreshold stimulation.

Interestingly, however, while optical VNC actuation was ineffective in enhancing velocity or rescuing categorially defined gait failure (FGI), initial observations suggested that it resulted in a more regular, “smoothed” gait pattern.

We therefore specifically investigated the oscillatory patterns of individual keypoint markers and as well as matching marker pairs of hindlimbs and forelimbs. In detail, we analyzed the synchronicity and periodicity (autocorrelation analysis) and frequency stability of gait oscillations (powers/peak amplitudes in frequencies) across speeds in both A53T-ChRmine (summary example overview via scalograms, Fig. 7e,f) and EV-ChRmine mice (Supplementary Fig. 14a-b), with and without activation of glutamatergic VNC neurons.

For oscillatory gait pattern analysis, we conducted an in-depth, longitudinal characterization across speeds (10, 15, 20, 25 cm/s) and conditions (week1, week8: without - “off” - or with perithreshold stimulation) on the estimated pose data (*n* = 853 videos; A53T-ChRmine (total *n* = 466)—week1: *n* = 111, week4: *n* = 197, week8: *n* = 158; EV-ChRmine (total *n* = 387)—week1: *n* = 78, week4: *n* = 149, week8: *n* = 160). We found that A53T-ChRmine (Fig. 7g), but not EV-ChRmine mice (Fig. 7h), developed complex gait aberrations, reflected by a reduced power at peak gait frequencies [3–4 Hz range] in ipsilesional/-stimulatory (left) limb markers (Fig. 7k). Vestibular optoactivation enabled us to alleviate these changes towards week1 levels. Specifically, using autocorrelation analysis, we further found that the temporal synchronicity (see Supplementary Fig. 14m–r) of ipsilesional/-stimulatory distal limb markers across speeds decreases in A53T-ChRmine at week4 and week8, when compared to EV-ChRmine mice (Fig. 7m–r). Importantly, we could optically aid in relieving this impairing effect of the parkinsonian model with a perithreshold activation of the glutamatergic VNC at both time points. Furthermore, in the off state, the relationship between peak amplitude and frequency across speeds (10–25 cm/s) was disrupted in A53T mice compared with EV controls, reflected by a loss of correlation between these measures (Fig. 7s). Under perithreshold VNC activation, this difference was no longer evident, indicating the de-correlation in A53T-ChRmine mice could be optically retuned towards normal (i.e., EV-ChRmine) patterns (Fig. 7t). When the analysis was restricted to 20 cm/s, we did not detect any difference between groups (Supplementary Fig. 14t-s), suggesting that sampling the full speed continuum provides a more faithful representation of the peak/amplitude relationship. Whereas right-sided markers and vertical coordinates (*y*-axis) did not show any clear oscillatory changes (Supplementary Fig. 14e–h), we found a trend for an increased autocorrelation in the neck marker in A53T mice, which might indicate axial rigidity, which importantly, saw mild reduction with perithreshold stimulation (Supplementary Fig. 14c, d).

Taken together, these results demonstrate that unilateral nigrostriatal α-synucleinopathy induces ipsilateral changes in limb-associated gait patterns, specifically power in major gait frequencies (peak amplitude analyzes), oscillatory stability of gait (autocorrelation analyzes) and peak amplitude-frequency relationships (correlation analyzes). Thresholded vestibular neuromodulation allows specific alleviation of these deficits via augmentation of excitatory VNC drive.

## Discussion

Our study has two key findings: first, we identified structural and functional contributions of excitatory VNC cells to locomotor behavior in general, and to posture and gait dynamics in particular. Second, mimicking thresholded human vestibular neuromodulation, we optically re-tuned parkinsonian gait and locomotor deficits with activation of VNC glutamatergic neurons in mice, specifically re-stabilizing gait. This might explain the mixed results from clinical trials on vestibular stimulation. In fact, the apparently improved stability during walking in our mice might come from improving vestibular contributions to balance, which has the best evidence (in parkinsonian states) in humans^21,23,27,61^, rather than gait itself^28^.

On an observational level, optical stimulation of VNC glutamatergic neurons yielded a stimulation intensity-dependent, diverse set of posturolocomotor alterations, which blended into each other. This is consistent with phenotypes reported by previous human studies on effects of lesions, or targeted neuromodulation^62–65^ and animal data^32,66–68^ on manipulation of central vestibular pathways subserving head, body and eye movements in yaw, pitch and roll plane^69^.

How are these diverse motor effects mediated by VNC neuronal circuits? Part of the answer to this question can be potentially found in the anatomical connectivity of the VNC. Because anatomical tracing was performed in non-parkinsonian mice, potential disease-related microstructural changes of vestibular projections could not be detected in our data. Notwithstanding, structural mapping of glutamatergic VNC neurons unveiled extensive bilateral projections to key motor hubs, predominantly in the thalamus, particularly the centromedian-parafascicular (CM-Pf) nucleus complex. In addition, our data identify strong projections to subthalamic regions such as the parasubthalamic nucleus and zona incerta, as well as VNC targets in the midbrain and caudal medulla. Reciprocally, VNC input largely originates from sensorimotor cortices, brainstem and subthalamus, and, canonically, cerebellum. On a functional level, optoactivation of VNC excitatory neurons led to enhanced activity of neuronal ensembles in these target regions, confirming the functional relevance of the glutamatergic VNC linked to the quintessential basal ganglia-brainstem axis for movement^9^.

Taken together, these structural and functional circuit findings suggest that VNC excitatory projections intersect with major motor circuitry at different levels of the neuraxis. Our data are in line with previous anatomical studies, which found a vestibulo-thalamo-striatal tract via the parafascicular nucleus in rats^24^ and data from an optogenetic fMRI study in mice^25^, underscoring integration of thalamo-subthalamic with VNC circuitry. The positioning of VNC afferences close to thalamostriatal CM-Pf neurons projecting into the dorsal striatum hints at how the VNC likely conveys its strong influence onto motor systems: by the functionally segregated projections from the intralaminar thalamus to the striatum^70^. The Pf, and its interconnections with the striatum (and STN) specifically, have been implicated in motor rescue in PD mice recently^38,39^. It is conceivable that under parkinsonian conditions in our experiments, the motor improvements are mediated via VNC excitatory inputs to the intralaminar thalamus. Moreover, the Pf is known to mediate ipsiversive head steering, and with prolonged stimulation, full body turns^71^, which corresponds to our observation of head tilting and turning effects caused by incremental VNC optoactivation. Specifically, the encoding of vector components in head velocity and directional steering by Pf^71^ might underlie thresholded VNC optoactivation effects on behavioral dynamics and point towards circuit dysfunction causing directionality bias in parkinsonian mice. Overt postural effects were quickly reversible, suggesting that longer-lasting state effects elicited by optostimulation play a minor role in mediating VSS categories. Nonetheless, we cannot exclude that the emergence of distinct VSS categories could be affected by in- or across-session changes such as systematically increased light intensities, but also experience- and emotional state-dependent states. Notably, we found signs for reduced vestibular responsiveness to optical stimulation in our PD mice. This might, e.g., relate to previously described increased glutamate receptor expression in PD mice, which in turn can be normalized through GVS^72^.

VNC optoactivation improved locomotor performance and harmonized gait patterns in our PD mice. Since we found that the VNC directly and extensively targets the reticular formation in the caudal medulla, it might leverage “hyperdirect” locomotor influence via connectivity with the Gi, sparsely also LPGi^12,37,73^. Further, our data demonstrate that global changes in behavioral dynamics are induced by thresholded VNC activation. The resulting enhanced usage of most frequent behavioral motifs suggests that the VNC also regulates more complex behavioral effects, likely via connections with higher-order circuitry such as the amygdala and frontal cortex.

Yet, we initially hypothesized that the vestibular system might strongly interact with the MLR in mediating effects on posture and locomotion, including under parkinsonian conditions^10,11,16,74^. DBS of the MLR in patients with PD and a posture- and gait-predominant phenotype has been explored, but results remain clinically inconsistent^13,14^. It is important to note that we only found weak glutamatergic connectivity of VNC neurons to the MLR (pedunculopontine nor cuneiform nuclei), especially since electrical vestibular stimulation seems to enhance deficient pedunculopontine nucleus connectivity in PD patients^74^. This may, at least in part, relate to our cell type-specific approach focused on the optical activation of excitatory *Vglut2*-VNC neurons. Given that GABAergic neurons seem to exert negligible global (motor) influence^32^, we postulate that the glutamatergic VNC population subserves most functional influence on posture and locomotion. However, a primarily segregated (likely the second largest) *vGlyT2*-defined glycinergic neuron population^31,33^ remains to be functionally explored, which might display different connectivity and behavioral influence. Furthermore, a distinct glutamatergic population of vestibulo-cerebellar neurons, defined via *vGLUT1*-expression, remains to be characterized in detail^33^.

From a translational bedside-to-bench perspective, we sought to generate mechanistic insights into the central circuit-level effects of non-invasive vestibular stimulation, such as galvanic vestibular stimulation (GVS). The former shows promise in addressing both non-motor and motor symptoms in typical and atypical parkinsonian states, including hard-to-treat axial features^20,21,23,26,27^. We aimed to mimic perceptually-thresholded stimulation with central vestibular network activation (like nGVS) by using optogenetics in healthy and parkinsonian mice. We chose the A53T model for its neuropathological profile associated with α-synucleinopathy and the exploration of an earlier-stage PD level for potential disease modification. Yet, a limitation is that subtle effects of viral/protein expression burden cannot be fully excluded and may have contributed, at least in part, to the observed phenotype. Exploring vestibular effects on severe hypo-/bradykinesia could be addressed in a more severe toxin-based mouse model for PD. Massive impairment of motor behavior (e.g., gait) by suprathreshold optogenetic stimulation is in line with well-known dose-response relationships in human vestibular stimulation^19^. Our individually thresholded optoactivation of the VNC vs. sham stimulation shifted mice into a pro-locomotor state by enhancing behavioral transitions, locomotor performance and locomotor capacity. However, our data demonstrate specificity of these effects in a parkinsonian context. Here, VNC optoactivation allowed for rescue of locomotor performance (movement), but did not alter locomotor capacity (movement distance). However, we found that some gait impairments, such as loss of gait synchronicity vs. healthy mice, could be improved.

While the behavioral effects of thresholded VNC activation were studied in detail, the precise pathway-specific effects remain unidentified. Our tracing studies suggest the mediation of motor effects via vestibulo-(sub)thalamic or vestibulo-medullary projections, warranting further investigation into single-circuit effects combining, e.g., projection-specific in vivo electrophysiology and optogenetic manipulation.

Interestingly, however, non-canonical VNC projections identified, e.g., to the amygdala and PAG, might also bear influence on vegetative function, such as heart rate dynamics associated with vestibular responses. Considering the broad impact of the vestibular system, including at a cortical level^25^, on navigation, affective and autonomic regulation, an integrated examination of both behavioral and autonomic readouts could yield important novel insights into vestibular function, especially from a translational perspective. Specifically, the complex effects of vestibular circuits on integrated behaviors (navigation, defense, etc.) require further investigation, not least because of their connectivity with a proposed global behavioral state regulator, the zona incerta (ZI)^55,75^. The strong influence of the VNC on sensorimotor circuitry is another hint towards the importance of brainstem-basal ganglia interactions for movement^9^. To sum up, through structural and functional mapping, as well as in vivo optogenetics, our data demonstrate a key regulatory role for excitatory vestibular neurons in axial movement, a function that can be exploited to alleviate motor and gait symptoms in Parkinsonian states.

## Methods

### Animals and ethics approval

All experiments were conducted in accordance with local and European animal welfare law, are ARRIVE-compliant and were approved by the local veterinary authorities and animal experimentation ethics committee (AZ 2-631 and 2-2030, Regierung von Unterfranken, Bavaria, Germany). Transgenic *Vglut2-ires-cre* (STOCK Slc17a6^tm2(cre)Lowl^/J, Jackson #016963, The Jackson Laboratory, USA) and C57Bl6/J mice (Charles River Laboratories Germany) were commercially acquired and bred at in-house breeding facilities (Institute of Clinical Neurobiology and Center for Experimental and Molecular Medicine, Wuerzburg, Germany). Experiments were either conducted at the Institute of Clinical Neurobiology or Center for Experimental and Molecular Medicine, Wuerzburg, Germany. A total of *n* = 92 mice were used (*n* = 38 optogenetics, *n* = 11 sham optogenetics, *n* = 13 tracing cross-bred transgenic/wildtype C57Bl6/J mice and *n* = 30 adult male C57Bl/6J). Animals were housed at 14/10-h light-dark cycle at a constant temperature 22 ± 1.5 °C and humidity of min. 50%. Animals had ad libitum access to rodent nutrition and water. No statistical methods were used to predetermine sample sizes. Mice were randomly assigned to experimental/control groups.

### Stereotaxic surgery procedures

A stereotaxic apparatus (Kopf, David Kopf Instruments, USA or Neurostar, Neurostar Germany) was used for injecting viral tracers, optogenetic constructs, and AAVs containing A53T-mutated or EV genomic particles. The surgical procedures generally followed previously described protocols^76,77^. In detail, animals were maintained under isoflurane anesthesia (4% induction, 1–2% maintenance) throughout the procedures, with systemic analgesics (Buprenorphine or Carprofen) administered ~30 min prior subcutaneously. Local anesthetic (Ropivacaine) was injected under the scalp, and a longitudinal incision was made after a 2-min wait. Veterinary eye ointment was applied to keep the eyes moisturized. A stereotaxic drill was used to create a small burr hole above the implantation and/or injection sites.

We used the following stereotaxic coordinates, derived from the stereotaxic mouse brain atlas^78^: VNC: [AP: −6.00; ML: −1.20; DV: −4.20/30], Substantia Nigra Pars Compacta (SNC): [AP: −3.10, ML: −1.40, DV: −4.40, −4.35, −4.30], Dorsolateral Striatum (dlSTR): [AP: 0; ML: +2.20; DV: −3.00, −2.70, −2.50], STN: [AP: −2.00; ML: +1.50; DV: −4.90]. Optical fibers were placed ~200–300 µm above the injection site of the optogenetic viral construct. *AAV1/2-A53T* and *AAV1/2-EV* constructs were serially injected (DV: −4.40, −4.35, −4.30) to target the dorsoventral spread of dopaminergic SNC cells. *retroAAV-mCherry* was serially injected into the dlSTR (DV: −3.00, −2.70, −2.50). The remaining viruses were injected directly into the target area as a single injection. Post-injection, a ~6-min wait time ensured adequate vector diffusion and minimized risk of viral spreading. The exposed skull was sutured for injection-only surgeries or covered with cyanoacrylate for optical fiber implantation to maximize stability. In postoperative care, animals were closely monitored, and analgesics (buprenorphine, carprofen or meloxicam) were administered preemptively and repetitively. Mice received a minimum of 4 days of rest before starting any behavioral experiments and were continuously monitored day-to-day.

### Viral tracing methods

For both anterograde and retrograde viral tracing studies, we utilized commercially available adeno-associated viruses (AAV) or AAV constructs that were gifted from other labs. Viruses were generated in an in-house viral preparation facility from plasmids according to standard protocols for viral cloning, clonal expansion, etc. For anterograde tracing of Vglut2^+^ glutamatergic VNC neurons, we utilized an eGFP-tagged anterograde synaptophysin tracer that co-expresses membrane-bound tdTomato for injection site soma tagging and overall projection fibre estimation (*AAV1-phSyn-FLEX-tdTomato-T2A-SypEGFP-WPRE*, Addgene Plasmid #51509). For the double-conditional tracing approach (*n* = 7), we utilized myc-tagged anterograde synaptophysin viral tracer (*AAV2/5-CAG-Floxed-Syp10xMyc-rev.WPRE*, UNC Vector Core) injected ipsilaterally into the left VNC with a concomitant retrograde tracing from the contralateral dorsolateral striatum (STR, *n* = 4) or subthalamic nucleus (STN, *n* = 3) by an additional retrograde tracer (*retroAAV-hSyn-DIO-mCherry*, Addgene Plasmid #50459), driving the expression of mCherry. Helper virus (*AAV9-Syn-FLEX-nGT.N2cG-GFP-WPRE3*, Charité Vector Core Unit) and pseudotyped rabies virus (*RABV-CVS-N2c(deltaG)-dsRed*, Charité Vector Core Unit) were used for monosynaptic retrograde tracing of VNC neurons with starter cell identity. Rabies helper virus was expressed for 2 weeks, after which pseudotyped rabies virus was injected and expressed for 1 week. All other viral tracers (both antero- and retrograde) were expressed for 4–5 weeks before euthanasia. This was followed by tissue processing, immunohistochemistry and finally quantification where applicable.

### Starter cell count estimation for rabies tracing

We estimated the amount of starter cells double positive for helper virus (GFP) and rabies virus (dsRed) in the VNC manually by quantification inside Fiji/ImageJ^79^. For each individual mouse, we took 11 images of the VNC along the anteroposterior axis, overlaid the atlas outline^78^ and quantified the number of double-positive cells in the VNC. We formed the sum of all cells on every 3 anteroposterior levels of the VNC (anterior, mid, posterior) per animal and then averaged those as well.

### Viral tracing quantification: QUINT pipeline

For both anterograde and retrograde rabies viral tracing studies, we utilized the QUINT pipeline and its associated, freely available toolboxes to enable brain-wide, reference atlas region-based quantification of microscopy image features (e.g., cells, synaptic boutons, etc.). *DeepSlice*^80^ was used for atlas co-registration (serial section number-enforced). *Ilastik*^81^-based pixel and/or object classification workflows were used for synapse and cell segmentation. Integrated atlas-region-based quantification was enabled by *nutil*^82^. We imaged 50% of each mouse brain at an epifluorescent microscope (~80–110 slices at 50 µm serial microtome sections) for QUINT-based quantification. In the anterograde tracing approach (*Syp-eGFP*), we used: animal #1-n=92 slices, animal #2-n=107 slices, animal #3-n=108 slices. In the retrograde rabies tracing approach (*RABV*), we used: animal #1-n=85 slices, animal #2-n=84 slices, animal #3-n=98 slices. Visualisation in a (pseudo)−3-dimensional atlas space was achieved via *MeshView*^83,84^ (https://meshview.apps.ebrains.eu/?atlas=ABA_Mouse_CCFv3_2017_25um). For full details on QUINT workflow, also see: https://quint-workflow.readthedocs.io/en/latest/QUINTintro.html.

For plotting of the results generated within nutil, Allen Mouse Brain Atlas–mapped regions were pooled into predefined anatomical blocks using explicit keyword-based matching of atlas region names. Pooling was performed on the region names provided by the Allen atlas mapping output, with each region assigned exclusively to a single bucket using a first-match rule to prevent double counting. All keyword matching was case-insensitive. Within the cortical block, regions were grouped based on the presence of the following keywords in their atlas names: anterior cingulate (anterior cingulate cortex), motor area (motor cortex), somatosensory (somatosensory cortex), visual (visual cortex), auditory (auditory cortex), retrosplenial (retrosplenial cortex), orbital area or orbitofrontal (orbitofrontal cortex), insular (insular cortex), temporal association (temporal association cortex), perirhinal, ectorhinal, and entorhinal. The forebrain nuclei block included regions containing the keywords caudoputamen or striatum (striatum), globus pallidus or pallidum (pallidum), septal (septal nuclei), and amygdala (amygdalar nuclei). The thalamic block comprised multiple subgroups. Regions were assigned to the epithalamus based on the keywords habenula or pineal. The prethalamus and reticular thalamic nucleus (RTN) group included regions containing prethalamus or thalamus-specific variants of reticular nucleus (e.g., “reticular nucleus of the thalamus” or “thalamic reticular nucleus”), while generic reticular nuclei outside the thalamus were explicitly excluded. Para- and preparasubthalamic nuclei were identified using the keywords parasubthalamic nucleus and preparasubthalamic nucleus, respectively, and the STN was matched using an exact boundary-based keyword. Additional thalamic subdivisions were identified using zona incerta and related fields of Forel (h1–h3), as well as keywords corresponding to relay (ventral posterior, ventral lateral, ventral anterior, lateral posterior), association (mediodorsal, pulvinar, posterior thalamic), geniculate (lateral geniculate, medial geniculate), and midline/intralaminar (reuniens, paraventricular, parafascicular, central medial, centrolateral, paracentral) nuclei. Regions assigned to the hypothalamic block were grouped using the keywords preoptic, anterior hypothalamic or supraoptic (anterior/supraoptic hypothalamus), tuberal, arcuate, ventromedial, or dorsomedial (tuberal hypothalamus), mammillary (mammillary nuclei), and posterior hypothalamic (posterior hypothalamus). The midbrain block included the MLR, defined by the keywords pedunculopontine nucleus (PPN), cuneiform nucleus (CnF), and precuneiform nucleus (PrCnF), including common abbreviations. Superior and inferior colliculi were identified using superior colliculus or inferior colliculus, including laminar subdivisions. The periaqueductal gray was defined using periaqueductal or periaqueductal gray (PAG), and the substantia nigra using substantia nigra, including compact (SNc) and reticular (SNr) subdivisions. The pons comprised regions matched by superior olivary, vestibular nucleus or vestibular nuclei, pontine reticular, and cerebellar deep nuclei identified by fastigial, interposed, dentate, or cerebellar nucleus. Finally, the medullary block included dorsal column nuclei identified by gracile or cuneate, the inferior olive (inferior olive, olivary), the nucleus tractus solitarius (solitary, solitarius), and medullary reticular nuclei defined by the keywords medullary reticular, gigantocellular, paragigantocellular, parvicellular, and lateral reticular. Only regions with non-zero object counts or load values were included in pooled analyzes. For each bucket, regional metrics were summed and expressed either as absolute values or as percentages of the total pooled load across all anatomical blocks.

### c-Fos activity analysis and deep image segmentations with deepflash2

We used an in-house built deep-learning model ensemble with U-net/encoder-decoder architecture for regional quantification of c-Fos immunoreactivity per ROI across A53T-ChRmine, EV-ChRmine and sham mCherry (VNC-mCherry cohort 1) cohorts as described previously^54,85^ with the *deepflash2* pipeline. Three independent raters (J.H., T.P., D.D.) annotated a total of *n* = 27 immunohistochemistry images of c-Fos stainings (*n* = 4 images from rats, *n* = 23 from mice). We used a test/train split of 8/19 (images) and trained an ensemble of 5 models. Ground truth was established and the U-Net convolutional neural network (CNN) model ensemble was compared to the 3 raters' performances. Considering the different Dice scores (dc), we found that the model ensemble, for the most part, performed at least as well as the individual raters in terms of accuracy (dc to estimated ground truth ≈ 0.8; see Supplementary Fig. 15). Via the ensemble, segmentation went as follows: instance segmentations by Cellpose, semantic segmentations via *deepflash2*. We imaged *n* = 3 anteroposterior levels for all ROIs: anterior, middle, posterior for each mouse (A53T: *n* = 12, EV: *n* = 10, mCherry: *n* = 6) at a pseudoconfocal microscope (Axioimager M2, Zeiss). Subsequently, we calculated the feature density in features per micrometer squared using a Python script (Python, Version 3.9) and statistically compared hemispheres at a group level. We analyzed a total of 526 images (A53T: *n* = 216, EV: *n* = 184, mCherry: *n* = 127) from the groups above by generating masks of ROIs using atlas outlines of brain regions^78^.

### c-Fos activity analysis and deep image segmentations with QUINT

For c-Fos immunoreactivity in the VNC and quantification of putatively activated cells under different light conditions, we utilized the QUINT pipeline (see above for details) in a simplified manner. We took *n* = 3 microscopic images from an epifluorescent microscope along the rostrocaudal axis of the VNC at *n* = 3 levels (anterior, middle, posterior). We did so for VNC-ChRmine mice (*n* = 10 total) that were stimulated under the 3 different conditions: perithreshold (*n* = 4 mice), VSS2 symptomatology (*n* = 3 mice) and VSS4 symptomatology (*n* = 3 mice), and the sham-stimulated VNC-mCherry group (VNC-mCherry cohort 2, *n* = 4 mice). We co-registered the full slice images to the atlas via DeepSlice^80^ again and quantified c-Fos-positive cellular signal with an Ilastik^81^-based pixel classification workflow. Afterwards, we finally quantified VNC (all vestibular subnucleus groups from the Allen Mouse Brain Atlas, v2017) *c-Fos* object count inside nutil^82^ in an integrated fashion.

### Generation of the Parkinsonian (A53T-aSyn) mouse model

For the generation of a Parkinsonian phenotype, we utilized the A53T-α-synuclein mouse model of PD^40^. We used a concentrated viral suspension (15 × 10^12^ gp/ml, 500 nl) for both the AAV1/2-A53T (*AAV1/2-CBA/CMV-hA53TaSyn-WPRE-bGH-polyA*) and AAV1/2-EV (*AAV1/2-CBA/CMV-(EV)-WPRE-bGH-polyA*) constructs^86^ (kindly gifted by or acquired from James Koprich and Jonathan M. Brotchie, Toronto Western Hospital, Canada). The stereotaxic procedure and serial dorsoventral injections of the A53T and EV construct for generation of parkinsonian A53T and healthy EV mice, respectively, was conducted as described above. Verification of α-synuclein (aSyn) overexpression and dopaminergic neurodegeneration in the SNC was achieved by immunohistochemistry, which was followed by immunofluorescence microscopy and stereological estimation, respectively.

### Optogenetic experiments

Mice used in optogenetic experiments (i.e., A53T-ChRmine, EV-ChRmine and VNC-ChRmine for VSS-*c-Fos* correlations), as well as the sham-stimulation mCherry groups all had *Vglut2-ires-cre* background. For optical actuation in A53T-ChRmine, EV-ChRmine and VNC-ChRmine groups, we utilized *ChRmine*^41^, a highly sensitive red-light-activated cation-conducting channelrhodopsin (CCR) delivered via a plasmid inside an anterograde AAV construct (*AAV2/5-EF1a-DIO-ChRmine-mScarlet-WPRE*, Addgene Plasmid #130998). For sham actuation, the mCherry groups were injected with an anterograde AAV construct, which delivered a plasmid lacking an optical actuator (*AAV2/5-EF1a-DIO-mCherry*, gifted by Johannes Letzkus Lab, available as Addgene Plasmid #50462). Commercially acquired 4–4.5 mm optical fibres (Doric Lenses, Canada) were placed 0.2–0.3 mm above the target region. A numerical aperture of 0.53 was chosen for all used fibres. Laser power at the fibre tip was gauged before or immediately after an optogenetic experiment with a photometer (ThorLabs Inc., New Jersey, USA). We utilized a laser driver (Doric Lenses, Canada) for generation of laser pulses and the accompanying Doric Neuroscience Studio software (Doric Lenses, Canada) for coding stimulation regimes. For all optogenetic modulation experiments, laser output was controlled using predefined pulse-train stimulation sequences.

For titration experiments, each stimulation condition (VSS1, 2, 3, 4) consisted of three pulse trains (12 trains in total), separated by 4 fixed inter-train pauses (33–42 s pseudorandomized pauses depending on the programmed condition). Within each train, light was delivered at 20 Hz (considering data on tonic vestibular neuron firing rates and intrinsic properties of ChRmine^33,41,42,87,88^) with a 20% duty cycle (10 ms pulses, 50 ms period), yielding 200 pulses per train and a total train duration of 10 s each. Thresholds for each VSS (VSS1, 2, 3, 4) were determined immediately before the full titration experiment per subject and then used in the paradigm settings. For titration experiments, the stimulation conditions were incrementally regulated from low-intensity to high-intensity trains (VSS1 → VSS4; see Supplementary Table 5 for example). In titration experiments, each VSS per animal corresponds to the first occurrence (and respective power) for that symptom category per animal, accounting for mixed or “overflow” symptom patterns, i.e., features of lower VSS levels (e.g., head tilts=VSS1) occurring alongside higher VSS levels (e.g., circling=VSS3). This meant that an animal can show a higher laser power for a higher VSS (e.g., VSS2) than a lower VSS (e.g., VSS1), since these were assigned at the level of symptom, not power. The respective laser powers per group are reported in the results for titration experiments in the OF (see Fig. 4). The conversion of current-based values into optical power at the optical fibre tip corresponding to each animal’s VSS1-4 threshold was determined by a photometer (PM100D, ThorLabs Inc., New Jersey, USA) on a per-session/animal basis.

For experiments with perithreshold stimulation (see Supplementary Table 6 for example) including OF, treadmill and cylinder test, we stimulated just below (i.e., online reduction of VSS1 threshold by 10 mA ~1–2 µW for each subject individually) the momentary (on testing day) individual threshold for vestibular symptoms to appear (i.e., VSS1 on the VSS) and in an interspersed manner during a total recording period of 10 min per mouse. We ran 12 trains of perithreshold stimulation in total, separated by 4 fixed inter-train pauses (33–42 s pseudorandomized pauses depending on the programmed condition). Within each train, light was again delivered at 20 Hz with a 20% duty cycle (10 ms pulses, 50 ms period), yielding 200 pulses per train and a total train duration of 10 s each. The conversion of current-based values into optical power at the optical fibre tip corresponding to each animal’s perithreshold level was determined by a photometer (PM100D, ThorLabs Inc., New Jersey, USA) on a per-session/animal basis.

The respective laser powers per group (EV-ChRmine, A53T-ChRmine) are reported in the results (see Fig. 4). For the VNC-mCherry cohort 2 (sham-stimulated), we used the mean laser power of the healthy EV-ChRmine group from perithreshold stimulation at week8 (mean ± s.e.m.: 9,857 ± 1,438 µW). On the treadmill, we stimulated on demand during each run with the same basic parameters (at 20 Hz with a 20% duty cycle (10 ms pulses, 50 ms period)). For perithreshold stimulation on the treadmill, we used the same principle as described above. For suprathreshold stimulation on the treadmill, in order to assess the specificity of the perithreshold condition and demonstrate its gait-impairing effects, we titrated the individual VSS3 threshold for each subject (i.e., mouse) before the suprathreshold experiment and used these individual thresholds. (A53T-ChRmine: 33,92 ± 7,958 µW, EV-ChRmine: 23,33 ± 4,978 µW; mean ± s.e.m.)

### Transfected mScarlet cell count estimation

We estimated the amount of transfected Vglut2-positive neurons in the VNC manually by quantification inside Fiji/ImageJ^79^. For each individual mouse, we took *n* = 3 images of the VNC along the anteroposterior axis, overlaid the atlas outline^78^ and quantified the number of mScarlet-positive cells in the VNC. We formed an average across all *n* = 3 anteroposterior levels per animal. This average was taken as the transfected cell count estimate and analyzed subsequently.

### Behavioural testing and analysis

#### Generalities and experimental timelines

Mice for behavioral testing from the Parkinsonian A53T-ChRmine (*n* = 14 total) and EV-ChRmine (*n* = 10 total) (with optogenetics) were tested at week1, which was at week1 after surgery, with repeated testing at week4 ([TITR]) and week8 (under different light conditions: off [OFF], with perithreshold stimulation [PT], with titration paradigm [TITR]). A sham-stimulated mCherry group (*n* = 6, VNC-mCherry cohort 1) was tested at week2 (off and with sham-optogenetics) and week3 (with sham-optogenetics, mock perithreshold) after surgery, when viral expression plateaued^41,42^. A concomitant sham-stimulated mCherry group (*n* = 5, VNC-mCherry cohort 2) was tested at week1 after surgery with repeated testing at week8 (under different light conditions: off [OFF], with sham perithreshold stimulation [PT]). Another cohort of optogenetic mice (VNC-ChRmine) was tested at week8 after surgery in a titration paradigm [TITR]. The A53T-Wildtype and EV-Wildtype cohorts were injected and then separated into 2 different groups each. A segregated group of mice from both was tested only at week4, while the others were tested only at week8 after surgery.

#### Cylinder test

The cylinder test was conducted as described previously^89,90^. We recorded mice in a transparent cylinder for 10 min after habituation on video. We then assessed both rotational asymmetry and asymmetry of forepaw use^40^ as indeces by using the following formulae [(1) and (2)]:

1 $${{{\rm{Forepaw}}}}\; {{{\rm{use}}}}\; {{{\rm{asymmetry}}}}\; {{{\rm{index}}}}=\left(\frac{{{{\rm{Ipsilesional}}}}\; {{{\rm{touches}}}}\,\left(n\right)\,+\,\left(0.5x{{{\rm{Bilateral}}}}\; {{{\rm{touches}}}}\left(n\right)\right)}{\left({{{\rm{Ipsilesional}}}}\; {{{\rm{touches}}}}\left(n\right)+{{{\rm{Contralesional}}}}\; {{{\rm{touches}}}}\,\left(n\right)+{{{\rm{Bilateral}}}}\; {{{\rm{touches}}}}\,\left(n\right)\right)}\right)$$

2 $${{{\rm{Rotatory}}}}\,{{{\rm{asymmetry\; index}}}}=\frac{{{{\rm{Counterclockwise}}}}\left({{{\rm{CCW}}}}\right){{{\rm{rotations}}}}\left(n\right)}{\left({{{\rm{CCW}}}}\left(n\right)+{{{\rm{Clockwise}}}}\left({{{\rm{CW}}}}\right){{{\rm{rotations}}}}\,\left(n\right)\right)}$$

#### Amphetamine-induced rotation test

The amphetamine-induced rotation test was conducted as described previously^44^. At week8, we injected mice intraperitoneally with 0,5 mg/kg body weight d-amphetamine and assessed for total rotations in the off vs. perithreshold condition with optogenetic actuation. Animals were pseudorandomized to start in the perithreshold vs. off condition, like in the cylinder test. A total 40 min (20 min-off; 20 min-perithreshold) were recorded. Ipsi-, contralateral and total rotations were counted by a human rater (M.F.). On this basis, statistical analyzes were conducted.

#### Top-view open field (TV-OF)

Open field (OF) trials were conducted in a square, white open field box (39,5  × 39,5 × 39,5 cm). Individual trials lasted 10 min. We recorded videos at 25 frames per second (fps) with a proprietary behavioral analysis system (EthoVision, Noldus Inc., Wageningen, Netherlands or Tucker Davies, Tucker Davis Technologies, USA). For kinematic feature analyzes, we used a custom-trained deep learning model, trained within *DeepLabCut* (DLC)^46^ on top-view videos of OF trials (see section Deep learning-based pose estimation) and motion measures as described previously^56^. Afterwards, coordinate and contour time-series were used for further analysis and feature extraction and analyzed via in-house built MATLAB (MATLAB R2022b, MathWorks, Natick, MA, USA) and Python (version 3.9 or 3.10) scripts.

#### Treadmill gait analysis

To assess gait thoroughly, we used a commercially available treadmill setup by Digigait (Mouse Specific Inc., Massachusetts, USA), which includes two (ventral and lateral) high-resolution cameras (Basler, 165 fps cameras, Basler AG, Ahrensburg, Germany). Animals were extensively habituated (min. 10 min) to the treadmill context before experiments. Both maximum velocity (*V*_max_, see next section) and recorded gait sessions (on video) at different treadmill belt velocities (10, 15, 20, 25 cm/s) were assessed at week1, week4 and week8. First videos were recorded, then *V*_max_ sessions were conducted, to reduce systematic error due to exhaustion. Videos analyzed showed dual views of both (ventral and lateral, see Supplementary Video 6) cameras and were regenerated and resampled in an integrated fashion by the commercially implemented MATLAB script (MathWorks, Natick, MA, USA) from Digigait in 30 fps. These resampled dual-view videos were analyzed (30 fps, see e.g., Supplementary Video 6) within DeepLabCut^46^.

#### Treadmill gait analysis - maximum velocity (V_max_)

To assess gait-associated locomotor performance, maximum velocity (*V*_max_) was used as a quantitative surrogate. Mice were tested to determine their *V*_max_ at each time point, based on the hypothesis that increasing treadmill velocity requires greater physical effort to maintain stable gait. *V*_max_ was tested at week1 and with perithreshold stimulation and in the off condition at week8. Animals were pseudorandomized to either perithreshold→off or off→perithreshold at week8. *V*_max_ analysis involved calculating the average *V*_max_ from *n* = 3 trials for each subject. *V*_max_ was determined by progressively increasing treadmill speed until gait failure (hitting the bumper with the rear). Initial treadmill speed was set between 15 and 20 cm/s to ensure a start in stable gait, then increased progressively. *V*_max_ was assessed regardless of direction of treadmill belt (forwards or reverse).

#### Treadmill gait analysis - fluidity of gait index (FGI)

In order to verify that suprathreshold vs. perithreshold stimulation impairs locomotion significantly, we devised a gait scoring system, based on previous findings of treadmill gait capacity with regard to consecutive strides in a murine disease model^60^. For this reason, we devised a trinomial scoring system, with which we aimed to capture overall gait stability per run: *the fluidity of gait index (FGI)*. We anchored the scoring system in a combined count of (a) consecutive strides^60^ and (b) gait interruptions per treadmill run and animal as such that: one condition (worse counted) had to be met—FGI 1 = failure: consecutive strides ≤4, gait interruptions ≤3 or hitting rear bumper; FGI 2 = sufficient: consecutive strides = 5–7, gait interruptions ≤2; FGI 3 = fluid gait: consecutive strides ≥8, gait interruptions ≤1]. Gait interruptions were defined as an interruption to a continuous gait pattern of ≥1 s and recontinuation of the continuous gait pattern afterwards. If the animal interrupted gait and hit the rear bumper, this was counted as failure (FGI 1). Evidently, any semiquantitative scoring system may be affected by interrater variability; hence, we opted to validate the FGI via assessment of interrater agreement by Cohen’s kappa (k). Both behavioral experimenters (M.F. + J.H.) individually and blindly scored all treadmill runs according to the pre-devised FGI scoring system and interrater agreement was calculated. A Cohen’s kappa (k) of 0.619 (95% CI: 0.540–0.699) and weighted kappa of 0.671 (which is considered a more accurate measure when data are ordinally scaled)^91^ ascertained the validity of the FGI scoring system and showed moderate two-rater inter-agreement in FGI allocation per individual trial.

### Deep learning-based pose estimation

For body part keypoint tracking and pose estimation, we used *DeepLabCut* (DLC, version 2.3.1)^46,47^. Specifically, we trained *n* = 2 different recurrent convolutional neural networks (RCNN) for (a) the TV-OF and (b) the combined lateral-ventral dual-view videos of treadmill gait. For the TV-OF RCNN, we utilized the pre-trained RCNN “*superanimal_topviewmouse”*^57^ and labeled an additional *n* = 255 number of frames taken from *n* = 16 videos (then 95% was used for training). We used a ResNet-152-based neural network with default parameters for 7 training iterations. We validated with 1 shuffle and found the test error was 1.09 pixels, train: 2.04 pixels (image size was ≈ 640 by 624). We used a *p*-cutoff of 0.6. This network was then used to analyze videos from similar experimental settings. Coordinate time-series were further processed in the different custom-made Python and MATLAB pipelines.

For the RCNN used on combined lateral and ventral (i.e., bottom), dual-view videos (30 fps) of gait on the treadmill, we labeled *n* = 1409 frames taken from *n* = 118 videos (then 95% was used for training). We used a ResNet-50-based neural network with default parameters for 3 training iterations. We validated with 1 shuffle and found the test error was 1.66 pixels and train: 4.45 pixels (image size was ≈ 718 by 682). We used a *p*-cutoff of 0.6. This network was then used to analyze videos from similar experimental settings. Coordinate time-series were further processed in the different custom-made MATLAB (MATLAB R2022b, MathWorks, Natick, MA, USA) and Python (version 3.9 or 3.10) pipelines.

### Gait analysis from deep learning

After training the RCNN via DLC, we analyzed an extensive dataset of treadmill videos (total n = 853 videos; A53T-ChRmine (total *n* = 466)—week1: *n* = 111, week4: *n* = 197, week8: *n* = 158; EV-ChRmine (total *n* = 387)—week1: *n* = 78, week4: *n* = 149, week8: *n* = 160) from different speed settings^37^ (10, 15, 20, 25 cm/s) from week1, week4 and week8 testing. We then used the resultant coordinate time series for further analysis via a custom MATLAB script (MATLAB R2022b, MathWorks, Natick, MA, USA). Unfiltered time series of body part coordinates obtained from *DeepLabCut* were imported into MATLAB, where they were calibrated based on pre-recording snippets and aligned between the two cameras. Subsequently, frequency analysis was conducted using continuous wavelet transform via the Wavelet Toolbox 6.2 (MathWorks, Natick, MA, USA) after linearly interpolating missing values or values with a confidence score below 0.95. Data points falling outside the cone of influence, where wavelet analysis has low confidence due to limited data, were excluded from further analysis. Scalograms were visualized as montages, representing different speeds for a given mouse and condition. The magnitude of the wavelet coefficients over time was integrated for each frequency and normalized by the overall signal power, providing a power spectrum. Power spectrum, peak frequency and peak power were extracted per recording and averaged per mouse for speeds, and then across mice. Autocorrelograms were generated from normalized distances between pairs of markers. Specifically, gait coordination was quantified from the periodic recurrence of inter-limb kinematic relationships. For each continuous locomotor bout recorded at a fixed treadmill speed, time series of pairwise distances between matched body parts (e.g., fore- and hind-limb joints) were computed from drift-corrected coordinates (per-frame normalization to snout position along the treadmill axis). Distance time series were *z*-scored within each bout, and unbiased autocorrelograms were computed using MATLAB’s xcorr function. The first non-zero positive peak of the autocorrelogram was used to estimate the dominant lag and peak magnitude. Power spectra and autocorrelograms were compared. Finally, an *F*-test was conducted to determine whether the relationships between peak frequency, peak amplitude, and speed differ significantly across experimental conditions. Linear models with and without interaction terms between these variables and the experimental condition were fit to the data. The *F*-test compared the goodness of fit between these models, with the *p*-value indicating whether the inclusion of interaction terms significantly improved the model’s ability to explain the variance in the data.

### Unsupervised behavioural classification via Keypoint-MoSeq (KPMS)

We trained a full, location-aware KPMS model (a switching linear dynamics system (SLDS) model) as described previously^58^ after fitting an initial autoregressive hidden Markov model (AR-HMM) with a kappa hyperparameter of 10^7^ (dimensionless) and resultant approximate median syllable duration of 7–8 at 25 frames per second (fps) ~175–200 ms. Results of principal component analysis (PCA) on explained variance by the result of the AR-HMM showed that 5 principal components explained ≥90% of variance. The kappa (stickiness) hyperparameter (see Supplementary Table 2 for settings/modeling hyperparameters) was maintained at 10^7^ for fitting the full model (final median syllable duration also at 12 at 25 fps ~175–200 ms). The model was applied on *n* = 82 OF videos from the parkinsonian A53T-ChRmine, EV-ChRmine and sham mCherry mice, each at week1, week8 in off and week8 with (sham) perithreshold stimulation. We found 49 principal behavioral syllables (syllable 0- > X = decreasing frequency) of which syllables with a frequency cut-off of >0.005 (syllable 0–44, excluded syllables: 40, 45, 47, 48) were used for analysis (see full set of 49 syllables in Supplementary Video 5 and for example videos of syllables as grid movies in Supplementary Videos 7–55). Downstream statistical analysis and plotting were conducted as described previously^58,59^ and are specifically described below:

All analyzes were performed in Python using NumPy and pandas for array/tabular operations, h5py for HDF5 I/O, cytoolz for efficient *n*-gram iteration, and Matplotlib/Seaborn plus NetworkX for visualization and graph construction. We parsed 1-D behavioral state sequences (“syllables”) from results.h5 files by scanning common dataset names (syllable, labels, *z*, states) and robustly deduplicating 1-D integer series. Each sequence was then matched to its cohort via a metadata csv file table. For each group, we constructed a bigram transition matrix by first extracting transition points (indices where consecutive labels differ), then counting 2-g over those transition labels. Matrices were normalized by default to bigram (divide by total transitions). To ensure comparability across groups, we defined a fixed node set of syllables by global frequency (computed over all matched sequences) and retained syllables exceeding a minimum frequency (0.5% of frames). We also computed per-syllable usage (state occupancy proportion) for each group on the same node set. Group matrices were shown as heatmaps with globally consistent color limits; between-group difference heatmaps displayed either signed differences (*Δ* probabilities) using diverging maps or absolute differences using sequential maps, with shared vmin/vmax derived across all pairs. We also rendered directed transition graphs per group: nodes represent syllables (size ∝ usage, constant global scaling), edges encode transition probabilities (width ∝ weight, globally scaled; optional thresholding to suppress negligible edges). “Difference graphs” depict edge changes between two conditions (yellow = increase in left vs. right, blue = decrease), and node edge colors analogously encode usage changes. Layouts were either circular (stable ordering by global index) or spring-embedded with a fixed seed for reproducibility. We tested group differences at the sequence level using two complementary non-parametric permutation frameworks: The first is a spectral distance test. For two groups ${G}_{1},{G}_{2}$, we aggregated their normalized bigram matrices ${M}_{1},{M}_{2}$ (built from the same filtered node set) and computed a singular-value spectrum distance $T=\parallel \sigma \left({M}_{1}\right)-\sigma \left({M}_{2}\right){\parallel }_{2}$ (SVD, no complex eigenvalues). The null distribution was obtained by repeatedly permuting sequences between groups (preserving group sizes) and recomputing T (1000 permutations). The *p*-value is the exceedance probability of ${T}_{{{\rm{obs}}}}$ under the null (plus one correction). Global directional-shift test. We quantified net reallocation of transition probabilities using: (i) a Direction Index

3 $${DI}=\frac{\sum \max (D,0)-\sum \max (-D,0)}{\sum {{{\rm{| }}}}D{{{\rm{| }}}}}{{{\rm{for}}}}\,D={M}_{1}-{M}_{2}({{{\rm{range}}}}[-1,1]);$$

(ii) the L1 norm (4) ${{||D||}}_{1}=\sum {|D|}$; and (iii) the Frobenius norm (5) ${{||D||}}_{F}=\sqrt{\sum {D}^{2}}{{{\boldsymbol{.}}}}$ Null distributions for ${DI},{{||D||}}_{1},$ and ${{||D||}}_{F}$ were generated by the same sequence-level permutations (2000). We report an exact one-sided *p*-value for ${DI}$ (in the direction of the observed sign), a two-sided *p*-value for ${{{\rm{| }}}}{DI}{{{\rm{| }}}},$ and upper-tail *p*-values for *L1* and Frobenius norms. Where multiple pairwise comparisons were performed, Bonferroni correction was applied to each *p*-family. Randomization used NumPy’s Generator with a fixed seed (default 42).

### Semisupervised behavioural classification via A-SoiD

To verify the Vestibular Symptom Scale (VSS) we devised, we made use of a semi-supervised machine learning approach within the A-SoiD pipeline, as described previously^49^ by annotation of specific behaviors in an ethogram, leveraging the *BORIS* software^48^ combined with pose estimation data from *DeepLabCut*^46^. Specifically, we extensively trained an active learning classifier on a diverse, yet balanced set of top-view pose estimation data using a total of *n* = 45 videos. Of those, we included *n* = 38 snippet videos (~10 s length, i. e. single optostimulation pulse; random selection, week4 and week8 from *n* = 7 A53T-ChRmine, *n* = 5 EV-ChRmine mice) of VSS symptomatology and *n* = 7 full behavioral videos (random selection, week4 and week8, 10 min length; *n* = 1 perithreshold, *n* = 2 off, *n* = 4 titration videos from *n* = 4 A53T-ChRmine, *n* = 3 EV-ChRmine mice) on identifying both vestibular symptoms and naturalistic behavior reliably. These videos were pose estimated within DLC with the model described previously in the section “Deep learning-based pose estimation”. Second, each video was loaded into BORIS and every single frame annotated in an ethogram with either one of two categories: (1) “Vestibular Symptomatology” and (2) naturalistically occurring behavior (“Naturalistic behaviour”). The ethograms were all exported as singular behavior binary files at 0.04 s (25 Hertz = Hz). The training (hyper-)parameters for the active learning classifier within A-SoiD were set as follows: DLC likelihood cutoff: 0.12, minimum bout duration: 0.05 s, training fraction: 0.1, test fraction: 0.9, max. number of iterations: 1000, confidence threshold for inclusion in training dataset: 0.7, video frame rate: 25 fps, ethogram rate: 0.04 s = 25 Hertz. To independently validate the final A-SOiD classifier (random forest; 729 features), we re-extracted pose-derived features from raw DLC trajectories and re-ran prediction using the frozen iterX model. Across all recordings (*n* = 130,241 temporal bins), the classifier achieved high overall accuracy (97.7%), with a micro-averaged F1 score of 0.977 and a weighted F1 score of 0.973. Performance was solid for the two primary behavioral classes, “Naturalistic Behaviour” (class 0; F1 = 0.987) and “Vestibular Symptomatology” (class 1; F1 = 0.944), indicating reliable discrimination between spontaneous and vestibular-evoked states. Stratified analysis revealed comparable performance across genotypes. In EV-ChRmine animals (*n* = 46,477 bins), classification accuracy reached 98.8% (micro F1 = 0.988; weighted F1 = 0.986), whereas in A53T-ChRmine animals (n = 83,764 bins) accuracy remained high at 97.1% (micro F1 = 0.971; weighted F1 = 0.967). Class-wise precision and recall for both “Naturalistic Behaviour” and “Vestibular Symptomatology” were consistent between groups, indicating that classifier performance generalizes across genotypes without evidence of systematic bias. Together, these results confirm stable behavioral state decoding from pose dynamics using our trained A-SoiD^49^ model (see Supplementary Fig. 10 for details). We then predicted titration data from week4 and week8 fully.

Via a built-in unsupervised clustering/embedding algorithm (B-SoiD)^50^, we aimed to identify subtypes from pose, predicted by our active learning classifier. We clustered and embedded the full set of week4 and 8 titration data (hdbscan, min. percentage for cluster (“Vestibular Symptomatology”: 5%, “Naturalistic behaviour”: 2%, normalization/relaxed embedding options: deactivated) and then used uniform manifold approximation and projection (UMAP) embeddings for visualization and cluster occupancy statistical analyzes to identify subtypes of behaviors and the relevance of longitudinal changes (week4 vs. week8) as follows in detail:

Analyzes were implemented in Python using NumPy and pandas (data handling), joblib (I/O of embeddings and assignments), SciPy (contingency χ²) and statsmodels (multinomial logit and inference). For each behavior, frame-level cluster labels (pred_assign) and 2-D UMAP embeddings were loaded from a serialized object. Filenames provided metadata parsed by regex. Let the categorical outcome be cluster membership $Y\in \{1,\ldots,K\}$. We modeled per-frame probabilities with a multinomial logit (A53T = A53T-ChRmine):

6 $$\Pr (Y=j\;{{{\rm{| }}}}\,X)=	 \frac{\exp \left({\eta }_{j}\right)}{\mathop{\sum }_{{\ell}=1}^{K}\exp \left({\eta }_{{\ell}}\right)},{\eta }_{j}={\beta }_{0j}+{\beta }_{{Aj}}{{{\bf{1}}}}\{{{{\rm{A}}}}53{{{\rm{T}}}}\}+{\beta }_{{Wj}}{{{\bf{1}}}}\{{{{\rm{week}}}}8\} \\ 	+ {\beta }_{{Ij}}{{{\bf{1}}}}\{{{{\rm{A}}}}53{{{\rm{T}}}}\}{{{\bf{1}}}}\{{{{\rm{week}}}}8\},$$

with EV-ChRmine and week4 as reference levels and one cluster as week1 (yielding K−1 logits). To preserve subject-level dependence, we first collapsed frames to subject×group×week×cluster counts, then expanded rows approximately by frequency weights (capped to ≤250,000 expanded rows with proportional downweighting) and fit statsmodels. MNLogit. Inference used a cluster-robust (sandwich) covariance clustered by subject (cov_cluster), guarding against within-subject correlation. We report three orthogonal Wald tests, each pooled over clusters (joint across the K−1 logits): Genotype main effect (overall EV-ChRmine vs. A53T-ChRmine, averaged across weeks). Linear contrast applied per logit

7 $${L}_{{{\rm{gen}}}}:{\beta }_{A\cdot }+\frac{1}{2}{\beta }_{I\cdot }=0$$

and assembled into a block-diagonal L; test statistic (8) $W={(L\hat{\beta })}^{{{{\rm{\top }}}}}{(L\hat{\Sigma }{L}^{{{{\rm{\top }}}}})}^{-1}(L\hat{\beta })\sim {\chi }_{{df}}^{2}$. Time effect in EV-ChRmine (week8 vs. week4). Joint Wald for the Week8 term across logits. Time effect in A53T-ChRmine (week8 vs. week4). Linear contrast

9 $${L}_{{{\rm{time@A}}}53{{\rm{T}}}}:{\beta }_{W\cdot }+{\beta }_{I\cdot }=0.$$

*P*-values are χ² upper-tail probabilities; we adjust across the three planned tests using Benjamini–Hochberg FDR at *q* = 0.10 (Holm is available). We annotated significance by *q*-value thresholds (ns, *, **, ***, ****). For an omnibus composition test (informative) to conduct a descriptive global check, we formed a group×week (GW) × cluster contingency table and computed Pearson’s χ² (scipy.stats.chi2_contingency). To respect subject structure, we also computed a subject-wise permutation *p*-value by shuffling the GW label at the subject level (2000 permutations), recomputing χ² each time, and reporting $\Pr ({\chi }_{{{\rm{perm}}}}^{2}\ge {\chi }_{{{\rm{obs}}}}^{2})$. For effect sizes on compositions, we did the following. To summarize distributional differences of cluster compositions, we report the Jensen–Shannon divergence (bits) and distance between normalized mean subject-level compositions, (10) ${JS}(p,q)=\frac{1}{2}{{{\rm{KL}}}}(p\parallel m)+\frac{1}{2}{{{\rm{KL}}}}(q\parallel m)$ with $\left(11\right)m=(p+q)/2({{{\rm{distance}}}}=\sqrt{{JS}});$ small ε pseudocounts avoid zeros as well as the Aitchison distance (Euclidean distance in CLR space) for compositional robustness.

Comparisons include EV-ChRmine vs. A53T-ChRmine (weeks collapsed) and week8 vs. week4 changes within EV-ChRmine and within A53T-ChRmine. For EV-ChRmine vs. A53T-ChRmine (collapsed over weeks) and for each Group×Week cell, we estimated subject-level mean cluster proportions and 95% CIs via non-parametric bootstrap over subjects (1000 resamples per stratum). CIs are percentile intervals. Cluster IDs were sorted numerically for consistent display; very rare clusters (<1% of frames in both groups) were hidden in figures. For visualization and export we did the following: UMAP scatterplots overlaid clusters with genotype-specific palettes and, separately, distinct colors for Group×Week combinations. Bar plots regarding cluster occupancy show subject-level means ±95% CI for EV-ChRmine vs. A53T-ChRmine and for Group×Week strata. Random operations used NumPy’s Generator with a fixed seed to aid reproducibility.

### Brain processing

At the end of the experiments, animals were euthanized and transcardially perfused at 65 rpm with mechanical pumps. Animals from (sham)optogenetics (including A53T-ChRmine, EV-ChRmine, VNC-ChRmine, sham mCherry 1 and sham mCherry 2 cohorts) were perfused at 90 min (for *c-Fos* expression)^52^ after the last optogenetic experiment in the open field. Anterograde (tdTomato-Syp-eGFP) and double conditional (Syp-Myc) tracing animals were perfused at 4–5 weeks after viral expression. Rabies tracing mice were perfused 1 week after injection of the pseudotyped rabies virus. Perfusion utilized a mixture of 0.01 mM phosphate-buffered saline (PBS) and 0.34% heparin sulfate for 3–5 min. Adequate perfusion was confirmed by observing hepatic blood clearing, adjusting needle placement or pump rate as needed. This was followed by 10–15 min of perfusion with 4% paraformaldehyde (PFA). Brains were then extracted, washed in 0.01 mM PBS, and post-fixed in 4% PFA for 12 h. Subsequently, brains were placed in a sterile-filtered 30% D-Saccharose solution for 48 h until dehydration and then frozen in tissue gel matrix (O.C.T., TissueTek) at −20 °C until cutting and staining. Brains were sectioned into 40 µm slices using a cryotome (Leica PM1950, Leica) and placed in a cryoprotection solution (30% glycerol, 30% PBS, 40% ethanol). Before immunostaining, slices were washed in 0.01 mM PBS and permeabilized with 0.1% Triton-X (ThermoFisher).

### Immunohistochemistry

For dopaminergic cell staining and α-synuclein overexpression verification, primary antibodies against tyrosine hydroxylase (chicken anti-tyrosine hydroxylase, 1:500, Abcam, ab76442) and α-synuclein (rabbit anti-α-synuclein, 1:30000, Sigma-Aldrich, Sigma #S3062) were used. Secondary antibodies were goat anti-chicken Alexa Fluor 488 (Invitrogen #A11039) and goat anti-rabbit Cy3 (Dianova #111-165−144). Slices were counterstained with DAPI (1:500,000, Sigma-Aldrich, Sigma #D8417) or FluoroNissl (1:200, NeuroTrace™ 435/455 Blue Fluorescent, N21479, ThermoFischer) and mounted in embedding medium (AquaPolymount, Polysciences) for microscopy. For stereological estimation, primary antibody against tyrosine hydroxylase (rabbit anti-tyrosine hydroxylase, 1:1000, Abcam, ab112) were used with secondary antibody (goat anti-rabbit, 1:100, Vektor #BA-1000) followed by ABC (Thermo Scientific #32050) and DAB (Vektor #SK 4100). Slices were then mounted and counterstained with cresyl violet solution (1 gram cresyl violet (Merck#1.05235.0025) + 10 ml acetic acid (100%; Sigma#33209-1L)) and subsequently put into an ascending concentration ethanol (70-96-100%) and finally a xylol bath. Slices were mounted and embedded in Vitroclud medium (R. Langenbrinck GmbH, Emmendingen, Germany, Article-No. 04-0001). The mScarlet and mCherry fluorophore tagging the viruses was imaged natively without secondary enhancement. For exemplary images, slices were counterstained with FluoroNissl (1:200, NeuroTrace™ 435/455 Blue Fluorescent, N21479, ThermoFisher). For anterograde viral tracing with *AAV1-phSyn-FLEX-tdTomato-T2A-SypEGFP-WPRE*, primary antibodies against GFP for enhancement (chicken-anti-GFP, 1:1500, Abcam, ab13970) and red fluorescent protein (rabbit-anti-RFP/dsRed/tdTomato, 1:1000, Clontech/Takara, #632496) were used. Secondary antibodies were donkey anti-chicken AF488 (1:1000, Jackson Laboratories, #703-545-155) and donkey anti-rabbit Cy3 (1:800, Jackson Laboratories #711-165-152). Slices were counterstained with FluoroNissl (1:200, NeuroTrace™ 435/455 Blue Fluorescent, N21479, ThermoFisher) and mounted in embedding medium (AquaPolymount, Polysciences) for microscopy. For double-conditional tracings with *AAV2/5-CAG-Floxed-Syp10xMyc-rev.WPRE* combined with *retroAAV-hSyn-DIO-mCherry*, primary antibodies against myc (goat-anti-Myc, 1:500, Abcam, ab9132) and red fluorescent protein (rabbit-anti-RFP, 1:500, Rockland/Biomol, #600-401-379) were used. Secondary antibodies were donkey anti-goat Cy5 (1:800, Jackson Laboratories, #705-175-147) and donkey anti-rabbit Cy3 (1:800, Jackson Laboratories, #711-165-152). Slices were counterstained with DAPI (1:500,000, Sigma-Aldrich, Sigma #D8417) and mounted in embedding medium (AquaPolymount, Polysciences) for microscopy. For the c-Fos^51,52^ staining of A53T-ChRmine, EV-ChRmine and VNC-mCherry cohort 1, we quenched brain slices for quantification of immunoreactive cells via *Deepflash2*^54^ with 100 mM glycine at 7.4 pH and blocked and permeabilized with 0.3% Triton-X, 0.1% Tween 20 at 10% normal goat serum in 0.01 mM PBS. Afterwards, slices were incubated in primary antibody (rabbit anti-c-Fos, 1:1000, Synaptic Systems, #226003) and tagged with secondary antibody (donkey anti-rabbit Cy5, 1:200, Jackson Laboratories, #711175152). Slices were counterstained with DAPI (1:500,000, Sigma-Aldrich, Sigma #D8417) and mounted in embedding medium (AquaPolymount, Polysciences) for microscopy. Due to antibody discontinuation, for the c-Fos^51,52^ staining of VNC-ChRmine and VNC-mCherry cohort 2, we quenched brain slices for quantification of immunoreactive cells via Ilastik^81^ with 100 mM glycine at 7.4 pH and blocked and permeabilized with 0.3% Triton-X, 0.1% Tween 20 at 10% normal goat serum in 0.01 mM PBS. Afterwards, slices were incubated in primary antibody (rabbit anti-c-Fos, 1:1000, Synaptic Systems, #226008) and tagged with secondary antibody (donkey anti-rabbit AF488, 1:200, Jackson Laboratories, #711-545-152). Slices were counterstained with FluoroNissl (1:200, NeuroTrace™ 435/455 Blue Fluorescent, N21479, ThermoFischer) and mounted in embedding medium (AquaPolymount, Polysciences) for microscopy.

### Microscopy and stereology

After immunofluorescence staining, brain slices were imaged at a pseudoconfocal epifluorescent microscope (Axioimager M2, Zeiss or Thunder DMi8 imager, Leica) at different magnifications (10× or 20×). Images for c-Fos analysis via *Deepflash2*^54^ (A53T-ChRmine, EV-ChRmine, VNC-mCherry cohort 1) were taken on a region of interest (ROI) basis for selected brain regions separately for each hemisphere at 20× magnification and z-stacked (1,0 stack size). Each ROI was imaged at 3 anatomically different levels (anterior, middle, posterior) according to the rostrocaudal extent of the respective brain region. Images for c-Fos analysis via *Ilastik*^54^ (VNC-ChRmine, VNC-mCherry cohort 2) were taken on a full slice basis with a different pseudoconfocal epifluorescent imager (Leica Thunder DMi8 imager, Leica) at 10× magnification. From each animal, we took multiple images from 3 anatomically different levels (anterior, middle, posterior. For illustrative images of the double-conditional viral tracing approach, slices were imaged at the same pseudoconfocal microscope (Axioimager M2, Zeiss) at 10× and 20× magnification and *z*-stacked. Exemplary images of injection sites of SN and VNC (Figs. 1–3 and Supplementary Figs. 1–8 and 11) were taken with a different pseudoconfocal epifluorescent imager (Leica Thunder DMi8 imager, Leica) at 10× magnification and z-stacked. Proprietary computational clearing algorithms (“Thunder and Lightning”, Leica) were used for background clearing. We used a maximum projection for these images. Images of anterograde (tdTomato-Syp-eGFP) and rabies (GFP, dsRed) viral tracing were taking at the pseudoconfocal epifluorescent imager (Leica Thunder DMi8 imager, Leica) at 10× without a z-stack at full slice images with an multi object slide holder (4 slots, Pecon) for high-throughput imaging. Proprietary computational clearing algorithms (“Thunder and Lightning”, Leica) were used for background clearing and images exported. Direct immunohistochemistry images were further used for a stereological quantification of dopaminergic SN pars compacta (SNC) cells with an optical fractionator microscope (Olympus BX53, OM Digital Solutions), the Stereo Investigator 64-bit software (MBF Bioscience, Williston, USA) and a Prior ProScan III device (MBF Bioscience, Williston, USA) in an unbiased manner, as described previously^92^. For stereology, serial 40 µm cuts of the SN were used and quantified (High magnification lens (used for quantification mode): 100×, Counting Frame X: 60.00 μm, Counting Frame Y: 60.00 μm, Grid Size X: 100.00 μm, Grid Size Y: 100.00 μm, Section Cut Thickness: 40.00 μm, GuardZone Type: Fixed Distance, GuardZone Height: 2.00 μm, Dissector Height: 16.00 μm). Both Nissl^+^ (grand average neuron estimate) and TH^+^ cells (dopaminergic neurons) were quantified and results exported from the Stereo Investigator software. Brightness and contrast were adjusted for print visibility on all images.

### Statistics and reproducibility

Experiments for data within Figs. 1b, d and 2b, d were repeated three times for the same animal with the same resultant pattern. Samples sizes were chosen based on previously conducted work in the field. Animals were randomly assigned to parkinsonian vs. control (EV), optogenetic or sham groups. The investigators were blinded to allocation during experiments but de-blinded for analyzes.

### Statistical analysis, plotting and data reporting

Statistical testing results are reported in Supplementary Fig. 1, 3, 4 and 7. Statistical analyzes and plotting of analyzes were conducted using GraphPad Prism (Version 10.0). Further, we utilized custom-built MATLAB (R2022b, MathWorks, Natick, MA, USA) and Python (Python, Version 3.7, 3.8, 3.9 or 3.10) scripts and respective packages for statistical analyzes and plotting. Assessment for normality was conducted by using normality/lognormality testing (the most reliable test according to sample size was used) and assessment of Q-Q plots. Individual statistical testing was conducted at a group level via analysis of variance (ANOVA, one- or two-way) or mixed-effects models whenever possible and relevant, to assess factor and interaction effects. In case of non-Gaussian distribution of data, corresponding standard non-parametric equivalents were used. Interrater reliability of the FGI score was assessed by using the Cohen’s kappa online calculator by GraphPad, found on the GraphPad website (https://www.graphpad.com/quickcalcs/kappa1.cfm). Data plotting is described individually for each plot in the figure legends. Significance is annotated as ^ns^*p* ≥ 0.05, ^*^p < 0.05, ^**^p < 0.01, ^***^p < 0.001, ^****^p < 0.0001, if not stated otherwise. Figures were created with Adobe Illustrator and BioRender (Supplementary Fig. 1). The mouse head drawing was initially created for the lab of Andreas Lüthi^77^ and adapted for this publication (Figs. 1–3 and 5–7; Supplementary Figs. 4, 8 and 12). Third-party consent is available for both.

## Supplementary information

Supplementary Information

Transparent Peer Review file

Description of Additional Supplementary Videos

Supplementary Video 1

Supplementary Video 2

Supplementary Video 3

Supplementary Video 4

Supplementary Video 5

Supplementary Video 6

Supplementary Videos 7

Supplementary Videos 8

Supplementary Videos 9

Supplementary Videos 10

Supplementary Videos 11

Supplementary Videos 12

Supplementary Videos 13

Supplementary Videos 14

Supplementary Videos 15

Supplementary Videos 16

Supplementary Videos 17

Supplementary Videos 18

Supplementary Videos 19

Supplementary Videos 20

Supplementary Videos 21

Supplementary Videos 22

Supplementary Videos 23

Supplementary Videos 24

Supplementary Videos 25

Supplementary Videos 26

Supplementary Videos 27

Supplementary Videos 28

Supplementary Videos 29

Supplementary Videos 30

Supplementary Videos 31

Supplementary Videos 32

Supplementary Videos 33

Supplementary Videos 34

Supplementary Videos 35

Supplementary Videos 36

Supplementary Videos 37

Supplementary Videos 38

Supplementary Videos 39

Supplementary Videos 40

Supplementary Videos 41

Supplementary Videos 42

Supplementary Videos 43

Supplementary Videos 44

Supplementary Videos 45

Supplementary Videos 46

Supplementary Videos 47

Supplementary Videos 48

Supplementary Videos 49

Supplementary Videos 50

Supplementary Videos 51

Supplementary Videos 52

Supplementary Videos 53

Supplementary Videos 54

Supplementary Videos 55

## Source data

Source Data

## Data Availability

The data generated in this study are provided in the Supplementary Information/Source Data file. The KPMS model is publicly available in the zenodo database under (10.5281/zenodo.20810587). Source data are provided with this paper.

## Code availability

Custom codes are available from the corresponding author(s) upon request.
