# Supplementary material for: Vestibular nucleus stimulation for ameliorating locomotor dynamics in a Parkinsonian mouse model
Source: Nat Commun. 2026 Aug 4;17:7772. doi: 10.1038/s41467-026-76183-2 (PMC13439372; doi:10.1038/s41467-026-76183-2)
Supplement: Supplementary file 1 [file 41467_2026_76183_MOESM1_ESM.pdf]

Supplementary Figures

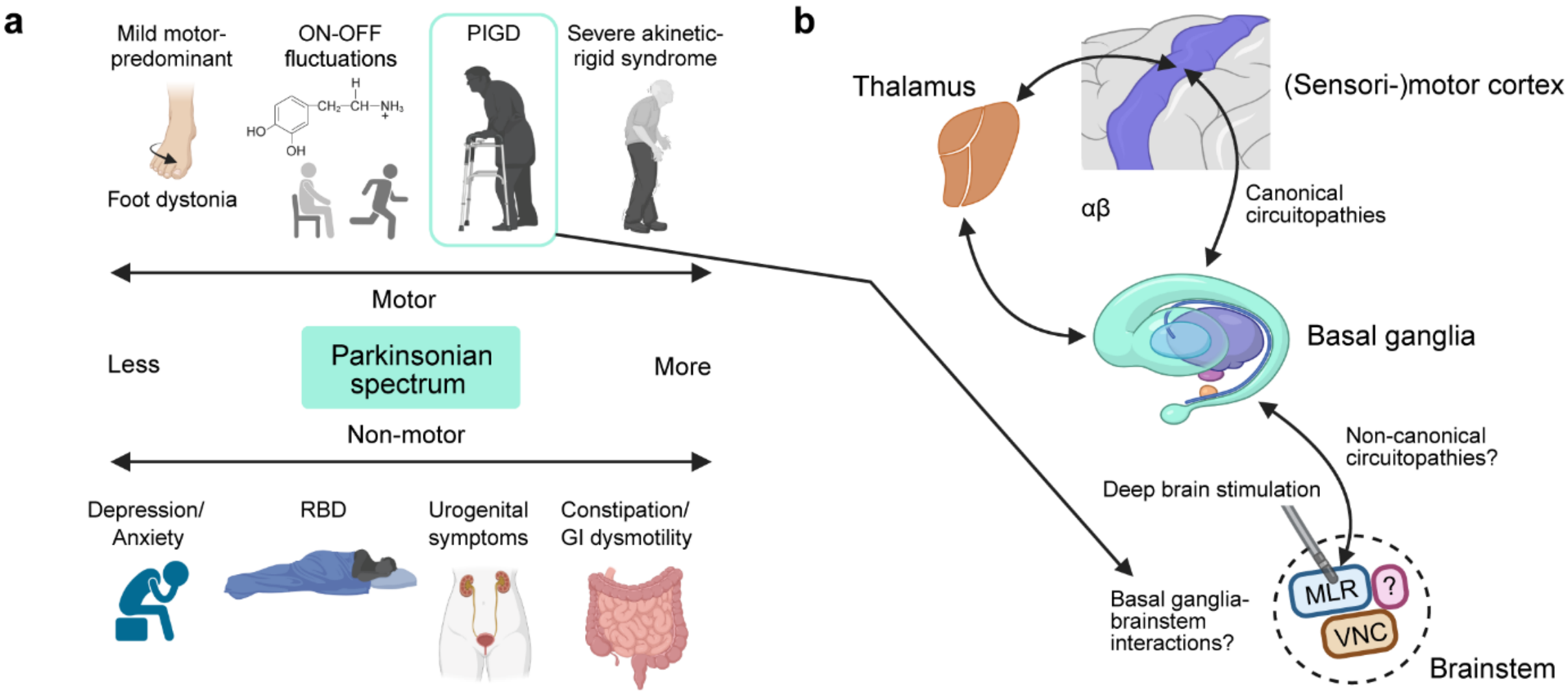

Supplementary Figure 1. Canonical vs. non-canonical sensorimotor circuitopathy in Parkinson's disease: a role for the vestibular system in gait and balance disorders? a. Parkinson's disease (PD) is increasingly recognized as a spectrum disorder with an individually and temporally dynamic set of non-motor and motor features. Isolatable subtypes comprise common phenotypes. Postural instability and gait difficulty (PIGD), an umbrella term, defines phenotypes with predominant axial involvement and is notoriously difficult to treat by neuromodulation and dopaminergic medication. b. Canonical models of basal ganglia pathophysiology in PD highlight oscillatory α- and β-band activity due to loss of dopaminergic input to the striatum. Difficult to treat axial phenotypes have been postulated to be partially reducible to non-canonical sensorimotor circuit dysfunction, including at the level of brainstem motor nuclei. Deep brain stimulation of the mesencephalic locomotor region (MLR), the pedunculopontine nucleus (PPN) and cuneiform nucleus (CnF), is assumed to act at that level. However, results show inconsistency. The role of the vestibular system as a primary integrator of balance, postural reflexes and gait in PD has hitherto remained nebulous. Sketches created in BioRender. Ip, A. (2026) https://BioRender.com/hp0597w.

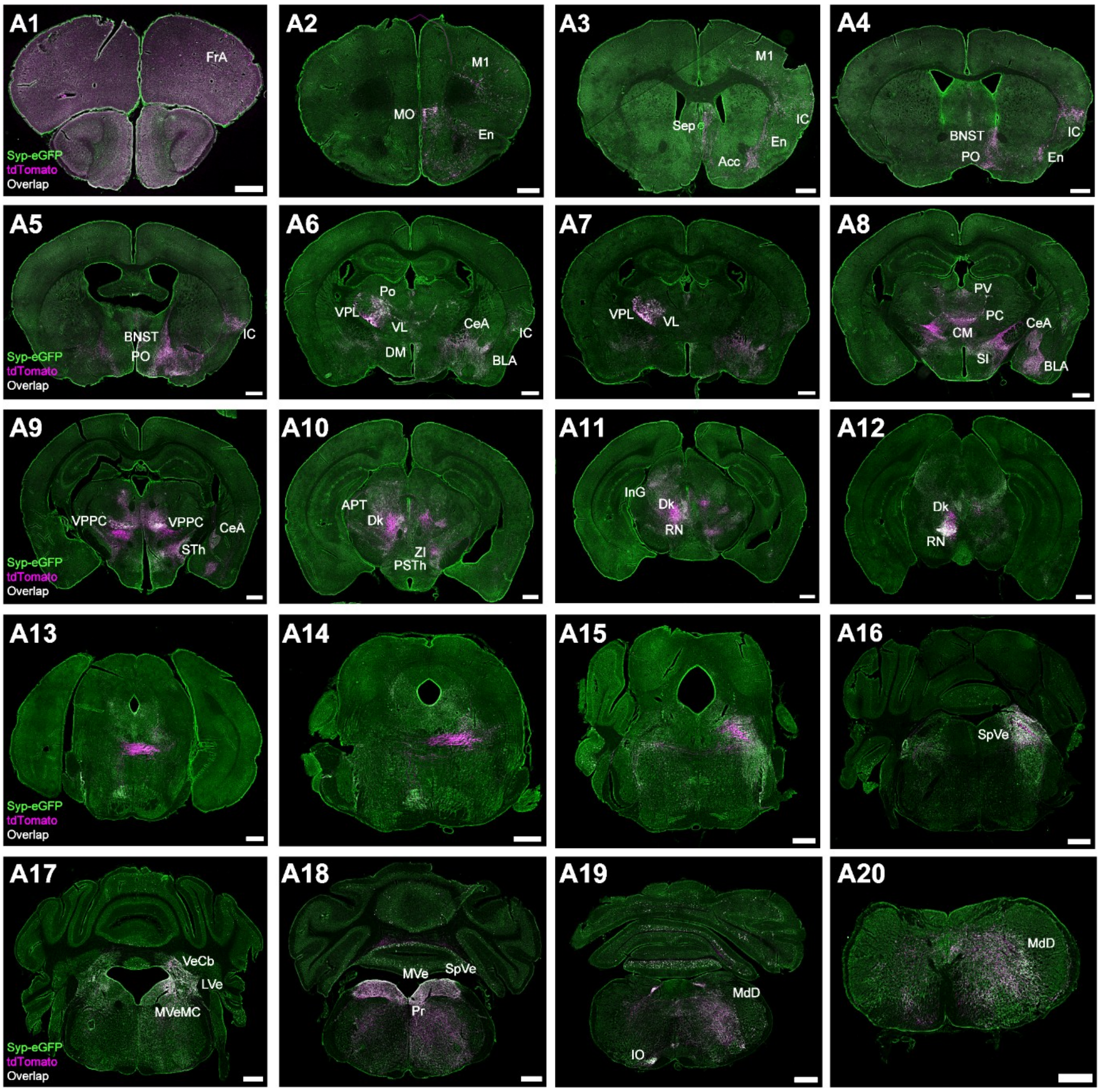

Supplementary Figure 2. Brain-wide projections of Vglut2-vestibular nucleus complex neurons. a. Full slice images of major coronal sections of the mouse brain along the rostro-caudal axis (panel numbers increase from rostral to caudal) illustrating anterograde tracings (from *n* = 3 mice). Synaptic terminal boutons are visible in white (overlap), whereas en passant only fibers appear in magenta. Monosynaptic projections from the glutamatergic VNC predominantly target (sub)-thalamic, midbrain and caudal medulla areas (A5 - A11). Previously unreported projections were identified to the prefrontal (A2) and insular cortex (A3 - 6), amygdala (A6 - 8), and basal forebrain (A5). Canonically expected projections to oculomotor- and cephalic-related nuclei, such as the pre-Edinger Westphal (PrEW), nucleus of Darkschewitsch (Dk, A10 - A11) and red nucleus (RN, A11 - A12) were also present. Left side of each image corresponds to the right hemisphere. Scale bars: all 1000 µm. Brain region abbreviations are taken from Allen Brain Atlas, v2017. AP levels (from bregma): A1 - A20: +2.58, +1.98, +1.54, +0.50, -0.22, -1.06, -1.22, -1.46, -2.18, -2.46, -3.16, -3.40, -4.04, -4.48, -4.84, -5.40, -5.88, -6.84, -7.64, -7.92 mm. Micrographs: experiments repeated *n* = 3 times with similar results.

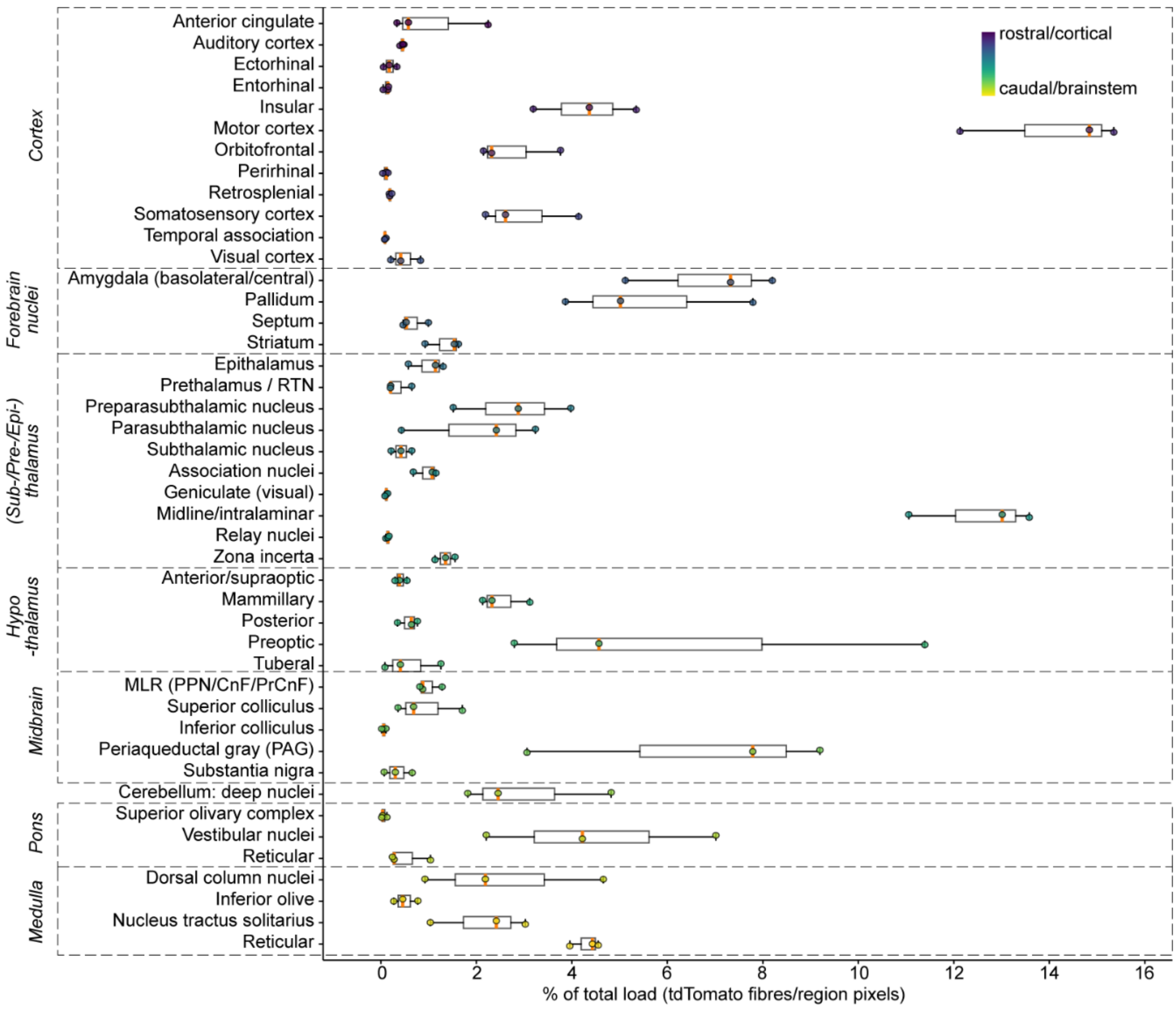

Supplementary Figure 3. Synaptic projections from VNC mainly overlap with membrane-bound tdTomato signal. Unbiased quantification across brain regions of membrane-bound tdTomato (see magenta signal in Supplementary Figure 2) reveals matching results with Syp-eGFP quantification of synaptic boutons. Yet, percentage of intralaminar thalamic group vs. caudal medulla reticular formation is larger (data from n = 3 animals shown). Orange lines are medians, boxes are interquartile ranges. Whiskers are most extreme data points.

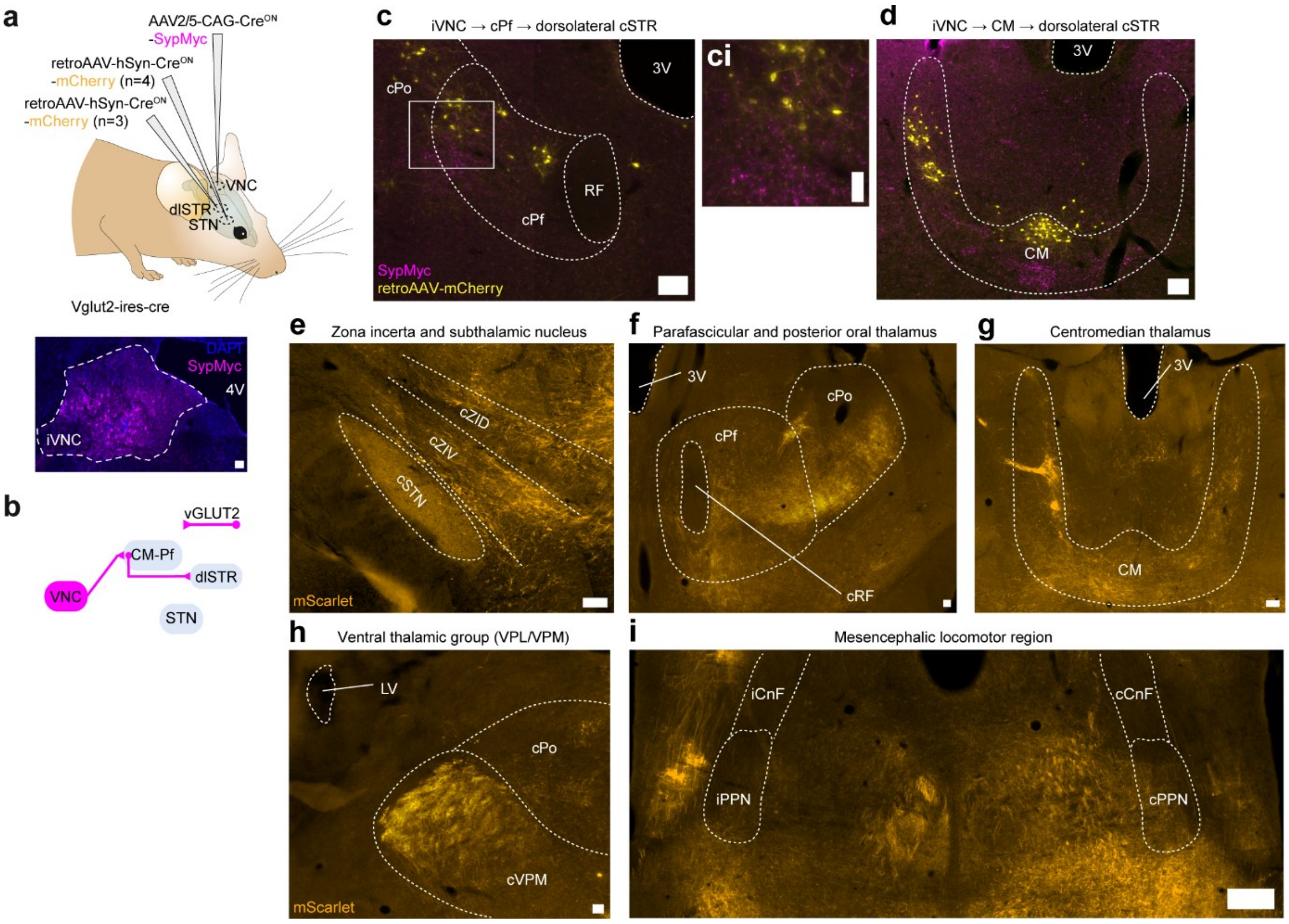

Supplementary Figure 4. Centromedian-parafascicular thalamus links vestibular nucleus complex to dorsolateral striatum. a. Sketch denotes double-conditional tracing cohort with additive injection paradigms (retroAVV into contralateral subthalamic nucleus (STN): *n* = 3 or contralateral dorsolateral striatum (dlSTR): *n* = 4). b. Wiring diagram of glutamatergic VNC's link via intralaminar thalamic group to dorsal striatum; for STN-injected animals, we found no consistent overlaps. c. Synaptic punctae in parafascicular (Pf) (and sparsely also posterior oral (Po)) nucleus of thalamus. VNC synapses and thalamo-striatal neurons overlap (*n* = 4) in Pf (ci). d. Synaptic punctae and thalamo-striatal neurons overlap (*n* = 4) in the centromedian (CM) nucleus. e. mScarlet fibres in ZI, but not STN of A53T/EV animals. f. mScarlet fibres in Pf. g. mScarlet signal in CM. h. mScarlet projections in the ventral thalamic group. i. Some mScarlet-positive fibres in the contralateral pedunculopontine nucleus of the mesencephalic locomotor region (MLR). Scale bars: 100 µm (a-i), 20 µm (ci). AP levels (from bregma): a: -5.80; c-i: -2.06, - 1.22, -1.94, -2.30, -2.06, -1.82, -4.84 mm. Micrographs: experiments repeated *n* = 3 times with similar results. Mouse head drawing adapted from [76].

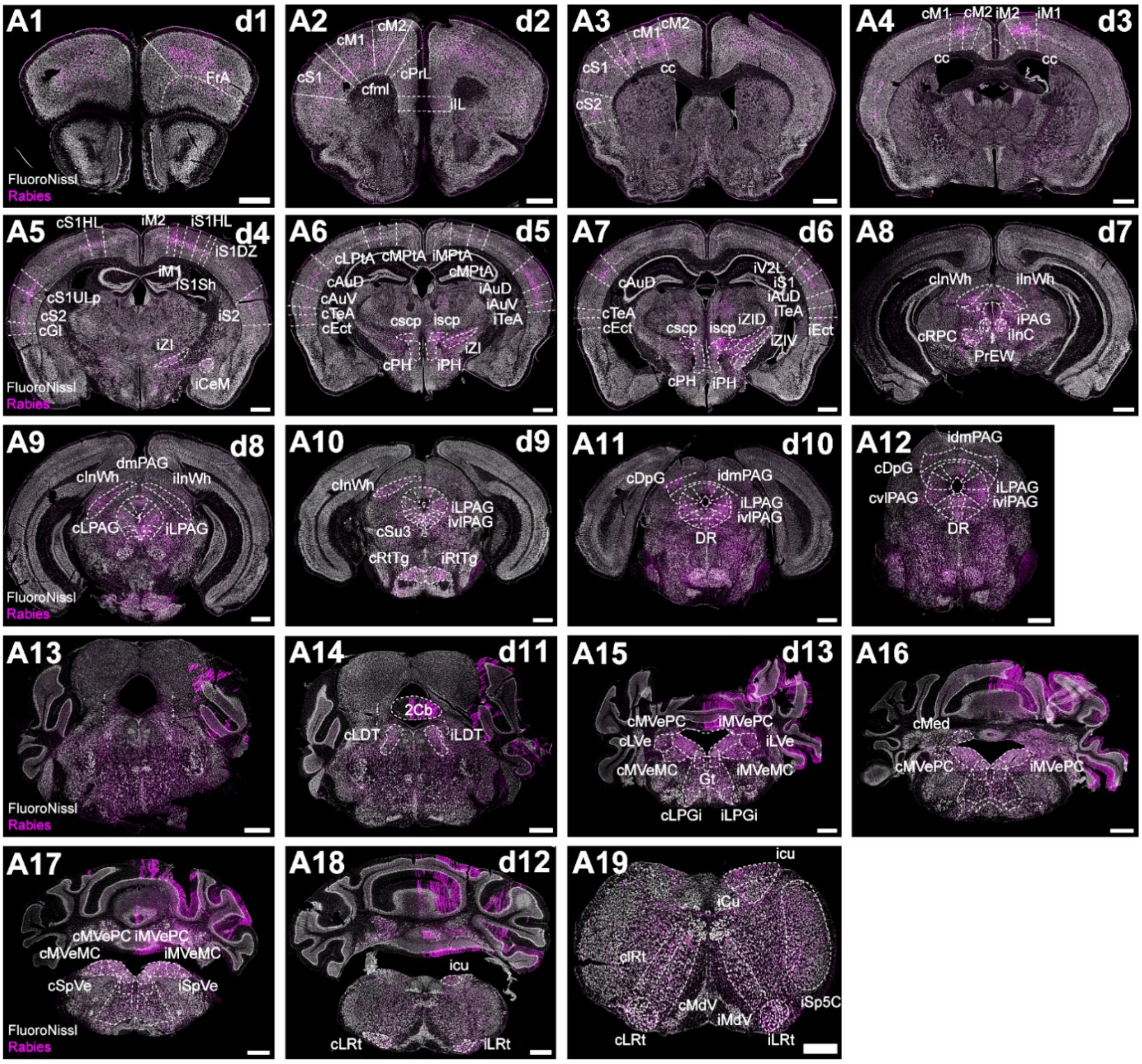

Supplementary Figure 5. Brain-wide inputs to the glutamatergic vestibular nucleus complex. Full slice images of all major coronal section levels along the rostro-caudal axis (rostral to caudal = ascending panel number) of the mouse brain of rabies tracings (from *n* = 3 mice). Each full slice image (A) shows its corresponding inset panel (d) from Figure 2d. Monosynaptic inputs to the VNC predominantly come from sensorimotor and frontal cortices (A1 - A6), sub- and hypothalamus (A5 - A8) as well as a diverse range of midbrain (including the periaqueductal gray (PAG), midbrain tegmentum) and cerebellar regions (lobules 2, 4, 5 and paraflocculus). Canonically expected are also inputs by oculomotor-related nuclei, such as the pre-Edinger Westphal (PrEW), nucleus of Darkschewitsch (Dk) or interstitial nucleus of Cajal (INC). Image left = Right hemisphere. Scale bars: all 1000 µm. Brain region abbreviations are taken from Allen Brain Atlas, v2017. AP levels (from bregma): A1 - A19: +2.68, +1.94, +0.74, -0.70, -1.06, -1.82, -2.30, -3.16, -3.64, -4.24, -4.48, -4.60, -5.02, -5.20, -5.80, -6.12, -6.72, -7.20 mm. Micrographs: experiments repeated *n* = 3 times with similar results.

Human A53T-mutated α-synuclein overexpression in the substantia nigra leads to nigrostriatal dopaminergic neurodegeneration and parkinsonian-like behaviour

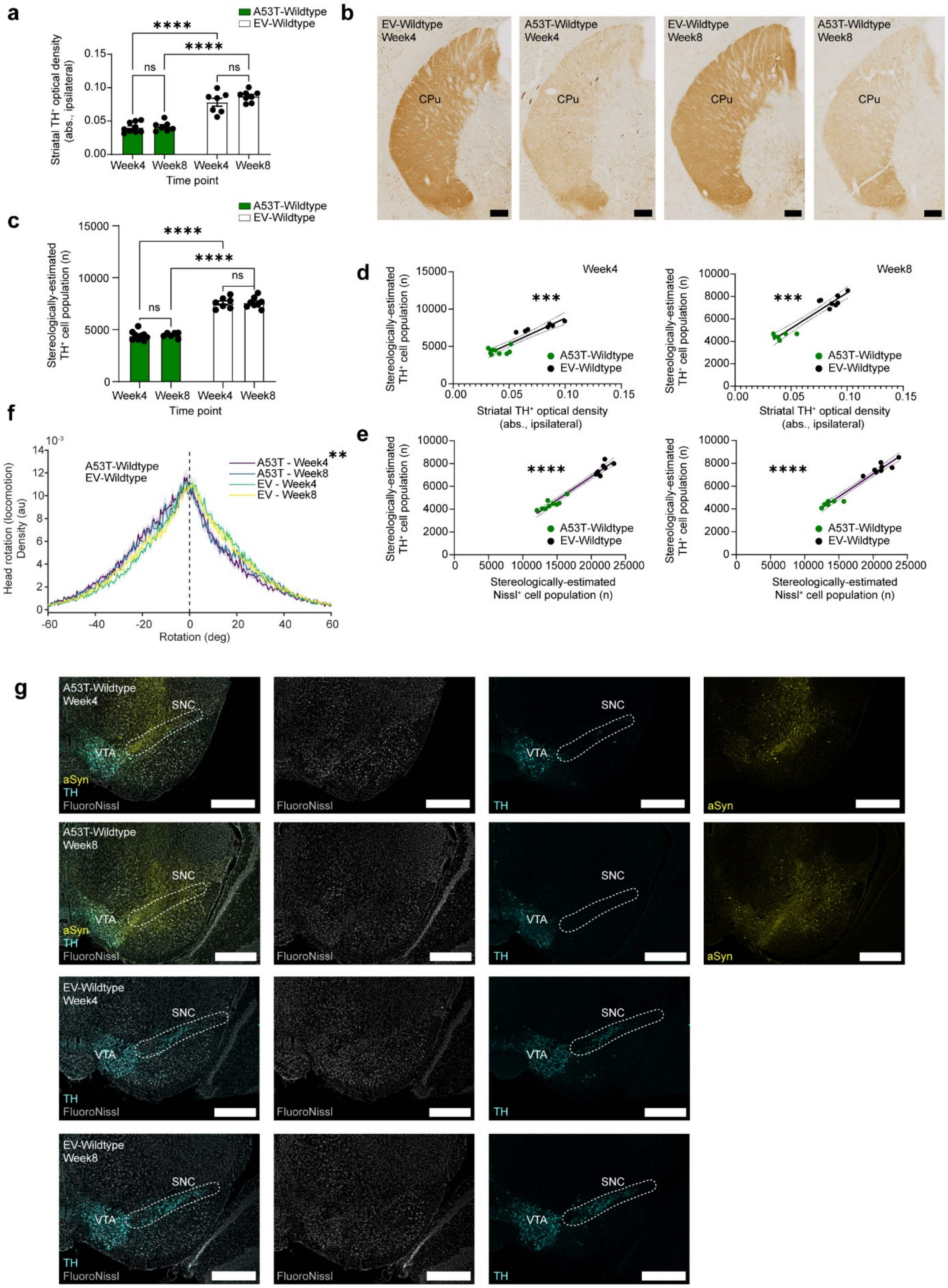

Supplementary Figure 6. Human A53T-mutated α-synuclein overexpression induces a parkinsonian (endo-)phenotype in mice. a. Striatal tyrosine hydroxylase (TH)-positive optical density (OD) showed non-progressive nigrostriatal projection loss at week 4 ($p < 0.0001$) and week 8 ($p < 0.0001$; two-way ANOVA: time point, $p = 0.1651$; group, $p < 0.0001$; interaction, $p = 0.3120$; two-sided Tukey tests; A53T-Wildtype-W4: $n = 9$, EV-Wildtype-W4: $n = 7$, A53T-Wildtype-W8: $n = 6$, EV-Wildtype-W8: $n = 8$). b. Representative images of TH+ nigrostriatal efferences in A53T-Wildtype and EV-Wildtype mice at weeks 4 and 8. Scale bars: 100 µm. c. Stereological estimation of TH-positive neurons in the substantia nigra pars compacta (SNC) showed neurodegeneration in A53T-Wildtype mice at weeks 4 and 8 ($p < 0.0001$; two-way ANOVA: time point, $p = 0.7995$; group, $p < 0.0001$; interaction, $p = 0.9031$; two-sided Tukey tests). a,c Data are mean ± s.e.m. d. SNC TH+ cells correlated positively with striatal TH+ OD at week 4 (Spearman's $r = 0.82$, $p < 0.001$; A53T-Wildtype: $n = 9$, EV-Wildtype: $n = 7$) and week 8 ($r = 0.83$, $p < 0.001$; A53T-Wildtype: $n = 6$, EV-Wildtype: $n = 8$). e. SNC TH+ cells strongly correlated with Nissl counts at week 4 ($r = 0.94$, $p < 0.0001$) and week 8 ($r = 0.93$, $p < 0.0001$), using sample sizes as in d. f. Keypoint-based kinematics showed heading bias in head rotation at week 4 but not week 8 (week 4: $p = 0.0013$; week 8: $p = 0.7966$; two-way ANOVA with two-sided Šídák's tests: week, $p = 0.3795$; group, $p = 0.0061$; interaction, $p = 0.0497$; sample sizes as in a). Head-rotation symmetry (not shown) differed at week 4 and week 8 (week 4: $p = 0.0106$; week 8: $p = 0.0019$; two-way ANOVA: week, $p = 0.3092$; group, $p < 0.0001$; interaction, $p = 0.3967$; sample sizes as in a). g. Ipsilateral SNC images of TH (cyan), alpha-synuclein (aSyn, yellow) and fluorescent Nissl stain (grey) show aSyn deposition and TH loss in A53T mice at weeks 4 and 8. Scale bars: 1000 µm. AP levels from bregma: b, -0.70 mm; g, -3.16 mm. Micrographs: experiments repeated $n = 3$ times with similar results.

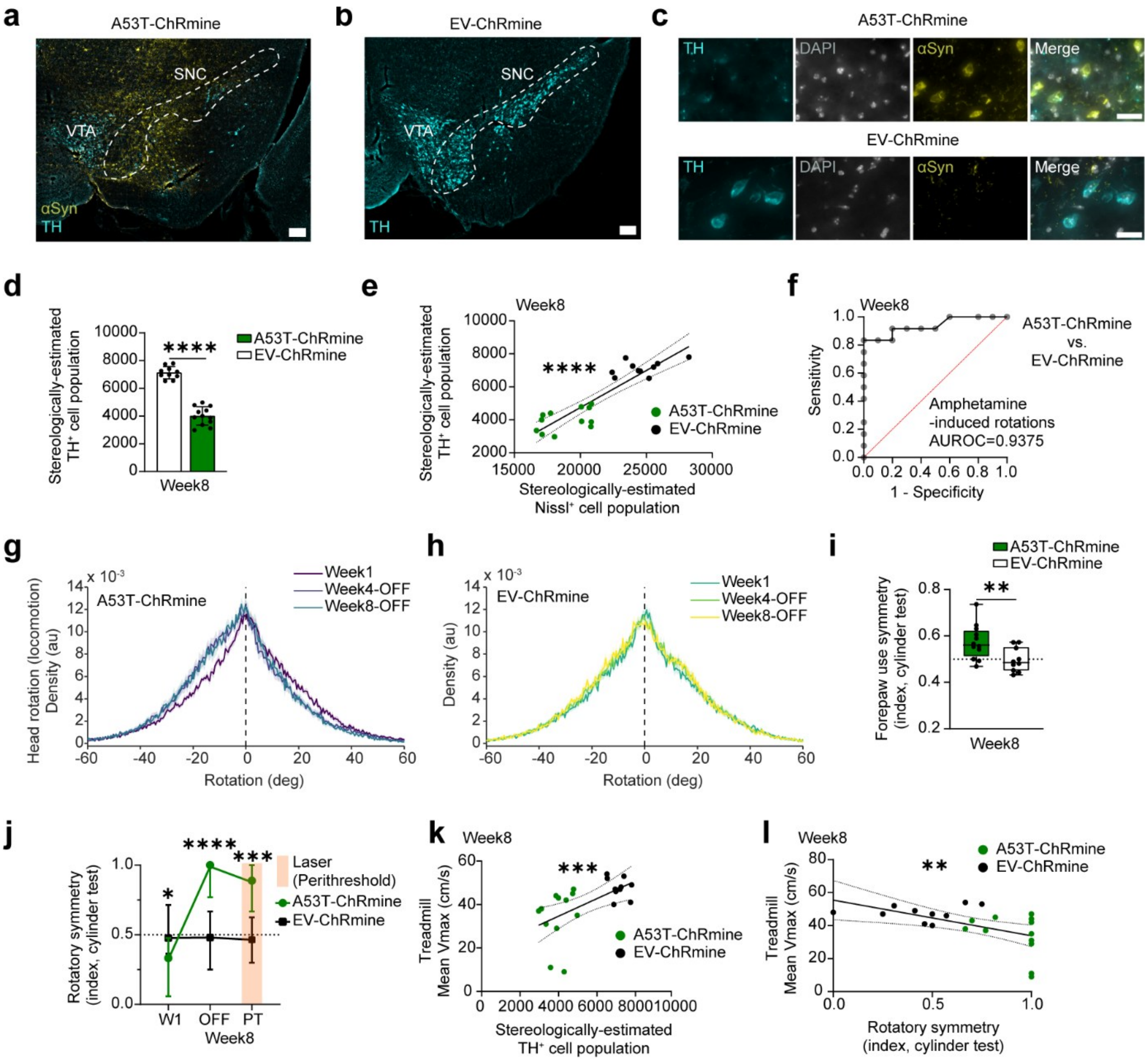

Supplementary Figure 7. Confirmation of parkinsonian (endo-)phenotype in mice receiving vestibular optoactivation. a. Example substantia nigra (SN) from an A53T-ChRmine mouse at week 8 showing peri-nigral α-synuclein deposition (aSyn, yellow) and loss of tyrosine hydroxylase-positive cells (TH, cyan) compared with EV-ChRmine (b). b. SN from an EV-ChRmine mouse showing preserved dopaminergic cells. c. TH, aSyn and DAPI immunoreactivity in the SN pars compacta (SNC) of A53T-ChRmine and EV-ChRmine mice, showing aSyn deposition only in A53T-ChRmine. Scale bars: 20 μm. d. Stereological TH+ SNC neuron counts were reduced in A53T-ChRmine mice (two-tailed unpaired *t*-test, $t = 12.74$, $p < 0.0001$; A53T-ChRmine: $n = 12$, EV-ChRmine: $n = 10$). Data are mean ± s.e.m. e. TH+ cells correlated with Nissl-stained neurons at week 8 (Spearman's $r = 0.8453$, $p < 0.0001$; A53T-ChRmine: $n = 12$, EV-ChRmine: $n = 10$). f. D-amphetamine verified nigrostriatal dopaminergic dysfunction: simple logistic regression discriminated A53T-ChRmine from EV-ChRmine mice using week-8 off total rotations (A53T-ChRmine: $n = 12$, EV-ChRmine: $n = 10$; AUROC = 0.9375, s.e. = 0.05201, $p = 0.0005$, Tjur's R squared = 0.6371). g-h. Keypoint-based kinematics showed development of significant locomotor heading bias in head-rotation distribution in A53T-ChRmine (g), but not EV-ChRmine (h), mice. i. Cylinder testing showed

increased forepaw use ipsilateral to injection in A53T-ChRmine mice (*n* = 12) versus EV-ChRmine mice (*n* = 10; mixed-effects model with two-sided Tukey tests: time/stimulation, $p = 0.1277$; group, $p = 0.0071$; interaction, $p = 0.2687$; week-8 off, $p = 0.0097$). Lines are medians, boxes interquartile ranges, whiskers extreme data points. j. A53T-ChRmine versus EV-ChRmine mice showed ipsilateral rotatory bias in the cylinder test at week-8 off ($p < 0.0001$). Data are median ± 95% CI. Maximum treadmill velocity correlated positively with TH+ cell count (k; week-8 off; A53T: *n* = 12, EV: *n* = 10; Spearman's $r = 0.6631$, $p = 0.0008$) and negatively with rotatory asymmetry (l; cylinder test, week-8 off; Spearman's $r = -0.6045$, $p = 0.0029$). Lines are linear intercepts; dotted lines, 95% CI. Scale bars: a,b, 200 μm. AP from bregma: a-b, -3.20 mm. Micrographs: experiments repeated *n* = 3 times with similar results.

Sham excitation of the vestibular nucleus complex does not lead to vestibular symptoms or kinematic changes

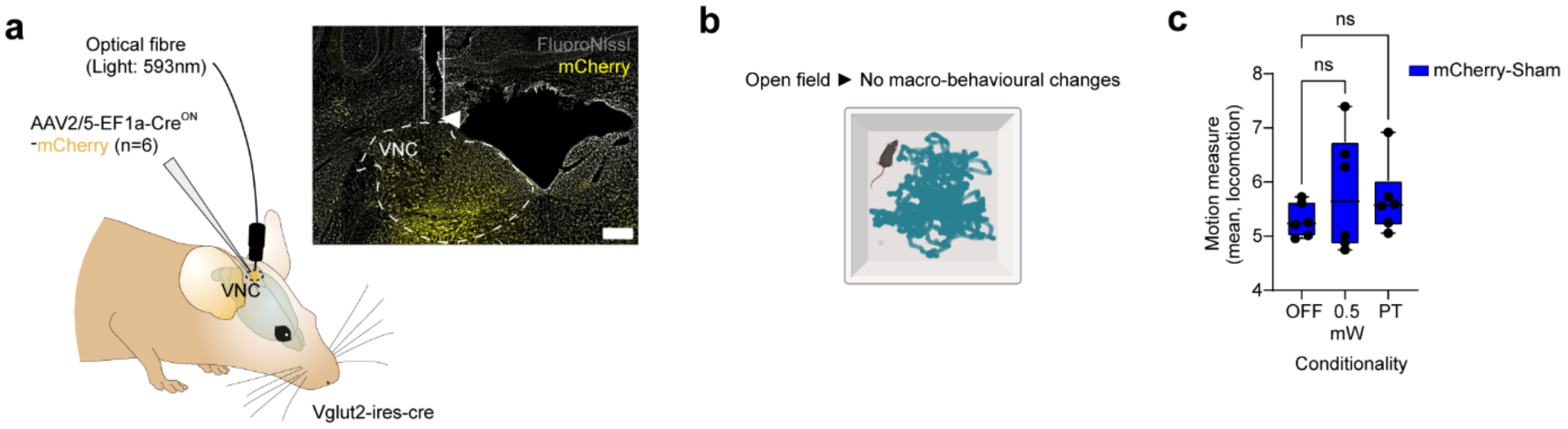

Supplementary Figure 8. Sham optogenetics control group. a. Depiction of sham optogenetics group (n = 6, sham VNC-mCherry cohort 1). Scale bar: 500 µm. Arrow points at optical fibre tip. AP: -6.12 mm from bregma. b. Upon light activation until 0,5 mW, sham mCherry mice did not show any form of similar acute vestibular symptoms (see Supplementary Video 4). c. Neither sham perithreshold (PT) illumination (using mean PT laser power across of EV-ChRmine cohort (week8): mean ± s.e.m.: 9,857 ± 1,438 µW), nor illumination at high suprathreshold range (0,5 mW) lead to changes in motion (repeated measures one-way ANOVA, F = 0,6404, p = 0.5130, two-sided Dunnett's multiple comparisons test; n = 6). Open field sketch sketch created in BioRender. Ip, A. (2026) https://BioRender.com/7uh3a5l. Micrographs: experiments repeated n = 3 times with similar results. Mouse head drawing adapted from [76].

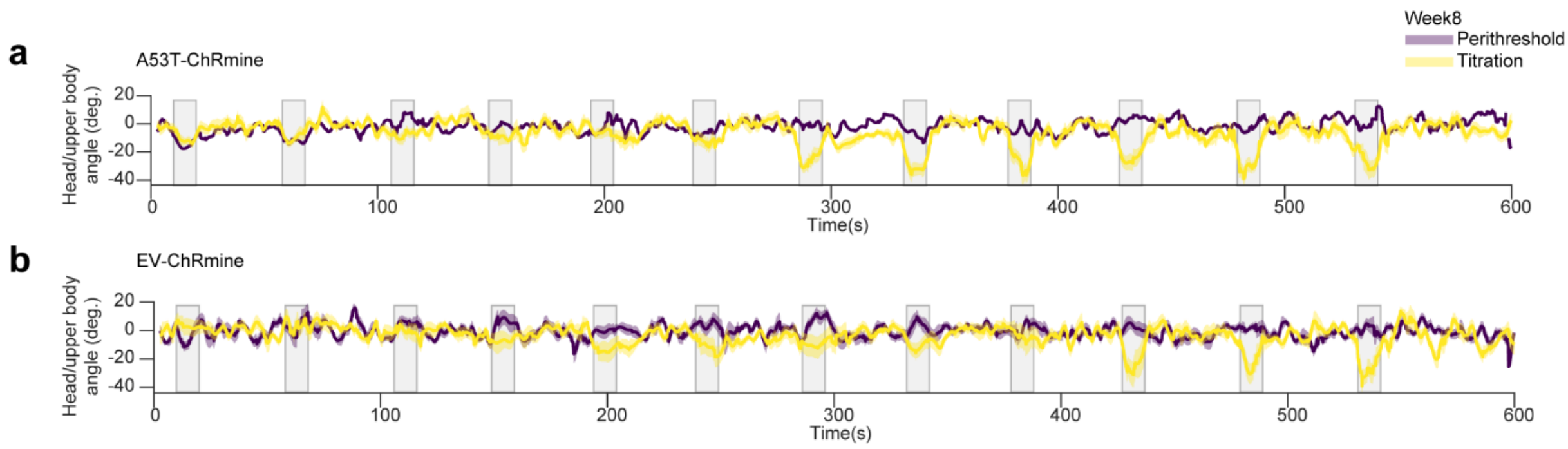

Supplementary Figure 9. Incremental vestibular optoactivation leads to progressive, yet quickly reversible postural aberrations. a. Group-averaged whole trace across week8 titration recordings of A53T-ChRmine group (n = 12) displaying head-to-upper body angle (in degrees) demonstrating progressive, yet temporary and quickly reversibly postural alterations synched with optogenetic pulse trains (grey blocks). b. Group-averaged whole trace across week8 titration recordings of EV-ChRmine group (n = 10) displaying identically disruptive effects, yet on average with more advanced pulse trains. Data show group mean ± s.e.m.

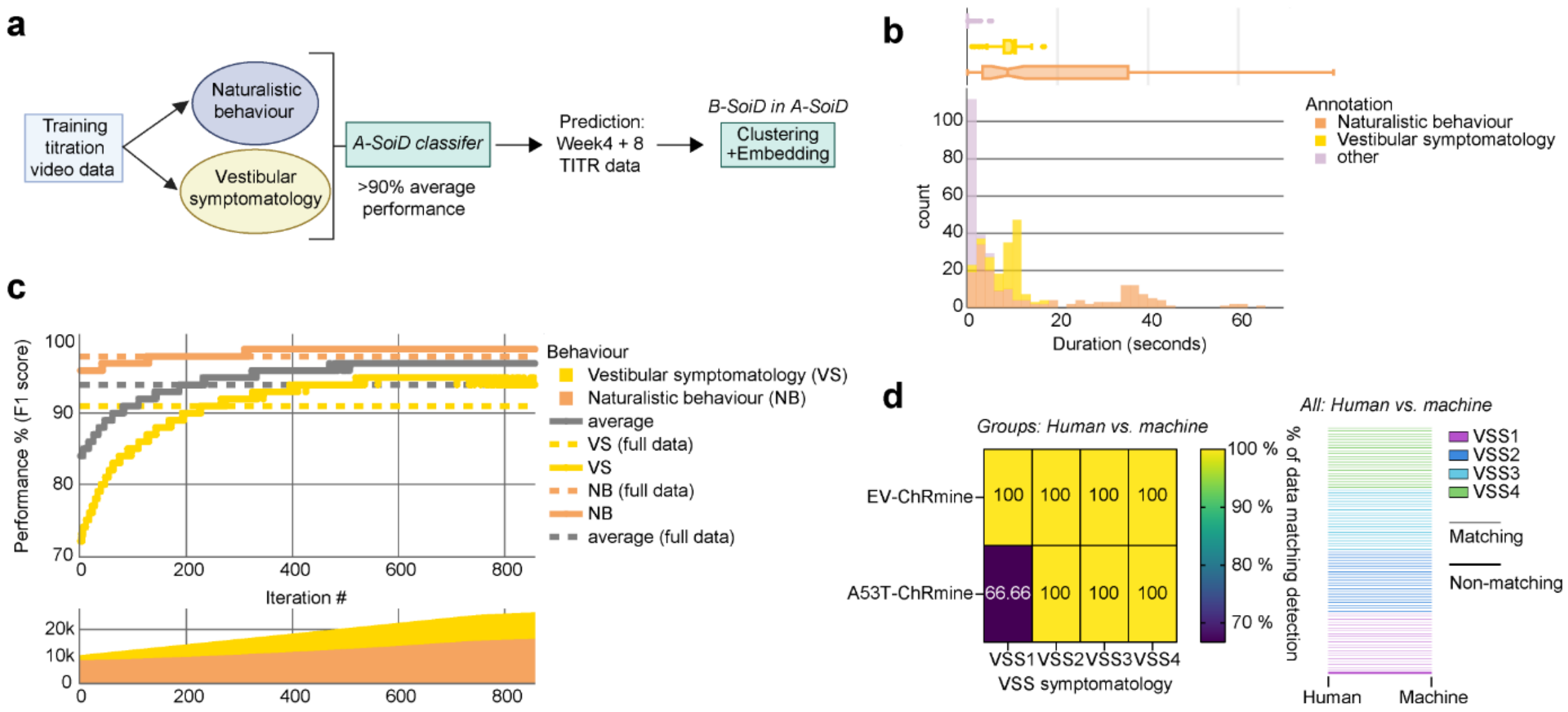

Supplementary Figure 10. (Semi-)supervised machine learning workflow and methodology via A-SoiD. a. Flow chart representing the model/classifier training and data used. The active learning classifier was trained on a total of *n* = 45 videos [diverse snippet videos (*n* = 38) and full videos (10 min; *n* = 7)] from titration at week4 and week8, thus generating a balanced training dataset. Videos were taken from A53T-ChRmine and EV-ChRmine mice. b. Frequency distribution of different durations of symptoms per category. 'other' represents noise from unannotated singular frames. c. Results from the active learning classifier trained by the A-SoiD pipeline [49]. Detection performance of both the VSS-like symptoms (*'Vestibular Symptomatology'*) and naturalistically occurring behaviour (*'Naturalistic behaviour'*) was high (91% and 98%, respectively). c. Frequency distribution of different durations of symptoms per category. *'Other'* represents noise from unannotated singular frames. d. Matching detection of VSS symptoms as predicted by a machine learning approach (A-SoiD [49]) vs. subjective rating (*n* = 22 videos, week8 titration, A53T: *n* = 12, EV: *n* = 10). Group-stratified matching analysis shows solid matching except for VSS1 of A53T-ChRmine group (66,66%) [A53T-ChRmine (Human/Machine): VSS1: 3/2, VSS2: 8/8, VSS3: 3/3, VSS4: 3/3; EV-ChRmine (Human/Machine): VSS1: 2/2, VSS2: 5/5, VSS3: 3/3, VSS4: 3/3]. (Video-level scoring, *n* = 2 raters, Right-hand plot: Line thickness differentiates matching vs. non-matching identification. Sketch in a created in BioRender.com.

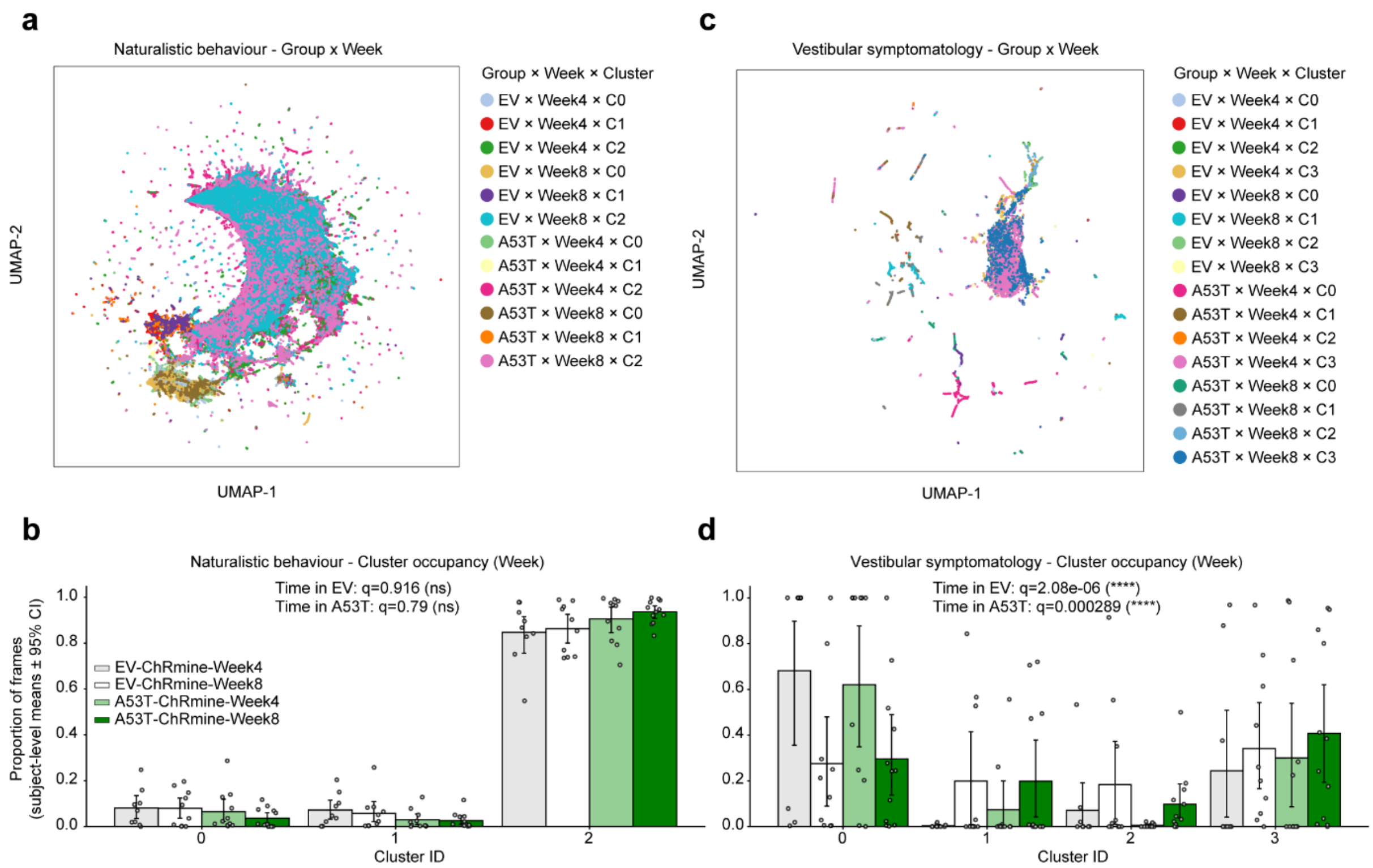

Supplementary Figure 11. Longitudinal cluster occupancy of naturalistic behaviour vs. vestibular symptomatology in A53T vs. EV mice. a,c. UMAP plots show the embedding of naturalistic behaviour (a) and vestibular symptomatology (c) during systematic titration at both weeks 4 and 8. Colors indicate cluster occupancy per group and across time. b,d. Proportion of frames per group and time point in each cluster of naturalistic behaviour (b) and vestibular symptomatology (d). showed a trend for differential cluster occupancy of A53T vs. EV (multinomial logistic regression with FDR/Holm-adjusted Wald tests) across time points.

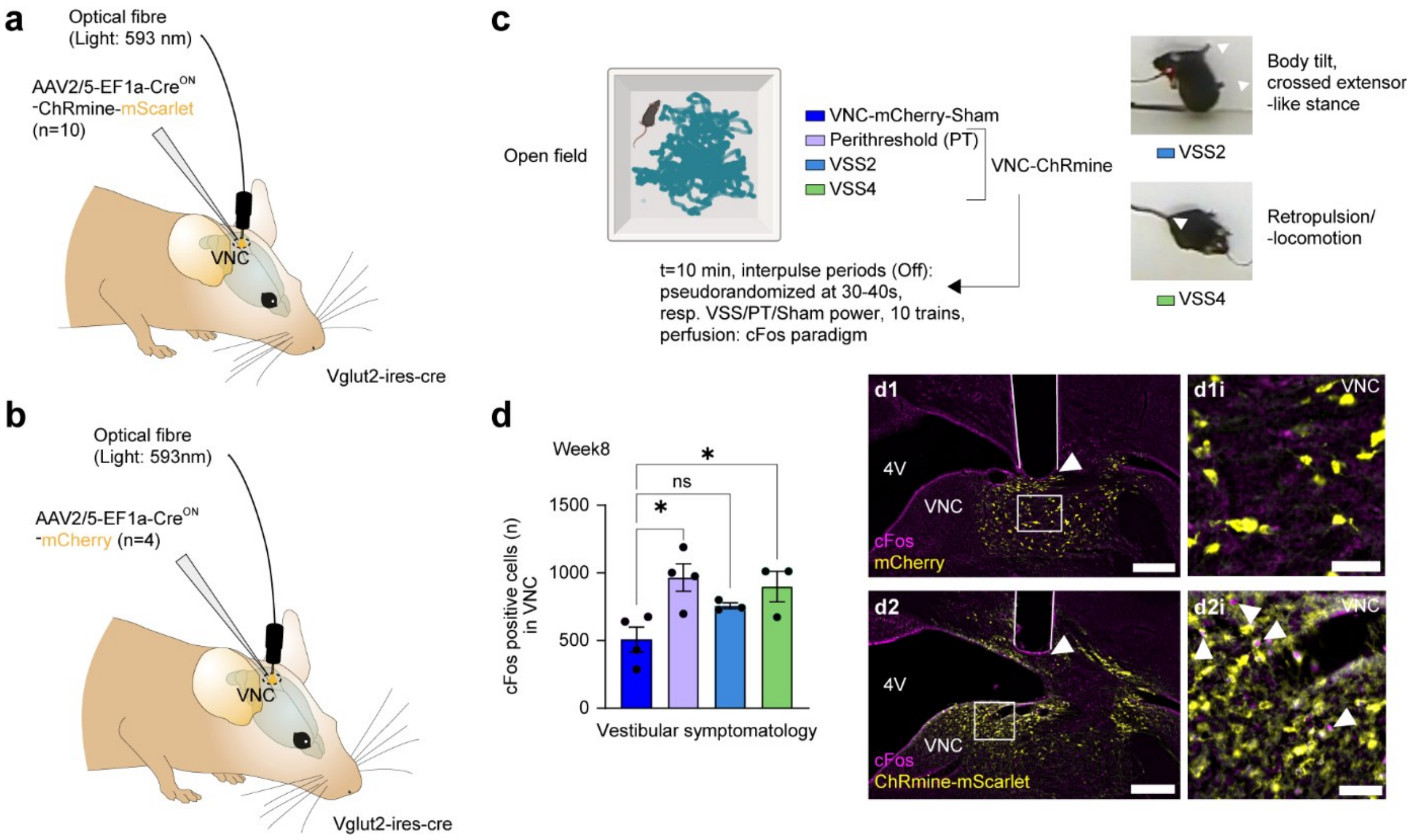

Supplementary Figure 12. Perithreshold vs. suprathreshold optoactivation of vestibular nucleus complex does not correlate with enhanced VNC *c-Fos* expression. a. We again used VNC-ChRmine mice (*n* = 10 total) that were stimulated with an identical protocol over 10 minutes with 3 different subgroups under respectively different conditions: perithreshold (*n* = 4 mice), VSS2 symptomatology (*n* = 3 mice) and VSS4 symptomatology (*n* = 3 mice). b. A sham-stimulated VNC-mCherry group (sham VNC-mCherry cohort 2, *n* = 4 mice) was used as well, stimulated at pseudo-threshold level (median perithreshold laser power of VNC-ChRmine group. c. We conducted an open field (OF) experiment stimulating at the different symptomatology/laser power paradigms (perithreshold, sham perithreshold, VSS2, VSS4). All mice were perfused 90 min after termination of a 10 min OF optoactivation experiment at week8. d. The QUINT framework (see Methods) was used and *c-Fos* expression in the VNC was quantified. We found that perithreshold and VSS4 optoexcitation leads to enhanced VNC c-Fos expression vs. sham (ordinary one-way ANOVA, $p = 0.0199$, $F = 5{,}230$, two-sided Dunnett's multiple comparisons test), but VSS2 stimulation does not, yet, showing a trend. Scale bars: 500 µm (d1,d2), 100 µm (d1i, d2i). d1-AP: -6.00 mm from bregma, d2-AP: -6.12 mm from bregma. Open field sketch sketch created in BioRender. Ip, A. (2026) https://BioRender.com/7uh3a5l. Micrographs: experiments repeated *n* = 3 times with similar results. Mouse head drawing adapted from [76].

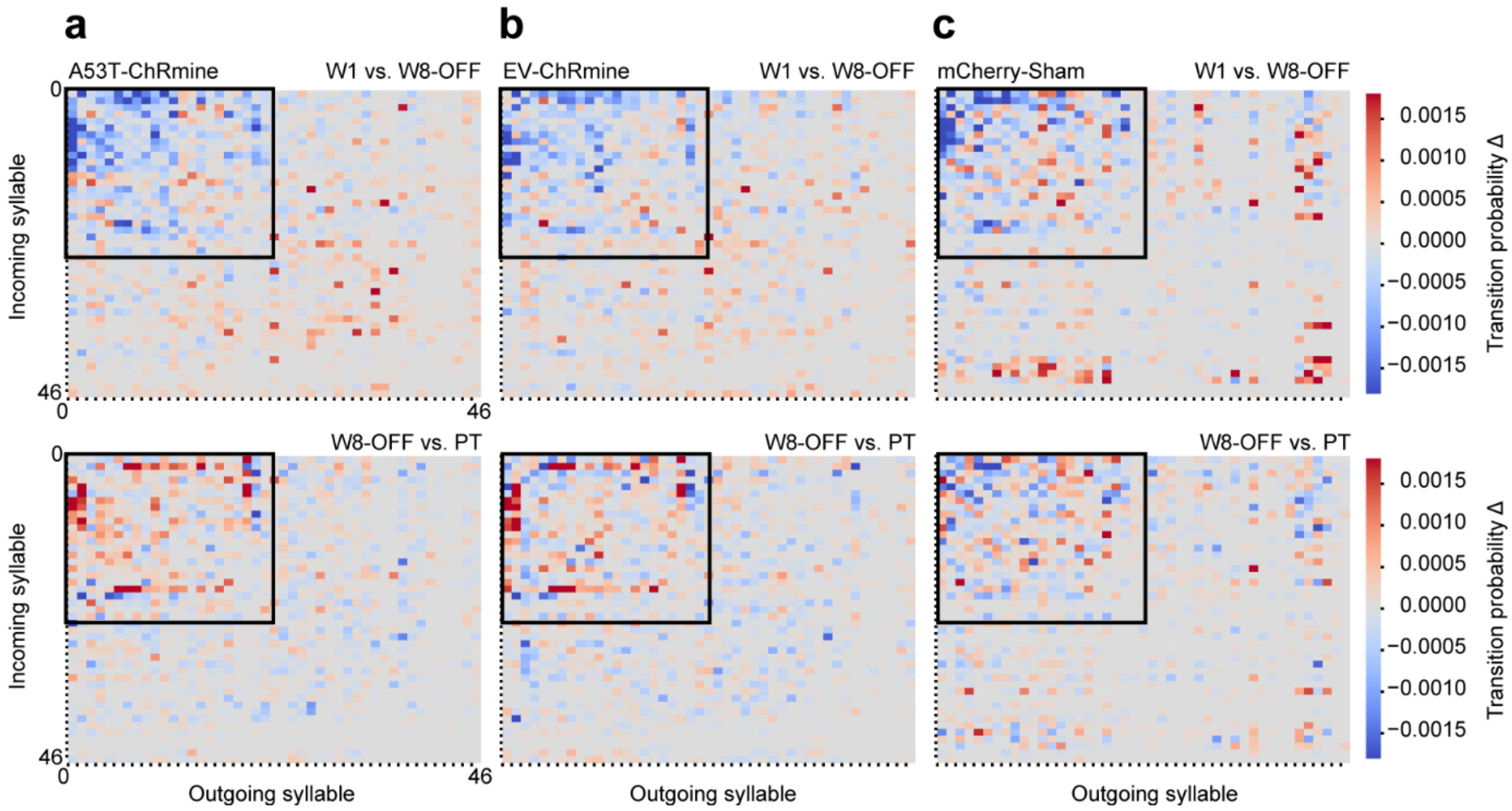

Supplementary Figure 13. Matrices of behavioural motif transitions mark distinct transition probability changes of behavioural modules with optoactivation of excitatory VNC. a. Transition matrices displaying transition probability differences between syllables comparing conditions (week1, week8: off, (sham)perithreshold stimulation) of vestibular nucleus complex (VNC)) and experimental group (A53T-ChRmine, EV-ChRmine, sham-stimulated mCherry). A53T-ChRmine show pronounced downregulation with time/habituation and disease model development (top), while perithreshold stimulation leads to upregulation of transitions in frequently occurring syllables. b. EV-ChRmine mice show less pronounced, yet similar change with time/habituation (top) and perithreshold stimulation (bottom). c. Sham mCherry mice also show some changes with time, however a different pattern with sham stimulation (bottom). Color bars indicate transition probability delta. X and Y-axis squares plot identical orders of syllables (0-46 both). Low frequency syllables excluded (< 0.005). Squares indicated frequently occurring syllables showing visually most apparent reconfiguration.

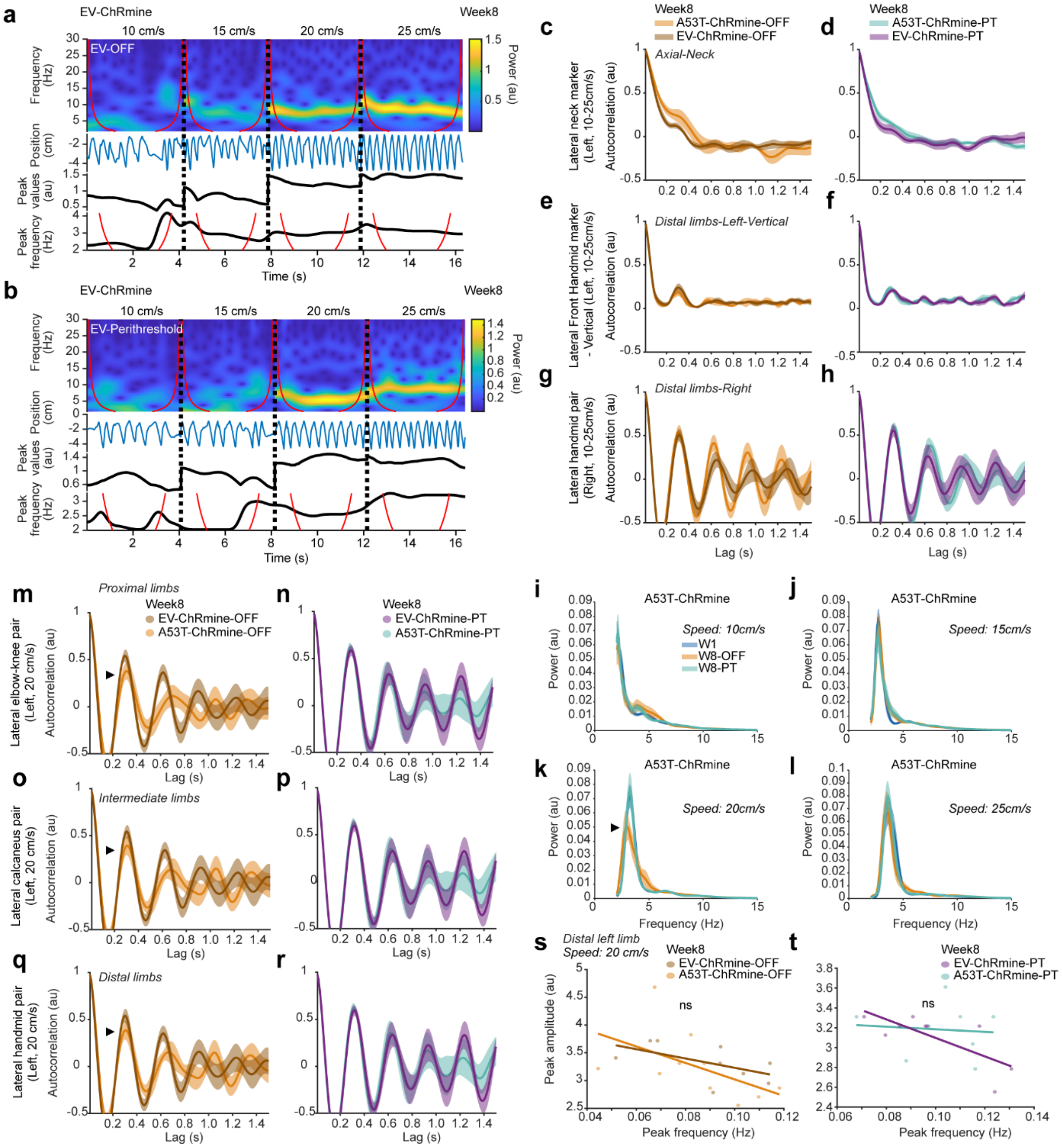

Supplementary Figure 14. Autocorrelograms of neck, right markers and vertical plane highlight therapeutic oscillatory changes and deficits specific to left-sided limb markers. Example scalograms (week8) of keypoint-based gait analysis of the left, lateral-view handmid marker of a single EV-ChRmine animal in off (a.) and with perithreshold (PT) VNC optoactivation (b.) of the excitatory VNC at week8 shows similarly increased power in peak frequencies, however less pronounced changes compared to A53T-ChRmine (see Fig. 5). c. Lateral-view axial neck marker (x-coordinate = horizontal) shows trends for increased autocorrelation in secondary peak in A53T- vs. EV-ChRmine mice, mildly reduced by PT (d.). e. Lateral-view left front paw marker (in y-coordinate = vertical) in A53T-ChRmine shows np change in off vs. with PT VNC optoactivation (f.). g. Lateral-view right front paw marker (x-coordinate = horizontal) shows no prominent autocorrelation changes in off or with VNC optoactivation (PT, h.). i-l. Periodograms show that loss of power at gait frequencies and alleviation by PT VNC actuation in A53T-ChRmine

is mainly driven by changes at 20 cm/s. m-r. Plots show loss of autocorrelation across left-sided limb markers in A53T- vs. EV-ChRmine mice at 20 cm/s speed and ‘harmonization’ of oscillatory pattern with VNC PT optoactivation. s-t. No differences in correlation between peak amplitude and frequencies of gait at 20 cm/s speed between A53T- and EV-ChRmine at off and with PT optoactivation of VNC (both $p > 0.05$). Autocorrelation values are mean ± s.e.m. Red lines indicate cone of influence for wavelet analysis. s,t: one-sided F-tests, lines are intercepts.

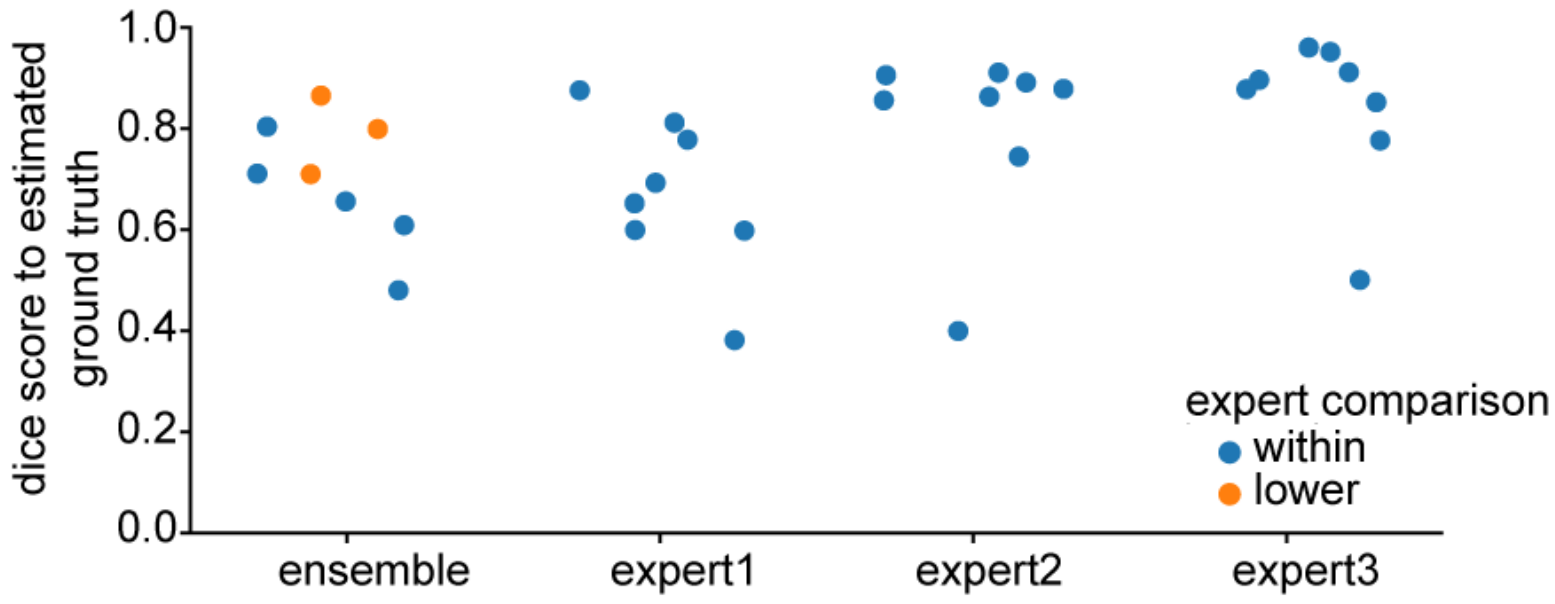

Supplementary Figure 15. Performance of *deepflash2* convolutional neural network (CNN) model ensemble against raters for ground truth in *c-Fos* quantification. Ground truth for c-Fos quantification of each rater (*n* = 3, J.H., T.P., D.D.) against the model ensemble. Data shows the dice scores to the estimated ground truth. For the most part, the model performed at least as well as any one of the experts. Blue dots are within expert range regarding scoring, orange being lower performance vs. expert range.

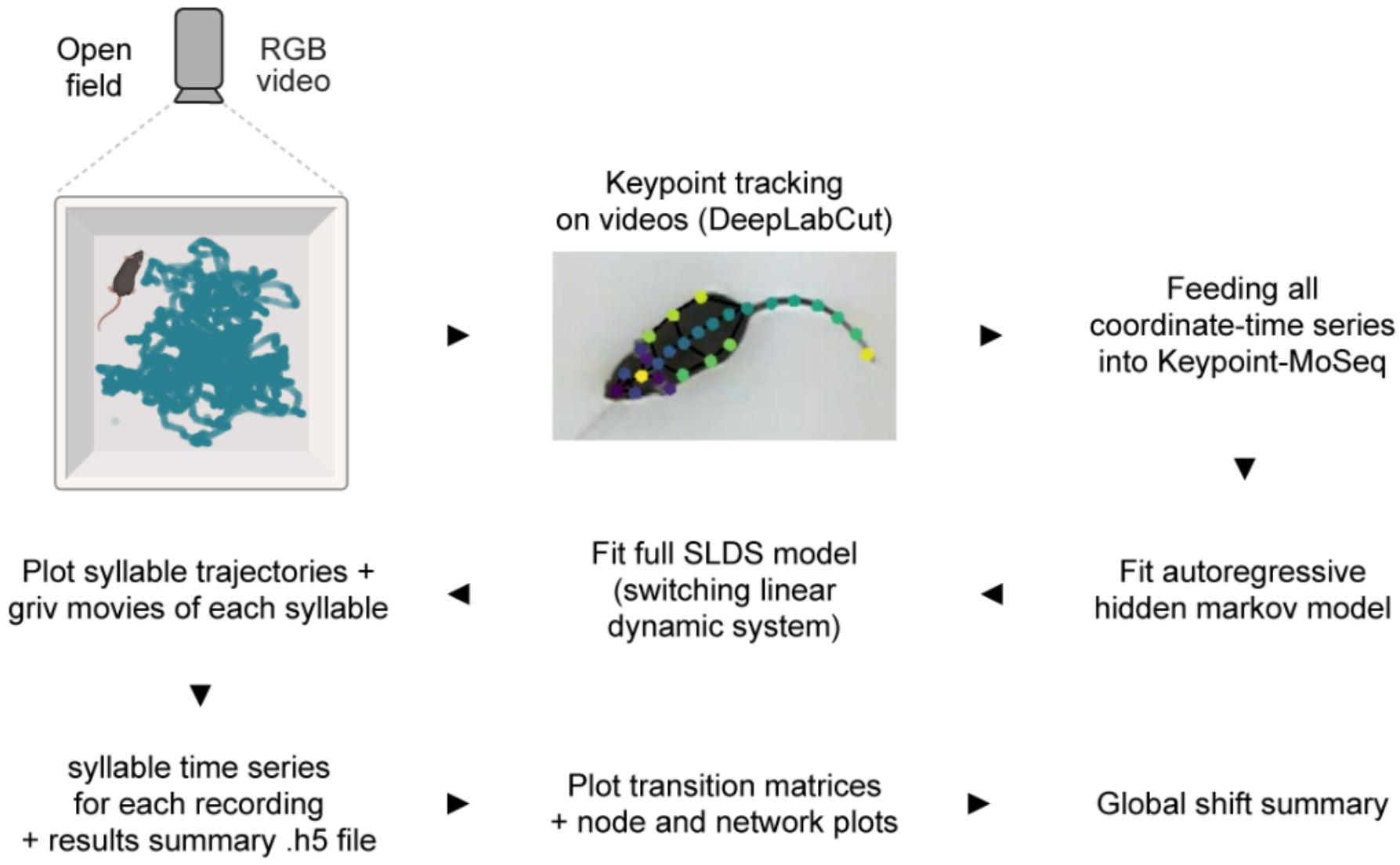

Supplementary Figure 16. Workflow overview for analysis of unbiased behavioural motifs from Keypoint-MoSeq (KPMS). We took videos recorded from mice in an open field setting and fed them into DeepLabCut (DLC) for pose estimation. Coordinate-time series from DLC were then fed into Keypoint-Moseq to first fit an autoregressive hidden Markov model (AR-HMM). On that basis, a full SLDS (switching linear dynamic system) model was fit. The output yielded a .h5 file containing all data and syllable-time series as well as syllable features (such as frequency). Syllables at a frequency cutoff < 0.005 (40, 45, 47, 48) across conditions were excluded (see Supplementary Table 8). After, we plotted transition matrices and node and network plots and computed a global shift summary. Open field sketch sketch created in BioRender. Ip, A. (2026) https://BioRender.com/7uh3a5l.

Supplementary Tables

Comparison	DI	p_dir_one_sided	p_L1	p_F	Interpretation
A53T BL→W8	0	0,04148	0,0004998	0,004998	Major reorganization of transitions; strong L1/F effects; weak directionality.
A53T W8 OFF→PT	0	0,7436	0,02399	0,04798	Moderate opto-induced changes; no directionality.
EV BL→W8	0	0,06397	0,04148	0,1904	Mild drift over time; small L1 change; no strong F change.
EV W8 OFF→PT	0	0,2579	0,006997	0,002999	Clear PT–OFF effect; strong L1/F without directionality.
mCherry BL→W8	0	0,6797	0,008496	0,008496	Moderate transition drift with aging/training.
mCherry W8 OFF→PT	0	0,1024	0,8386	0,8541	No detectable opto-induced change; transitions remain stable.

Supplementary Table 1. Global shift summary. Systematic presentation of global transition-matrix shift permutation analysis results. Global changes in behavioural transition structure were quantified from group-level normalized bigram transition matrices using sequence-level permutation testing. For each comparison, transition matrices were constructed on the same filtered syllable node set and compared using the Direction Index (DI), L1 norm, and Frobenius norm. DI quantifies the net signed reallocation of transition probabilities between conditions and was assessed using an exact one-sided permutation p-value in the direction of the observed shift. The L1 and Frobenius norms quantify the overall magnitude of transition-matrix reorganization irrespective of direction and were therefore assessed using upper-tail permutation p-values. Permutations preserved group sizes and were performed at the sequence level. Where multiple pairwise comparisons were performed, p-values were Bonferroni-corrected within each p-value family. Significant L1 and/or Frobenius effects indicate global reorganization of behavioural transitions, whereas significant DI indicates a consistent directional imbalance in transition-probability redistribution.

```yaml
#==================== FITTING =====================#
[redacted]
# upper bound of uniform noise added to the data during initial AR-HMM fitting; this is used to regularize the model
added_noise_level: 0.1
[redacted]
# number of frames used to fit the PCA model during initialization
PCA_fitting_num_frames: 1000000
[redacted]
# used to define outliers for interpolation when the model is initialized
conf_threshold: 0.5
[redacted]
#================== HYPER PARAMS ==================#
# parameters to convert neural net likelihoods to error size priors
error_estimator:
  intercept: 0.25
  slope: -0.5
# keypoint observation hyperparameters
obs_hypparams:
  nu_s: 5
  nu_sigma: 100000.0
  sigmasq_0: 0.1
  sigmasq_C: 0.1
# autoregressive hyperparameters
ar_hypparams:
  K_0_scale: 10.0
  S_0_scale: 0.01
  latent_dim: 5
  nlags: 3
# transition hyperparameters
trans_hypparams:
  alpha: 5.7
  gamma: 1000.0
  kappa: 1000000.0
  num_states: 100
# centroid movement hyperparameters
cen_hypparams:
  sigmasq_loc: 2.3392014691478034
#===================== OTHER ======================#
# suffix used to match videos to recording names; this can usually be left empty (see `util.find_matching_videos` for details)
recording_name_suffix: ''
# whether to print progress messages during fitting
verbose: false
# pseudocount used regularize neural network confidences
conf_pseudocount: 0.001
# directory with videos from which keypoints were derived (used for crowd movies)
video_dir: /workspace/data
# colormap used for visualization; see `matplotlib.cm.get_cmap` for options
keypoint_colormap: autumn
# whether to whiten principal components; used to initialize the latent pose trajectory `x`
whiten: true
# whether to keep the heading angle fixed; this should only be True if the pose is constrained to a narrow range of angles, e.g. a headfixed mouse.
fix_heading: false
# frames per second of the video recordings
fps: 25
outlier_scale_factor: 6.0
```


Supplementary Table 2. Keypoint-MoSeq model hyperparameter summary. Full config.yaml file of hyperparameters of our Keypoint-MoSeq model.

test	wald_stat	df	p_value	q_value	stars(q)	mcp_method
EV vs A53T (overall across weeks)	22,32557087	3	5,58076E-05	8,37113E-05	****	fdr_bh
Time effect in EV (Week8 vs Week4)	31,42367134	3	6,9218E-07	2,07654E-06	****	fdr_bh
Time effect in A53T (Week8 vs Week4)	18,8805326	3	0,000289398	0,000289398	****	fdr_bh
**chi2_stat**	**dof**	**p_chi2**	**perm_p**			
2100,939646	9	0	0,6885			
**comparison**	**JS_div_bits**	**JS_dist**	**Aitchison**			
EV vs A53T (overall)	0,027646553	0,166272525	1,018792622			
Time in EV (W8 vs W4)	0,176108363	0,419652669	3,49579993			
Time in A53T (W8 vs W4)	0,105041103	0,324100452	2,578026669			
**cluster**	**EV_mean**	**EV_CI_low**	**EV_CI_high**	**A53T_mean**	**A53T_CI_low**	**A53T_CI_high**
0	0,245809687	0,057739182	0,472951157	0,340342922	0,182683872	0,525641211
1	0,152113023	0,011111293	0,337563745	0,18522447	0,056378795	0,337182803
3	0,402551036	0,19220907	0,648940016	0,398989469	0,215872964	0,584664796
2	0,199526253	0,026819331	0,401310949	0,075443139	0,015961387	0,160089053
**group**	**week**	**cluster**	**mean**	**CI_low**	**CI_high**	
A53T	Week4	0	0,621935874	0,359895017	0,877034632	
A53T	Week4	1	0,074026993	0,000589681	0,178106358	
A53T	Week4	3	0,298100866	0,071923315	0,535102471	
A53T	Week4	2	0,005936268	0,001919192	0,010521268	
A53T	Week8	0	0,297233288	0,137635765	0,484831064	
A53T	Week8	1	0,198123693	0,043145367	0,377535173	
A53T	Week8	3	0,40671232	0,197307785	0,61976401	
A53T	Week8	2	0,097930699	0,037248079	0,187431001	
EV	Week4	0	0,68128585	0,362462659	0,897643098	
EV	Week4	1	0,004214219	0,00076365	0,009002751	
EV	Week4	3	0,244197981	0,041750842	0,508199002	
EV	Week4	2	0,07030195	0,002290951	0,189357197	
EV	Week8	0	0,275031978	0,0893335	0,506408018	
EV	Week8	1	0,199203113	0,008849558	0,396194863	
EV	Week8	3	0,341517065	0,155913113	0,538280495	
EV	Week8	2	0,184247844	0,01253655	0,38660355	
**cell sizes - group**	**week**	**n_subjects**	**n_frames**			
A53T	Week4	11	6815			
A53T	Week8	12	4663			
EV	Week4	9	1391			
EV	Week8	10	2058			

Supplementary Table 3. Statistical results of 'Vestibular symptomatology' via multinomial regression. Summary of multinomial logistic regression analyses comparing vestibular symptomatology clusters between EV and A53T mice and across time points. Wald χ^2 statistics with 3 degrees of freedom are reported for the prespecified contrasts, with p-values adjusted for multiple testing using the Benjamini–Hochberg false discovery rate method. Omnibus model fit is summarized by likelihood-ratio χ^2 testing and permutation-based model assessment. Effect-size summaries include Jensen–Shannon divergence, Jensen–Shannon distance, and Aitchison distance. Cluster-wise estimated mean probabilities are reported with confidence intervals, together with group-, week-, and cluster-level sample sizes.

test	wald_stat	df	p_value	q_value	stars(q)	mcp_method
EV vs A53T (overall across weeks)	6,011909641	2	0,049491476	0,148474429	ns	fdr_bh
Time effect in EV (Week8 vs Week4)	0,174421446	2	0,916483951	0,916483951	ns	fdr_bh
Time effect in A53T (Week8 vs Week4)	1,283199607	2	0,526449534	0,789674301	ns	fdr_bh
**chi2_stat**	**dof**	**p_chi2**	**perm_p**			
8974,636164	6	0	0,4935			
**comparison**	**JS_div_bits**	**JS_dist**	**Aitchison**			
EV vs A53T (overall)	0,005972973	0,077285012	0,535315561			
Time in EV (W8 vs W4)	0,000651082	0,025516305	0,185751931			
Time in A53T (W8 vs W4)	0,003170504	0,056307233	0,450411264			
**cluster**	**EV_mean**	**EV_CI_low**	**EV_CI_high**	**A53T_mean**	**A53T_CI_low**	**A53T_CI_high**
0	0,075475916	0,034882968	0,117167544	0,053011849	0,029532303	0,078302562
1	0,064174209	0,035891113	0,099985408	0,032049084	0,016102831	0,051211463
2	0,860349875	0,799422057	0,916805962	0,914939067	0,888186099	0,940738511
**group**	**week**	**cluster**	**mean**	**CI_low**	**CI_high**	
A53T	Week4	0	0,065330938	0,022054607	0,119350332	
A53T	Week4	1	0,029487717	0,008610879	0,054867626	
A53T	Week4	2	0,905181346	0,845778159	0,956705112	
A53T	Week8	0	0,036783127	0,01594674	0,060253921	
A53T	Week8	1	0,026481718	0,011023156	0,046242138	
A53T	Week8	2	0,936735155	0,907253751	0,962491763	
EV	Week4	0	0,080985641	0,035189005	0,135084808	
EV	Week4	1	0,072350719	0,032557576	0,115231273	
EV	Week4	2	0,84666364	0,75584946	0,915039435	
EV	Week8	0	0,079465917	0,036338369	0,124528117	
EV	Week8	1	0,057762756	0,021038969	0,109275556	
EV	Week8	2	0,862771326	0,800638537	0,925480558	
**cell sizes - group**	**week**	**n_subjects**	**n_frames**			
A53T	Week4	11	158163			
A53T	Week8	12	175555			
EV	Week4	9	133589			
EV	Week8	10	148066			

Supplementary Table 4. Statistical results of 'Naturalistic behaviour' via multinomial regression. Summary of multinomial logistic regression analyses comparing naturalistic behavioural-state distributions between EV and A53T mice and across time points. Wald χ^2 statistics with 2 degrees of freedom are reported for prespecified group and time contrasts, with p-values adjusted for multiple testing using the Benjamini–Hochberg false discovery rate method. Omnibus model fit is summarized by likelihood-ratio χ^2 testing and permutation-based model assessment. Effect-size summaries include Jensen–Shannon divergence, Jensen–Shannon distance, and Aitchison distance. Cluster-wise estimated mean probabilities are reported with confidence intervals, together with group-, week-, and frame-level sample sizes.

Frequency (Hz)	Duty Cycle (%)	Pulse Width (ms)	Effective Period (ms)
20	20	10	50
1	50	500	1000
20	20	10	50
1	50	500	1000
20	20	10	50
1	50	500	1000
20	20	10	50
1	50	500	1000

Time OFF (ms)	Number of Pulse per Sequence	Num Pulses (total)	Number of Sequence(s)
40	200	600	3
500	33	33	1
40	200	600	3
500	37	37	1
40	200	600	3
500	39	39	1
40	200	600	3
500	59	59	1

Delay Between Sequence(s) (ms)	Sequence Duration (ms)	Train Duration (ms)	Starting Delay (ms)	Total Duration (ms)
38000	10000	106000	10000	116000
0	33000	33000	116000	33000
35000	10000	100000	149000	100000
0	37000	37000	249000	37000
36000	10000	102000	286000	102000
0	39000	39000	388000	39000
42000	10000	114000	427000	114000
0	59000	59000	541000	59000

Block	Label	Details	start_ms
0	Initial Pause		0
1	Stim 160 mA	20 Hz, 20%, 3×200 pulses, gap 38 s	10000
2	Rest (0 mA)	1 Hz, 50%	116000
3	Stim 195 mA	20 Hz, 20%, 3×200 pulses, gap 35 s	149000
4	Rest (0 mA)	1 Hz, 50%	249000
5	Stim 210 mA	20 Hz, 20%, 3×200 pulses, gap 36 s	286000
6	Rest (0 mA)	1 Hz, 50%	388000
7	Stim 220 mA	20 Hz, 20%, 3×200 pulses, gap 42 s	427000
8	Rest (0 mA)	1 Hz, 50%	541000

end_ms	duration_ms	start_time	end_time	duration_time
10000	10000	00:00:00.000	00:00:10.000	00:00:10.000
116000	106000	00:00:10.000	00:01:56.000	00:01:46.000
149000	33000	00:01:56.000	00:02:29.000	00:00:33.000
249000	100000	00:02:29.000	00:04:09.000	00:01:40.000
286000	37000	00:04:09.000	00:04:46.000	00:00:37.000
388000	102000	00:04:46.000	00:06:28.000	00:01:42.000
427000	39000	00:06:28.000	00:07:07.000	00:00:39.000
541000	114000	00:07:07.000	00:09:01.000	00:01:54.000
600000	59000	00:09:01.000	00:10:00.000	00:00:59.000

Supplementary Table 5. Example of titration optogenetic paradigm parameters. An example of parameters used in a titration optogenetic paradigm experiment.

VNPD	opto_pulse_no	start	stop	duration_in_s	pauses_pseudorandomized_33-42s	interpulse_pause_first-second-pulse-no
Optogenetics	1	00:00:10	00:00:20	10		
Stimulation	2	00:00:58	00:01:08	10	00:00:38	1_2
Paradigm	3	00:01:46	00:01:56	10	00:00:38	2_3
	4	00:02:29	00:02:39	10	00:00:33	3_4
	5	00:03:14	00:03:24	10	00:00:35	4_5
	6	00:03:59	00:04:09	10	00:00:35	5_6
	7	00:04:46	00:04:56	10	00:00:37	6_7
	8	00:05:32	00:05:42	10	00:00:36	7_8
	9	00:06:18	00:06:28	10	00:00:36	8_9
	10	00:07:07	00:07:17	10	00:00:39	9_10
	11	00:07:59	00:08:09	10	00:00:42	10_11
	12	00:08:51	00:09:01	10	00:00:42	11_12
final_off		00:09:01	00:10:00		00:00:59	

Supplementary Table 6. Perithreshold optogenetic paradigm overview. An example of parameters used in a perithreshold optogenetic paradigm experiment.

Data [#,##]	Figure	Main test		Post-hoc	
		Test	Significance	Test	Significance
mScarlet-positive VNC cells (week8)	Fig. 3b	Two-tailed unpaired t-test	EV-ChRmine (n = 10) vs. A53T-ChRmine (n = 12): p > 0.05		
Laser Power per VSS (A53T-ChRmine)	Fig. 3e	mixed-effects model	VSS***, TimePoint**, Interaction*	Two-tailed Tukey's multiple comparisons	Week4 vs. Week8 VSS1: p = 0.06, VSS2: p = 0.12, VSS3: p = 0.03, VSS4: p = 0.01
Laser Power per VSS (EV-ChRmine)	Fig. 3e	mixed-effects model	VSS***, TimePoint*, Interaction*	Two-tailed Tukey's multiple comparisons	Week4 vs. Week8 VSS1: p = 0.15, VSS2: p = 0.21, VSS3: p = 0.02, VSS4: p = 0.01
Naturalistic behaviour cluster occupancy	Fig. 3h	multinomial logistic regression	See Supplementary Table 4	FDR/Holm-adjusted Wald tests	EV-ChRmine vs. A53T-ChRmine: p = 0.148
Vestibular symptomatology cluster occupancy	Fig. 3h	multinomial logistic regression	See Supplementary Table 3	FDR/Holm-adjusted Wald tests	EV-ChRmine vs. A53T-ChRmine: p = 8.37e-5
Laser Power per VSS (Week4)	Fig. 4a	mixed-effects model	VSS****, Group^{ns}, Interaction*	Two-tailed Tukey's multiple comparisons	EV-ChRmine vs. A53T-ChRmine: VSS1: p = 0.84, VSS2: p = 0.30, VSS3: p = 0.04, VSS4: p = 0.13
Laser Power per VSS (Week8)	Fig. 4b	mixed-effects model	VSS****, Group*, Interaction**	Two-tailed Tukey's multiple comparisons	EV-ChRmine vs. A53T-ChRmine: VSS1: p = 0.69, VSS2: p = 0.26, VSS3: p = 0.002, VSS4: p < 0.0001
mScarlet-positive VNC cells in function of laser power per VSS (EV-ChRmine, Week4)	Fig. 4c	Spearman's r (n = 9)	VSS 1: r = -0.66, p = 0.03, VSS 2: r = -0.10, p = 0.40, VSS 3: r = 0.03, p = 0.47, VSS 4: r = -0.68, p = 0.03		
mScarlet-positive VNC cells in function of laser power per VSS (EV-ChRmine, Week8)	Fig. 4c	Spearman's r (n = 9)	VSS 1: r = -0.50, p = 0.08, VSS 2: r = -0.51, p = 0.08, VSS 3: r = -0.82, p = 0.005, VSS 4: r = -0.76, p = 0.01		
mScarlet-positive VNC cells in function of laser power per VSS (A53T-ChRmine, Week4)	Fig. 4d	Spearman's r (n = 11)	VSS 1: r = -0.32, p = 0.16, VSS 2: r = -0.52, p = 0.18, VSS 3: r = -0.29, p = 0.06, VSS 4: r = -0.51, p = 0.05		
mScarlet-positive VNC cells in function of laser power per VSS (A53T-ChRmine, Week8)	Fig. 4d	Spearman's r (n = 12)	VSS 1: r = -0.04, p = 0.45, VSS 2: r = -0.48, p = 0.06, VSS 3: r = -0.55, p = 0.03, VSS 4: r = -0.63, p = 0.02		
Laser power for perithreshold (week4)	Fig. 4f	one-tailed Welch's t-test	EV-ChRmine vs. A53T-ChRmine: t = 0.184, p = 0.428		
Laser power for perithreshold (week8)	Fig. 4g	one-tailed Welch's t-test	EV-ChRmine vs. A53T-ChRmine:		

			t = 2.157, *p* = 0.027		
Pf cFos+ cell count (ipsilateral)	Fig. 5f (top)	Kruskal-Wallis (Group: A53T-ChRmine vs. EV-ChRmine vs. mCherry-Sham)	*H*-statistic: 10.71, *p* = 0.005	Two-tailed Dunn's multiple comparisons test	A53T vs. EV: *p* = 0.86 A53T vs. mCherry: *p* = 0.0668 EV vs. mCherry: *p* = 0.0076
Pf cFos+ cell count (contralateral)	Fig. 5f (bottom)	Kruskal-Wallis (Group: A53T-ChRmine vs. EV-ChRmine vs mCherry-Sham)	*H*-statistic: 8.72, *p* = 0.013	Two-tailed Dunn's multiple comparisons test	A53T vs. EV: *p* = 0.80 A53T vs. mCherry: *p* = 0.12 EV vs. mCherry: *p* = 0.0879
VNC cFos+ cell count (ipsilateral)	Fig. 5g (top)	Kruskal-Wallis (Group: A53T-ChRmine vs. EV-ChRmine vs. mCherry-Sham)	*H*-statistic: 14.56, *p* = 0.0007	Two-tailed Dunn's multiple comparisons test	A53T vs. EV: *p* = 0.75 A53T vs. mCherry: *p* = 0.0365 EV vs. mCherry: *p* = 0.0160
VNC cFos+ cell count (contralateral)	Fig. 5g (bottom)	Kruskal-Wallis (Group: A53T-ChRmine vs. EV-ChRmine vs. mCherry-Sham)	*H*-statistic: 10.71, *p* = 0.005	Two-tailed Dunn's multiple comparisons test	A53T vs. EV: *p* = 0.81 A53T vs. mCherry: *p* = 0.0477 EV vs. mCherry: *p* = 0.0420
ZI cFos+ cell count (ipsilateral)	Fig. 5h(top)	Kruskal-Wallis (Group: A53T-ChRmine vs. EV-ChRmine vs. mCherry-Sham)	*H*-statistic: 13.70, *p* = 0.001	Two-tailed Dunn's multiple comparisons test	A53T vs. EV: *p* = 0.60 A53T vs. mCherry: *p* = 0.15 EV vs. mCherry: *p* = 0.0559
ZI cFos+ cell count (contralateral)	Fig. 5h (bottom)	Kruskal-Wallis (Group: A53T-ChRmine vs. EV-ChRmine vs. mCherry-Sham)	*H*-statistic: 14.09, *p* = 0.0008	Two-tailed Dunn's multiple comparisons test	A53T vs. EV: *p* = 0.97 A53T vs. mCherry: *p* = 0.15 EV vs. mCherry: *p* = 0.0934
Gi cFos+ cell count (ipsilateral)	Fig. 5i (top)	Kruskal-Wallis (Group: A53T-ChRmine vs. EV-ChRmine vs. mCherry-Sham)	*H*-statistic: 15.69, *p* = 0.0004	Dunn's multiple comparisons test	A53T vs. EV: *p* = 0.60 A53T vs. mCherry: *p* = 0.0071 EV vs. mCherry: *p* = 0.0160
Gi cFos+ cell count (contralateral)	Fig. 5i (bottom)	Kruskal-Wallis (Group: A53T-ChRmine vs. EV-ChRmine vs. mCherry-Sham)	*H*-statistic: 14.30, *p* = 0.0008	Dunn's multiple comparisons test	A53T vs. EV: *p* = 0.65 A53T vs. mCherry: *p* = 0.0477 EV vs. mCherry: *p* = 0.0312
Motion measure during locomotion (A53T-ChRmine)	Fig. 6c (left)	Lognormal RM one-way ANOVA	*F* = 13.08, *p* < 0.0001	Two-tailed Holm-Šídák's multiple comparisons test	Adjusted P Values - W1 vs. W8-OFF: *p* = 0.0003 W8-OFF vs. PT: *p* = 0.0415

					W8-OFF vs. TITR: p = 0.0766
Motion measure during locomotion (EV-ChRmine)	Fig. 6c (middle)	Friedman test	Friedman statistic = 22.68, $p < 0.0001$	Two-tailed Dunn's multiple comparisons test	Adjusted P Values - W1 vs. W8-OFF: p = 0.0016 W8-OFF vs. PT: p = 0.0281 W8-OFF vs. TITR: p = 0.0055
Motion measure during locomotion (mCherry-Sham)	Fig. 6c (right)	RM one-way ANOVA	F = 11.07, p = 0.0231	Two-tailed Šídák's multiple comparisons test	Adjusted P Values - W1 vs. W8-OFF: p = 0.0140, W8-OFF vs. sham PT: p = 0.7740
Distance moved (A53T-ChRmine)	Fig. 6d (left)	mixed-effects model	Fixed effect (type III): $p < 0.0001$	Two-tailed Holm-Šídák's multiple comparisons test	Adjusted P Values - W1 vs. W8-OFF: p = 0.0006 W8-OFF vs. PT: p = 0.6016 ON vs. TITR: p = 0.0010
Distance moved (EV-ChRmine)	Fig. 6d (middle)	RM one-way ANOVA	F = 9.550, p = 0.0076	Two-tailed Šídák's multiple comparisons test	Adjusted P Values - W1 vs. W8-OFF: p = 0.0441 W8-OFF vs. PT: p = 0.0413 ON vs. TITR: p = 0.0028
Distance moved (mCherry-Sham)	Fig. 6d (right)	Friedman test	Friedman statistic = 7.600, p = 0.0239	Two-tailed Dunn's multiple comparisons test	Adjusted P Values - W1 vs. W8-OFF: p = 0.0228, W8-OFF vs. sham PT: $p > 0.9999$
A53T-ChRmine syllable transition	Fig. 6g (top)	See Supplementary Table 1	W1 vs. OFF	See Supplementary Table 1	See Supplementary Table 1
A53T-ChRmine syllable transition	Fig. 6g (bottom)	See Supplementary Table 1	W8-OFF vs. PT	See Supplementary Table 1	See Supplementary Table 1
EV-ChRmine syllable transition	Fig. 6g (top)	See Supplementary Table 1	W1 vs. OFF	See Supplementary Table 1	See Supplementary Table 1
EV-ChRmine syllable transition	Fig. 6g (bottom)	See Supplementary Table 1	W8-OFF vs. PT	See Supplementary Table 1	See Supplementary Table 1
mCherry-Sham syllable transition	Fig. 6g (top)	See Supplementary Table 1	W1 vs. OFF	See Supplementary Table 1	See Supplementary Table 1
mCherry-Sham syllable transition	Fig. 6g (bottom)	See Supplementary Table 1	W8-OFF vs. PT	See Supplementary Table 1	See Supplementary Table 1
Average Vmax	Fig. 7b	Two-way RM ANOVA	time/stim*, groups**, interaction^{ns}	Šídák's multiple comparisons test	W8-Perithreshol

					d*, W8-Off**, Week1^{ns}
Proportion of failed trial (A53T-ChRmine)	Fig. 7c	Two-sided Fisher’s exact test)	FGI1 vs. FGI2+3****		
Proportion of failed trial (EV-ChRmine)	Fig. 7d	Two-sided Fisher’s exact test)	FGI1 vs. FGI2+3****		
Power-Periodogram (A53T-ChRmine)-10-25 cm/s	Fig. 7g	mixed-effects model	time/stim^{ns}, groups^{ns}, interaction^{ns}	Two-tailed Šídák's multiple comparisons tes t	Week1 vs. week 8 OFF: $p > 0.05$ Week1 vs. week 8 PT: $p > 0.05$
Power-Periodogram (EV-ChRmine)-10-25 cm/s	Fig. 7h	mixed-effects model	time/stim^{ns}, groups^{ns}, interaction^{ns}	Two-tailed Šídák's multiple comparisons tes t	Week1 vs. week 8 OFF: $p > 0.05$ Week1 vs. week 8 PT: $p > 0.05$
Power-Periodogram (A53T-ChRmine)-10 cm/s	Fig. 7i	mixed-effects model	time/stim^{ns}, groups^{ns}, interaction^{ns}	Two-tailed Šídák's multiple comparisons tes t	Week1 vs. week 8 OFF: $p > 0.05$ Week1 vs. week 8 PT: $p > 0.05$
Power-Periodogram (A53T-ChRmine)-15 cm/s	Fig. 7j	mixed-effects model	time/stim^{ns}, groups^{ns}, interaction^{ns}	Two-tailed Šídák's multiple comparisons tes t	Week1 vs. week 8 OFF: $p > 0.05$ Week1 vs. week 8 PT: $p > 0.05$
Power-Periodogram (A53T-ChRmine)-20 cm/s	Fig. 7k	mixed-effects model	time/stim^{ns}, groups^{ns}, interaction^{ns}	Two-tailed Šídák's multiple comparisons tes t	Week1 vs. week 8 OFF: $p < 0.05$ Week1 vs. week 8 PT: $p > 0.05$
Power-Periodogram (A53T-ChRmine)-25 cm/s	Fig. 7l	mixed-effects model	time/stim^{ns}, groups^{ns}, interaction^{ns}	Two-tailed Šídák's multiple comparisons tes t	Week1 vs. week 8 OFF: $p > 0.05$ Week1 vs. week 8 PT: $p > 0.05$
Autocorrelation (EV-ChRmine vs. A53T-ChRmine)-20 cm/s-Week8-Lateral elbow-knee pair- OFF	Fig. 7m	mixed-effects model	time/stim^{ns}, groups^{ns}, interaction^{ns}	Two-tailed Šídák's multiple comparisons tes t	Week1 vs. week 8 OFF: $p > 0.05$ Week1 vs. week 8 PT: $p > 0.05$
Autocorrelation (EV-ChRmine vs. A53T-ChRmine)-20 cm/s-Week8-Lateral elbow-knee pair- PT	Fig. 7n	mixed-effects model	time/stim^{ns}, groups^{ns}, interaction^{ns}	Two-tailed Šídák's multiple comparisons tes t	Week1 vs. week 8 OFF: $p >$ 0.05Week1 vs. week 8 PT: $p > 0.05$
Autocorrelation (EV-ChRmine vs. A53T-ChRmine)-20 cm/s-Week8-Lateral calcaneus pair- OFF	Fig. 7o	mixed-effects model	time/stim^{ns}, groups^{ns}, interaction^{ns}	Two-tailed Šídák's multiple comparisons tes t	Week1 vs. week 8 OFF: $p > 0.05$ Week1 vs. week 8 PT: $p > 0.05$
Autocorrelation (EV-ChRmine vs. A53T-ChRmine)-20 cm/s-Week8-Lateral calcaneus pair- PT	Fig. 7p	mixed-effects model	time/stim^{ns}, groups^{ns}, interaction^{ns}	Two-tailed Šídák's multiple comparisons tes t	Week1 vs. week 8 OFF: $p > 0.05$ Week1 vs. week 8 PT: $p > 0.05$
Autocorrelation (EV-ChRmine vs. A53T-ChRmine)-20 cm/s-Week8-Lateral handmid pair- OFF	Fig. 7q	mixed-effects model	time/stim^{ns}, groups^{ns}, interaction^{ns}	Two-tailed Šídák's multiple comparisons tes t	Week1 vs. week 8 OFF: $p > 0.05$ Week1 vs. week 8 PT: $p > 0.05$

Autocorrelation (EV-ChRmine vs. A53T-ChRmine)-20 cm/s-Week8-Lateral handmaid pair- PT	Fig. 7r	mixed-effects model	time/stim^{ns}, groups^{ns}, interaction^{ns}	Two-tailed Šídák's multiple comparisons tes t	Week1 vs. week 8 OFF: *p* > 0.05 Week1 vs. week 8 PT: *p* > 0.05
Peak amplitude – peak frequency correlations	Fig. 7s,u	F-tests EV-ChRmine-W8-OFF vs. A53T-ChRmine-W8-OFF	Distal left limb (10-25 cm/s)*	One-sided F-test, $F(1,66)$ = 5.322, *p* = 0.024	
Peak amplitude – peak frequency correlations	Fig. 7s,u	F-tests EV- ChRmine-W8-Perithreshold vs. A53T-ChRmine- W8-Perithreshold	Distal left limb (10-25 cm/s)^{ns}	One-sided F-test, $F(1,63)$ = 1.113, *p* = 0.296	
Optical density	Supplementary Fig. 6a	Two-way RM ANOVA	time point^{ns}, group****, interaction^{ns}	Two-tailed Šídák's multiple comparisons test	Week4 & week8: *p* < 0.0001
TH-positive neurons count	Supplementary Fig. 6c	Two-way RM ANOVA	time point^{ns}, group****, interaction^{ns}	Two-tailed Šídák's multiple comparisons test	Week4 & week8: *p* < 0.0001
Optical density vs. TH-positive neurons count correlation	Supplementary Fig. 6d	Spearman's r	Week4: *r* = 0.82, *p* < 0.001 Week8: *r* = 0.83, *p* < 0.001		
TH-positive neurons count correlation vs. Nissl-based cell count correlation	Supplementary Fig. 6e	Spearman's r	Week4: *r* = 0.94, *p* < 0.0001 Week8: *r* = 0.93, *p* < 0.0001		
Head rotation during locomotion	Supplementary Fig. 6f	Two-way RM ANOVA	week^{ns}, group**, interaction*	Two-tailed Šídák's multiple comparisons test	Week4: *p* = 0.0013, week8: *p* > 0.05
Symmetry of head rotation during locomotion	Supplementary Fig. 6f	Two-way RM ANOVA	week^{ns}, group****, interaction^{ns}	Two-tailed Šídák's multiple comparisons test	Week4: *p* = 0.0106, week8: *p* = 0.0019
TH-positive neurons in the SNC	Supplementary Fig. 7d	two-tailed unpaired t-test	*t* = 12.74, *p* < 0.0001		
TH-positive neurons count correlation vs Nissl-based cell count correlation	Supplementary Fig. 7e	Spearman's r	*r* = 0.8453, *p* < 0.0001		
D-amphetamine-induced rotations	Supplementary Fig. 7f	Simple logistic regression model	AUROC=0.9375, s.e.=0.05201, *p* = 0.0005		
Forepaw use symmetry	Supplementary Fig. 7i	mixed-effects model	time/stimulation^{ns}, group**, interaction^{ns}	Two-tailed Tukey's multiple comparisons	Week8-off: adjusted *p* = 0.0097
Rotatory symmetry	Supplementary Fig. 7j	mixed-effects model	time/stimulation****, group****, interaction****	Two-tailed Tukey's multiple comparisons	Week8-off: adjusted *p* < 0.0001
Mean Vmax vs. TH-positive neurons count	Supplementary Fig. 7k	Spearman's r	*r* = 0.6631, *p* = 0.0008		
Mean Vmax vs. rotatory symmetry	Supplementary Fig. 7l	Spearman's r	*r* = -0.6045, *p* = 0.0029		
Motion measure	Supplementary Fig. 8c	one-way ANOVA	*F* = 0.6404, *p* > 0.05	Two-tailed Dunnett's multiple comparisons tes t	All *p* > 0.05
Naturalistic behaviour cluster occupancy	Supplementary Fig. 11b	multinomial logistic regression	See Supplementary Table 4	FDR/Holm-adjusted Wald tests	See Supplementary Table 4
Vestibular symptomatology cluster occupancy	Supplementary Fig. 11d	multinomial logistic regression	See Supplementary Table 3	FDR/Holm-adjusted Wald tests	See Supplementary Table 3

VNC cFos expression	Supplementary Fig. 12d	one-way ANOVA	*F* = 5.230, $p < 0.05$	Two-tailed Dunnett's multiple comparisons test	Adjusted P Values: ShamPT vs. PT: $p = 0.0107$, ShamPT vs. VSS2: $p = 0.2145$ ShamPT vs. VSS4: $p = 0.0386$
Peak amplitude – peak frequency correlations	Supplementary Fig. 14s	F-tests EV-ChRmine-W8-OFF vs. A53T-ChRmine-W8-OFF	Distal left limb (20 cm/s)^{ns}	One-sided F-test, $F(1,15) = 0.405$, $p = 0.534$	
Peak amplitude – peak frequency correlations	Supplementary Fig. 14t	F-tests EV-ChRmine-W8-Perithreshold vs. A53T-ChRmine-W8-Perithreshold	Distal left limb (20 cm/s)^{ns}	One-sided F-test, $F(1,13) = 1.513$, $p = 0.24$	

[#]for statistical tests and corresponding results for Fig. 4 and Supplementary Fig. 11 - see Supplementary Table 3 + 4; [##]For statistical tests and their results for Fig. 6 - see Supplementary Table 1

Supplementary Table 7. Statistical testing and results. Outline of primary statistical tests and post-hoc results.

syllable	descriptor	frequency_all_selected_conditions	A53T_Baseline_OFF	A53T_Week8_OFF	A53T_Week8_PT
0	compact straight stance	0,066615	0,051972	0,076283	0,071174
1	compact leftward curl	0,090087	0,073854	0,102893	0,116339
2	low straight glide	0,048713	0,041332	0,046188	0,048738
3	elongated head-forward stretch	0,031472	0,035072	0,036521	0,030894
4	compact slight-turn stance	0,032055	0,024498	0,029178	0,025114
5	compact right-curled dip	0,031013	0,025545	0,032691	0,029777
6	straight midline glide	0,026869	0,021574	0,029773	0,022391
7	broad straight extension	0,022719	0,020389	0,022663	0,018028
8	tight straight pause	0,020644	0,017546	0,023769	0,024247
9	compact side bend	0,021050	0,019265	0,027371	0,019323
10	elongated straight track	0,019678	0,016905	0,023708	0,022052
11	elongated slight-tail curve	0,019214	0,019101	0,026760	0,020668
12	straight head-forward push	0,018182	0,019619	0,012507	0,010915
13	sharp leftward bend	0,018906	0,021584	0,011312	0,012421
14	long straight forward push	0,017248	0,018224	0,026204	0,020457
15	compact straight shuffle	0,015172	0,015817	0,012796	0,012054
16	elongated head sweep	0,015870	0,019968	0,014353	0,010426
17	long forward stretch	0,023134	0,028392	0,032613	0,023686
18	tight left bend	0,018562	0,018060	0,016432	0,015288
19	broad rightward curve	0,049497	0,054851	0,064120	0,048016
20	long straight forward step	0,033389	0,015520	0,032608	0,065200
21	elongated straight run	0,015709	0,018106	0,022091	0,018840
22	very long stretched run	0,021024	0,038382	0,022624	0,015488
23	narrow straight nose-forward	0,010733	0,012329	0,011001	0,010548
24	curved rightward arc	0,018688	0,020789	0,016649	0,021224
25	diagonal left sweep	0,013092	0,016392	0,013825	0,013588
26	pronounced right C-turn	0,011206	0,016874	0,013041	0,014021
27	maximal full-body extension	0,015295	0,017326	0,017671	0,015488
28	long low straight extension	0,009147	0,012375	0,006192	0,005302
29	elongated slight-right bend	0,010013	0,016084	0,009139	0,007275
30	broad straight advance	0,014844	0,012077	0,015059	0,020751
31	pronounced S-turn pivot	0,009019	0,014786	0,007049	0,008770
32	long straight tail sweep	0,013324	0,012975	0,013864	0,009064
33	strong right-bend arc	0,012150	0,024842	0,009533	0,011054
34	compact right curl	0,007806	0,007424	0,001468	0,001584
35	curved leftward head sweep	0,013495	0,024786	0,011340	0,009253
36	compact straight pause	0,006333	0,008111	0,006276	0,010737
37	long extended forward rush	0,009460	0,010333	0,010545	0,011515
38	tight crouched curl	0,015176	0,014094	0,014642	0,019707
39	compact C-turn	0,006802	0,004140	0,001745	0,001023
40	compact rounded crouch	0,002827	0,004643	0,002246	0,002656
41	tight straight low shuffle	0,008989	0,005146	0,001907	0,001595
42	compact straight forward	0,008813	0,001437	0,000356	0,000339
43	compact nose-forward push	0,010113	0,001226	0,000022	0,000106
44	deep leftward C-turn	0,005065	0,008306	0,003758	0,002751
45	long straight slide	0,004083	0,007506	0,003096	0,003618
46	pronounced rightward C-turn	0,005559	0,010205	0,004269	0,003740
47	sharp right turn pivot	0,003988	0,003617	0,006381	0,008553
48	deep leftward stretch-turn	0,003105	0,005982	0,001840	0,001645

syllable	descriptor	EV_Baseline_OFF	EV_Week8_OFF	EV_Week8_PT	mCherry_Baseline_OFF
0	compact straight stance	0,050253	0,071170	0,070776	0,055080
1	compact leftward curl	0,082128	0,094985	0,103747	0,040360
2	low straight glide	0,037461	0,042373	0,059359	0,050307
3	elongated head-forward stretch	0,028232	0,030792	0,031810	0,026360
4	compact slight-turn stance	0,031475	0,034668	0,034098	0,035680
5	compact right-curled dip	0,028479	0,033654	0,031950	0,025200
6	straight midline glide	0,025670	0,031112	0,024601	0,033867
7	broad straight extension	0,020892	0,030138	0,020653	0,023000
8	tight straight pause	0,018750	0,020059	0,018506	0,020747
9	compact side bend	0,016835	0,023828	0,020680	0,019187
10	elongated straight track	0,013432	0,021820	0,017439	0,021773
11	elongated slight-tail curve	0,017823	0,018618	0,014145	0,023907
12	straight head-forward push	0,015154	0,021033	0,019886	0,027627
13	sharp leftward bend	0,016335	0,013835	0,017139	0,033093
14	long straight forward push	0,017282	0,018551	0,012818	0,011973
15	compact straight shuffle	0,016288	0,013008	0,017299	0,018747
16	elongated head sweep	0,019458	0,019185	0,013925	0,019427
17	long forward stretch	0,028720	0,024949	0,023754	0,002333
18	tight left bend	0,015634	0,017384	0,014471	0,032840
19	broad rightward curve	0,073948	0,064019	0,020147	0,015733
20	long straight forward step	0,016415	0,031393	0,061687	0,009773
21	elongated straight run	0,018704	0,017164	0,019133	0,000493
22	very long stretched run	0,038142	0,019685	0,017732	0,000107
23	narrow straight nose-forward	0,014066	0,010533	0,011470	0,007360
24	curved rightward arc	0,025870	0,024875	0,022534	0,004640
25	diagonal left sweep	0,014874	0,013061	0,012151	0,007227
26	pronounced right C-turn	0,015321	0,010046	0,010397	0,001760
27	maximal full-body extension	0,018003	0,020826	0,023441	0,000040
28	long low straight extension	0,011711	0,009192	0,011110	0,012120
29	elongated slight-right bend	0,013125	0,011680	0,013664	0,002240
30	broad straight advance	0,013639	0,015389	0,017559	0,009040
31	pronounced S-turn pivot	0,012678	0,010220	0,012624	0,000000
32	long straight tail sweep	0,009355	0,016724	0,017379	0,013893
33	strong right-bend arc	0,017189	0,011654	0,013604	0,000000
34	compact right curl	0,005412	0,003082	0,002287	0,038547
35	curved leftward head sweep	0,024129	0,012368	0,016665	0,000107
36	compact straight pause	0,007934	0,004423	0,005875	0,002627
37	long extended forward rush	0,012238	0,014682	0,010377	0,000000
38	tight crouched curl	0,013713	0,016010	0,023221	0,005840
39	compact C-turn	0,003703	0,001294	0,001394	0,039227
40	compact rounded crouch	0,003890	0,003616	0,002854	0,000280
41	tight straight low shuffle	0,004497	0,001854	0,001374	0,043360
42	compact straight forward	0,002202	0,000607	0,000373	0,062053
43	compact nose-forward push	0,002335	0,000107	0,000173	0,067640
44	deep leftward C-turn	0,007020	0,007318	0,006435	0,001920
45	long straight slide	0,006252	0,002962	0,004975	0,001053
46	pronounced rightward C-turn	0,012598	0,004983	0,004768	0,000360
47	sharp right turn pivot	0,003156	0,002522	0,003454	0,001253
48	deep leftward stretch-turn	0,007941	0,001961	0,003054	0,000480

syllable	descriptor	mCherry_Week8_OFF	mCherry_Week8_PT	mean_duration_from_stats_df
0	compact straight stance	0,080270	0,083726	0,471576
1	compact leftward curl	0,072289	0,084874	0,747780
2	low straight glide	0,067699	0,067218	0,456737
3	elongated head-forward stretch	0,027864	0,027264	0,351884
4	compact slight-turn stance	0,046894	0,048656	0,380455
5	compact right-curled dip	0,038647	0,040275	0,384853
6	straight midline glide	0,029839	0,032895	0,359335
7	broad straight extension	0,027677	0,027877	0,358446
8	tight straight pause	0,021619	0,020698	0,339961
9	compact side bend	0,020791	0,020404	0,351508
10	elongated straight track	0,019817	0,021966	0,346156
11	elongated slight-tail curve	0,013945	0,012598	0,337583
12	straight head-forward push	0,025249	0,025942	0,332758
13	sharp leftward bend	0,035778	0,033496	0,354050
14	long straight forward push	0,007927	0,006299	0,323827
15	compact straight shuffle	0,018483	0,017629	0,294554
16	elongated head sweep	0,014199	0,010129	0,310021
17	long forward stretch	0,006859	0,006432	0,458886
18	tight left bend	0,029479	0,024034	0,372025
19	broad rightward curve	0,032882	0,035204	1,140686
20	long straight forward step	0,019097	0,024595	0,705397
21	elongated straight run	0,001161	0,000667	0,394733
22	very long stretched run	0,001695	0,000627	0,584077
23	narrow straight nose-forward	0,005218	0,007527	0,308473
24	curved rightward arc	0,005912	0,004431	0,569170
25	diagonal left sweep	0,010102	0,008808	0,409762
26	pronounced right C-turn	0,000827	0,000841	0,333342
27	maximal full-body extension	0,000827	0,000801	0,464031
28	long low straight extension	0,007099	0,007006	0,312608
29	elongated slight-right bend	0,002149	0,001668	0,336738
30	broad straight advance	0,010276	0,013598	0,573171
31	pronounced S-turn pivot	0,000200	0,000254	0,357748
32	long straight tail sweep	0,014666	0,014279	0,554717
33	strong right-bend arc	0,000067	0,000307	0,538375
34	compact right curl	0,023340	0,017936	0,340191
35	curved leftward head sweep	0,000587	0,000440	0,632758
36	compact straight pause	0,001815	0,001041	0,299879
37	long extended forward rush	0,000427	0,000307	0,471811
38	tight crouched curl	0,010769	0,007313	0,774916
39	compact C-turn	0,022553	0,019577	0,332226
40	compact rounded crouch	0,001294	0,000227	0,145497
41	tight straight low shuffle	0,035671	0,031160	0,496372
42	compact straight forward	0,033349	0,037352	0,413293
43	compact nose-forward push	0,044519	0,044959	0,540467
44	deep leftward C-turn	0,001428	0,000961	0,329995
45	long straight slide	0,001081	0,000827	0,285331
46	pronounced rightward C-turn	0,000254	0,000107	0,377357
47	sharp right turn pivot	0,000294	0,000347	0,294686
48	deep leftward stretch-turn	0,000214	0,000400	0,258549

Supplementary Table 8. Syllable index, descriptor and frequencies. Systematic presentation of syllable index with frequency and a descriptor. For each syllable, the table lists the assigned behavioural descriptor, overall frequency across all selected conditions, condition-specific usage frequencies for A53T-ChRmine, EV-ChRmine, and sham mCherry mice at baseline, week 8 off stimulation, and week 8 with perithreshold stimulation, as applicable. Syllables are ordered by decreasing global frequency, and frequencies represent state-occupancy proportions. Mean syllable duration was derived from the KPMS statistics output. Red indicates syllables with global frequency < 0.005.
