# Supplementary material for: Vestibular nucleus stimulation for ameliorating locomotor dynamics in a Parkinsonian mouse model
Source: Nat Commun. 2026 Aug 4;17:7772. doi: 10.1038/s41467-026-76183-2 (PMC13439372; doi:10.1038/s41467-026-76183-2)
Supplement: Supplementary file 2 [file 41467_2026_76183_MOESM2_ESM.pdf]

nature portfolio

Peer Review File

Vestibular nucleus stimulation for ameliorating locomotor dynamics in a parkinsonian mouse model

Corresponding Author: Professor Chi Wang Ip

This file contains all reviewer reports in order by version, followed by all author rebuttals in order by version.

Version 0:

Reviewer comments:

Reviewer #1

(Remarks to the Author)
General opinion
The manuscript (MS) addresses an innovative and clinically relevant topic, utilising an optogenetic tool in combination with advanced AI-assisted behavioural analyses to investigate the therapeutic potential of vestibular modulation in a Parkinson's disease (PD) mouse model. The experimental design and employed techniques are rigorous and sophisticated, with significant potential implications for understanding the role of vestibular circuitry in posture and gait dysfunction associated with PD.
However, several methodological and interpretational issues need clarification and improvement. Key concerns include insufficient clarify on the experimental timelines and inconsistent data pooling across different time points and groups, ambiguous or incomplete methodological descriptions, and unclear or potentially misleading data presentation. Addressing these issues would substantially enhance the MS's clarity, reproducibility, and overall scientific rigor.
My detailed comments are provided below for further clarification and improvement. Given the extensive nature of our comments, we have structured them sequentially, aligned with the manuscript's organisation. Among these comments, the key concerns we consider particularly important include comments numbered 3, 8-12 (major).

1. ChRmine
1.1. In line 89-90, ChRmine is first introduced as an optogenetics. Given the wide range of available optogenetic tools, please consider providing a brief description of ChRmine for readers less familiar with the tool. For example, you might state: 'we utilised an optogenetic tool, ChRmine, which is a red-shifted, cation-conducting channelrhodopsin, that excites neurons upon red-light stimulation', drawing from additional context provided in lines 718–719.
1.2. The MS does not provide sufficient detail regarding the light conditions, particularly the light powers, or the verification of neuronal activation in their experiments (more discussions later).
1.3. It appears that the original publication for ChRmine (PMID: 31320556) is not referenced. Please include this reference to allow readers to verify the properties of this tool in the original paper.
1.4. The MS mentions the construct 'AAV2/5-hSyn-DIO-ChRmine-mSccarlet' in lines 719-720 but does not provide the Addgene ID or cloning details. Please include the relevant Addgene ID(s) and briefly describe any subcloning steps (for example, if you subcloned the DIO construct using Addgene plasimid #130994) to ensure reproducibility.

2. A53T PD mouse model (part 1)
2.1. Model generation details
: the MS does not provide sufficient information regarding the generation of the A53T overexpression PD mouse model. Based on the authors' previous publications (PMID: 28143577; PMID: 21029459), it appears that the AAV1/2-A53T construct likely uses the CAG promoter. For viral-based overexpression models, different promoters can have significant impacts on the expression levels and patterns. Thus, please clearly state the full format of viral construct (e.g. AAV1/2-CAG-A53T) in the MS.
2.2. Data organisation and presentation
: The MS introduces the A53T and EV groups early (lines 91-97), but clear validation data for the model are not presented until Fig. 3. I suggest considering the re-organisation of some data so that the model validation is clearly delineated before proceeding to subsequent experiments in the study.
2.3. Group descriptions
: there are only a few descriptions of the A53T group in certain sections (e.g. lines 100, 110-112), with limited supporting

data. Consider separating the A53T data from Fig.1, which focuses on optogenetic titration and vestibular symptomatology (VS), or clearly provide or indicate the relevant data for this group.

3. Titration of optomodulation and VVS scaling
3.1. Data presentation and grouping
: The results describing titration of optomoudlation and the scaling of VS are intriguing, as 4 distinct VSs are observed with increasing laser power thresholds. However, the data presentation is confusing and potentially misleading. Data appear to be mixed from different experimental groups (VN-ChR [VN-ChR-SNc-EV], PD [VN-ChR-SNc-A53T], and optical control [VN-mCh]) and time points without clear separation or statistical justification. I recommend that the authors first present the results for the VN-ChR group separately (e.g., Fig 1. a-f or a-j) and then provide separate or statistically compared data for the PD group (e.g. Fig. 1g-k or k). This separation will clarify the origin of each dataset and the interpretation of the results.
3.2. Light conditions and thresholds
: The MS does not clearly describe the light conditions used in the titration experiments. Specifically, it is unclear what the exact light intensities are for each VVS to appear (i.e. the threshold values). It seems necessary to revise Fig. 1c,d (clarify whether the y-axis represents laser power and whether two stimulation cycles are applied during the 10-minute total duration. Additionally, please specify if the different VSs correspond to the first observation of the VVS at distinct light intensities or if these are the most prominent behaviours at those intensities). I suggest including a table that lists the light intensities at which each distinct VVS first appears, along with the mean +/- SEM (if differ for each mouse) and the probability (or frequency) of each VVS observed across the range of intensities (would be 4 in total light powers, using EV group only). If these experiments were performed across the entire optogenetic cohort (both EV and A53T), please state for each group separately and statistically compare the groups (not just stating in lines 110-112) at fixed time point (i.e. 8-wk).
3.3. Ordering and reference to prior studies
: The current ordering of panels is somewhat confusing – for example, Fig. 1c appears before the authors define what the VVSs are. It may be more logical to first present the reference data from a previous study (e.g. PMID: 34171443), which demonstrates that optogenetic activations (ChR) of Vglu2+ VN neurons induces vestibular disfunctions, followed by the pilot titration results and the aforementioned table. This would help contextualise the observed VSSs and experimental conditions.
3.4. Interpulsed light protocol
: The authors used the interpulsed periods of 30-40 s during titration experiments; however, it is unclear whether these intervals are sufficient for the vestibular system to fully return to its baseline (pre-stimulated) state. Although these intervals might reset ChRmine photocycle and the membrane potential, the recovery of the resting excitability balance of the ipsilateral and contralateral VN neurons may require a longer period. In a comparable study (PMID: 34171443), transient unilateral optogenetic stimulation (20 Hz, 5 ms for 30 s) of Vglu2+ VN neurons induced posture-locomotor deficits that persisted for up to 5 min pose-stimulation (some minutes for the VVS2). This raises the possibility that the short intervals in the current study might lead to cumulative effects, where residual activation influences the subsequent responses. It is unclear whether the different VVSs result solely from incremental light powers, repetitive stimulation or accumulation (as discussed in lines 149-150), the total duration or mixed effects (e.g. the observation of the VVS3 after VSS1 could have resulted from the prolonged stimulation, as discussed in lines 479-480). Thus, I suggest performing the 4 titrated light conditions (mean power for each VVS threshold) from their pilot experiments as separate groups (for each group using a constant light power at 20 Hz, 10 ms pulse duration at 30-40 s intervals for 10 min) to confirm that the light intensity alone is responsible for eliciting distinct VSS thresholds.
3.5. Mechanistical considerations
: it remains unclear whether the graded VVS responses result from an increased number of Vglut2+ VN neurons being activated, increased spike probabilities, or both. Additional experiments – such as quantifying cFos expression at each light intensity (compared to the VN-mCh control group) or electrophysiology recordings – would help elucidate the underlying mechanisms.

4. Semi-supervised ML and classifier verification
4.1. Training vs prediction data
: The MS employs a semi-supervised ML approach with UMAP analysis, using 4-wk data for training and applying the trained classifier to predict behaviour at 8-wk. could the authors clarify the rationale behind using 4-wk data for training, particularly given that the 4-wk time point appears suddenly in the text? A clear explanation is needed regarding why this time point was chosen for training.
4.2. Source of training data
: In Sup. Fig.2, the legend does not specify which group(s) of mice contributed the training data (and the number of mice involved). Additionally, it is unclear whether the data from mice used in training were excluded from the 8-wk prediction analysis. Please clarify the origin and separation of training vs prediction datasets to ensure transparency and to rule out potential bias and overfitting.
4.3. UMAP visualisation
: although the statistical analysis (in the legend) indicate that the clusters are significantly different, the UMAP visualisation in Fig. 1e does not clearly display distinct clusters. I suggest exploring alternative dimensional reduction methods and adjusting the colour schemes to improve the visual distinctiveness of the clusters.
4.4. Prediction accuracy across groups
: Were there any differences in prediction accuracy between the A53T and EV groups? This information is critical to assess whether the classifier performs equally well across different experimental conditions as abnormal mice often are not well classified.
4.5. Presentation of classification results
: For Sup. Fig. 2d, the current format is not very readable. I recommend presenting the data in a table that details the percentages of human vs machine classifications for each VSS, separately for the EV and A53T groups.

5. Fig. 1g-k, are the VVS defined by human or predicted? It is confusing because in the previous panel they just verified the ML method for the prediction of VVSs. In logical thinking, one might expect they had done so to use the ML predictions for the later studies (Fig. 1g-k). Please clarify the origin of these labels to unsure methodological transparency.

6. Clarification on VVS labelling
6.1. The MS does not clearly indicate whether the VVS labels shown in Fig. 1g-k were defined by human raters or generated by the ML predictions. In the previous panel, the ML method was validated for predicting VVSs, so it is not immediately clear why the subsequent data do not explicitly state the origin of these labels. Please clarify whether the labels used in Fig. 1g-k are based on human or machine.

7. Fig. 1k – Longitudinal data and interpretation
7.1. Time points and longitudinal design
: the data is described as 'longitudinal,' yet only two time points are shown: the baseline (1-wk after surgery) and 8-wk. For a robust longitudinal study, I recommend including at least one intermediate time point (e.g. 4-wk), as that data at this interval are available. Given that A53T model mice display complex behavioural changes as PD progresses, which is not always unidirectional or has a time threshold for a certain parameter to change, reflecting the dynamics of the pathological changes. Thus, additional time points would better capture these dynamic progressions of the pathology.
7.2. Interpretation of the data
: The MS lacks a clear interpretation of the longitudinal findings. For example, is the observed decreased sensitivity in the PD group (noting that the A53T and EV groups are currently mixed) attributable to alterations in PD circuitry, such as increased glutamatergic receptor expression in VN neurons (PMID: 36347898)? A brief interpretation of the result would enhance the study's impact.
7.3. Baseline consideration
: The term 'baseline' is used to describe data from 1-wk post-surgery. However, the current data indicate optogenetic responses at this time, suggesting that the overexpressed A53T also may have already impacted the neural circuit at some degree (the largely skewed rotatory symmetric index of A53T mice at BL in Fig. 3i might represent a minor onset state). Strictly, it seems inappropriate to state this time point as 'presymptomatic time point' in line 739. The term 'baseline' should represent pre-surgical condition; otherwise, this time point should be referred to as '1-wk' or 'Week 1' to avoid confusion.
7.4. Group comparisons
: it would be beneficial if the authors compare A53T vs EV at 8 wk (or for both groups, 1-wk and 8-wk) to determine whether differences in the optomodulation of VN neurons exist between the groups.

8. Perithreshold stimulation condition
8.1. Light power determination
: It is unclear whether the light power for perithreshold stimulation was determined individually for each mouse or derived from an average value of previous results (lines 147-149). Please clarify this point and specify the exact light power used (e.g. in mW at the fibre tip).
8.2. Translation of '1 mA below threshold'
: The MS states that stimulation was set at '1 mA below threshold' (line 732). Could the authors explain how this current offset translates into light output in mW, and elaborate on the significance of the 1mA difference? Additionally, please clarify how this perithreshold light power differs from the VVS1 threshold (i.e. the light intensity at which the first vestibular symptom is observed).
8.3. Mechanistical considerations
: To better elucidate the mechanism underlying perithrhold stimulation, I recommend that the authors compare the cFos analysis under this condition (Fig. 2q VNC data; this seems better to come here for the verification of optogenetic conditions), alongside the other conditions (discussed above). Comparing the number and distribution of activated neuronal population between the perithrehold and VVS1 threshold conditions would provide valuable mechanistic insight.

9. Fig. 1 – Data segregation and group comparisons
9.1. The MS presents data from different experimental groups (EV and A53T), yet these data are mixed without clear separation or statistical comparisons. Additionally, data from the VN-mCh control group are not explicitly provided. Prior work (e.g. PMID: 36347898) has shown that glutamatergic receptor compositions in the VN can differ in PD models, suggesting that the A53T PD mouse model may exhibit distinct pathophysiological changes that affect VN neurons. Without showing the data from the EV and A53T groups separately and providing appropriate statistical comparisons, it is difficult to justify pooling them as a single optogenetic cohort. I strongly recommend that the authors clearly segregate the data between the different groups (and include the VN-mCh control data) and provide statistical comparisons to address any group-specific effects. This clarification is essential for accurate interpretation of the results and for ensuring methodological transparency.

10. Structural and functional mapping of VN connections
10.1. Connectivity mapping study
: the authors performed anatomical connectivity studies using viral tracings in normal mice and reported laterality in the VN targets (Fig. 2a-c). However, the MS lacks quantitative and statistical analysis of these data. Terms such as 'sparse' and 'dense' are used to describe projections, but it is unclear how these are defined or measured. I recommend that the authors provide numerical descriptions and statistical comparisons for the connectivity patterns (e.g. quantified metrics in the method section).
10.2. Functional mapping (cFos) study
: the authors use deepflash2 to segment and quantify cFos expression in 526 images from key regions (VNC, Gi, Pf and ZI),

and they report that the data are pooled from both hemispheres for analysis. Pooling assumes no significant differences between the ipsilateral and contralateral sides. However, the anatomical tracing data clearly reveal laterality in connectivity (Fig. 2c). This raises several questions: (1) what is the justification for pooling hemispheric data in the cFos analysis? (2) were there no significant differences between the two sides in the analysed regions? (3) would reporting differences (or the lack thereof) between the ipsilateral and contralateral VN provide insights into the robust bilateral communication in the VN, which might underlie balanced integration of vestibular information and potentially contribute to the therapeutic effects of nGVS or perithreshold stimulation? Considering that the optogenetic stimulation was applied to unilateral VN – and given that PD may further disrupt network balance – it is essential to assess whether any asymmetries exist in cFos activation. I recommend that the authors analyse and report cFos counts separately for the ipsilateral and contralateral sides (or as percentages relative to a control side) before pooling. This approach would clarify whether unilateral VN stimulation results in balanced activation across hemispheres or if significant differences exist that could impact the interpretation of the comparisons between the EV, A53T mCh groups (Fig. 2q-t).

11. A53T PD mouse model (part 2)
11.1. Comparison to previous study
: the authors modified their model generation protocol compared to their previous work (PMID: 28143577). Notably, the viral load used in this study (15 x 10E+12 gp/mL, 0.5 uL) is approximately 4 times higher than in their previous study (7.74 x 10E+12 gp/mL, 1.5 uL). Assuming the viral construct is the same (AAV1/2-CAG-A53T), it is surprising that the TH+ cell loss in the SNc in this study (~43% at 8-wk) is only modestly increased compared to the previous study (~33% at 10-wk) as a higher viral load would typically lead to a greater degree of DA loss. This discrepancy raises questions about the injection method (DV-serial injections) and site – Fig. 3a suggest it may be too medial – and whether the higher dose was effectively delivered to the target region. Additional explanation regarding these points seems critical for evaluating the robustness and comparability of the PD model.
11.2. Histological validation
: The histological validation is limited to 8-wk IHC images (Fig. 3a,c), even though the authors claim that TH+ cell counts are similar at 4- and 8-wk (Fig. 3b). To substantiate this claim, I recommend that the authors provide: (1) representative images and quantitative analyses of the TH fibre intensity (or complexity) in the striatum at both 4- and 8-wk; (2) SNc images from the 4-wk time point; (3) comparative data (e.g. percentages) between the ipsilateral and the contralateral sides. Additionally, please label the groups clearly (Fig. 3a,c) and, if possible, provide images for each channel separately for Fig. 3a, where the extent of DA degeneration is not clearly visible, along with clear indications of the corresponding areas in enlarged images in Fig. 3c.
11.3. Control group
: It would be beneficial to include a control protein overexpression group (e.g. AAV1/2-CAG-GFP) because high protein overloading is known to damage DA neurons in the SNc, (as discussed in previous studies, e.g. PMID: 28143577; PMID: 21029459). Given that the injection protocol has been optimised to deliver a high viral load, including such a control is essential for distinguishing the effects of the A53T mutation from potential protein overloading effects.
11.4. Data pooling concerns
: The authors state in lines 256-257 and 740-741 that they pooled 4-wk and 8-wk data for behavioural analysis, claiming that 'an independent cohort of A53T mice, euthanized at week 4 after injection of AAV1/2-A53T showed similar levels of dopaminergic cell loss'. However, overexpression of aSyn (A53T) is known to produce gradual pathophysiological and behavioural changes over time (PMID: 24267638; PMID: 37463889). The discussion (lines 521-523) also notes that the A53T model as 'mimicking the progressive course of PD', suggesting that pathological states at 4- and 8-wk might differ. If data from these time points are to be pooled, the authors should provide: (1) the histological validation demonstrating no significant differences between the 4- and 8-wk time points, (particularly in the striatum); (2) behavioural data showing comparable outcomes at both time points. Otherwise, it may be more appropriate to select a single, well-defined time point for analysis.
11.5. Behavioural data timeline
: It appears that most behavioural tests were performed at 8-wk, yet the data pooling raises concern regarding proper labelling. Please ensure that the data are accurately labelled (e.g. 'Week 8 off' should not include 'Week 4' data). A schematic diagram outlining the experimental timeline for the behavioural testes would be helpful if multiple time points are considered.
11.6. Cylinder test
: Aside from the baseline concerns (see, comment 7.3), the authors describe the effect of perithreshold optostimulation (PT) as a 'mild reduction' in the asymmetric rotation. Please provide quantitative details on the magnitude of this reduction and indicate whether the OFF and PT conditions differ significantly via t-tests. It may also help to reorganise the figure panels (j, k and i) so that data are presented in a logical sequence.
11.7. Perithreshold group in treadmill test
: the MS presents PT group only for the cylinder test (Fig. 3i) but not for the treadmill test (Fig. 3j,k). Clarification is needed as to whether the treadmill test was not performed for the PT group or if the results did not differ from the OFF state. This information is important for understanding the full impact of PT on motor function.
12. Pose estimation and behavioural analysis in OF
12.1. Data pooling and experimental time points
: the authors pool data from different time points (4-wk and 8-wk) for OFF and PT conditions for the EV and A53T groups. Notably, the timeline for the mCh (lines 744-746) does not match that of the experimental groups (OFF at 2-wk and PT at 3-wk). To ensure robust and comparable analyses, the authors should segregate the data for each group at each time point separately and align the experimental timelines across groups. Note, the latter is important especially for repetitive behavioural experiments in OF (lines 369-373).
12.2. Pipeline schematic for the analysis workflow
: fig. 4a occupies considerable space but lacks detailed information about the behavioural pipeline. A comprehensive

schematic outlining the entire process would help readers understand the methods: from video capture, keypoint extraction using DeepLabCut (with retained RCNN model), through to custom post-processing and behavioural classification with Keypoint-MoSeq; besides, details on dataset compositions, tracking accuracy metrics marked in the schematic.
12.3. Syllable presentation and organisation
: In Fig. 4b, only a subset of syllables with behavioural labels is shown, and the criteria for their selection remain unclear. I recommend that the authors organise all syllables hierarchically – ideally in a vertical dendrogram (Fig. 4c) – that includes the syllable number, the corresponding supervised behavioural labels, and key kinematic metrics (e.g. mean velocities) displayed via a heatmap. If space is allowed, representative images of each behavioural motif could be provided be included (Sub. Fig. 3). This would facilitate a more meaningful interpretation of the data.
12.4. Locomotor performance
: in lines 348-350, the authors report that PT stimulation enhanced the peak locomotor performance in VN-ChR groups (EV and A53T), as indicated by increased mean velocity and standard deviation (SD) of velocity within the running syllable (line 348-350, Fig. 4h and Ext. Fig. 2a). However, the data are confusing. There appears to be a significant difference in the OFF state between the VN-ChR groups and the mCh group (elevated relative to mCh group), yet the magnitude of the change from OFF to PT seems similar across all groups. Additionally, the mCh group's OFF to PT difference is reported as non-significant, but the absolute difference and large error bars in the PT state are concerning – while the SD of velocity for this group at PT shows much smaller error bars. It is unclear how the mean and SD of velocity within the running syllable were derived and normalised. Please provide more explanation of these calculations the p value for mCh group in Fig. 4h.
12.5. Additional concerns
: fig. 4j, why OFF states of the EV and A53T groups are already elevated compared to mCh? Fig. 4i, the mCh group's running duration under PT appears increased, despite the authors reporting a non-significant difference (p = 17). Fig. 4j, baseline differences between the optogenetic cohort and mCh are apparent, suggesting possible light-independent effect of ChR overexpression in VN. The authors should clarify these discrepancies by providing complete statistical comparisons (otherwise set the true baseline). Fig. 4j, the data for mCh group at OFF condition are missing, which limits interpretation in lines 372-373. Lastly, similar patterns of changes from OFF to PT across all groups (despite authors claim those are ns) raise the possibility that the PT condition itself might have no-specific effects (ChR-independent), possibly due to the PT experimental condition.
13. Gait dynamics study
13.1. Figure number
: in line 413-453, Fig. 5 seems mislabelled as Fig. 6, please correct the number.
13.2. Maximal velocity
: In line 417-418, I recommend including the mean values for the comparisons.
13.3. Suprathreshold light condition
: Please clearly state the exact light power (VSS2-3 threshold). Was a consistent light power used across subjects in all groups or differ to each mouse?
13.4. Fluidity of gait index (GFI)
: in lines 427-429, the authors report that PT stimulation does not alter gait (i.e. gait failure is not increased), whilst suprathreshold stimulation markedly enhanced gait failure for PD mice. In Fig. 5c, the data for the PD group suggests that the percentage of gait failure under PT condition is less than 1% of the total trial, which seems surprisingly low given that PD mice typically exhibit impaired gait. One would expect the PD group to display a lower FGI and more gait failure than EV group at least in the OFF condition. I recommend that the authors present the mean FGF values and the percentage of gait failures for both EV and A53T groups under OFF, PT, and suprathreshold conditions. Additionally, please provide a more detailed description of the FGI definition, particularly how gait interruptions are determined. This additional information would greatly improve the interpretability and reproducibility of the results.
13.5. Oscillatory gait pattern analysis
: the dual-view video recordings are captured at 30 fps (line 815), which implies a maximum resolvable frequency of 15 Hz according to the Nyquist theorem. However, the scalogram's y-axis is labelled from 0-30 Hz. The authors should clarify whether this is a labelling error, or if the data were resampled or processed in a way that extended the frequency range – this is not clearly described in the methods and appears inconsistent with standard signal processing principles. Additionally, it would be beneficial if the authors provided the corresponding frequencies (in Hz) for the peak powers observed (Fig. 5g-l, in lines 438-440) to aid in interpreting the gait dynamics. Furthermore, the MS would benefit from more detailed description of how autocorrelations were computed. As a minor suggestion, I recommend re-organising the panels in Ext. fig. 3 by placing the scalograms on left, so that the order aligns with the presentation in the main Fig.5 and the text.

Reviewer #2

(Remarks to the Author)
This very interesting, original, and timely study by Hartig and collaborators presents the first detailed investigation in parkinsonian mice into the effects of optogenetic stimulation of glutamatergic (Vglut2+) neurons in the vestibular nucleus complex, a promising therapeutic target for improving motor function in Parkinson's disease. While little is known about the mechanisms and motor effects of stimulating the vestibular circuitry, which has been gaining clinical interest, the authors make a significant and original contribution by filling this important knowledge gap. Notably, the results show that stimulation can improve locomotor performance under optimal conditions. The study is admirable for its novelty, high resolution, attention to detail, and its relevance to both the motor control and clinical fields.

Strengths:
1. New target for Parkinson's disease: This study is the first to investigate the potential effects of this new target in such detail. The study benefits from the authors' expertise, as they are leaders in both experimental models of Parkinson's

disease and the clinical application of deep brain stimulation, which enriches the study's depth and clinical relevance.
2. Original, high-resolution, unbiased analysis of motor behavior: The authors show that optogenetic stimulation of the vestibular nucleus complex can have a nuanced impact on motor behaviors, depending on stimulation strength. Using advanced deep learning-based movement analysis, they classified behavior into short, identifiable coordination patterns called syllables and identified transition patterns between them. They also examined whether specific transitions were induced or if stimulations had particular effects on behavioral transitions between syllables. This high-resolution approach is impressive and offers valuable insights into how stimulation may affect movement at a finer level. The study also explores the effects of gradual stimulation, providing a comprehensive description of possible motor effects as a function of stimulation strength, thus creating a "behavioral atlas" of potential outcomes with direct clinical relevance.
3. The Parkinson's model: The model used in this study involved intra-substantia nigra injections of an adeno-associated virus encoding the mutated A53T version of α-synuclein. This model, previously characterized by some of the authors, successfully recapitulates key features of Parkinson's disease, such as chronic dopamine depletion and abnormal motor behavior within 8 weeks, allowing the authors to test preclinical interventions.
4. Value to both clinical and fundamental research: The findings are valuable not only to the clinical field but also to fundamental research, offering unprecedented, detailed behavioral analyses of the target region, which are of great interest to the motor control community.
5. Foundation to study the pathway involved: The authors use functional neuroanatomy (c-fos expression) to identify specific brain areas activated by the stimulation, laying a solid foundation for future research into the underlying pathways involved.
6. Timeliness and importance: This research is timely and important, providing critical insights into the complexities of the substrate at an early stage. Such studies are essential for refining stimulation parameters and preventing the premature dismissal of potential targets in human trials due to an incomplete understanding of the cell types or circuits affected by its electrical stimulation. The approach taken by the authors is commendable for its foresight and meticulous attention to detail.
7. The study is well-written, the analysis was carefully performed, and the conclusions are precise and well-balanced. The methodology is sound, and the work meets the expected standards in the field. There are sufficient details in the methods section for the work to be reproducible.

Weaknesses:
No major weaknesses detected. No additional experiments are needed. Only some minor details remain unclear, but these will be very easily clarified by the authors by simply modifying the text and/or figures (see list below).

Details:
Abstract:
• "Improved locomotor performance but not capacity." This phrase is vague and should be explained more clearly for a broader readership.

Introduction:
• L42: When reporting MLR studies, it is important to distinguish between cell type-specific studies in animals that have successfully identified specific populations to stimulate (as in the present study) and clinical studies using deep brain stimulation (DBS), which likely cannot (yet) target pro-locomotor neurons and have reported mixed results. Acknowledging this difference in the text is crucial to avoid prematurely dismissing the MLR target (or any other brain target) based solely on DBS studies conducted before the circuitry was thoroughly examined in animal models and before the identification of relevant target cell populations in preclinical studies. It could be explicitly mentioned that targeting specific pro-locomotor cell subtypes with DBS remains a challenge. This also provides an likely explanation for the cause of mixed results in the MLR.
• L49: "This technique has shown promise" – Please be more specific about the effects reported.
• L53: "Improve Parkinson's symptoms in rats" – Please clarify which symptoms were improved and how.

Results:
• "In optogenetic pilot experiments": It would be nice to provide even in the main test, at least once, details about the stimulation parameters (train duration, stimulation frequency, pulse duration) and some basic information, such as how long after stimulation the behavioral effects are observed.
• L150: "Yet, repeated stimulation may idiosyncratically change thresholds" – Could you explain this in simpler terms for a wider audience?
• L183-184: "We took a myc-tagged AAV184 delivered anterograde synaptophysin tracer injected into the VNC" – Please specify whether the injection was unilateral.
• L262: Rotation on which side? Body turns? Circling? Towards which side? Ipsi or contra to stim? This information is not always provided. Consider improving precision throughout the paper.
• Concerning limb kinematics, I could not find a raw image of limb kinematics from which the wavelets were extracted. Maybe add a panel somewhere if possible?
• L268: "Correlated" – negatively or positively? Could you please specify systematically everywhere in the paper?
• Fig. 4i: It appears strange that the off-PT for mCherry is so high on the right. It is almost significant, but it is not. Is there an explanation for this?
• L332: "With perithreshold optoactivation" – Be specific about what "peri" means. 0.75% to 1.25%? Can you provide a more specific range?
• L346: "Bird's eye view" – Perhaps it would be useful to show the raw kinematics and raw trajectories in some panels.
• L361: "We found that those behaviors bear a covert representation in behavioral modules under low-intensity stimulation conditions." Please clarify.
• L427: What is the effect of the synuclein injection on gait failures (i.e., before any optogenetic stimulation)?
• L431: "Smoothening of the gait" – Please make it more quantifiable. It sounds a bit abstract.

• L439: I like the wavelet analysis, but it's difficult to understand what it corresponds to in terms of movement. What does a reduction in power in the 20 Hz band signify, and how much of an aberration does this represent at the movement level?

Discussion:
• L459: his section could offer a clearer, movement-based perspective. Would it be interesting to comment on how much these stimulations mimic human effects obtained when targeting the vestibular circuitry?
• L513-516: Perhaps this is the right place to specify the difference between locomotor capacity and locomotor performance.

Reviewer #3

(Remarks to the Author)
This manuscript from the Tovote and Ip labs set out to study mechanisms and therapeutic effects of vestibular nucleus complex (VNC) stimulation in healthy rodents as well as a model of Parkinson's disease (PD). Strengths include detailed circuit tracing focused on the VNC network, use of subthreshold optical protocols to modulate neuronal activity in a manner relevant for translational applications, a very thorough and careful use of machine learning approaches for their behavioral studies (this was excellent!), and an elegant PD model. This study clearly enhances our knowledge of VNC function and has direct relevance to motor symptoms of PD mice. That said, in my opinion, data presentation and clarity of the figures/text requires some serious work before moving forward. I found myself searching for the relevant data panels for several results described in the text (which is unacceptable and is not ideal for the readers), sometimes necessary controls or supportive data for an experiment/technique showed up few figures later (see details below), and their molecular/microscopy work was not presented in a convincing manner.

The study is well-designed. I have the following suggestions to strengthen this study, which will enhance its' impact in the field (in the order that they appear). If I have misunderstood any points, please help point me in the right direction.

1. When I read the abstract and main text, they give the impression that VNC circuitry is functionally examined but that is not the case. All their manipulations are focused on VNC cell bodies (i.e., no terminal work). If they want to mention their circuit work, this should be clarified to indicate anatomical studies only.

2. Fig 1a-b. I honestly cannot examine the micrographs to a rigorous level due to their small size. For essential staining/tracing/labeling data throughout the paper, please provide zoom-out and zoom-in images to help the reader appreciate the accuracy/specificity.

3. Line 92. When they first introduce the A53T approach, they need to state unilateral or bilateral targeting. This is very important for PD and the motor behavior field. Related to this point, when the model is first introduced, we would expect a thorough characterization of this AAV approach and yet some data only show up I believe in Fig 3, which is not acceptable. Relevant panels should be moved here or in a supplemental figure that can be referenced when this model is first introduced. For this PD model, they need to provide alpha-synuclein expression, viral spread, cell death quantifications, and a representative injection site (some of this is already in Fig 3 but please confirm).

4. Line 99. They need to provide these pilot data or clearly indicate which panel this is referring to. Results without supportive data should be avoided. Check the entire paper for a similar issue, please.

5. Fig 1c. Add exact mW values to the graph. Same for other graphs throughout the paper. This is very important information.

6. Lines 113-116. Where is this data? If not included in the paper, please provide them as a bar graph per mouse.

7. Fig 1e. The 5 colors chosen are clearly not distinct enough as it's not immediately clear to my eye upon first reviewing this panel where the clusters are. Please try to choose a color panel that is clearer to the reader.

8. Fig 2e. This data should be enlarged otherwise it's not very helpful to the reader.

9. Line 131. For performance, since two occasions led to ~90% accuracy, I think it's better to provide the exact values for each (not using ~). This is important validation data of their approach.

10. Lines 132-134. Provide UMAP cluster data with a brief description followed by these conclusions for enhanced clarity.

11. Line 140. VSS1 is at 0.05 and for accuracy should be called out in the text as showing a 'very strong trend', for instance. This will alert the reader in a helpful manner.

12. Lines 141-143. Please add a concise interpretation of this observation.

13. Lines 147-151. Why is this described here when the experimental application does not follow? Please move this to the manuscript section right before it will be tested in mice, otherwise it is very confusing.

14. In Fig 1, the authors need to indicate which data points correspond to A53T vs. EV mice. Even if there are no differences,

this is critical to evaluate these data. Also, these animals are distinct and therefore possibly using different color data points, this is easy to highlight (e.g., panels g-j and k).

15. Fig 2b-c. Please add mScarlet data to this summary table.

16. Fig 2. For microscopy images, please add the anterior-posterior information either on the images themselves or in the legend text. Do this across the paper please.

17. Line 195. Should the Fig 2f-i just be 2f? Double check. Also, the Po data is not useful as presented. Provide a full-size image.

18. Fig 2m-n. Is panel n a different coordinate section than panel m? If not, why is CnF not labeled in panel m? If these two panels are from different coordinates (even slightly), please provide better matched images to be more convincing.

19. Line 207. Add unilateral or bilateral targeting information here.

20. Line 212. I know what OF may mean but others may not. For each abbreviation when first used, add a full name.

21. Line 213. The cFos data for PF is a trend and this should be highlighted here for accuracy. For each cFos group/graph, please add representative cFos images for further support.

22. Fig 3. For model validation, I don't believe a full figure is very helpful. Instead, the other figure panels if increased in size and the authors need more main figure space, some of this figure could be used. Just a suggestion.

23. Fig 3a. Please provide separate channels for each image in the supplemental data.

24. Fig 4i. How to explain the clear trend in the mCherry group? This matches the A53T/EV groups and is concerning. Please add text to the paper to justify/explain.

25. Fig 4j. Add mCherry OFF data to support habituation effect claim (since from BL to OFF shows the biggest decrease in the other graphs).

26. Fig 6b. Add an mCherry group here as an important control.

27. Fig 6u. The authors should describe the significance of the 20 cm/s data being n.s. between peak amplitude and peak frequency.

28. EDF1. Confirm that it is referenced in the main text.

Version 1:

Reviewer comments:

Reviewer #1

(Remarks to the Author)
General opinion
Overall, the authors have made a substantial effort to revise the manuscript, and several methodological details and additional datasets have been added. However, the concerns raised in the initial review are only partially addressed. Across multiple sections, a recurring issue remains a mismatch between the evidential strength of the data and the level of mechanistic or translational interpretation applied. Several datasets—most notably the anatomical tracing (Figures 1–2), the optogenetic titration experiments, and the c-Fos mapping—are inherently exploratory or descriptive in nature, often based on limited sample sizes, yet are framed using language that implies causal hierarchy, functional integration, or therapeutic relevance. In several instances, the conclusions would benefit from more cautious wording that better reflects the scope and limitations of the underlying data.
In addition, aspects of the authors' responses to reviewer comments rely on interpretative or assumptive statements rather than direct empirical evidence. Whilst these statements may represent reasonable hypotheses, they do not substitute for explicit reporting of the requested analyses (such as group-stratified classifier performance metrics) and occasionally deflect from the specific questions raised.
Taken together, whilst the revised manuscript contains valuable data and technical advances, further revisions are required to (i) align interpretation more closely with evidential support, (ii) temper mechanistic and translational claims where direct validation is lacking, and (iii) more directly and quantitatively address several outstanding methodological concerns. Addressing these points would substantially strengthen the rigor, clarity, and interpretability of the study.

Reviewer#1 comments
Comment 1.2.
We carefully reviewed the revised Methods section, Supplementary Tables, and the figures referenced by the authors. This comment is partially addressed. Whilst the stimulation paradigms for the titration experiments are now described in extensive technical detail, the core concerns regarding how stimulation intensity is defined and controlled remain

insufficiently resolved. In the Methods and Supplementary Tables, stimulation intensity is still operationally defined in terms of current (mA), with an approximate and unvalidated conversion to optical power, which limits reproducibility across animals and laboratories.
Although the authors now report laser power at the fibre tip (µW) across VSS1–4 and between groups (Fig. 4), this reporting is not consistently aligned with the experimental definition of stimulation intensity, which remains primarily current-based. For clarity and reproducibility, optogenetic stimulation should be defined and reported consistently in terms of optical power, with behavioural thresholds used only for individual calibration rather than as primary descriptors of stimulation strength.
Furthermore, whilst Figure 4 reports individual-animal laser powers associated with each VSS category, the assignment of VSS remains based on the 'most prominent behaviour' (line 1074) observed per animal rather than on a predefined quantitative criterion. The authors should provide a quantitative definition of the 'most prominent behaviour' is determined. In addition, within-animal threshold transitions are not shown, limiting interpretability at the individual-animal level.
Finally, although the authors refer to Supplementary Figures 5 and 6, we do not find information in these figures that directly addresses the above concerns.

Comment 2.2.
Whilst the authors state that model validation data are now presented earlier, the main validation results are largely relegated to the Supplementary section. Consequently, the logical dependency between disease model establishment and the subsequent tracing and optogenetic experiments remains insufficiently delineated in the main text. Simply reordering sections, without foregrounding model validation as a central result, does not fully address the original concern.
Importantly, the anatomical tracing data presented in Figures 1 and 2 were obtained in non-parkinsonian mice and therefore represent baseline VNC connectivity. Whilst these data are informative, the manuscript implicitly treats them as a structural foundation for interpreting functional and therapeutic effects in the parkinsonian A53T model. In the absence of evidence that these circuits are preserved, altered, or functionally engaged in the disease context, such extrapolation is not sufficiently justified.

Comment 2.3.
Whilst the authors now present A53T and EV titration data separately (Figs. 3 and 4), the interpretation that A53T animals exhibit vestibular hypo- or dysfunction is not directly supported by the evidence presented.
The data convincingly demonstrate a shift in optogenetic stimulation thresholds, such that higher optical power is required to elicit specific VSS categories in A53T mice. This supports the conclusion that A53T mice show reduced responsiveness in terms of optogenetic threshold under the defined stimulation paradigm.
However, threshold shifts alone do not establish altered vestibular function per se. No independent assessment of baseline vestibular behaviour or reflexes in the absence of optogenetic stimulation (e.g. vestibular reflexes or balance-related assays) is reported. Whilst the additional cFos analysis confirms effective optogenetic engagement of VNC neurons (Supplementary Fig. 12), it does not establish intrinsic vestibular hypo- or dysfunction under baseline conditions, and interpretations should therefore remain limited to altered optogenetic responsiveness.
Accordingly, interpretations invoking vestibular hypo- or dysfunction should be tempered and reframed in terms of altered optogenetic responsiveness or stimulation thresholds, unless supported by additional, independent validation of vestibular function.

Comment 3.2.
The issues raised here largely overlap with those discussed in Comment 1.2 and are therefore not reiterated separately.

Comment 3.4.
The authors address this point by showing that optogenetically induced postural effects are temporary and rapidly reversible (Supplementary Fig. 9), and by discussing differences from previous studies in stimulation duration and pulse parameters. Whilst these observations are informative, they do not fully resolve the concern raised.
Specifically, the reversibility of overt postural effects does not exclude the possibility of residual or cumulative effects on vestibular circuit excitability across successive stimulation blocks. The manuscript does not provide an experimental control in which each VSS-associated light intensity is applied as an independent condition, without prior stimulation history, as originally suggested.
Therefore, it remains unclear whether the emergence of distinct VSS categories reflects light intensity alone, or is influenced by repeated stimulation, temporal accumulation, or order effects. This limitation should be more explicitly acknowledged in the interpretation of the titration experiments.

Comment 4.4.
The original comment asked a single, specific question: whether prediction accuracy differed between the A53T and EV groups. Whilst the authors' response describes revisions to the training procedure and reports overall classifier performance, it does not directly address this point, as group-stratified performance metrics are not provided. Please report classifier performance separately for A53T and EV (e.g. precision, recall and F1 score, and ideally confusion matrices) on the held-out test set to directly address this point.

Comment 4.5.
Whilst the alluvial-style plot may be visually intuitive, it does not provide the requested group-stratified quantification. Please include a concise table reporting human–machine agreement (counts and percentages) for each VSS category, separately for EV and A53T, so that any group-dependent performance differences can be assessed transparently.

Comment 7.2.
Whilst the discussion has been expanded, the statement that the authors 'found signs for vestibular hypoexcitability' in PD

mice (line 630) remains stronger than what is directly supported by the presented data (see Comment 2.3). The results demonstrate altered optogenetic stimulation thresholds and responsiveness, but do not independently establish baseline vestibular hypo- or hyperexcitability. The authors are encouraged to further temper this terminology, or to explicitly frame these findings in terms of altered optogenetic responsiveness or circuit-level modulation.

Comment 7.3.
The authors have corrected the time-point labelling for clarity. The original comment arose specifically because week 4 data were labelled as 'baseline' in the originally submitted manuscript, which created ambiguity regarding the temporal interpretation of the results. The concern was therefore terminological, not related to experimental feasibility or expectations regarding viral expression at early time points.
In this context, the explanation of why titration experiments were not performed at week 1 does not directly address the source of the confusion. Rather, the issue stems from mislabelling, which in turn prompted the original comment.
Whilst the authors indicate that the relevant labels have now been corrected (i.e. 'baseline' replaced with the appropriate time point), given the importance of temporal definitions for longitudinal interpretation, the authors are encouraged to re-check all figures, supplementary figures, and legends to ensure consistent and unambiguous labelling of time points throughout the manuscript, so as to avoid future confusion or misinterpretation (see also Supplementary Fig. 6g; minor comments below).

Comment 10.2.
The authors have provided the requested data. The c-Fos analysis provides useful evidence for region-specific activity changes in VNC target areas following perithreshold stimulation; however, aspects of the current framing and interpretation go beyond what is directly supported by c-Fos expression alone.
In particular, the subsection heading and some concluding statements imply a level of functional integration and causal network hierarchy that is not established by these data. c-Fos is an indirect proxy of neuronal activity and does not, by itself, demonstrate directionality, functional coupling, or circuit-level integration.
Given the limited sample sizes per group and the exploratory nature of this mapping, the authors are encouraged to temper the language throughout Figure 5 and the associated text, and to frame these findings more cautiously as evidence of activity engagement and/or recruitment in specific regions, rather than definitive 'axis'-level modulation or an 'interface' between systems.

Comment 11.3.
Whilst the authors argue that control protein overexpression is unlikely to account for the observed effects, subtle contributions of expression burden under the current experimental conditions cannot be fully excluded. The conclusions should therefore be interpreted with this consideration in mind.

Comment 11.6.
The reported 'mild reduction (5.5%)' (line 251) is not accompanied by a statistical comparison between OFF and PT within the A53T group. Please indicate in the main text whether the OFF vs PT difference is statistically significant, including the statistical test and exact p-value.

Comment 11.6.
The authors have expanded the Methods and improved the transparency of the Keypoint-MoSeq analysis. Whilst the current presentation is acceptable, the figure still functions primarily as a high-level conceptual overview rather than a step-by-step analysis pipeline. An improved workflow schematic would substantially improve readability.

Comment 12.3.
The authors chose to remove in-syllable analyses, but this does not resolve the original concern regarding the selection criteria and organisation of behavioural syllables. The manuscript still lacks a clear and systematic presentation of the full syllable repertoire, which limits interpretability of the KPMS results.

Comment 12.4.
Whilst the authors chose to remove the in-syllable kinematic analyses, this does not resolve the original concerns regarding baseline group differences, similar OFF–PT changes across groups, or the possibility of non-specific effects of the PT condition.

Comment 13.4.
Although the authors provide a detailed and transparent description of the FGI scoring system, the interpretation of the corresponding results is not fully supported when considered alongside the broader locomotor data presented in Fig. 7.
First, the authors state that 'there is no massive gait failure of W1 vs. W8-OFF' because the A53T phenotype is relatively mild with respect to hypokinesia. However, Fig. 7b demonstrates a clear and significant reduction in maximal treadmill velocity in A53T-ChRmine mice compared to EV controls, indicating a measurable locomotor impairment. This discrepancy suggests that, whilst overt gait failure events are rare under OFF conditions, the model nevertheless exhibits meaningful baseline motor deficits that are not captured by the categorical FGI metric.
Second, baseline (OFF) gait failure rates in Fig. 7c,d are low in both A53T- and EV-ChRmine mice and do not robustly differentiate the parkinsonian group from controls. This indicates that, as implemented here (FGI1 vs. FGI2+3), the FGI readout may have limited sensitivity for detecting disease-related baseline gait impairment, despite the presence of hypokinetic features revealed by velocity measures.
Third, perithreshold (PT) stimulation produces little to no measurable change in gait failure relative to OFF in either group. Whilst this is consistent with the authors' statement that PT does not worsen gait, it does not provide convincing evidence for

a beneficial or rescuing effect at the level of gait failure. The description that impaired gait is ‘mildly reduced’ by PT is difficult to reconcile with the categorical nature of the FGI and the minimal visible difference between OFF and PT conditions. Finally, the use of trial counts pooled across animals further complicates interpretation, as it remains unclear whether any apparent differences reflect consistent within-animal effects or are driven by variability in trial numbers contributed by individual animals.
Overall, the FGI data convincingly demonstrate the deleterious effects of suprathreshold stimulation but do not support a meaningful improvement in gait failure with perithreshold stimulation. The authors are encouraged to more clearly acknowledge the limited sensitivity of FGI to baseline hypokinetic deficits, to align statements regarding disease severity with the velocity data in Fig. 7b, and to clearly distinguish FGI-based outcomes from the oscillatory gait ‘retuning’ effects described in subsequent analyses.

Additional major comment (Figures 1–2 and related text)
In the revised manuscript, the authors added extensive qualitative brain-tracing images and analysis. In the current manuscript, Figures 1 and 2 form the foundation of the manuscript and present extensive anatomical tracing of Vglut2-VNC output and input circuits. Whilst these datasets are valuable and technically sound as exploratory brain-wide mapping studies, their current presentation and interpretation extend beyond what is supported by the underlying data. Both figures are based on n = 3 animals and primarily provide descriptive connectivity maps, yet the text repeatedly uses quantitative and functional language (e.g. ‘quantitatively predominant targeting’, ‘hyperdirect influence’, and ‘anatomical prerequisite for modulatory impact’) that implies functional hierarchy and translational relevance.
In this context, the absence of inter-animal variability measures, individual data points, or other indicators of robustness makes such strong framing difficult to justify. Whilst n = 3 and error-bar–free representations are not uncommon in tracing studies, they are typically accompanied by more cautious, descriptive language. As currently written, the manuscript treats these anatomical results as a functional and conceptual foundation for downstream perithreshold stimulation experiments and therapeutic analogies, which creates a mismatch between data strength and claim level.
We therefore believe that this section requires substantial revision, including a more restrained interpretation of Figures 1 and 2, clearer acknowledgement of their exploratory nature, and tighter alignment between the anatomical data and the functional/translational claims built upon them. Without such revision, the anatomical results risk being over-interpreted relative to their evidential scope.
Additional minor comments
- The labelling in Supplementary Fig. 6g appears potentially inconsistent. Could the authors please verify the labels for image rows 3 and 4? In addition, based on visual inspection, the Week4 panels seem to show fewer SNc TH+ neurons than the Week8 panels in EV-WT, which appears counterintuitive given the reported quantifications. Please clarify whether the correct images/time points are shown and, if applicable, provide the corresponding quantification for the specific panels displayed.
- Figure 5i legend in the figure is cropped.
- Line 251, please correct the decimal separator from 5,5% to 5.5%.
- Label A53T-ChRmine (OFF) and A53T-ChRmine (PT) on the Fig7e and F respectively to improve readability.

Review #2 comments
Abstract: Addressed in part. The revised abstract removes the ambiguous phrase, but it still does not clearly define what is meant by ‘performance’ versus ‘capacity’ in measurable terms (i.e. which readouts correspond to each), which was the core request for broader readership.

Introduction
• L42: Addressed.
• L49: Addressed.
• L53: Addressed.
• ‘In optogenetic pilot experiments’: This point is partially addressed. Whilst detailed stimulation parameters are now provided in the Methods and Supplementary Tables, the reviewer’s request to include a concise description of key stimulation parameters and the temporal profile of behavioural effects at least once in the main text has not been fully met.
• L150: Addressed.
• L183-184: Addressed.
• L262: Addressed.
• Limb kinematics raw image: Addressed.
• L268: Addressed.
• Fig. 4i: The reviewer’s question regarding the unexpectedly elevated OFF–PT values in the mCherry group remains insufficiently addressed. While the authors note the removal of in-syllable kinematic analyses, this does not directly explain the near-significant elevation observed in the control group. Clarification regarding potential contributing factors (e.g. inter-animal variability, sample size, outliers, or analytical considerations), or an explicit acknowledgment that this variability remains unexplained, would improve transparency.

• L332: The revised figure clarifies that perithreshold stimulation was implemented as a fixed offset below each animal’s VSS1 threshold (10 mA, corresponding to ~1–2 µW at the fibre tip), which partially addresses the reviewer’s request. However, for reproducibility, it would be helpful to also provide a concise operational definition of ‘perithreshold’ in the main text, ideally in power-based terms and/or as a relative range or percentage of threshold. In addition, clarification of how the mA-to-µW conversion was calibrated across animals would further strengthen methodological transparency.

• L346: addressed.
• L361: The response does not fully clarify how the reviewer's concern regarding the conceptual meaning of 'covert representation' under low-intensity stimulation was resolved. While the authors note that in-syllable kinematic analyses were removed, it remains unclear whether the statement itself was removed, rephrased, or conceptually withdrawn in the revised manuscript. Please explicitly indicate how this issue was resolved in the revised manuscript.

• L427: The authors' response clarifies that the primary purpose of the gait failure metric (FGI) was to contrast suprathreshold versus perithreshold stimulation. However, this does not directly address the reviewer's original question regarding baseline gait failure in A53T mice prior to optogenetic stimulation (e.g. W1 or W8-OFF). Notably, Fig. 7b shows a significant reduction in maximal treadmill velocity in A53T-ChRmine mice relative to EV controls, indicating a measurable baseline locomotor impairment. In this context, the low incidence of gait failure events in Fig. 7c,d may reflect a limited sensitivity of the FGI to baseline hypokinetic deficits, rather than an absence of disease-related motor impairment. Explicitly clarifying what conclusions can and cannot be drawn about baseline gait failure from the FGI data, and acknowledging that the FGI primarily captures overt gait breakdown rather than more subtle hypokinetic features revealed by velocity-based measures, would help align the interpretation across figures.

• L431: The authors appropriately clarify that 'smoothing of gait' was initially descriptive and is now followed by oscillatory analyses (Fig. 7; Supplementary Fig. 14), which provide quantitative support for specific gait-related signals. However, several reported effects appear modest and/or marker- and speed-dependent, and it is not always clear that they generalise across parameters. To avoid over-interpretation, the authors are encouraged to (i) temper terms such as 'resynchronisation/retuning', or explicitly define them operationally, and (ii) provide a concise summary (direction and effect size and/or a summary statistic) indicating which oscillatory metrics show consistent PT-associated changes across speeds/markers versus more selective or exploratory effects.

• L439/L459: While the oscillatory analyses are described as in-depth and robust, the reported effects are largely confined to specific conditions, most notably a single speed (20 cm/s) and selected ipsilesional limb markers. Effects are not consistently observed across speeds, markers, or coordinates, and several findings are described as trends or mild changes. In this context, phrases such as 'retuning towards week1 levels', 'resynchronisation', or 'harmonisation' may benefit from more cautious framing or explicit operational definitions. A brief summary highlighting which oscillatory metrics show statistically supported and consistent PT-associated changes, versus more condition-specific effects, would further strengthen the interpretation.

Reviewer #3

(Remarks to the Author)
They did a good job with this revision.

Fig4c-d = I would change the "yellow" datapoints since they are very hard to locate.

Version 2:

Reviewer comments:

Reviewer #1

(Remarks to the Author)
General opinion
I thank the authors for their careful and substantial efforts in addressing the previous round of comments. The revised manuscript is clearly improved in multiple aspects, particularly with regard to methodological transparency, interpretational tempering, and additional analytical clarification. Importantly, most of the major scientific concerns raised previously have now been adequately addressed without requiring additional experimental work.
In particular, the additions regarding group-stratified classifier performance metrics, the expanded explanation of the A-SOiD workflow, the clarification of sham perithreshold stimulation effects, and the moderated interpretation of locomotor 'rescue' versus locomotor 'performance' are all appreciated and substantially strengthen the manuscript.
After carefully cross-checking the rebuttal against the revised manuscript, I find that the majority of my previous concerns are now satisfactorily resolved. The remaining issues are relatively minor and relate primarily to interpretational consistency, presentation, and methodological clarity.
In particular, whilst the anatomical tracing sections are now framed substantially more cautiously and appropriately as exploratory analyses, a small number of stronger interpretational expressions still remain in isolated parts of the manuscript and figure legends. Ensuring that the intended interpretational tempering is applied consistently throughout the text and figures would further improve clarity and consistency.

Comment 1.2
Whilst the revised Methods now clarify that each VSS category corresponds to the first occurrence of the respective symptom pattern, some individual trajectories in Fig. 4h appear non-monotonic across VSS levels. It therefore remains somewhat unclear how symptom categories were assigned in cases where higher-order symptoms emerged at lower stimulation powers than lower-order categories. Adding a brief clarifying sentence in the Methods or figure legend describing

how such cases were operationally handled would further improve interpretability.

Comment 2.2
The authors have substantially tempered the original anatomical-functional interpretation and appropriately acknowledge the exploratory nature and limited sample size of the tracing experiments. This substantially improves the alignment between the evidential scope of the data and the interpretation. However, several stronger interpretational terms remain in parts of the revised manuscript and figure legends.

Comment 3.4
Adequately addressed. Whilst the potential influence of stimulation history or cumulative vestibular activation is not experimentally excluded, the revised manuscript now explicitly acknowledges this limitation and appropriately tempers the interpretation of the titration experiments accordingly. No further experimental clarification appears necessary.

Comment 4.5
The additional group-stratified matching analysis is helpful and substantially improves transparency of the classifier performance. However, the reported human-versus-machine matching counts for several VSS categories appear relatively small (e.g. n = 2–3 events), making it somewhat difficult to assess the robustness of the validation procedure. A brief clarification regarding how these event counts were derived (e.g. event-level versus video-level scoring, number of human annotators, and whether validation was performed by a single or multiple raters) would further improve interpretability. Including this clarification in the Methods section or figure legend would be helpful. Given the relatively small sample sizes underlying some of the reported perfect matching values, the authors are encouraged to interpret these classifier validation results cautiously and avoid overstating their robustness.

Comment 11.6
The expanded methodological description of the Keypoint-MoSeq pipeline is appreciated. However, this still does not fully substitute for the previously requested static workflow schematic illustrating the complete KPMS analysis pipeline in a concise stepwise format. Given the analytical complexity of the manuscript, inclusion of such a schematic would substantially improve readability, accessibility, and reproducibility for readers who are not already familiar with the pipeline.

Comment 12.3
The authors provide a reasonable rationale for avoiding selective, manually curated syllable analyses and for instead focusing on the full behavioural state space. However, this does not fully resolve the original concern regarding the systematic presentation and interpretability of the complete syllable repertoire. Whilst Supplementary Video 5 is useful, a concise static supplementary figure or table summarising all syllables (e.g. index, relative frequency, and brief descriptor/example frame) would substantially improve accessibility and interpretability of the KPMS results for readers.

Minor presentation consistency
Minor formatting inconsistencies remain (e.g. mixed decimal notation styles) and should be standardised throughout the manuscript prior to publication.

Summary
Overall, I believe the manuscript is now substantially strengthened and that the remaining issues are relatively minor, primarily relating to presentation, consistency, and accessibility rather than underlying scientific validity. I do not believe additional experiments are necessary. Provided that the above remaining points are adequately addressed, I would be supportive of publication.

Open Access This Peer Review File is licensed under a Creative Commons Attribution 4.0 International License, which permits use, sharing, adaptation, distribution and reproduction in any medium or format, as long as you give appropriate credit to the original author(s) and the source, provide a link to the Creative Commons license, and indicate if changes were made.
In cases where reviewers are anonymous, credit should be given to 'Anonymous Referee' and the source.
The images or other third party material in this Peer Review File are included in the article's Creative Commons license, unless indicated otherwise in a credit line to the material. If material is not included in the article's Creative Commons license and your intended use is not permitted by statutory regulation or exceeds the permitted use, you will need to obtain permission directly from the copyright holder.
To view a copy of this license, visit https://creativecommons.org/licenses/by/4.0/

REVIEWER COMMENTS

Reviewer #1 (Remarks to the Author):

General opinion
The manuscript (MS) addresses an innovative and clinically relevant topic, utilising an optogenetic tool in combination with advanced AI-assisted behavioural analyses to investigate the therapeutic potential of vestibular modulation in a Parkinson's disease (PD) mouse model. The experimental design and employed techniques are rigorous and sophisticated, with significant potential implications for understanding the role of vestibular circuitry in posture and gait dysfunction associated with PD.
However, several methodological and interpretational issues need clarification and improvement. Key concerns include insufficient clarify on the experimental timelines and inconsistent data pooling across different time points and groups, ambiguous or incomplete methodological descriptions, and unclear or potentially misleading data presentation. Addressing these issues would substantially enhance the MS's clarity, reproducibility, and overall scientific rigor.
My detailed comments are provided below for further clarification and improvement. Given the extensive nature of our comments, we have structured them sequentially, aligned with the manuscript's organisation. Among these comments, the key concerns we consider particularly important include comments numbered 3, 8-12 (major).

1. ChRmine
1.1. In line 89-90, ChRmine is first introduced as an optogenetics. Given the wide range of available optogenetic tools, please consider providing a brief description of ChRmine for readers less familiar with the tool. For example, you might state: 'we utilised an optogenetic tool, ChRmine, which is a red-shifted, cation-conducting channelrhodopsin, that excites neurons upon red-light stimulation', drawing from additional context provided in lines 718–719.

- ➔ **We appreciate the reviewer's help in improving the clarity of the manuscript for the broad, interdisciplinary readership of the journal. We adapted the statement as such: "For vestibular nucleus stimulation, we used optogenetics by introducing the photosensitive actuator *ChRmine* [42,43] (a red-shifted, cation-conducting channelrhodopsin that excites neurons upon red/orange-light illumination) into the VNC of *Vglut2*-cre mice (Fig. 3a)."**

1.2. The MS does not provide sufficient detail regarding the light conditions, particularly the light powers, or the verification of neuronal activation in their experiments (more discussions later).

- ➔ **We thank this reviewer for pointing out the lack of detail on the light conditions in optogenetic experiments. Responses are found in the respective sections where specifics are addressed; overall we have now included concrete statements of light powers/light conditions within the Methods section on lines 1053-1096 in detail. We included two supplementary tables that outline the paradigm parameters further under Supplementary Figure 5 & 6.**

1.3. It appears that the original publication for ChRmine (PMID: 31320556) is not referenced. Please include this reference to allow readers to verify the properties of this tool in the original paper.

- ➔ **We have now referenced the paper in the Results section on lines 225-226.**

1.4. The MS mentions the construct 'AAV2/5-hSyn-DIO-ChRmine-mSccarlet' in lines 719-720 but does not provide the Addgene ID or cloning details. Please include the relevant Addgene ID(s) and briefly describe any subcloning steps (for example, if you subcloned the DIO construct using Addgene plasimid #130994) to ensure reproducibility.

➔ **We systematically checked and adapted this accordingly under lines 932-950 and 1042-1051. We noticed that we falsely stated expression under the hSyn (human synapsin) promotor, when indeed both viruses were expressed under the Ef1a promotor (the lab uses both constructs). We corrected this error and apologize for not having noticed this earlier.**

2. A53T PD mouse model (part 1)
2.1. Model generation details
: the MS does not provide sufficient information regarding the generation of the A53T overexpression PD mouse model. Based on the authors' previous publications (PMID: 28143577; PMID: 21029459), it appears that the AAV1/2-A53T construct likely uses the CAG promoter. For viral-based overexpression models, different promoters can have significant impacts on the expression levels and patterns. Thus, please clearly state the full format of viral construct (e.g. AAV1/2-CAG-A53T) in the MS.

➔ **We now added viral vector details on lines 1044-1046 in the Methods section.**

2.2. Data organisation and presentation
: The MS introduces the A53T and EV groups early (lines 91-97), but clear validation data for the model are not presented until Fig. 3. I suggest considering the re-organisation of some data so that the model validation is clearly delineated before proceeding to subsequent experiments in the study.

➔ **We thank this reviewer for suggesting optimization of our sequential presentation of results. In the results section, we now first clearly present full PD model validation data in lines 197-220 and 233-255 (see Supplementary Fig. 6 and 7). In order to achieve this we moved model validation figures into the supplementary part. In the Results section, we now show the viral tracing (including the newly added retrograde rabies tracing) first, then model validation, before moving into the optogenetic experiments and various behavioural results.**

2.3. Group descriptions
: there are only a few descriptions of the A53T group in certain sections (e.g. lines 100, 110-112), with limited supporting data. Consider separating the A53T data from Fig.1, which focuses on optogenetic titration and vestibular symptomatology (VS), or clearly provide or indicate the relevant data for this group.

➔ **We follow the reviewer's advice (also in other comments) for pooling of data in certain figures (especially former Figure 1). We now separated titration data for A53T-ChRmine and EV-ChRmine (see Figure 3 and Supplementary Figure 11). In addition, we added the presence of VSS 1-4 in A53T vs. EV data in the Results section (Figure 3 and also especially Figure 4), which was a very valuable hint, since it made us realize indeed why the vestibular threshold stimulation might have worked – the PD animals were optically less responsive vs. the healthy mice when looking at the overall thresholds indicating some sort of vestibular hypo-/dysfunction.**

3. Titration of optomodulation and VVS scaling
3.1. Data presentation and grouping
: The results describing titration of optomoudlation and the scaling of VS are intriguing, as 4 distinct VSs are observed with increasing laser power thresholds. However, the data presentation is confusing and potentially misleading. Data appear to be mixed from different experimental groups (VN-ChR [VN-ChR-SNc-EV], PD [VN-ChR-SNc-A53T], and optical control [VN-mCh]) and time points without clear separation or statistical justification. I recommend that the authors first present the results for the VN-ChR group separately (e.g., Fig 1. a-f or a-j) and

then provide separate or statistically compared data for the PD group (e.g. Fig. 1g-k or k). This separation will clarify the origin of each dataset and the interpretation of the results.

- ➔ **We appreciate pointing out this issue in data presentation and grouping. We rearranged and clarified former Figure 1 (now Figure 3) by showing the results for titration data separately for the EV-ChRmine and A53T-ChRmine group described on lines 256-315. We now show the clustering and embedding results annotated separately for each group to provide distinction and statistically compare those results (see Figure 4) as seen in the Results section under lines 256-315 (see Supplementary Figure 11 as well).**

3.2. Light conditions and thresholds
: The MS does not clearly describe the light conditions used in the titration experiments. Specifically, it is unclear what the exact light intensities are for each VVS to appear (i.e. the threshold values). It seems necessary to revise Fig. 1c,d (clarify whether the y-axis represents laser power and whether two stimulation cycles are applied during the 10-minute total duration. Additionally, please specify if the different VSs correspond to the first observation of the VVS at distinct light intensities or if these are the most prominent behaviours at those intensities). I suggest including a table that lists the light intensities at which each distinct VVS first appears, along with the mean +/- SEM (if differ for each mouse) and the probability (or frequency) of each VVS observed across the range of intensities (would be 4 in total light powers, using EV group only). If these experiments were performed across the entire optogenetic cohort (both EV and A53T), please state for each group separately and statistically compare the groups (not just stating in lines 110-112) at fixed time point (i.e. 8-wk).

- ➔ **We now provide the exact light conditions used for both titration and perithreshold experiments (see Methods section under Lines 1052-1096 and Supplementary Tables 5, 6) and have analysed and compared these data (see Figures 3,4) between groups and longitudinally. Regarding the table the reviewer suggested, we rather solved that with the panels we provide under Fig. 4.**

3.3. Ordering and reference to prior studies
: The current ordering of panels is somewhat confusing – for example, Fig. 1c appears before the authors define what the VVSs are. It may be more logical to first present the reference data from a previous study (e.g. PMID: 34171443), which demonstrates that optogenetic activations (ChR) of Vglu2+ VN neurons induces vestibular disfunctions, followed by the pilot titration results and the aforementioned table. This would help contextualise the observed VSSs and experimental conditions.

- ➔ **As stated above, we have logically rearranged figures and their corresponding panels and also clearly reference the aforementioned work (see Line 268) when first presenting these findings and our vestibular symptom scale (VSS). We hope that this sufficiently solves the issue.**

3.4. Interpulsed light protocol
: The authors used the interpulsed periods of 30-40 s during titration experiments; however, it is unclear whether these intervals are sufficient for the vestibular system to fully return to its baseline (pre-stimulated) state. Although these intervals might reset ChRmine photocycle and the membrane potential, the recovery of the resting excitability balance of the ipsilateral and contralateral VN neurons may require a longer period. In a comparable study (PMID: 34171443), transient unilateral optogenetic stimulation (20 Hz, 5 ms for 30 s) of Vglu2+ VN neurons induced posture-locomotor deficits that persisted for up to 5 min pose-stimulation (some minutes for the VVS2). This raises the possibility that the short intervals in the current study might lead to cumulative effects, where residual activation influences the subsequent responses. It is unclear whether the different VVSs result solely from incremental light powers, repetitive stimulation or accumulation (as discussed in lines 149-150), the total duration or mixed effects (e.g. the observation of the VVS3 after VSS1 could have resulted from the prolonged stimulation, as discussed in lines 479-480). Thus, I suggest performing the 4 titrated light conditions (mean power for each VVS threshold) from their pilot experiments as separate groups (for each group using a constant light

power at 20 Hz, 10 ms pulse duration at 30-40 s intervals for 10 min) to confirm that the light intensity alone is responsible for eliciting distinct VSS thresholds.

➔ **We thank the reviewer for pointing out this issue. We show that for our experiments, the postural disruptions were temporary and more or less quickly reversible as noted in the Results section under Lines 280-283 (see Supplementary Fig. 9). The differences in comparison with the other study (PMID: 34171443) might lie in the different pulse widths and 3x duration (30s vs. 10s) which we believe might account for this difference when looking at the experiments of Montardy et al., which also used a different optical actuator. The 3x duration of the stimulation likely has a longer lingering effect on the mice. Beyond the scope of our study, systematic experiments with in-vitro electrophysiology, calcium imaging or specifically a combination of in-vivo calcium imaging and optogenetics could further clarify this question.**

3.5. Mechanistical considerations
: it remains unclear whether the graded VVS responses result from an increased number of Vglut2+ VN neurons being activated, increased spike probabilities, or both. Additional experiments – such as quantifying cFos expression at each light intensity (compared to the VN-mCh control group) or electrophysiology recordings – would help elucidate the underlying mechanisms.

➔ **We are grateful for the reviewer's concern regarding mechanistic insight. To address this point, we have performed an additional experiment (see Supplementary Fig. 12). We did not find that the VSS correlates with stronger cFos expression, data now presented in the Results section in lines 316-325. As the reviewer suggested, a future study (see above comments) using electrophysiological approaches could further elucidate that matter. Yet, this was not the primary focus of our work, but rather the translational investigation of the VNC in PD mice as a potential neuromodulatory/therapeutic target and the underlying circuitry.**

4. Semi-supervised ML and classifier verification
4.1. Training vs prediction data
: The MS employs a semi-supervised ML approach with UMAP analysis, using 4-wk data for training and applying the trained classifier to predict behaviour at 8-wk. could the authors clarify the rationale behind using 4-wk data for training, particularly given that the 4-wk time point appears suddenly in the text? A clear explanation is needed regarding why this time point was chosen for training.

➔ **We thank the reviewer for raising the methodological point. We retrained the classifier again. Initially the classifier was trained on snippet videos (short, ~10s) of week 4 titration videos. It is now trained on randomly chosen full videos of titration experiments, perithreshold experiments and off recordings from week 4 and 8 and also snippet videos of VSS symptomatology from week 4 and week 8. We then applied the classifier to both, naturalistic behaviour and vestibular symptomatology across the entirety of week 4 and week 8 videos and report this in the Results section in lines 284-315 (see the now Supplementary Fig. 10 again for workflow details, previously part of Suppl. Fig. 2) and see Methods section, lines 1272-1343 for details on**

methodology and training dataset. The classifier was trained so that it performed well on naturalistic behaviour and vestibular symptomatology recognition (hence the addition of snippet videos of VSS symptomatology to better balance the training dataset).

4.2. Source of training data
: In Sup. Fig.2, the legend does not specify which group(s) of mice contributed the training data (and the number of mice involved). Additionally, it is unclear whether the data from mice used in training were excluded from the 8-wk prediction analysis. Please clarify the origin and separation of training vs prediction datasets to ensure transparency and to rule out potential bias and overfitting.

- ➔ **In our retrained classifier, EV-ChRmine and A53T-ChRmine mice contributed both equally to training this classifier; we show the number of videos that went into training and mice corresponding to this (Line 1276-1281). We set the test/train split rigorously. Training at 10%, Testing at 90%. Potential bias and risk of overfitting thus seem low to us.**

4.3. UMAP visualisation
: although the statistical analysis (in the legend) indicate that the clusters are significantly different, the UMAP visualisation in Fig. 1e does not clearly display distinct clusters. I suggest exploring alternative dimensional reduction methods and adjusting the colour schemes to improve the visual distinctiveness of the clusters.

- ➔ **Thanks to the reviewer for raising this issue on the UMAPs. Since we have reused this dimensionality reduction technique implemented with BSoiD (PMID: 34465784) inside ASoiD (PMID: 38383746), but have both adjusted and optimized our embedding process/group separation and statistical analysis, we also have adjusted the colour schemes respectively (see Fig. 3 and Supplementary Fig. 11) in order to provide visual distinctiveness.**

4.4. Prediction accuracy across groups
: Were there any differences in prediction accuracy between the A53T and EV groups? This information is critical to assess whether the classifier performs equally well across different experimental conditions as abnormal mice often are not well classified.

- ➔ **We understand the reviewer's concern, which is linked to the other points raised above. In fact, the classifier was now (re-)trained and performance testing done on a dataset of both A53T-ChRmine and EV-ChRmine data (90% from training data according to test/train split). We primarily tested the performance on the behavioural categories by calculating F1 scores. Since the training dataset was well balanced, there is no hint towards a different performance since if A53T-ChRmine predictions would work poorly, the classifier would never have reached high performance values/generalisability. A different possibility would have been to train two separate classifiers, however that raises a whole range of (in our humble opinion) bigger problems such as higher likelihood for overinterpretation, bias and hence falsified results, since one would have to compare the results of two different classifiers and would run into various statistical issues and interpretational issues when comparing A53T-ChRmine vs. EV-ChRmine. To the best of our**

knowledge, we see no reason to assume the classifier works worse for one group or the other according to the balanced training dataset. Last but not least, the biggest problem here is sample size. With the sample sizes we have, training a classifier for each group separately would probably have yielded poor performance (we initially tried this for a bit) for both classifiers (if training one separately for EV-ChRmine and A53T-ChRmine each).

4.5. Presentation of classification results
: For Sup. Fig. 2d, the current format is not very readable. I recommend presenting the data in a table that details the percentages of human vs machine classifications for each VSS, separately for the EV and A53T groups.

➔ **We believe that this is a visually intuitive way (alluvial-like plot) to present matching (in this case of detection of a VSS symptom class). Our idea of showing this was for it to be immediately clear, that of all recognized VSS, the classifier only did not recognize VSS 1 presence in one video/animal at week 8.**

5. Fig. 1g-k, are the VVS defined by human or predicted? It is confusing because in the previous panel they just verified the ML method for the prediction of VVSs. In logical thinking, one might expect they had done so to use the ML predictions for the later studies (Fig. 1g-k). Please clarify the origin of these labels to unsure methodological transparency.

➔ **We understand the reviewer's request for clarifying this point. The VSS is human-defined since the symptoms are what we (subjectively) observed and thus wanted to somehow show systematically via a semiquantitative scaling system. The reason for utilizing a ML approach was the unbiased nature of pose estimation + clustering + embedding with the primary goal of seeing whether the pose itself would reveal subtypes of vestibular behavioural changes. (see in Results section under lines 284-315). In the new Fig. 4, which presents the results in reoriented and updated fashion, we show the VSS label and for the clustering analyses, we clearly show the clusters based on pose and ML as "cluster 1" etc.**

6. Clarification on VVS labelling
6.1. The MS does not clearly indicate whether the VVS labels shown in Fig. 1g-k were defined by human raters or generated by the ML predictions. In the previous panel, the ML method was validated for predicting VVSs, so it is not immediately clear why the subsequent data do not explicitly state the origin of these labels. Please clarify whether the labels used in Fig. 1g-k are based on human or machine.

➔ **We have addressed this comment in the preceding paragraph.**

7. Fig. 1k – Longitudinal data and interpretation
7.1. Time points and longitudinal design
: the data is described as 'longitudinal,' yet only two time points are shown: the baseline (1-wk after surgery) and 8-wk. For a robust longitudinal study, I recommend including at least one intermediate time point (e.g. 4-wk), as that data at this interval are available. Given that A53T model mice display complex behavioural changes as PD progresses, which is not always unidirectional or has a time threshold for a certain parameter to change, reflecting the dynamics of the pathological changes. Thus, additional time points would better capture these dynamic progressions of the pathology.

➔ **We totally agree with the reviewer on this point and thank him/her for bringing this up. Hence, we ran additional independent cohorts of A53T-Wildtype and EV-Wildtype mice, which did not show differences in major disease pathology (SN, striatum) or behaviour. Consequently, (and due to other comments by this reviewer regarding clarity of defined time points) we chose to focus on week 8 for our core results (open field, gait data) to avoid overly complicating the statistical comparisons by adding additional conditions. In addition, we now added week 4 data to the titration experiments/threshold analyses, where this appears especially crucial to us vs. in the model-related open field & gait data, for which model (A53T) we show no significant differences between week 4 and week 8.**

7.2. Interpretation of the data
: The MS lacks a clear interpretation of the longitudinal findings. For example, is the observed decreased sensitivity in the PD group (noting that the A53T and EV groups are currently mixed) attributable to alterations in PD circuitry, such as increased glutamatergic receptor expression in VN neurons (PMID: 36347898)? A brief interpretation of the result would enhance the study's impact.

➔ **We thank the reviewer for bringing this interesting study to our attention. Looking at the updated threshold findings (see current Fig. 4), we now discussed this further (see Discussion section on lines 632-634).**

7.3. Baseline consideration
: The term 'baseline' is used to describe data from 1-wk post-surgery. However, the current data indicate optogenetic responses at this time, suggesting that the overexpressed A53T also may have already impacted the neural circuit at some degree (the largely skewed rotatory symmetric index of A53T mice at BL in Fig. 3i might represent a minor onset state). Strictly, it seems inappropriate to state this time point as 'presymptomatic time point' in line 739. The term 'baseline' should represent pre-surgical condition; otherwise, this time point should be referred to as '1-wk' or 'Week 1' to avoid confusion.

➔ **We are grateful for the reviewer for noting this discrepancy. This has been an error on our side on former Fig. 1k (now on Figure 3). Here, we falsely annotated "baseline" when in fact "week 4" was meant. We did not conduct titration testing at baseline/week 1 (since of course it was not to be expected to have viral expression at that time). We have changed baseline to week 1 in all figures and in the main text where applicable.**

7.4. Group comparisons
: it would be beneficial if the authors compare A53T vs EV at 8 wk (or for both groups, 1-wk and 8-wk) to determine whether differences in the optomodulation of VN neurons exist between the groups.

➔ **This has been done now consistently (also see your comment on this general matter in 7.1).**

8. Perithreshold stimulation condition
8.1. Light power determination
: It is unclear whether the light power for perithreshold stimulation was determined individually for each mouse or derived from an average value of previous results (lines 147-149). Please clarify this point and specify the exact light power used (e.g. in mW at the fibre tip).

➔ **We have clarified this matter in methods (Line 1079-1096). Also see Fig. 4a,b for exact mW values for each mouse per group for each VSS. And see Fig. 4f,g for exact mW values for each mouse per group for the perithreshold condition.**

8.2. Translation of ‘1 mA below threshold’
: The MS states that stimulation was set at ‘1 mA below threshold’ (line 732). Could the authors explain how this current offset translates into light output in mW, and elaborate on the significance of the 1mA difference? Additionally, please clarify how this perithreshold light power differs from the VVS1 threshold (i.e. the light intensity at which the first vestibular symptom is observed).

➔ **We thank the reviewer for raising this point and apologise for any confusion on this matter. In fact, we took a 10mA online reduction (instead of the false 1mA statement previously, for which we missed a 0) of the VSS 1 power per mouse after titrating the individual VSS1 threshold. We elaborated this in detail in the Methods section (Line 1079-1096). This yielded approximately 1-2 uW lower laser power at fibre tip vs. the VSS1 condition per mouse.**

8.3. Mechanistical considerations
: To better elucidate the mechanism underlying perithrhold stimulation, I recommend that the authors compare the cFos analysis under this condition (Fig. 2q VNC data; this seems better to come here for the verification of optogenetic conditions), alongside the other conditions (discussed above). Comparing the number and distribution of activated neuronal population between the perithrehold and VVS1 threshold conditions would provide valuable mechanistic insight.

➔ **According to the reviewer’s point here, we have now run an additional experiment to see whether cFos expression in the VNC is different under 4 different conditions (sham, perithreshold, VSS2 and VSS4). It appears that while verum stimulation leads to increased VNC cFos activation, there is no difference between PT, VSS2 and VSS4, suggesting mechanisms primarily independent of crude cell recruitment. We show these data on Supplementary Fig. 12.**

9. Fig. 1 – Data segregation and group comparisons
9.1. The MS presents data from different experimental groups (EV and A53T), yet these data are mixed without clear separation or statistical comparisons. Additionally, data from the VN-mCh control group are not explicitly provided. Prior work (e.g. PMID: 36347898) has shown that glutamatergic receptor compositions in the VN can differ in PD models, suggesting that the A53T PD mouse model may exhibit distinct pathophysiological changes that affect VN neurons. Without showing the data from the EV and A53T groups separately and providing appropriate statistical comparisons, it is difficult to justify pooling them as a single optogenetic cohort. I strongly recommend that the authors clearly segregate the data between the different groups (and include the VN-mCh control data) and provide statistical comparisons to address any group-specific effects. This clarification is essential for accurate interpretation of the results and for ensuring methodological transparency.

➔ **The reviewer is absolutely right in this regard. Our initial rationale for pooling for the titration data presentation was that we didn’t see any clear differences in the VSS responses of A53T vs. EV. But now we understand from the clustered pose-behavioural data (see current Fig. 3 and Supplementary Fig. 11) that there appear to be differences. For all our data, we now clearly**

separated A53T from EV, specifically for A53T-ChRmine vs. EV-ChRmine and have compared these in all relevant results against each other.

10. Structural and functional mapping of VN connections
10.1. Connectivity mapping study
: the authors performed anatomical connectivity studies using viral tracings in normal mice and reported laterality in the VN targets (Fig. 2a-c). However, the MS lacks quantitative and statistical analysis of these data. Terms such as 'sparse' and 'dense' are used to describe projections, but it is unclear how these are defined or measured. I recommend that the authors provide numerical descriptions and statistical comparisons for the connectivity patterns (e.g. quantified metrics in the method section).

- ➔ **We now provide a fully new cohort of brain-wide quantification of vestibular projections by the QUINT pipeline, which has allowed for detailed, atlas region-based quantification of synaptic density in projection regions. (see current Fig. 1., also Supplementary Fig. 2-3).**

10.2. Functional mapping (cFos) study
: the authors use deepflash2 to segment and quantify cFos expression in 526 images from key regions (VNC, Gi, Pf and ZI), and they report that the data are pooled from both hemispheres for analysis. Pooling assumes no significant differences between the ipsilateral and contralateral sides. However, the anatomical tracing data clearly reveal laterality in connectivity (Fig. 2c). This raises several questions: (1) what is the justification for pooling hemispheric data in the cFos analysis? (2) were there no significant differences between the two sides in the analysed regions? (3) would reporting differences (or the lack thereof) between the ipsilateral and contralateral VN provide insights into the robust bilateral communication in the VN, which might underlie balanced integration of vestibular information and potentially contribute to the therapeutic effects of nGVS or perithreshold stimulation? Considering that the optogenetic stimulation was applied to unilateral VN – and given that PD may further disrupt network balance – it is essential to assess whether any asymmetries exist in cFos activation. I recommend that the authors analyse and report cFos counts separately for the ipsilateral and contralateral sides (or as percentages relative to a control side) before pooling. This approach would clarify whether unilateral VN stimulation results in balanced activation across hemispheres or if significant differences exist that could impact the interpretation of the comparisons between the EV, A53T mCh groups (Fig. 2q-t).

- ➔ **We thank the reviewer for this essential comment. We now provide the data per hemisphere for each animal from each of the 3 groups. For hemisphere-region combinations, the significances are thus lower, but the overall result remains the same (see current Fig. 5 f-i).**

11. A53T PD mouse model (part 2)
11.1. Comparison to previous study
: the authors modified their model generation protocol compared to their previous work (PMID: 28143577). Notably, the viral load used in this study (15 x 10E+12 gp/mL, 0.5 uL) is approximately 4 times higher than in their previous study (7.74 x 10E+12 gp/mL, 1.5 uL). Assuming the viral construct is the same (AAV1/2-CAG-A53T), it is surprising that the TH+ cell loss in the SNc in this study (~43% at 8-wk) is only modestly increased compared to the previous study (~33% at 10-wk) as a higher viral load would typically lead to a greater degree of DA loss. This discrepancy raises questions about the injection method (DV-serial injections) and site – Fig. 3a suggest it may be too medial – and whether the higher dose was effectively delivered to the target region. Additional explanation regarding these points seems critical for evaluating the robustness and comparability of the PD model.

- ➔ **We thank the reviewer for point this out. However, there is a misunderstanding in this statement. Previous work that you referenced (PMID: 28143577) used a viral titre of 5.16 × 10^{12} gp/mL at a volume of 1.5 µL, not 7.74 × 10^{12} gp/mL at a volume of 1.5 µL. In fact, when comparing the viral genomes delivered then:**

Condition A (previous study)

5.16 × 10^{12} gp/mL, 1.5 µL

$$\mathbf{1.5\ \mu L = 1.5 \times 10^{-3}\ mL}$$

$$\mathbf{5.16 \times 10^{12} \times 1.5 \times 10^{-3} = 7.74 \times 10^{9}}\ \text{gp}$$

Condition B (current study)

15 × 10^{12} gp/mL, 0.5 µL

$$\mathbf{0.5\ \mu L = 5 \times 10^{-4}\ mL}$$

$$\mathbf{15 \times 10^{12} \times 5 \times 10^{-4} = 7.5 \times 10^{9}}\ \text{gp}$$

Comparison

- **Condition A: 7.74 × 10^9 gp**
- **Condition B: 7.50 × 10^9 gp**

Difference

- **Absolute difference:**

$$\mathbf{7.74 - 7.50 = 0.24 \times 10^{9} = 2.4 \times 10^{8}}\ \text{gp}$$

- **Relative difference:**

$$\mathbf{\frac{7.74}{7.50} \approx 1.032}\quad (\approx 3.2\%)$$

These studies are thus essentially matched in viral load. The difference is only ~3%, which seems biologically negligible compared to typical variability in AAV prep, injection loss, spread, and expression efficiency to us.

Yet, our higher concentrated viral solution (that still delivered higher titrated viral load) led to more severe neurodegeneration (~50% OD in striatum vs. EV and ~60% vs EV in TH cell count in SN). This might be due to the higher titre of the virus, even though the overall viral genomes/particles delivered was pretty much the same. We again validated the viral titre and

quantity and thus amount of viral genomes used at both week 4 and week 8 in order to address these questions. The SN cell reduction is about the same in our new week 8 cohort of Wildtype mice (A53T-Wildtype) vs. the A53T-ChRmine mice and the amounts used were the same. We think that the representative image we chose (in previous Fig. 3) maybe was not that well representative after all. We thus chose a new image (see Supplementary Fig. 7) – compare with A53T-Wildtype pattern as well as seen via Supplementary Fig. 6 – the IHC patterns were very consistent.

11.2. Histological validation
: The histological validation is limited to 8-wk IHC images (Fig. 3a,c), even though the authors claim that TH+ cell counts are similar at 4- and 8-wk (Fig. 3b). To substantiate this claim, I recommend that the authors provide: (1) representative images and quantitative analyses of the TH fibre intensity (or complexity) in the striatum at both 4- and 8-wk; (2) SNc images from the 4-wk time point; (3) comparative data (e.g. percentages) between the ipsilateral and the contralateral sides. Additionally, please label the groups clearly (Fig. 3a,c) and, if possible, provide images for each channel separately for Fig. 3a, where the extent of DA degeneration is not clearly visible, along with clear indications of the corresponding areas in enlarged images in Fig. 3c.

- ➔ **We ran 4 additional cohorts of mice and now provide the data requested by the reviewer in Supplementary Fig. 6. With stereology we compare against the ipsilateral side of healthy (EV) animals. Previous studies on the model have identically used ipsilateral quantification with comparison against the EV group as well (PMID: 28143577).**

11.3. Control group
: It would be beneficial to include a control protein overexpression group (e.g. AAV1/2-CAG-GFP) because high protein overloading is known to damage DA neurons in the SNc, (as discussed in previous studies, e.g. PMID: 28143577; PMID: 21029459). Given that the injection protocol has been optimised to deliver a high viral load, including such a control is essential for distinguishing the effects of the A53T mutation from potential protein overloading effects.

- ➔ **We agree with the reviewer that overexpression of control proteins such as GFP can, in principle, exert toxic effects on SNc dopaminergic neurons, as previously reported (PMID: 21029459). Importantly, however, the relevant control experiments have already been performed under comparable conditions and with matched viral particle load. In that prior work, GFP overexpression produced at most a modest reduction in SNc dopaminergic cell counts—approximately half of the magnitude observed with A53T-αSyn—did not induce dystrophic neurites, and, critically, did not lead to nigrostriatal dopaminergic denervation as assessed by striatal optical density. Moreover, our current injection protocol delivers the same number of viral particles, ensuring comparability in terms of viral load and expression burden. Given these existing data, we believe that repeating an AAV1/2-CAG-GFP control group in the present study would be redundant and unlikely to alter the interpretation, while our conclusions remain specifically attributable to A53T-αSyn rather than non-specific protein overloading.**

11.4. Data pooling concerns
: The authors state in lines 256-257 and 740-741 that they pooled 4-wk and 8-wk data for behavioural analysis, claiming that 'an independent cohort of A53T mice, euthanized at week 4 after injection of AAV1/2-A53T showed similar levels of dopaminergic cell loss'. However, overexpression of aSyn (A53T) is known to produce gradual pathophysiological and behavioural changes over time (PMID: 24267638; PMID: 37463889). The discussion (lines 521-523) also notes that the A53T model as 'mimicking the progressive course of PD', suggesting that pathological states at 4- and 8-wk might differ. If data from these time points are to be pooled, the authors should provide: (1) the histological validation demonstrating no significant differences between the 4- and 8-wk time points, (particularly in the striatum); (2) behavioural data showing comparable outcomes at both time points. Otherwise, it may be more appropriate to select a single, well-defined time point for analysis.

➔ **As stated before, we have removed the data pooling for several reasons, including that the histological data from week 4 and week 8 seem almost identical (see Supplementary Fig. 6). We thus selected a single, well-defined time point for most of our analyses (week 8).**

11.5. Behavioural data timeline
: It appears that most behavioural tests were performed at 8-wk, yet the data pooling raises concern regarding proper labelling. Please ensure that the data are accurately labelled (e.g. 'Week 8 off' should not include 'Week 4' data). A schematic diagram outlining the experimental timeline for the behavioural testes would be helpful if multiple time points are considered.

➔ **We have removed the pooling and made sure that accurate labels of week and conditionalities are always available.**

11.6. Cylinder test
: Aside from the baseline concerns (see, comment 7.3), the authors describe the effect of perithreshold optostimulation (PT) as a 'mild reduction' in the asymmetric rotation. Please provide quantitative details on the magnitude of this reduction and indicate whether the OFF and PT conditions differ significantly via t-tests. It may also help to reorganise the figure panels (j, k and i) so that data are presented in a logical sequence.

➔ **We have provided a % regarding rotation asymmetry reduction and have shown statistical comparisons. See line 252 and current Supplementary Fig. 7.**

11.7. Perithreshold group in treadmill test
: the MS presents PT group only for the cylinder test (Fig. 3i) but not for the treadmill test (Fig. 3j,k). Clarification is needed as to whether the treadmill test was not performed for the PT group or if the results did not differ from the OFF state. This information is important for understanding the full impact of PT on motor function.

➔ **We thank the reviewer for this comment. We have provided these data under Fig. 7b.**

12. Pose estimation and behavioural analysis in OF
12.1. Data pooling and experimental time points
: the authors pool data from different time points (4-wk and 8-wk) for OFF and PT conditions for the EV and A53T groups. Notably, the timeline for the mCh (lines 744-746) does not match that of the experimental groups (OFF at 2-wk and PT at 3-wk). To ensure robust and comparable analyses, the authors should segregate the data for each group at each time point separately and align the experimental timelines across groups. Note, the latter is important especially for repetitive behavioural experiments in OF (lines 369-373).

➔ **As stated above we have removed the data pooling and present aligned data from baseline (week 1), week 8 (under off and perithreshold condition; additionally, titration condition for A53T-ChRmine and EV-ChRmine, since only those had vestibular symptoms upon stimulation) for A53T-ChRmine, EV-ChRmine and sham mCherry mice. See Fig. 6.**

12.2. Pipeline schematic for the analysis workflow
: fig. 4a occupies considerable space but lacks detailed information about the behavioural pipeline. A comprehensive schematic outlining the entire process would help readers understand the methods: from video capture, keypoint extraction using DeepLabCut (with retained RCNN model), through to custom post-processing and behavioural classification with Keypoint-MoSeq; besides, details on dataset compositions, tracking accuracy metrics marked in the schematic.

- ➔ **We have provided a detailed explanation on this in the Methods section on lines 1182 to 1198 and then 1228 to 1270** (see **Supplementary Table 2 for settings/modelling hyperparameters).**

12.3. Syllable presentation and organisation
: In Fig. 4b, only a subset of syllables with behavioural labels is shown, and the criteria for their selection remain unclear. I recommend that the authors organise all syllables hierarchically – ideally in a vertical dendrogram (Fig. 4c) – that includes the syllable number, the corresponding supervised behavioural labels, and key kinematic metrics (e.g. mean velocities) displayed via a heatmap. If space is allowed, representative images of each behavioural motif could be provided be included (Sub. Fig. 3). This would facilitate a more meaningful interpretation of the data.

- ➔ **We have rerun the KPMS model for reasons stated before (data pooling) and concerns raised under 12.4 and 12.5. We have thus chosen not to include in-syllable statistics but rather use kpms only for a bird's eye view of behavioural (transition) structure.**

12.4. Locomotor performance
: in lines 348-350, the authors report that PT stimulation enhanced the peak locomotor performance in VN-ChR groups (EV and A53T), as indicated by increased mean velocity and standard deviation (SD) of velocity within the running syllable (line 348-350, Fig. 4h and Ext. Fig. 2a). However, the data are confusing. There appears to be a significant difference in the OFF state between the VN-ChR groups and the mCh group (elevated relative to mCh group), yet the magnitude of the change from OFF to PT seems similar across all groups. Additionally, the mCh group's OFF to PT difference is reported as non-significant, but the absolute difference and large error bars in the PT state are concerning – while the SD of velocity for this group at PT shows much smaller error bars. It is unclear how the mean and SD of velocity within the running syllable were derived and normalised. Please provide more explanation of these calculations the p value for mCh group in Fig. 4h.

- ➔ **As stated above under 12.3, we have decided not to use the in-syllable kinematics anymore and instead use a measure as rigorous as possible for performance, namely motion measure by contour tracking vs. capacity (from keypoint distance moved). We rather use KPMS and thus behavioural syllable analysis only for a bird's eye view of behavioural (transition) structure.**

12.5. Additional concerns
: fig. 4j, why OFF states of the EV and A53T groups are already elevated compared to mCh? Fig. 4i, the mCh group's running duration under PT appears increased, despite the authors reporting a non-significant difference (p = 17). Fig. 4j, baseline differences between the optogenetic cohort and mCh are apparent, suggesting possible light-independent effect of ChR overexpression in VN. The authors should clarify these discrepancies by providing complete statistical comparisons (otherwise set the true baseline). Fig. 4j, the data for mCh group at OFF condition are missing, which limits interpretation in lines 372-373. Lastly, similar patterns of changes from OFF to PT across all groups (despite authors claim those are ns) raise the possibility that the PT condition itself might have no-specific effects (ChR-independent), possibly due to the PT experimental condition.

- ➔ **We have fully provided the kinematics data in Fig. 6c, d.**

13. Gait dynamics study
13.1. Figure number
: in line 413-453, Fig. 5 seems mislabelled as Fig. 6, please correct the number.

➔ **We have corrected the mislabeling.**

13.2. Maximal velocity
: In line 417-418, I recommend including the mean values for the comparisons.

➔ **Mean values are now presented in the results section in lines 547-548**

13.3. Suprathreshold light condition
: Please clearly state the exact light power (VSS2-3 threshold). Was a consistent light power used across subjects in all groups or differ to each mouse?

➔ **For suprathreshold stimulation on the treadmill, the VSS3 threshold/animal was used. We provide the exact values in the methods section: "For suprathreshold stimulation on the treadmill, in order to assess the specificity of the perithreshold condition and demonstrating its gait impairing effects, we titrated the individual VSS3 threshold for each subject (i.e. mouse) before the suprathreshold experiment and used these individual thresholds. (A53T-ChRmine: 33,92 ± 7,958 µW, EV-ChRmine: 23,33 ± 4,978 µW; mean ± s.e.m.)".**

13.4. Fluidity of gait index (GFI)
: in lines 427-429, the authors report that PT stimulation does not alter gait (i.e. gait failure is not increased), whilst suprathreshold stimulation markedly enhanced gait failure for PD mice. In Fig. 5c, the data for the PD group suggests that the percentage of gait failure under PT condition is less than 1% of the total trial, which seems surprisingly low given that PD mice typically exhibit impaired gait. One would expect the PD group to display a lower FGI and more gait failure than EV group at least in the OFF condition. I recommend that the authors present the mean FGF values and the percentage of gait failures for both EV and A53T groups under OFF, PT, and suprathreshold conditions. Additionally, please provide a more detailed description of the FGI definition, particularly how gait interruptions are determined. This additional information would greatly improve the interpretability and reproducibility of the results.

➔ **We thank the reviewer for this comment. We describe the FGI scoring system in detail in the Methods section on lines 1164-1181. We added detail on description of how interruptions were determined. We also included data from W1, W8-OFF, W8-PT and W8-Suprathreshold on Fig. 7c,d. Since the phenotype of the A53T vs. e.g. 6-OHDA mice is not that severe with regards to hypokinesia, there is no massive gait failure of W1 vs. W8-OFF.**

13.5. Oscillatory gait pattern analysis
: the dual-view video recordings are captured at 30 fps (line 815), which implies a maximum resolvable frequency of 15 Hz according to the Nyquist theorem. However, the scalogram's y-axis is labelled from 0-30 Hz. The authors should clarify whether this is a labelling error, or if the data were resampled or processed in a way that extended the frequency range – this is not clearly described in the methods and appears inconsistent with standard signal processing principles. Additionally, it would be beneficial if the authors provided the corresponding frequencies (in Hz) for the peak powers observed (Fig. 5g-I, in lines 438-440) to aid in interpreting the gait dynamics. Furthermore, the MS would benefit from more detailed description of how autocorrelations were computed. As a minor suggestion, I recommend re-organising the panels in Ext. fig. 3 by placing the scalograms on left, so that the order aligns with the presentation in the main Fig.5 and the text.

➔ **We thank the reviewer for this detailed comment regarding some confusion of the gait video processing. In fact, the dual-view videos (see Supplementary Video 6) were all 30fps/Hz resampled videos as automatically generated by the DigiGait® software from the singular lateral**

and ventral view videos (which were recorded at 165fps/hz each) original frequency. Hence, there is no error in the scalograms, but we unfortunately didn't elaborate this enough and apologize for that. We have described this in a more detailed fashion in the Methods section on lines 1148-1153. We added the peak frequency range these effects take place at (3-4 Hz) on line 571 in Results. We elaborated on the computation of the autocorrelograms (line 1211 onwards). The panels from former EDF. 3 (now Supplementary Fig. 14) were re-organized in line with former main Fig. 5 (now Fig. 7).

Reviewer #2 (Remarks to the Author):

This very interesting, original, and timely study by Hartig and collaborators presents the first detailed investigation in parkinsonian mice into the effects of optogenetic stimulation of glutamatergic (Vglut2+) neurons in the vestibular nucleus complex, a promising therapeutic target for improving motor function in Parkinson's disease. While little is known about the mechanisms and motor effects of stimulating the vestibular circuitry, which has been gaining clinical interest, the authors make a significant and original contribution by filling this important knowledge gap. Notably, the results show that stimulation can improve locomotor performance under optimal conditions. The study is admirable for its novelty, high resolution, attention to detail, and its relevance to both the motor control and clinical fields.

Strengths:

1. New target for Parkinson's disease: This study is the first to investigate the potential effects of this new target in such detail. The study benefits from the authors' expertise, as they are leaders in both experimental models of Parkinson's disease and the clinical application of deep brain stimulation, which enriches the study's depth and clinical relevance.
2. Original, high-resolution, unbiased analysis of motor behavior: The authors show that optogenetic stimulation of the vestibular nucleus complex can have a nuanced impact on motor behaviors, depending on stimulation strength. Using advanced deep learning-based movement analysis, they classified behavior into short, identifiable coordination patterns called syllables and identified transition patterns between them. They also examined whether specific transitions were induced or if stimulations had particular effects on behavioral transitions between syllables. This high-resolution approach is impressive and offers valuable insights into how stimulation may affect movement at a finer level. The study also explores the effects of gradual stimulation, providing a comprehensive description of possible motor effects as a function of stimulation strength, thus

creating a “behavioral atlas” of potential outcomes with direct clinical relevance.

3. The Parkinson’s model: The model used in this study involved intra-substantia nigra injections of an adeno-associated virus encoding the mutated A53T version of α-synuclein. This model, previously characterized by some of the authors, successfully recapitulates key features of Parkinson’s disease, such as chronic dopamine depletion and abnormal motor behavior within 8 weeks, allowing the authors to test preclinical interventions.

4. Value to both clinical and fundamental research: The findings are valuable not only to the clinical field but also to fundamental research, offering unprecedented, detailed behavioral analyses of the target region, which are of great interest to the motor control community.

5. Foundation to study the pathway involved: The authors use functional neuroanatomy (c-fos expression) to identify specific brain areas activated by the stimulation, laying a solid foundation for future research into the underlying pathways involved.

6. Timeliness and importance: This research is timely and important, providing critical insights into the complexities of the substrate at an early stage. Such studies are essential for refining stimulation parameters and preventing the premature dismissal of potential targets in human trials due to an incomplete understanding of the cell types or circuits affected by its electrical stimulation. The approach taken by the authors is commendable for its foresight and meticulous attention to detail.

7. The study is well-written, the analysis was carefully performed, and the conclusions are precise and well-balanced. The methodology is sound, and the work meets the expected standards in the field. There are sufficient details in the methods section for the work to be reproducible.

Weaknesses:

No major weaknesses detected. No additional experiments are needed. Only some minor details remain unclear, but these will be very easily clarified by the authors by simply modifying the text and/or figures (see list below).

Details:

Abstract:

• "Improved locomotor performance but not capacity." This phrase is vague and should be explained more clearly for a broader readership.

- ➔ **We adapted the abstract in this regard.**

Introduction:

• L42: When reporting MLR studies, it is important to distinguish between cell type-specific studies in animals that have successfully identified specific populations to stimulate (as in the present study) and clinical studies using

deep brain stimulation (DBS), which likely cannot (yet) target pro-locomotor neurons and have reported mixed results. Acknowledging this difference in the text is crucial to avoid prematurely dismissing the MLR target (or any other brain target) based solely on DBS studies conducted before the circuitry was thoroughly examined in animal models and before the identification of relevant target cell populations in preclinical studies. It could be explicitly mentioned that targeting specific pro-locomotor cell subtypes with DBS remains a challenge. This also provides an likely explanation for the cause of mixed results in the MLR.

➔ **The reviewer's point is well taken and now implemented in lines 43-45. Here, we further contextualize the MLR cellular subtypes in relation to deep brain stimulation.**

• L49: "This technique has shown promise" – Please be more specific about the effects reported.

➔ **We thank the reviewer for this comment and specify this point in lines 54-55. The effects mainly resolve around improvement of postural deficits, less so regarding improvement of gait disorder.**

• L53: "Improve Parkinson's symptoms in rats" – Please clarify which symptoms were improved and how.

➔ **We specify in lines 55-56 that nGVS improved locomotion in parkinsonian rats, possibly via enhanced GABA release by the substantia nigra.**

• "In optogenetic pilot experiments": It would be nice to provide even in the main test, at least once, details about the stimulation parameters (train duration, stimulation frequency, pulse duration) and some basic information, such as how long after stimulation the behavioral effects are observed.

➔ **We thank the reviewer for this comment. We have provided all basic stimulation parameters for all experiments in the methods section on lines 1068-1096. Also see Supplementary Table 5,6.**

• L150: "Yet, repeated stimulation may idiosyncratically change thresholds" – Could you explain this in simpler terms for a wider audience?

➔ **We thank the reviewer for this comment. We adapted the respective language to accommodate a wider audience. See lines 370-371.**

• L183-184: "We took a myc-tagged AAV184 delivered anterograde synaptophysin tracer injected into the VNC" – Please specify whether the injection was unilateral.

➔ **We thank the reviewer for this comment. The injection was done unilaterally on the left side (see Supplementary Fig. 4a), updated in methods on line 940.**

• L262: Rotation on which side? Body turns? Circling? Towards which side? Ipsi or contra to stim? This information is not always provided. Consider improving precision throughout the paper.

- ➔ **This is an important point that the reviewer makes. We added this information consistently to the paper. (see lines 266-273)**

• Concerning limb kinematics, I could not find a raw image of limb kinematics from which the wavelets were extracted. Maybe add a panel somewhere if possible?

- ➔ **We thank the reviewer for this comment. A raw image with keypoint-based predictions is seen on current Figure 7a.**

• L268: "Correlated" – negatively or positively? Could you please specify systematically everywhere in the paper?

- ➔ **We thank the reviewer for this comment. We see the point of adding whether something is correlated positively or negatively. We added this to all instances where this is relevant in statements about correlations throughout the paper.**

• Fig. 4i: It appears strange that the off-PT for mCherry is so high on the right. It is almost significant, but it is not. Is there an explanation for this?

- ➔ **We have excluded the in-syllable kinematics from the paper (see comments from reviewer #1) and rather use KPMS primarily for the overall behavioural (transition) structure.**

• L332: "With perithreshold optoactivation" – Be specific about what “peri” means. 0.75% to 1.25%? Can you provide a more specific range?

- ➔ **We thank the reviewer for this comment. We specified the parameters/idea of the perithreshold stimulation condition on lines 1079 onwards and in Supplementary Table 6 (example).**

• L346: "Bird’s eye view" – Perhaps it would be useful to show the raw kinematics and raw trajectories in some panels.

- ➔ **We thank the reviewer for this comment. Raw open field kinematics are provided on Figure 6c,d and the motion “trajectory” (here, heatmap of motion) is provided as well on Figure 6e,f-**

• L361: "We found that those behaviors bear a covert representation in behavioral modules under low-intensity stimulation conditions." Please clarify.

- ➔ **Not applicable anymore, since we removed the in-syllable kinematics from the paper.**

• L427: What is the effect of the synuclein injection on gait failures (i.e., before any optogenetic stimulation)?

➔ **We primarily aimed to use the gait failure measure to show that suprathreshold vs. perithreshold stimulation causes enhanced gait failure. We now have also included data from W1, W8-OFF, W8-PT and W8-Suprathreshold on Fig. 7c,d. Since the phenotype of the A53T vs. e.g. 6-OHDA mice is not that severe with regards to hypokinesia, there is no massive gait failure of A53T mice at W1 vs. W8-OFF. (also see response to and comment 13.4. by reviewer #1).**

• L431: "Smoothening of the gait" – Please make it more quantifiable. It sounds a bit abstract.

➔ **We agree with this point. This is why we used the "smoothening" term only to describe our inital subjective observations. Based on this, we then performed the oscillatory gait analysis (Fig. 7/Supplementary Fig. 14) to quantitatively describe the effect.**

• L439: I like the wavelet analysis, but it's difficult to understand what it corresponds to in terms of movement. What does a reduction in power in the 20 Hz band signify, and how much of an aberration does this represent at the movement level?

➔ **In the discussion we state "VNC optoactivation improved locomotor performance and harmonized gait patterns". A reduction in power means that the gait cycles are shifting away from their "natural frequency range" (around 4 Hz) with disease model establishment and this naturalistic frequency range is retuned with the stimulation. Further and more importantly, the main effect seems to be a harmonisation of the gait patterns as seen by the autocorrelation analyses (see Fig. 7m-r).**

• L459: his section could offer a clearer, movement-based perspective. Would it be interesting to comment on how much these stimulations mimic human effects obtained when targeting the vestibular circuitry?

➔ **This is great advice which we happily followed up on in the discussion: "Second, mimicking thresholded human vestibular neuromodulation, we optically re-tuned parkinsonian gait and locomotor deficits with activation of VNC glutamatergic neurons in mice, specifically re-stabilizing gait. This might explain the mixed human results from clinical trials on vestibular stimulation. In fact, the apparently improved stability during walking might come from improving vestibular contributions to balance, which has the best evidence in (parkinsonian states) in humans[60,21,23,27]."**

• L513-516: Perhaps this is the right place to specify the difference between locomotor capacity and locomotor performance.

- ➔ **That's a valid point by the reviewer. We added what we mean by that on lines 668-669. Specifically, capacity relates to movement distance why performance relates to movement itself.**

Reviewer #3 (Remarks to the Author):

This manuscript from the Tovote and Ip labs set out to study mechanisms and therapeutic effects of vestibular nucleus complex (VNC) stimulation in healthy rodents as well as a model of Parkinson's disease (PD). Strengths include detailed circuit tracing focused on the VNC network, use of subthreshold optical protocols to modulate neuronal activity in a manner relevant for translational applications, a very thorough and careful use of machine learning approaches for their behavioral studies (this was excellent!), and an elegant PD model. This study clearly enhances our knowledge of VNC function and has direct relevance to motor symptoms of PD mice. That said, in my opinion, data presentation and clarity of the figures/text requires some serious work before moving forward. I found myself searching for the relevant data panels for several results described in the text (which is unacceptable and is not ideal for the readers), sometimes necessary controls or supportive data for an experiment/technique showed up few figures later (see details below), and their molecular/microscopy work was not presented in a convincing manner.

The study is well-designed. I have the following suggestions to strengthen this study, which will enhance its' impact in the field (in the order that they appear). If I have misunderstood any points, please help point me in the right direction.

1. When I read the abstract and main text, they give the impression that VNC circuitry is functionally examined but that is not the case. All their manipulations are focused on VNC cell bodies (i.e., no terminal work). If they want to mention their circuit work, this should be clarified to indicate anatomical studies only.

- ➔ **The reviewer raises an important point. The statement of functional interrogation refers to the cFos analyses, which are considered markers of recent neuronal activity; Yet, it is correct that we did not perform any electrophysiological recordings, calcium imaging or terminal optogenetics. We adjusted the statement regarding cFos and adjusted the title, abstract (see lines 23-24) and main text accordingly where applicable.**

2. Fig 1a-b. I honestly cannot examine the micrographs to a rigorous level due to their small size. For essential staining/tracing/labeling data throughout the paper, please provide zoom-out and zoom-in images to help the reader appreciate the accuracy/specificity.

- ➔ **We thank the reviewer for helping to improve the readability of our paper. Throughout the manuscript, including the added figures, we increased the size of the micrographs and tried to provide zoom in & zoom out images wherever possible and relevant.**

3. Line 92. When they first introduce the A53T approach, they need to state unilateral or bilateral targeting. This is very important for PD and the motor behavior field. Related to this point, when the model is first introduced, we would expect a thorough characterization of this AAV approach and yet some data only show up I believe in Fig 3, which is not acceptable. Relevant panels should be moved here or in a supplemental figure that can be referenced when this model is first introduced. For this PD model, they need to provide alpha-synuclein expression, viral spread, cell death quantifications, and a representative injection site (some of this is already in Fig 3 but please confirm).

- ➔ **We included this information and with the updated, in-depth characterisation of the PD model, the other comments should be resolved as well (see Supplementary Fig. 6,7).**

4. Line 99. They need to provide these pilot data or clearly indicate which panel this is referring to. Results without supportive data should be avoided. Check the entire paper for a similar issue, please.

- ➔ **Resolved; no "pilot" data is included. What we meant by this statement is that when we started this project initially, we of course first checked what happens with optogenetic actuation of different intensities in 2-3 pilot mice and found this. Then we conducted our "real" experiments.**

5. Fig 1c. Add exact mW values to the graph. Same for other graphs throughout the paper. This is very important information.

- ➔ **Resolved; data added to all relevant graphs. (see specifically Fig. 3, 4)**

6. Lines 113-116. Where is this data? If not included in the paper, please provide them as a bar graph per mouse.

- ➔ **We appreciate the reviewer's observation and have now removed this statement from former lines 113-116, to avoid confusion.**

7. Fig 1e. The 5 colors chosen are clearly not distinct enough as it's not immediately clear to my eye upon first reviewing this panel where the clusters are. Please try to choose a color panel that is clearer to the reader.

- ➔ **That is an important point the reviewer makes. We changed the colors according to the reviewer's request (see Fig. 4 and Supplementary Fig. 11)**

8. Fig 2e. This data should be enlarged otherwise it's not very helpful to the reader.

- ➔ **For all our updated tracing figures we have generally enlarged the images and (relating to other comments on this issue by reviewer #2) we provided insets/zoom-in images whenever possible and relevant. (see Fig. 2,3 and Supplementary Fig. 1,2,4)**

9. Line 131. For performance, since two occasions led to ~90% accuracy, I think it's better to provide the exact values for each (not using ~). This is important validation data of their approach.

- ➔ **We extracted and provided the exact classifier performance (i.e. F1 score) for detection of each behavioural class on line 293.**

10. Lines 132-134. Provide UMAP cluster data with a brief description followed by these conclusions for enhanced clarity.

- ➔ **"Cluster data" are provided by means of the UMAP embeddings and cluster occupancy analyses (see Fig. 3 and Supplementary Fig. 11); we are not sure what the reviewer further means by that.**

11. Line 140. VSS1 is at 0.05 and for accuracy should be called out in the text as showing a 'very strong trend', for instance. This will alert the reader in a helpful manner.

- ➔ **Not applicable anymore, since we separated the data.**

12. Lines 141-143. Please add a concise interpretation of this observation.

- ➔ **See new Fig. 4 (c,d) and respective text section: "Since we saw that the thresholds for vestibular symptoms changed over time in both healthy EV- and parkinsonian A53T-ChRmine mice, we next asked whether these dynamics differed between groups when examined longitudinally. We therefore compared the laser powers required to elicit each VSS category at both week4 (Fig. 4a) and week8 (Fig. 4b). A53T-ChRmine mice required significantly higher laser powers than EV-ChRmine mice to elicit strong vestibular symptoms (VSS3 and 4), specifically at week8 (Fig. 4b), indicating hyporesponsiveness to vestibular optoactivation in PD mice. Furthermore, we tested whether the laser powers needed to elicit each VSS category correlated with mScarlet-positive VNC cell count, i.e. opsin-expressing neurons). We found that, predominantly at the extreme end of the symptom scale (VSS3 and 4), laser power thresholds negatively correlated with the number of mScarlet-positive cells in the EV- (Fig. 4c) and A53T-ChRmine animals (Fig. 4d) at both week4**

and week8. This means that only the extreme, strong vestibular symptomatology seems to depend on the amount of activated VNC neurons."

13. Lines 147-151. Why is this described here when the experimental application does not follow? Please move this to the manuscript section right before it will be tested in mice, otherwise it is very confusing.

➔ **We changed the ordering logic of introducing our perithreshold stimulation paradigm to right before we first report results on it (reporting with Fig. 4 on lines 362-374; first usage immediately after via Fig. 5 and onwards).**

14. In Fig 1, the authors need to indicate which data points correspond to A53T vs. EV mice. Even if there are no differences, this is critical to evaluate these data. Also, these animals are distinct and therefore possibly using different color data points, this is easy to highlight (e.g., panels g-j and k).

➔ **In line with the other comments on this matter from reviewer #1, A53T mice are always clearly indicated and separated from EV mice as seen on Fig. 3-7 and corresponding Supplementary Figures.**

15. Fig 2b-c. Please add mScarlet data to this summary table.

➔ **This is obsolete due to removal of table and rather insertion of quantification results for all the tracings (see Fig. 1 and 2, Supplementary Fig. 2,4)**

16. Fig 2. For microscopy images, please add the anterior-posterior information either on the images themselves or in the legend text. Do this across the paper please.

➔ **Adapted by provision of AP information in the figure legends providing microscopy images.**

17. Line 195. Should the Fig 2f-i just be 2f? Double check. Also, the Po data is not useful as presented. Provide a full-size image.

➔ **This is obsolete due to removal of table and quantification. The Po data is not discussed anymore and rather only PF and CM of which we have clear supporting data in relation to the quantifications introduced (Fig. 1). We provided full size images on Supplementary Fig. 4 which is data from former Fig. 2.**

18. Fig 2m-n. Is panel n a different coordinate section than panel m? If not, why is CnF not labeled in panel m? If these two panels are from different coordinates (even slightly), please provide better matched images to be more convincing.

- **This is a great point by the reviewer which should become obsolete with new images in Fig. 1, Supplementary Fig. 2,3.**

19. Line 207. Add unilateral or bilateral targeting information here.

- **Implemented. We show the data separated per hemisphere for the cFos data (see Fig. 5).**

20. Line 212. I know what OF may mean but others may not. For each abbreviation when first used, add a full name.

- **Implemented across the paper. We have rechecked (in line with other reviewers' comments) the abbreviations and hope the issue is resolved.**

21. Line 213. The cFos data for PF is a trend and this should be highlighted here for accuracy. For each cFos group/graph, please add representative cFos images for further support.

- **We have adapted the panels and provided data per hemisphere as suggested by reviewer #1. We have also adapted the text in this regard and have provided regional images for all inspected regions of interest (see Fig. 5).**

22. Fig 3. For model validation, I don't believe a full figure is very helpful. Instead, the other figure panels if increased in size and the authors need more main figure space, some of this figure could be used. Just a suggestion.

- **We agree and hence have rather provided two supplementary figures instead (see Supplementary Fig. 6+7).**

23. Fig 3a. Please provide separate channels for each image in the supplemental data.

- **We have provided separate channels for alpha-synuclein, tyrosine hydroxylase and counterstaining with fluorescent Nissl for those now (see Supplementary Fig. 6+7).**

24. Fig 4i. How to explain the clear trend in the mCherry group? This matches the A53T/EV groups and is concerning. Please add text to the paper to justify/explain.

- **We have rerun the keypoint-moseq pipeline entirely and have decided to not use the in-syllable kinematics and focus on the unsupervised results from this approach to inform on global changes in behavioural structure and usage (see Fig. 6).**

25. Fig 4j. Add mCherry OFF data to support habituation effect claim (since from BL to OFF shows the biggest decrease in the other graphs).

- ➔ **We thank this reviewer for noting this lack of data presentation. We have added these data in Fig. 6c,d.**

26. Fig 6b. Add an mCherry group here as an important control.

- ➔ **We agree that a control group for unspecific light effects is important. In fact, we show in the open field that light illumination up until 0,5 mW leads to no significant vestibular symptoms and no significant effects on locomotion. We thus did not test this again on the treadmill for Vmax testing.**

27. Fig 6u. The authors should describe the significance of the 20 cm/s data being n.s. between peak amplitude and peak frequency

- ➔ **We thank the reviewer for the suggestion and have discussed this on lines 580-583.**

28. EDF1. Confirm that it is referenced in the main text.

- ➔ **We thank this reviewer for noticing. Former EDF1 is now Supplementary Fig. 14. It has been referenced on line 586.**

Additional comments by the authors:

- We updated Supplementary Fig. 1 panels a-b for better layout and anatomical representation

Point-by-point author responses to remarks by the reviewers:

REVIEWER COMMENTS

Reviewer #1 (Remarks to the Author):

General opinion

Overall, the authors have made a substantial effort to revise the manuscript, and several methodological details and additional datasets have been added. However, the concerns raised in the initial review are only partially addressed.

Across multiple sections, a recurring issue remains a mismatch between the evidential strength of the data and the level of mechanistic or translational interpretation applied. Several datasets—most notably the anatomical tracing (Figures 1–2), the optogenetic titration experiments, and the c-Fos mapping—are inherently exploratory or descriptive in nature, often based on limited sample sizes, yet are framed using language that implies causal hierarchy, functional integration, or therapeutic relevance. In several instances, the conclusions would benefit from more cautious wording that better reflects the scope and limitations of the underlying data.

In addition, aspects of the authors' responses to reviewer comments rely on interpretative or assumptive statements rather than direct empirical evidence. Whilst these statements may represent reasonable hypotheses, they do not substitute for explicit reporting of the requested analyses (such as group-stratified classifier performance metrics) and occasionally deflect from the specific questions raised.

Taken together, whilst the revised manuscript contains valuable data and technical advances, further revisions are required to (i) align interpretation more closely with evidential support, (ii) temper mechanistic and translational claims where direct validation is lacking, and (iii) more directly and quantitatively address several outstanding methodological concerns. Addressing these points would substantially strengthen the rigor, clarity, and interpretability of the study.

Reviewer#1 comments

Comment 1.2.

We carefully reviewed the revised Methods section, Supplementary Tables, and the figures referenced by the authors. This comment is partially addressed. Whilst the stimulation paradigms for the titration experiments are now described in extensive technical detail, the core concerns regarding how stimulation intensity is defined and controlled remain insufficiently resolved. In the Methods and Supplementary Tables, stimulation intensity is still operationally defined in terms of current (mA), with an approximate and unvalidated conversion to optical power, which limits reproducibility across animals and laboratories.

Although the authors now report laser power at the fibre tip (μW) across VSS1–4 and between groups (Fig. 4), this reporting is not consistently aligned with the experimental definition of stimulation intensity, which remains primarily current-based. For clarity and reproducibility, optogenetic stimulation should be defined and reported consistently in terms of optical power, with behavioural thresholds used only for individual calibration rather than as primary descriptors of stimulation strength.

Furthermore, whilst Figure 4 reports individual-animal laser powers associated with each VSS category, the assignment of VSS remains based on the 'most prominent behaviour' (line 1074) observed per animal rather than on a predefined quantitative criterion. The authors should provide a quantitative definition of the 'most prominent

behaviour' is determined. In addition, within-animal threshold transitions are not shown, limiting interpretability at the individual-animal level.

Finally, although the authors refer to Supplementary Figures 5 and 6, we do not find information in these figures that directly addresses the above concerns.

-> First, we appreciate the reviewer's careful assessment of our revised manuscript. With respect to stimulation intensity reporting, we may have misunderstood the concern, as we believe the manuscript now reports stimulation intensities in terms of optical power (not only current strength). In particular, the reviewer notes that stimulation intensities are not provided in terms of optical power but rather as current, while also acknowledging that we report clear power-based values for all 4 VSS categories. In addition, we provide explicit optical power-based values for perithreshold stimulation in Figure 4f and 4g, shown separately for week4 and week8 testing and for both A53T-ChRmine and EV-ChRmine animals.

-> It is true that in contrast to many optogenetic perturbation experiments, our experimental design necessitated an individualized, symptom-titrated determination of stimulation strength, rather than an across-animals single stimulation strength analysis. Hence, during the conduction of the experiments, we acutely used an online, primarily current-based way to determine the individual animal's behavioural (peri)threshold level. Importantly, as stated in the manuscript (and consistent with standard practice), we then converted these values into optical power at fibre tip based on photometric measurement, performed on a per-animal/per-session basis. All optical fibres were commercially acquired from Doric Lenses Inc. (Canada), which are routinely pretested for basic optical throughput verification as well.

->The perithreshold power values are reported per animal (Fig. 4f,g). In our view, providing the exact fibre-tip power values both for the 1) individual VSS symptomatology thresholds as well as for the 2) perithreshold experiments supports full reproducibility of our experiments. Other groups could either 1) use the mean or median perithreshold (PT) values provided by us under comparable optogenetic construct, fibre, targeting condition and general technical setup, or 2) more realistically, apply the same standard practice of calibrating perithreshold behaviour online to account for inter-animal variability and convert these values into optical power.

-> Regarding the reviewer's comment "*Furthermore, whilst Figure 4 reports individual-animal laser powers associated with each VSS category, the assignment of VSS remains based on the 'most prominent behaviour' (line 1074) observed per animal rather than on a predefined quantitative criterion. The authors should provide a quantitative definition of the 'most prominent behaviour' is determined.*" We agree that this point can be clarified. First, during our pilot experiments, the symptoms that we observed were categorially operationalized in the VSS, which was developed for that purpose as a 'clinical' symptom scale. Symptoms under titration stimulation beyond the ones categorized within the VSS did not occur. Second, some mice exhibited mixed or "overflow" symptom patterns i.e. features of lower VSS levels (e.g. head tilts=VSS1) occurring alongside higher VSS levels (e.g. circling=VSS3). The statement "*most prominent behaviour"* in that context might not have been precise enough. In fact, for each VSS symptom reported, it is always the behaviour first occurring at an iteratively higher laser intensity. We acknowledge that this online behavioural assignment has an observer-based component; importantly, these observations thus motivated our semi-supervised, pose-based machine-learning analysis, which indicates that the symptom patterns observed 'clinically' and categorized within the VSS

largely converge into a single major cluster at the purely pose-based/kinematic level. We adjusted these statements on lines 1103-1112.

-> "In addition, within-animal threshold transitions are not shown, limiting interpretability at the individual-animal level.": We now also provide per-animal threshold transitions in Figure 4h and i. These findings extend interpretability in further underscoring the relationship of the categorial VSS to optical power across weeks, mice and both groups.

-> We thank the reviewer for noting the supplementary material issue; this was a labelling error on our side, and we intended to refer to Supplementary Tables 5 and 6 which showed an exemplary titration stimulation and an overview of the perithreshold paradigm in detail. However, Supplementary Table 5 contained only current based values, which we have corrected and included corresponding optical powers as well.

Comment 2.2.
Whilst the authors state that model validation data are now presented earlier, the main validation results are largely relegated to the Supplementary section. Consequently, the logical dependency between disease model establishment and the subsequent tracing and optogenetic experiments remains insufficiently delineated in the main text. Simply reordering sections, without foregrounding model validation as a central result, does not fully address the original concern.
Importantly, the anatomical tracing data presented in Figures 1 and 2 were obtained in non-parkinsonian mice and therefore represent baseline VNC connectivity. Whilst these data are informative, the manuscript implicitly treats them as a structural foundation for interpreting functional and therapeutic effects in the parkinsonian A53T model. In the absence of evidence that these circuits are preserved, altered, or functionally engaged in the disease context, such extrapolation is not sufficiently justified.

-> We thank the reviewer for this constructive comment. We believe that the manuscript follows a clear and coherent experimental thread. Specifically, we first delineate input-output patterns of the VNC on a whole-brain level, then, we present an in-depth validation of the A53T α-synuclein model in independent cohorts and subsequently perform optogenetic experiments that build upon the validated disease model. Finally, we provide evidence for target region (from tracing) engagement using a proxy of neuronal activity (c-Fos), as also noted by the reviewer.

-> Whilst the reviewer is correct that we did not perform additional anatomical tracing in parkinsonian mice, we have so far not observed evidence for macroscopic anatomical circuit alterations beyond the SN and the nigrostriatal pathway in the A53T-aSyn PD model. Nevertheless, we agree that we cannot formally exclude more subtle disease-related changes without disease-state tracing, and we clarified this limitation in the manuscript (lines 622-625).

-> With respect to the comment "In the absence of evidence that these circuits are preserved, altered, or <u>functionally</u> engaged in the disease context, such extrapolation is not sufficiently justified.", we would like to emphasise that, although we cannot prove that all these circuits are anatomically identical under PD conditions, we do provide disease-context evidence for a similar functional engagement of major VNC target areas. Specifically, c-Fos mapping, used as a proxy of neuronal activity, demonstrates recruitment of key downstream regions under matched stimulation conditions in both healthy <u>and</u> diseased PD mice, whereas this engagement is not observed in sham-stimulated mCherry control mice

under a perithreshold paradigm. We have strengthened this point in the manuscript (line 421-426) to more clearly support the relevance of the delineated VNC circuitry when interpreting the functional and therapeutic effects observed in the A53T disease context.

Comment 2.3.

Whilst the authors now present A53T and EV titration data separately (Figs. 3 and 4), the interpretation that A53T animals exhibit vestibular hypo- or dysfunction is not directly supported by the evidence presented.

The data convincingly demonstrate a shift in optogenetic stimulation thresholds, such that higher optical power is required to elicit specific VSS categories in A53T mice. This supports the conclusion that A53T mice show reduced responsiveness in terms of optogenetic threshold under the defined stimulation paradigm.

However, threshold shifts alone do not establish altered vestibular function per se. No independent assessment of baseline vestibular behaviour or reflexes in the absence of optogenetic stimulation (e.g. vestibular reflexes or balance-related assays) is reported. Whilst the additional cFos analysis confirms effective optogenetic engagement of VNC neurons (Supplementary Fig. 12), it does not establish intrinsic vestibular hypo- or dysfunction under baseline conditions, and interpretations should therefore remain limited to altered optogenetic responsiveness.

Accordingly, interpretations invoking vestibular hypo- or dysfunction should be tempered and reframed in terms of altered optogenetic responsiveness or stimulation thresholds, unless supported by additional, independent validation of vestibular function.

-> We thank the reviewer for this important clarification. We agree that we must temper this statement in the manuscripts' discussion and did so on line 652-654 that now reads: "Notably, we found signs for reduced vestibular responsiveness to optical stimulation in our PD mice. This might e.g. relate to previously described increased glutamate receptor expression in PD mice, which in turn can be normalized through GVS [71]."

Comment 3.2.

The issues raised here largely overlap with those discussed in Comment 1.2 and are therefore not reiterated separately.

-> As these points overlap with Comment 1.2 (by the reviewer), we thus refer to our detailed responses above.

Comment 3.4.

The authors address this point by showing that optogenetically induced postural effects are temporary and rapidly reversible (Supplementary Fig. 9), and by discussing differences from previous studies in stimulation duration and pulse parameters. Whilst these observations are informative, they do not fully resolve the concern raised. Specifically, the reversibility of overt postural effects does not exclude the possibility of residual or cumulative effects on vestibular circuit excitability across successive stimulation blocks. The manuscript does not provide an experimental control in which each VSS-associated light intensity is applied as an independent condition, without prior stimulation history, as originally suggested.

Therefore, it remains unclear whether the emergence of distinct VSS categories reflects light intensity alone, or is influenced by repeated stimulation, temporal accumulation, or order effects. This limitation should be more explicitly acknowledged in the interpretation of the titration experiments.

-> We thank the reviewer for this thoughtful point. We agree that demonstrating rapid reversibility of overt postural effects (Supplementary Fig. 9) does not fully exclude more subtle residual or cumulative

changes in vestibular circuit excitability across successive stimulation blocks, nor does it rule out potential order effects during the titration procedure. As the reviewer notes, our design does not include a control in which each VSS-associated intensity is tested as an independent condition without prior stimulation history. We have therefore explicitly acknowledged this limitation in the discussion (lines 648-651).

Comment 4.4.
The original comment asked a single, specific question: whether prediction accuracy differed between the A53T and EV groups. Whilst the authors' response describes revisions to the training procedure and reports overall classifier performance, it does not directly address this point, as group-stratified performance metrics are not provided. Please report classifier performance separately for A53T and EV (e.g. precision, recall and F1 score, and ideally confusion matrices) on the held-out test set to directly address this point.
-> We thank the reviewer for the clarification. To address the question directly, we now report group-stratified performance metrics in the methods section of the paper one lines 1328-1342.

Comment 4.5.
Whilst the alluvial-style plot may be visually intuitive, it does not provide the requested group-stratified quantification. Please include a concise table reporting human–machine agreement (counts and percentages) for each VSS category, separately for EV and A53T, so that any group-dependent performance differences can be assessed transparently.

-> We thank the reviewer for this suggestion. To address this point transparently, we now include a concise group-stratified human-machine agreement per group on Supplementary Fig. 10d and its associated figure legend.

Comment 7.2.
Whilst the discussion has been expanded, the statement that the authors 'found signs for vestibular hypoexcitability' in PD mice (line 630) remains stronger than what is directly supported by the presented data (see Comment 2.3). The results demonstrate altered optogenetic stimulation thresholds and responsiveness, but do not independently establish baseline vestibular hypo- or hyperexcitability. The authors are encouraged to further temper this terminology, or to explicitly frame these findings in terms of altered optogenetic responsiveness or circuit-level modulation.

-> As stated in our response on comment 2.3., we agree with the reviewer that this statement should be tempered, and we have revised the wording accordingly in the Discussion (line 652). We also re-checked the manuscript and confirm that we do not refer to vestibular "hypofunction" elsewhere. This terminology was limited to the Discussion section and has now been adjusted.

Comment 7.3.
The authors have corrected the time-point labelling for clarity. The original comment arose specifically because week 4 data were labelled as 'baseline' in the originally submitted manuscript, which created ambiguity regarding the temporal interpretation of the results. The concern was therefore terminological, not related to experimental feasibility or expectations regarding viral expression at early time points.
In this context, the explanation of why titration experiments were not performed at week 1 does not directly address the source of the confusion. Rather, the issue stems from mislabelling, which in turn prompted the

original comment.
Whilst the authors indicate that the relevant labels have now been corrected (i.e. ‘baseline’ replaced with the appropriate time point), given the importance of temporal definitions for longitudinal interpretation, the authors are encouraged to re-check all figures, supplementary figures, and legends to ensure consistent and unambiguous labelling of time points throughout the manuscript, so as to avoid future confusion or misinterpretation (see also Supplementary Fig. 6g; minor comments below).
-> We thank the reviewer for clarifying that the concern was purely terminological. We apologise for the initial mislabelling of the week 4 data as “baseline,” which could indeed create ambiguity in the longitudinal interpretation. We have corrected this throughout the manuscript (replacing “baseline” with the appropriate time point) and have re-checked all main figures, supplementary figures, and figure legends to ensure consistent and unambiguous labelling of time points across the entire manuscript, including the referenced Supplementary Fig. 6g.

Comment 10.2.
The authors have provided the requested data. The c-Fos analysis provides useful evidence for region-specific activity changes in VNC target areas following perithreshold stimulation; however, aspects of the current framing and interpretation go beyond what is directly supported by c-Fos expression alone.
In particular, the subsection heading and some concluding statements imply a level of functional integration and causal network hierarchy that is not established by these data. c-Fos is an indirect proxy of neuronal activity and does not, by itself, demonstrate directionality, functional coupling, or circuit-level integration.
Given the limited sample sizes per group and the exploratory nature of this mapping, the authors are encouraged to temper the language throughout Figure 5 and the associated text, and to frame these findings more cautiously as evidence of activity engagement and/or recruitment in specific regions, rather than definitive ‘axis’-level modulation or an ‘interface’ between systems.
-> We tempered the language throughout Figure 5 and its associated passages (see line 408 onwards) in the main text.

Comment 11.3.
Whilst the authors argue that control protein overexpression is unlikely to account for the observed effects, subtle contributions of expression burden under the current experimental conditions cannot be fully excluded. The conclusions should therefore be interpreted with this consideration in mind.
-> We agree with the reviewer that subtle contributions of expression burden cannot be fully excluded under the present experimental conditions. We have therefore tempered the wording and explicitly acknowledge this as a limitation in the discussion and interpret our conclusions with this consideration in mind. Lines 682-683 now read: “Yet, a limitation is that subtle effects of viral/protein expression burden cannot be fully excluded and may have contributed, at least in part, to the observed phenotype”

Comment 11.6.
The reported ‘mild reduction (5.5%)’ (line 251) is not accompanied by a statistical comparison between OFF and PT within the A53T group. Please indicate in the main text whether the OFF vs. PT difference is statistically significant, including the statistical test and exact p-value.

-> We did so on line 253-254: “Of note, this static rotatory asymmetry did show a mild, non-significant reduction (5.5 %, corr. p = 0,4867, Tukey’s multiple comparisons) with perithreshold VNC actuation”.

Comment 11.6.

The authors have expanded the Methods and improved the transparency of the Keypoint-MoSeq analysis. Whilst the current presentation is acceptable, the figure still functions primarily as a high-level conceptual overview rather than a step-by-step analysis pipeline. An improved workflow schematic would substantially improve readability.

-> We thank the reviewer for this suggestion. In the revised manuscript, we have clarified the Keypoint-MoSeq methodology by combining (i) the original Keypoint-MoSeq publication reference, (ii) Fig. 5 as a conceptual overview, and (iii) an expanded Methods section with the specific analysis steps used in our study beyond the original publication by the Datta group.

Comment 12.3.

The authors chose to remove in-syllable analyses, but this does not resolve the original concern regarding the selection criteria and organisation of behavioural syllables. The manuscript still lacks a clear and systematic presentation of the full syllable repertoire, which limits interpretability of the KPMS results.

-> We thank the reviewer for this comment and agree that interpretability benefits from a clear view of the underlying syllable repertoire. In our study, the complete set of syllables is provided in Supplementary Video 5, shown as dynamic keypoint-based gifs (which we also attached below). In Keypoint-MoSeq, syllables are organised hierarchically and indexed such that syllables 0→X correspond to decreasing frequency, consistent with the standard framework described in the original methodology paper (Weinreb et al.), which we cite and follow. We intentionally do not single out or manually “select” specific syllables for focused analysis, precisely because the criteria for doing so can be ambiguous and may introduce selection bias and multiple-comparison concerns. Instead, we use the full syllable behavioural state space as a bird’s-eye, non-assumptive, data-driven representation of behaviour, and interpret longitudinal group differences at the level of the full repertoire and its distributional structure rather than a small, preselected subset.

Comment 12.4.

Whilst the authors chose to remove the in-syllable kinematic analyses, this does not resolve the original concerns regarding baseline group differences, similar OFF–PT changes across groups, or the possibility of non-specific effects of the PT condition.

-> We thank the reviewer for reiterating these concerns. In the revised manuscript, we provide analyses that clearly distinguish true PT stimulation from the sham PT condition, in our view thus excluding subtle non-specific effects beyond reasonable doubt. Our statistical results indicate a) a clear and highly significant baseline (week 1) vs. week 8 OFF change in the PD group, contrasted with a smaller, less significant change in EV and mCherry controls that is consistent with expected habituation-related effects. Importantly, we b) do not observe a statistically significant effect of the sham PT condition, whereas we do detect significant effects under true PT stimulation in both EV-ChRmine and A53T-ChRmine groups. Together, these analyses argue against a purely non-specific PT effect and support a stimulation-specific component in the observed changes. For comparability, we specifically aligned the

analyses (global shift analysis for Fig. 6g-i) to our basic kinematic analysis (Fig. 6c,d) doing only longitudinal intragroup comparisons.

Comment 13.4.

Although the authors provide a detailed and transparent description of the FGI scoring system, the interpretation of the corresponding results is not fully supported when considered alongside the broader locomotor data presented in Fig. 7.

First, the authors state that ‘there is no massive gait failure of W1 vs. W8-OFF’ because the A53T phenotype is relatively mild with respect to hypokinesia. However, Fig. 7b demonstrates a clear and significant reduction in maximal treadmill velocity in A53T-ChRmine mice compared to EV controls, indicating a measurable locomotor impairment. This discrepancy suggests that, whilst overt gait failure events are rare under OFF conditions, the model nevertheless exhibits meaningful baseline motor deficits that are not captured by the categorical FGI metric.

Second, baseline (OFF) gait failure rates in Fig. 7c,d are low in both A53T- and EV-ChRmine mice and do not robustly differentiate the parkinsonian group from controls. This indicates that, as implemented here (FGI1 vs. FGI2+3), the FGI readout may have limited sensitivity for detecting disease-related baseline gait impairment, despite the presence of hypokinetic features revealed by velocity measures.

Third, perithreshold (PT) stimulation produces little to no measurable change in gait failure relative to OFF in either group. Whilst this is consistent with the authors’ statement that PT does not worsen gait, it does not provide convincing evidence for a beneficial or rescuing effect at the level of gait failure. The description that impaired gait is ‘mildly reduced’ by PT is difficult to reconcile with the categorical nature of the FGI and the minimal visible difference between OFF and PT conditions.

Finally, the use of trial counts pooled across animals further complicates interpretation, as it remains unclear whether any apparent differences reflect consistent within-animal effects or are driven by variability in trial numbers contributed by individual animals.

Overall, the FGI data convincingly demonstrate the deleterious effects of suprathreshold stimulation but do not support a meaningful improvement in gait failure with perithreshold stimulation. The authors are encouraged to more clearly acknowledge the limited sensitivity of FGI to baseline hypokinetic deficits, to align statements regarding disease severity with the velocity data in Fig. 7b, and to clearly distinguish FGI-based outcomes from the oscillatory gait ‘retuning’ effects described in subsequent analyses.

-> We thank the reviewer for this detailed and constructive assessment of the FGI results. We agree with the reviewer’s interpretation that the FGI metric primarily captures overt gait failure events (e.g., stopping/falling) and is therefore most informative for demonstrating the deleterious effects of suprathreshold stimulation, which was our original rationale for including it. In response to the reviewer’s previous comment, we nevertheless extended the FGI analysis to also report the perithreshold (PT) condition, to explicitly test whether threshold-level stimulation produces any measurable change in gait-failure events. As the reviewer correctly notes, when considered alongside the broader locomotor readouts in Fig. 7, this also highlights an important limitation: FGI has limited sensitivity for graded baseline hypokinetic deficits. Accordingly, we have revised the text to avoid implying “no impairment” under OFF conditions and aligned our description of disease severity with Fig. 7b, which shows a significantly lower maximal treadmill velocity in A53T-ChRmine mice relative to EV controls. Consistent with this, baseline OFF gait-failure rates (FGI1 vs. FGI2+3) are low in both groups and do not robustly differentiate A53T from controls, indicating that baseline hypokinesia is better captured by continuous locomotor metrics (e.g., maximal velocity) than by categorical failure events. Regarding PT stimulation,

we agree that the FGI data do not support a meaningful “rescue” of gait failure; rather, they show that PT does not worsen gait failure relative to OFF. We therefore tempered the corresponding wording and clearly distinguish FGI-based outcomes (gait failure events) from the subsequent oscillatory gait “retuning” analyses, a term we reserved for the “retuning” to normal in the correlation analysis. In addition we have changed the title to: Vestibular nucleus stimulation for ameliorating locomotor deficits in Parkinson's disease.

“Finally, the use of trial counts pooled across animals further complicates interpretation, as it remains unclear whether any apparent differences reflect consistent within-animal effects or are driven by variability in trial numbers contributed by individual animals.” -> Lastly, we acknowledge that pooling trial counts across animals can complicate interpretation. However, in our analysis, each animal contributed the same number of trials to the analysis per each group.

Additional major comment (Figures 1–2 and related text)
In the revised manuscript, the authors added extensive qualitative brain-tracing images and analysis. In the current manuscript, Figures 1 and 2 form the foundation of the manuscript and present extensive anatomical tracing of Vglut2-VNC output and input circuits. Whilst these datasets are valuable and technically sound as exploratory brain-wide mapping studies, their current presentation and interpretation extend beyond what is supported by the underlying data. Both figures are based on n = 3 animals and primarily provide descriptive connectivity maps, yet the text repeatedly uses quantitative and functional language (e.g. ‘quantitatively predominant targeting’, ‘hyperdirect influence’, and ‘anatomical prerequisite for modulatory impact’) that implies functional hierarchy and translational relevance.
In this context, the absence of inter-animal variability measures, individual data points, or other indicators of robustness makes such strong framing difficult to justify. Whilst n = 3 and error-bar–free representations are not uncommon in tracing studies, they are typically accompanied by more cautious, descriptive language. As currently written, the manuscript treats these anatomical results as a functional and conceptual foundation for downstream perithreshold stimulation experiments and therapeutic analogies, which creates a mismatch between data strength and claim level.
We therefore believe that this section requires substantial revision, including a more restrained interpretation of Figures 1 and 2, clearer acknowledgement of their exploratory nature, and tighter alignment between the anatomical data and the functional/translational claims built upon them. Without such revision, the anatomical results risk being over-interpreted relative to their evidential scope.

-> We thank this reviewer for this careful and constructive assessment. We agree that Figures 1 and 2 represent exploratory brain-wide tracing datasets based on a limited number of animals (n = 3 per tracing experiment) and that our wording in places could be interpreted as implying a stronger functional hierarchy than can be concluded from these anatomical data alone. We have therefore revised the corresponding Results/Discussion text to adopt a more restrained, descriptive framing.

Specifically, we have 1) removed or rephrased quantitative/functional language that could be over-interpreted (e.g., terms such as “quantitatively predominant targeting”, “hyperdirect influence”, “anatomical prerequisite for modulatory impact”) and replaced it with descriptive statements about observed projection patterns and candidate nodes for subsequent functional testing. 2) We now explicitly state that the tracing provides a baseline connectivity map and does not by itself establish functional hierarchy or translational relevance. 3) We have tightened the logic linking anatomy to

downstream experiments by clarifying that the tracing serves to nominate targets and constrain hypotheses, while functional engagement and behavioural effects are supported by the optogenetic and c-Fos results presented later in the manuscript. Overall, we believe these revisions bring the anatomical section into closer alignment with the evidential scope of the tracing data and avoid over-interpreting exploratory connectivity maps as functional proof.

Additional minor comments
- The labelling in Supplementary Fig. 6g appears potentially inconsistent. Could the authors please verify the labels for image rows 3 and 4? In addition, based on visual inspection, the Week4 panels seem to show fewer SNc TH+ neurons than the Week8 panels in EV-WT, which appears counterintuitive given the reported quantifications. Please clarify whether the correct images/time points are shown and, if applicable, provide the corresponding quantification for the specific panels displayed.

-> We thank the reviewer for carefully checking Supplementary Fig. 6g and apologise for the confusing panel ordering. We have re-checked the figure and can confirm that the images/time points themselves were correctly assigned, but the ordering/row labelling of panels 3 and 4 was inconsistent. We have corrected the ordering/labels in Supplementary Fig. 6g to ensure that rows 3 and 4 are unambiguous.

Regarding the reviewer's observation that the Week4 EV-WT panels appear to show fewer SNc TH+ neurons than the Week8 panels, we agree that representative immunohistochemistry images can vary visually and are not always perfectly intuitive when inspected in isolation. Importantly, the quantitative analysis shown in Supplementary Fig. 6c (based on the predefined quantification pipeline/ROI selection) shows no significant difference in SNc TH+ neurons between EV-WT Week4 and Week8. We have clarified in the legend/text that the panels in Supplementary Fig. 6g are representative examples and that variability in TH staining patterns across the SNc can arise from section level and rostro-caudal (antero–posterior) position.

- Figure 5i legend in the figure is cropped.

-> We corrected the image crop.
- Line 251, please correct the decimal separator from 5,5% to 5.5%.

-> We corrected the decimal separator.
- Label A53T-ChRmine (OFF) and A53T-ChRmine (PT) on the Fig7e and F respectively to improve readability.
-> A53T-ChRmine (OFF) and (PT) are already labelled on Figure 7 panels e and f.

Review #2 comments
Abstract: Addressed in part. The revised abstract removes the ambiguous phrase, but it still does not clearly define what is meant by 'performance' versus 'capacity' in measurable terms (i.e. which readouts correspond to each), which was the core request for broader readership.
-> We thank the reviewer for addressing this. We agree that "performance" and "capacity" should be defined in measurable terms directly for a broad readership. We have therefore explicitly defined both terms at first mention by linking them to the corresponding readouts: performance refers to changes in motion value (behavioural dynamics/locomotor performance metric), whereas capacity refers to distance

moved (locomotor capacity). These clarifications are now stated in the figure title at lines 431–432 and mentioned in the main text on lines 465, 469.

Introduction

• L42: Addressed.

• L49: Addressed.

• L53: Addressed.

• ‘In optogenetic pilot experiments’: This point is partially addressed. Whilst detailed stimulation parameters are now provided in the Methods and Supplementary Tables, the reviewer’s request to include a concise description of key stimulation parameters and the temporal profile of behavioural effects at least once in the main text has not been fully met.

->We thank the reviewer for this clarification. In addition to the detailed parameters provided in the Methods and Supplementary Tables, we have now added a concise summary of the key stimulation parameters in the main text (lines 269-271): “Across experiments, we generally used 20 Hz orange light illumination at 10 ms pulse width (…).” To address the requested temporal profile of behavioural effects, we also added a brief statement in the same section indicating that the behavioural/postural effects emerged “Vestibular symptoms occurred promptly upon activation of VNC illumination and in pilot experiments, […]”.

• L150: Addressed.

• L183-184: Addressed.

• L262: Addressed.

• Limb kinematics raw image: Addressed.

• L268: Addressed.

• Fig. 4i: The reviewer’s question regarding the unexpectedly elevated OFF–PT values in the mCherry group remains insufficiently addressed. While the authors note the removal of in-syllable kinematic analyses, this does not directly explain the near-significant elevation observed in the control group. Clarification regarding potential contributing factors (e.g. inter-animal variability, sample size, outliers, or analytical considerations), or an explicit acknowledgment that this variability remains unexplained, would improve transparency.

-> We thank the reviewer for highlighting this point. We removed the in-syllable analyses because we became concerned about their statistical validity when applied across many syllables, including the risk of inflated type-I error and unstable estimates in low-frequency syllables. Since running was not a very common syllable, the inter-animal variability in the in-syllable kinematics (and respective plotting as median with 95% CI) likely gave this impression. However, we acknowledge that we cannot fully explain this variability. Yet, in the revised kpms model and downstream analyses, focusing on global behavioural usage and transition-level metrics, we observe a clear PT effect in EV- and A53T-ChRmine groups, whereas no corresponding PT effect is present in the mCherry controls. We have updated the manuscript accordingly and appreciate the reviewer’s request, which amongst other comments has helped us identify and correct this issue.

• L332: The revised figure clarifies that perithreshold stimulation was implemented as a fixed offset below each animal’s VSS1 threshold (10 mA, corresponding to ~1–2 μW at the fibre tip), which partially addresses the reviewer’s request. However, for reproducibility, it would be helpful to also provide a concise operational definition

of ‘perithreshold’ in the main text, ideally in power-based terms and/or as a relative range or percentage of threshold. In addition, clarification of how the mA-to-μW conversion was calibrated across animals would further strengthen methodological transparency.

-> We thank the reviewer for this helpful suggestion. In the main text, we now explain that we stimulated 1-2 uW below the individual animal’s VSS1 threshold for perithreshold stimulation and provide conversion information in the methods section, which was conducted by a calibrated photometer at a per-session basis. Also see comments on 1.2 from reviewer 1 and methods section lines 1106-1122.

• L346: addressed.

• L361: The response does not fully clarify how the reviewer’s concern regarding the conceptual meaning of ‘covert representation’ under low-intensity stimulation was resolved. While the authors note that in-syllable kinematic analyses were removed, it remains unclear whether the statement itself was removed, rephrased, or conceptually withdrawn in the revised manuscript. Please explicitly indicate how this issue was resolved in the revised manuscript.

-> We thank the reviewer for pointing this out. In the revised manuscript, we have removed the statement referring to a ‘covert representation’ under low-intensity stimulation, because this interpretation was based on the in-syllable kinematic analyses that we subsequently removed due to the statistical concerns outlined above. Accordingly, the concept is not retained in the revised text, and our interpretation is now limited to the remaining, robust behavioural and transition-level results.

• L427: The authors’ response clarifies that the primary purpose of the gait failure metric (FGI) was to contrast suprathreshold versus perithreshold stimulation. However, this does not directly address the reviewer’s original question regarding baseline gait failure in A53T mice prior to optogenetic stimulation (e.g. W1 or W8-OFF). Notably, Fig. 7b shows a significant reduction in maximal treadmill velocity in A53T-ChRmine mice relative to EV controls, indicating a measurable baseline locomotor impairment. In this context, the low incidence of gait failure events in Fig. 7c,d may reflect a limited sensitivity of the FGI to baseline hypokinetic deficits, rather than an absence of disease-related motor impairment. Explicitly clarifying what conclusions can and cannot be drawn about baseline gait failure from the FGI data, and acknowledging that the FGI primarily captures overt gait breakdown rather than more subtle hypokinetic features revealed by velocity-based measures, would help align the interpretation across figures.

-> We adjusted our statements on FGI vs. Vmax, also in line with comments by reviewer 1: “Hence, while a forced paradigm (max. velocity) seems to de-mask a gait deficit in our PD mice, the FGI could not reliably capture this, yet substantiate the deleterious effects of suprathreshold stimulation.” (see lines 548 onwards)

• L431: The authors appropriately clarify that 'smoothing of gait' was initially descriptive and is now followed by oscillatory analyses (Fig. 7; Supplementary Fig. 14), which provide quantitative support for specific gait-related signals. However, several reported effects appear modest and/or marker- and speed-dependent, and it is not always clear that they generalise across parameters. To avoid over-interpretation, the authors are encouraged to (i) temper terms such as 'resynchronisation/retuning', or explicitly define them operationally, and (ii) provide a

concise summary (direction and effect size and/or a summary statistic) indicating which oscillatory metrics show consistent PT-associated changes across speeds/markers versus more selective or exploratory effects.
-> We believe this overlaps with L439/L459 and is responded to below.

• L439/L459: While the oscillatory analyses are described as in-depth and robust, the reported effects are largely confined to specific conditions, most notably a single speed (20 cm/s) and selected ipsilesional limb markers. Effects are not consistently observed across speeds, markers, or coordinates, and several findings are described as trends or mild changes. In this context, phrases such as 'retuning towards week1 levels', 'resynchronisation', or 'harmonisation' may benefit from more cautious framing or explicit operational definitions. A brief summary highlighting which oscillatory metrics show statistically supported and consistent PT-associated changes, versus more condition-specific effects, would further strengthen the interpretation.
-> We used these terms since we show specific disease-associated changes, in turn specifically addressed by the stimulation. We believe that the absence of coarse, across-marker effects rather demonstrates the robustness and specificity of these results, specifically within the biological framework of a unilateral PD model with unilateral optogenetic target area stimulation. However, we agree that terms such as resynchronisation and the like must be tempered in this context. Accordingly, we have further tempered terms such as "retuning", "resynchronisation", and "harmonisation" to more descriptive language. In addition we have changed the title to: Vestibular nucleus stimulation for ameliorating locomotor deficits in Parkinson's disease.

Reviewer #3 (Remarks to the Author):
They did a good job with this revision.
Fig4c-d = I would change the "yellow" datapoints since they are very hard to locate.

-> We thank the reviewer for the positive comment on our revision. We changed the yellow datapoints to a rather orange colour for better visibility.

Response to reviewers:

REVIEWERS' COMMENTS

Reviewer #1 (Remarks to the Author):

General opinion

I thank the authors for their careful and substantial efforts in addressing the previous round of comments. The revised manuscript is clearly improved in multiple aspects, particularly with regard to methodological transparency, interpretational tempering, and additional analytical clarification. Importantly, most of the major scientific concerns raised previously have now been adequately addressed without requiring additional experimental work.

In particular, the additions regarding group-stratified classifier performance metrics, the expanded explanation of the A-SOiD workflow, the clarification of sham perithreshold stimulation effects, and the moderated interpretation of locomotor ‘rescue’ versus locomotor ‘performance’ are all appreciated and substantially strengthen the manuscript.

After carefully cross-checking the rebuttal against the revised manuscript, I find that the majority of my previous concerns are now satisfactorily resolved. The remaining issues are relatively minor and relate primarily to interpretational consistency, presentation, and methodological clarity.

In particular, whilst the anatomical tracing sections are now framed substantially more cautiously and appropriately as exploratory analyses, a small number of stronger interpretational expressions still remain in isolated parts of the manuscript and figure legends. Ensuring that the intended

interpretational tempering is applied consistently throughout the text and figures would further improve clarity and consistency.

Comment 1.2

Whilst the revised Methods now clarify that each VSS category corresponds to the first occurrence of the respective symptom pattern, some individual trajectories in Fig. 4h appear non-monotonic across VSS levels. It therefore remains somewhat unclear how symptom categories were assigned in cases where higher-order symptoms emerged at lower stimulation powers than lower-order categories. Adding a brief clarifying sentence in the Methods or figure legend describing how such cases were operationally handled would further improve interpretability.

→ We appreciate the reviewer's concern regarding the symptom trajectories. Symptom categories were assigned on a symptom-basis, not a threshold basis. Thus, it was indeed possible (this is *n* = 1 animal in the EV group) that there wasn't a strictly ascending threshold optical power-VSS relationship, and an animal might have shown head bobbing and tilting at a higher laser power that VSS2 symptoms. Since the VSS was pre-defined after pilot experiments, where most animals showed this ascending, monotonic relationship, for this animal we assumed it had to do with an idiosyncratic response that VSS1 symptoms (i.e. head tilt and bobbing) indeed rather occurred at higher laser power than VSS2 symptoms (i.e. body tilting). See adapted lines 747 to 752.

Comment 2.2

The authors have substantially tempered the original anatomical-functional interpretation and appropriately acknowledge the exploratory nature and limited sample size of the tracing experiments. This substantially improves the alignment between the evidential scope of the data and the interpretation. However, several stronger interpretational terms remain in parts of the revised manuscript and figure legends.

→ We agree that some stronger interpretational wording remained, especially in the tracing figure legends, which we have changed: e.g. removed statements such as "strong", "robustly", where applicable.

Comment 3.4

Adequately addressed. Whilst the potential influence of stimulation history or cumulative vestibular activation is not experimentally excluded, the revised manuscript now explicitly acknowledges this limitation and appropriately tempers the interpretation of the titration experiments accordingly. No further experimental clarification appears necessary.

→ No further comments.

Comment 4.5

The additional group-stratified matching analysis is helpful and substantially improves transparency of the classifier performance. However, the reported human-versus-machine matching counts for several VSS categories appear relatively small (e.g. n = 2–3 events), making it somewhat difficult to assess the robustness of the validation procedure. A brief clarification regarding how these event counts were derived (e.g. event-level versus video-level scoring, number of human annotators, and whether validation was performed by a single or multiple raters) would further improve interpretability. Including this clarification in the Methods section or figure legend would be helpful. Given the relatively small sample sizes underlying some of the reported perfect matching values, the authors are encouraged to interpret these classifier validation results cautiously and avoid overstating their robustness.

→ We appreciate the reviewers concern and added a brief clarification regarding these issues in the Figure Legend for Supplementary Figure 10. Also see in Methods: "To independently validate the final A-SOiD classifier (random forest; 729 features), we re-extracted pose-derived features from raw DLC trajectories and re-ran prediction using the frozen iterX model. Across all recordings (*n* = 130,241 temporal bins), the classifier achieved high overall accuracy (97.7%), with a micro-averaged F1 score of 0.977 and a weighted F1 score of 0.973. Performance was solid for the two primary behavioural classes, 'Naturalistic Behaviour' (class 0; F1 = 0.987) and 'Vestibular Symptomatology' (class 1; F1 = 0.944), indicating reliable discrimination between spontaneous and vestibular-evoked states. Stratified analysis revealed comparable performance across genotypes. In EV-ChRmine animals (*n* = 46,477 bins), classification accuracy reached 98.8% (micro F1 = 0.988; weighted F1 = 0.986), whereas in A53T-ChRmine animals (n = 83,764 bins) accuracy remained high at 97.1% (micro F1 = 0.971; weighted F1 = 0.967). Class-wise precision and recall for both 'Naturalistic Behaviour' and 'Vestibular Symptomatology' were consistent between groups, indicating that classifier performance generalizes across genotypes without evidence of systematic bias. Together, these results confirm stable behavioural state decoding from pose dynamics using our trained A-SoiD[49] model (see Supplementary Figure 10 for details). We then predicted titration data from week4 and week8 fully."

Comment 11.6

The expanded methodological description of the Keypoint-MoSeq pipeline is appreciated. However, this still does not fully substitute for the previously requested static workflow schematic illustrating the complete KPMS analysis pipeline in a concise stepwise format. Given the analytical complexity of the manuscript, inclusion of such a schematic would substantially improve readability, accessibility, and reproducibility for readers who are not already familiar with the pipeline.

→ We included a workflow schematic as Supplementary Fig. 16 as requested.

Comment 12.3

The authors provide a reasonable rationale for avoiding selective, manually curated syllable analyses and for instead focusing on the full behavioural state space. However, this does not fully resolve the original concern regarding the systematic presentation and interpretability of the complete syllable

repertoire. Whilst Supplementary Video 5 is useful, a concise static supplementary figure or table summarising all syllables (e.g. index, relative frequency, and brief descriptor/example frame) would substantially improve accessibility and interpretability of the KPMS results for readers.

→ We appreciate the reviewers' concern. We provide the relative frequency, index and a short descriptor for each syllable in Supplementary Table 8. Supplementary Video 5 substitutes for a sample frame since it shows the full pose dynamics that constitute each syllable.

Minor presentation consistency

Minor formatting inconsistencies remain (e.g. mixed decimal notation styles) and should be standardised throughout the manuscript prior to publication.

→ We cross-checked and homogenized these formatting inconsistencies.

Summary

Overall, I believe the manuscript is now substantially strengthened and that the remaining issues are relatively minor, primarily relating to presentation, consistency, and accessibility rather than underlying scientific validity. I do not believe additional experiments are necessary. Provided that the above remaining points are adequately addressed, I would be supportive of publication.
