# Supplementary material for: Vestibular nucleus stimulation for ameliorating locomotor dynamics in a Parkinsonian mouse model
Source: Nat Commun. 2026 Aug 4;17:7772. doi: 10.1038/s41467-026-76183-2 (PMC13439372; doi:10.1038/s41467-026-76183-2)
Supplement: Supplementary file 3 [file 41467_2026_76183_MOESM3_ESM.docx]

**Description of Additional Supplementary Files**

Supplementary Video 1

Description: Example of a mouse with titration at 4 different thresholds for the different degrees of VSS-classified symptoms to appear.

Supplementary Video 2

Description: Example montage video of a mouse with repeated stimulation at different laser powers across a top-view open field (OF) trials during titration experiments

Supplementary Video 3

Description: Example montage video of a mouse with repeated stimulation at different laser powers across a top-view open field (OF) trials during titration experiments

Supplementary Video 4

Description: Video of a mCherry mouse sham-stimulated intermittently at 0,5 mW laser power in the open field

Supplementary Video 5

Description: Video showing all syllable trajectories

Supplementary Video 6

Description: Single dual view treadmill video with gait under suprathreshold vestibular optogenetic actuation shows gait impairment

Supplementary Video 7

Description: Syllable 0 - compact straight stance

Supplementary Video 8

Description: Syllable 1 - compact leftward curl

Supplementary Video 9

Description: Syllable 2 - low straight glide

Supplementary Video 10

Description: Syllable 3 - elongated head-forward stretch

Supplementary Video 11

Description: Syllable 4 - compact slight-turn stance

Supplementary Video 12

Description: Syllable 5 - compact right-curled dip

Supplementary Video 13

Description: Syllable 6 - straight midline glide

Supplementary Video 14

Description: Syllable 7 - broad straight extension

Supplementary Video 15

Description: Syllable 8 - tight straight pause

Supplementary Video 16

Description: Syllable 9 - compact side bend

Supplementary Video 17

Description: Syllable 10 - elongated straight track

Supplementary Video 18

Description: Syllable 11 - elongated slight-tail curve

Supplementary Video 19

Description: Syllable 12 - straight head-forward push

Supplementary Video 20

Description: Syllable 13 - sharp leftward bend

Supplementary Video 21

Description: Syllable 14 - long straight forward push

Supplementary Video 22

Description: Syllable 15 - compact straight shuffle

Supplementary Video 23

Description: Syllable 16 - elongated head sweep

Supplementary Video 24

Description: Syllable 17 - long forward stretch

Supplementary Video 25

Description: Syllable 18 - tight left bend

Supplementary Video 26

Description: Syllable 19 - broad rightward curve

Supplementary Video 27

Description: Syllable 20 - long straight forward step

Supplementary Video 28

Description: Syllable 21 - elongated straight run

Supplementary Video 29

Description: Syllable 22 - very long stretched run

Supplementary Video 30

Description: Syllable 23 - narrow straight nose-forward

Supplementary Video 31

Description: Syllable 24 - curved rightward arc

Supplementary Video 32

Description: Syllable 25 - diagonal left sweep

Supplementary Video 33

Description: Syllable 26 - pronounced right C-turn

Supplementary Video 34

Description: Syllable 27 - maximal full-body extension

Supplementary Video 35

Description: Syllable 28 - long low straight extension

Supplementary Video 36

Description: Syllable 29 - elongated slight-right bend

Supplementary Video 37

Description: Syllable 30 - broad straight advance

Supplementary Video 38

Description: Syllable 31 - pronounced S-turn pivot

Supplementary Video 39

Description: Syllable 32 - long straight tail sweep

Supplementary Video 40

Description: Syllable 33 - strong right-bend arc

Supplementary Video 41

Description: Syllable 34 - compact right curl

Supplementary Video 42

Description: Syllable 35 - curved leftward head sweep

Supplementary Video 43

Description: Syllable 36 - compact straight pause

Supplementary Video 44

Description: Syllable 37 - long extended forward rush

Supplementary Video 45

Description: Syllable 38 - tight crouched curl

Supplementary Video 46

Description: Syllable 39 - compact C-turn

Supplementary Video 47

Description: Syllable 40 - compact rounded crouch

Supplementary Video 48

Description: Syllable 41 - tight straight low shuffle

Supplementary Video 49

Description: Syllable 42 - compact straight forward

Supplementary Video 50

Description: Syllable 43 - compact nose-forward push

Supplementary Video 51

Description: Syllable 44 - deep leftward C-turn

Supplementary Video 52

Description: Syllable 45 - long straight slide

Supplementary Video 53

Description: Syllable 46 - pronounced rightward C-turn

Supplementary Video 54

Description: Syllable 47 - sharp right turn pivot

Supplementary Video 55

Description: Syllable 48 - deep leftward stretch-turn
